# An update on the Society for Immunotherapy of Cancer consensus statement on tumor immunotherapy for the treatment of cutaneous melanoma: version 2.0

**DOI:** 10.1186/s40425-018-0362-6

**Published:** 2018-05-30

**Authors:** Ryan J. Sullivan, Michael B. Atkins, John M. Kirkwood, Sanjiv S. Agarwala, Joseph I. Clark, Marc S. Ernstoff, Leslie Fecher, Thomas F. Gajewski, Brian Gastman, David H. Lawson, Jose Lutzky, David F. McDermott, Kim A. Margolin, Janice M. Mehnert, Anna C. Pavlick, Jon M. Richards, Krista M. Rubin, William Sharfman, Steven Silverstein, Craig L. Slingluff, Vernon K. Sondak, Ahmad A. Tarhini, John A. Thompson, Walter J. Urba, Richard L. White, Eric D. Whitman, F. Stephen Hodi, Howard L. Kaufman

**Affiliations:** 1 Massachusetts General Hospital, 55 Fruit Street, Boston, MA 02114 USA; 2 Georgetown University, Washington, DC 20057 USA; 3 University of Pittsburgh, Pittsburgh, PA 15213 USA; 4 St. Luke’s Cancer Center and Temple University, Center Valley, PA 18034 USA; 5 Loyola University Medical Center, Maywood, IL 60153 USA; 6 Roswell Park Cancer Institute, Buffalo, NY 14263 USA; 7 University of Michigan, Ann Arbor, MI 48109 USA; 8 University of Chicago Medical Center, Chicago, IL 60637 USA; 9 Cleveland Clinic, Cleveland, OH 44195 USA; 10 Emory Winship Cancer Institute, Atlanta, GA 30322 USA; 11 Mt. Sinai Medical Center, Miami Beach, FL 33140 USA; 12 Beth Israel Deaconess Medical Center, Boston, MA 02215 USA; 13 City of Hope, Duarte, CA 91010 USA; 14 Rutgers Cancer Institute of New Jersey, New Brunswick, NJ 08901 USA; 15 New York University Cancer Institute, New York, NY 10016 USA; 16 Lutheran General Hospital, Park Ridge, IL 60068 USA; 17 The Sidney Kimmel Comprehensive Cancer Center at Johns Hopkins, Baltimore, MD 21231 USA; 18 Melanoma Research Foundation, Woodcliff Lake, NJ 07077 USA; 19 University of Virginia, Charlottesville, VA 22908 USA; 20 H. Lee Moffitt Cancer Center and Research Institute, Tampa, FL 33612 USA; 21 Cleveland Clinic Taussig Cancer Center, Cleveland, OH 44195 USA; 22 Seattle Cancer Care Alliance, Seattle, WA 98109 USA; 23 Earle A. Chiles Research Institute, Providence Cancer Center, Portland, OR 97213 USA; 24 Carolinas Medical Center, Charlotte, NC 28204 USA; 25 Carol G. Simon Cancer Center, Morristown, NJ 07046 USA; 26 Dana-Farber Cancer Institute, Boston, MA 02215 USA

**Keywords:** Guidelines, Immunotherapy, Melanoma, Treatment

## Abstract

**Background:**

Cancer immunotherapy has been firmly established as a standard of care for patients with advanced and metastatic melanoma. Therapeutic outcomes in clinical trials have resulted in the approval of 11 new drugs and/or combination regimens for patients with melanoma. However, prospective data to support evidence-based clinical decisions with respect to the optimal schedule and sequencing of immunotherapy and targeted agents, how best to manage emerging toxicities and when to stop treatment are not yet available.

**Methods:**

To address this knowledge gap, the Society for Immunotherapy of Cancer (SITC) Melanoma Task Force developed a process for consensus recommendations for physicians treating patients with melanoma integrating evidence-based data, where available, with best expert consensus opinion. The initial consensus statement was published in 2013, and version 2.0 of this report is an update based on a recent meeting of the Task Force and extensive subsequent discussions on new agents, contemporary peer-reviewed literature and emerging clinical data. The Academy of Medicine (formerly Institute of Medicine) clinical practice guidelines were used as a basis for consensus development with an updated literature search for important studies published between 1992 and 2017 and supplemented, as appropriate, by recommendations from Task Force participants.

**Results:**

The Task Force considered patients with stage II-IV melanoma and here provide consensus recommendations for how they would incorporate the many immunotherapy options into clinical pathways for patients with cutaneous melanoma.

**Conclusion:**

These clinical guidleines provide physicians and healthcare providers with consensus recommendations for managing melanoma patients electing treatment with tumor immunotherapy.

**Electronic supplementary material:**

The online version of this article (10.1186/s40425-018-0362-6) contains supplementary material, which is available to authorized users.

## Background

Cutaneous melanoma continues to be a serious public health threat with a slow, but steady increase in annual incidence over the past four decades [1]. In 2017, there were an estimated 87,110 new cases and 9730 deaths due to melanoma in the United States. While melanomas detected early can often be treated by complete surgical excision with good outcomes, the development of metastatic disease, which is associated with reduced survival, is correlated with increasing stage and other high-risk features of the primary tumor [2]. Contemporary systemic therapeutic options for patients with metastatic melanoma include cytotoxic chemotherapy, molecularly targeted therapy, and immunotherapy. Since 2011, the treatment landscape for patients with melanoma has changed considerably with regulatory approval of 11 new drugs and/or combination regimens [3]. Immunotherapy agents in particular have been associated with durable long-term survival in responding patients and have emerged as first-line treatment in most melanoma populations [4].

The immunotherapy agents approved for melanoma include cytokines, such as interferon α2b/pegylated interferon α2b for high-risk adjuvant therapy and high-dose interleukin-2 (IL-2) for metastatic disease; ipilimumab and nivolumab, immune checkpoint inhibitors targeting cytotoxic T lymphocyte antigen 4 (CTLA-4) and programmed cell death 1 (PD-1), respectively for high-risk adjuvant melanoma, and four T cell checkpoint inhibitors for metastatic melanoma, including ipilimumab (anti-CTLA-4), pembrolizumab (anti-PD-1), nivolumab (anti-PD-1) and the combination of ipilimumab/nivolumab; finally, one gene-modified oncolytic virus, talimogene laherparepvec (T-VEC), has been approved for intralesional therapy [5–12]. While the clinical trials supporting regulatory approvals have dramatically changed the melanoma treatment landscape and provided patients and providers with several new options, there is relatively little data for evidence-based decisions in regard to optimal sequencing of these agents, methods or biomarkers to select the right treatment for individual patients, or rigorous information on how best to manage potential adverse events or indicators for optimal duration of therapy. The availability of other therapeutic options, in particular targeted therapy for patients whose melanoma harbors a mutation in BRAF, highlight the importance of having data or consensus agreement from experts in the field on how best to manage patients while waiting for new clinical and clinical trial data to help inform decision-making.

To address the gap in evidence-based data, the Society for Immunotherapy of Cancer (SITC) established a Melanoma Task Force to provide consensus recommendations for clinical decision making for patients with melanoma. SITC is a non-profit professional organization dedicated to improving cancer patient outcomes through the use of cancer immunotherapy. The Task Force consisted of melanoma experts, including physicians, nurses and patient advocates who met in person and communicated through email to consider major issues and provide recommendations related to patient selection, toxicity management, treatment cessation and treatment sequencing. The panel published the first consensus statement in 2013 [4], and this publication represents an update based on more recent assessment of the peer-reviewed literature and clinical experience of the expert Task Force participants. These recommendations are not intended to supplant sound clinical judgment but to provide clinicians who care for melanoma patients the most current thinking on how experts integrate immunotherapy into the treatment armamentarium for patients with advanced cutaneous melanoma.

## Methods

### Consensus statement policy

SITC utilized the National Academy of Medicine (formerly Institute of Medicine) March 2011 Standards for Developing Trustworthy Clinical Guidelines as a model for organizing and preparing this consensus statement [13]. These standards include a transparent process for guideline development and funding, managing and reporting conflicts of interest, maintaining a multidisciplinary and balanced group composition, establishing an evidence-based foundation for recommendations and rating system to assess the strength of the evidence, reporting the results through a peer-reviewed publication and publicly available website, and updating the statement as changes in the field warrant revisions.

The Melanoma Task Force was established through SITC in 2011, with additional panel members added as necessary (Additional file 1). A Steering Committee led a panel discussion to develop clinical treatment guidelines considering four basic issues for each immunotherapy agent in current clinical practice: patient selection, toxicity management, assessment of response, and therapy sequencing and combinations. The in-person meeting was supplemented by email voting on several issues due to the rapid development of new findings and drug approvals for melanoma over the last 2 years. Full consensus recommendations can be found on the SITC website [14]. Owing to disparities in drug approval and availability in some countries, this panel focused solely on drugs approved by the U.S. Food and Drug Administration (FDA). An advance copy of this manuscript was submitted to the FDA for comment before submission for publication. The panel also recognized that the AJCC Cancer Staging Manual, 8th Edition has been released but the clinical trial data reviewed utilized earlier versions of AJCC staging; as such, the recommendations presented in this manuscript were largely based on 7th edition staging criteria. However, recommendations that extrapolate clinical trial data using 7th edition staging criteria in the setting of completion lymph node dissection (CLND), are made to the current era using the 8th edition staging system in the non-CLND era where appropriate.

### Consensus panel and conflicts of interest

Potential panel members were solicited from the SITC membership and supplemented with non-member melanoma multidisciplinary experts, clinicians and groups in the U.S. expected to be affected by the development of any recommendations, including patients, patient advocates and nurses. Panel members were screened for conflicts of interest using the SITC disclosure form, which mandates full financial and other disclosures including relationships with commercial entities that might reasonably be expected to have direct regulatory or commercial impact resulting from the publication of this statement. Disclosures of potential conflicts of interest are noted in this manuscript. No commercial funding was used to support the consensus panel, literature review or preparation of the manuscript.

The consensus panel convened in June 2016 in accordance with the National Academy of Medicine and SITC guidelines to review results from a previously distributed questionnaire collecting information on the participants’ role in the care of patients with melanoma, primary clinical focus, experience with FDA-approved agents used for immunotherapy treatments, and current practices in the use or recommendation for use of such agents. Additional questionnaires were distributed electronically after the meeting to collect further information, including a final questionnaire in the late summer of 2017. The final consensus statement was made available to the entire SITC membership for open comment and these comments were considered for the final manuscript and are available in supplementary materials (see Additional file 2) and online at the SITC website [14].

### Literature review and rating system

A search of the scientific literature (using the MEDLINE database) was conducted focusing on current therapeutic approaches in humans. The search terms included “melanoma” and “interferon”, “interleukin-2”, “ipilimumab”, “vemurafenib,” “BRAF,” “dabrafenib, dacarbazine, temozolomide”, “pembrolizumab”, “nivolumab”, “PD-1/PD-L1”, “combination”, “talimogene laherparepvec”, “adverse event”, and “toxicity”. The search resulted in retrieval of nearly 2400 manuscripts, which were screened by Task Force members to include only papers with clinically relevant information and removing duplicates from independent searches, resulting in a final bibliography of 1643 manuscripts (see Additional file 3) catalogued using EndNote X5.0.1. The bibliography was supplemented with additional literature identified by the panel, as appropriate. Literature was graded into three levels of evidence, as previously described [4]. Level A evidence is based on strong supporting evidence, such as data derived from appropriately powered prospective, randomized clinical trials or meta-analyses; Level B is based on moderate supporting data, such as uncontrolled, prospective clinical trials; and Level C is based on weaker supporting data, such as retrospective reviews and case reports.

## Consensus recommendations

The Task Force considered individual melanoma stages independently and provided the following consensus recommendations described by stage of disease. These recommendations were based on data available for AJCC version 7 staging guidelines; where appropriate, modifications relevant for AJCC version 8, which became active in January 2018, are noted. The majority of the immunotherapy trials on which the following recommendations are based included patients with ECOG Performance Status 0 or 1. These guidelines are intended to assist clinicians in critical decision-making for patients with melanoma and should not supplant clinical judgment for individual patient management.

### Immunotherapy for stage II melanoma

#### Initial assessment

Patients with stage II melanoma have an excellent overall survival (OS) of 80% or better provided the primary tumor is completely excised [2]. A subset of tumors, characterized as deep (Breslow thickness > 4 mm), and/or with ulceration, and possibly those with a high tumor mitotic rate (≥1 per mm^2^), are considered at higher risk for recurrence [15]. Practically speaking, using both AJCC 7th and 8th additions, Stage IIB and IIC are considered higher risk. The panel discussed at length the changing landscape with respect to how to define high risk and when to consider further intervention with the goal of preventing tumor relapse. There was unanimous agreement that all stage II patients should have a comprehensive diagnostic workup and be reviewed by a multidisciplinary team, including physicians with expertise in surgical oncology, medical oncology, dermatology and dermatopathology to accurately determine tumor stage and estimate the risk of melanoma recurrence for individual patients. This workup should include sentinel lymph node biopsy information, as appropriate [16].

#### Consensus management of stage II melanoma

The panel considered the therapeutic approach to stage II melanoma should be based on an assessment of risk for tumor recurrence or metastatic spread but recognized that there is considerable controversy in how to determine risk stratification. Further, changes in the AJCC staging system and emerging data using a variety of histologic and molecular assays for risk assessment have made firm recommendations challenging. For the purposes of our discussions, we defined high risk stage II as patients with tumors > 4 mm in depth (with or without ulceration) or tumors > 2–4 mm with ulceration. While this definition may change with further prospective data, the general approach to patient management can be considered based on clinical assessment of higher versus lower risk.

**Fig. 1:**
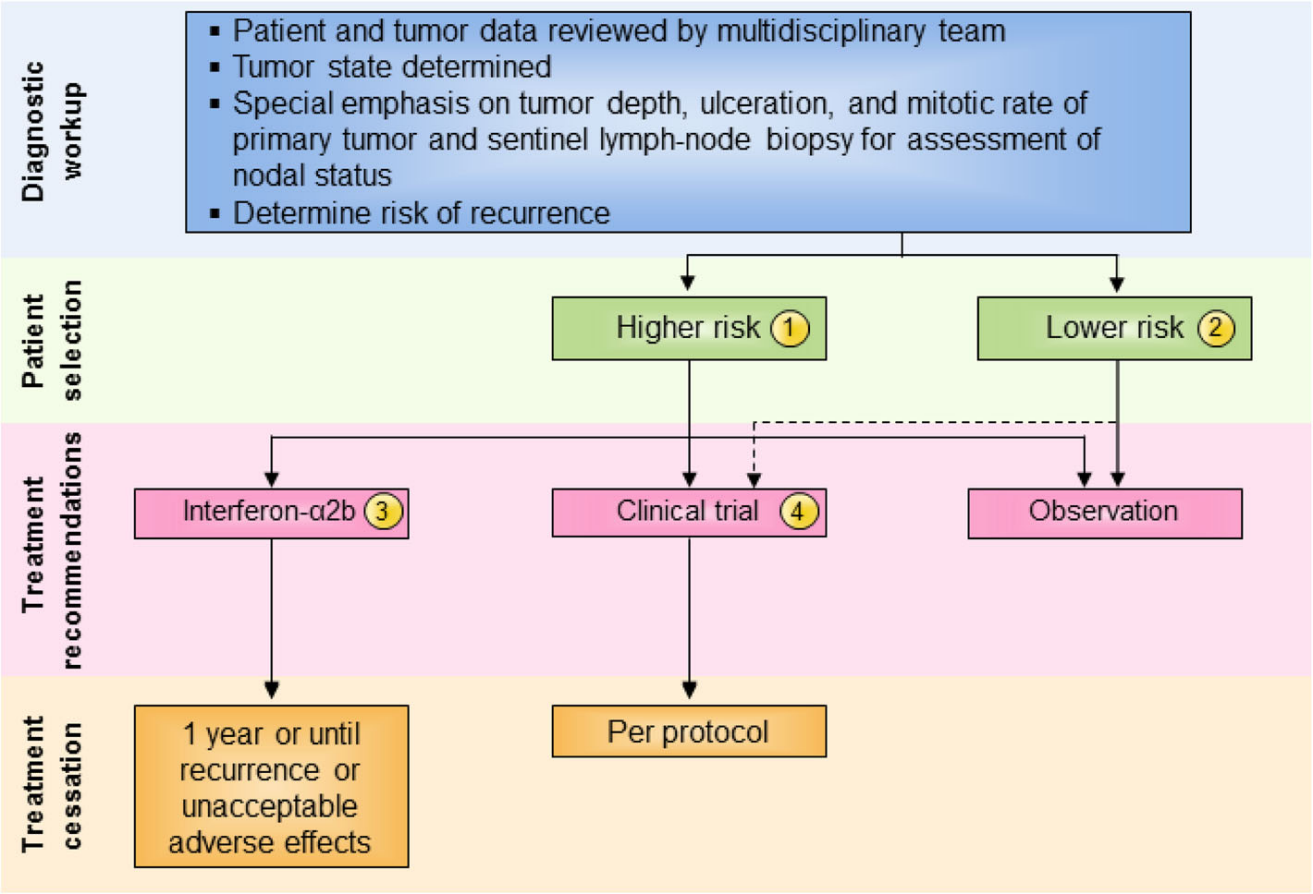
Stage II melanoma immunotherapy treatment algorithm. All treatment options shown may be appropriate, and final selection of therapy should be individualized based on patient eligibility and treatment availability at the physician’s discretion. These algorithms represent consensus sequencing suggestions by the panel. (1) High-risk disease is defined as tumors > 4 mm in depth (with or without ulceration) or > 2–4 mm with ulceration. There is limited consensus on adjuvant therapy for this group with 10% of the panel recommending interferon-α2b, 20% recommending observation, 45 and 15% recommending therapeutic and/or biomarker-based clinical trial participation, respectively, and no panelists recommending pegylated-interferon-α2. (2) There is no evidence that immunotherapy is useful in patients with lower risk stage II melanoma, although the panel did recommend clinical trial participation, if available. Protocol-specific eligibility would need to be followed to select appropriate study candidates. (3) Patients should have a good performance status without evidence of significant depression, psychiatric history or underlying autoimmune disease to be considered for interferon-α2b. There are limited data available on interferon-α2b as treatment for stage II disease. (4) Clinical trials were the preferred treatment recommendation for patients with stage II disease associated with higher risk of tumor recurrence

There was general agreement that patients with lower risk stage I and IIA melanoma can be observed and that there is no evidence that currently warrants treatment of these patients (Fig. 1). The panel, however, was divided on the role of immunotherapy for patients with higher-risk stage IIB-C melanoma (see Fig. 1) and recognized the limited Level A data available to inform clinical decision-making. The panel did consider emerging Level B data suggesting new recommendations are needed for high-risk stage II melanoma patients. Whereas before the majority of the panel recommended that high-risk patients be treated with standard 1-year high dose interferon-α2b, now a small majority (55%) recommend enrollment onto a clinical trial - either unselected or selected by a biomarker known to be associated with either risk (prognostic) or responsiveness to the therapy (predictive) - as a preferred option for these patients. Among panel members who did not recommend a clinical trial, twice as many recommended observation (20%) as did the pursuit of standard of care adjuvant interferon α-2b (10%). This is a reflection of a number of factors including: 1) improved systemic therapy for recurrent, metastatic disease [4]; 2) acknowledgment of the limitations of the AJCC staging system to identify those at high and low risk of recurrence (e.g., a significant number of patients with low risk [by currently available methods] melanoma will still die of disease [15]); and 3) emerging, as yet non-validated biomarkers, which may better identify patients at greatest risk of recurrence (e.g., ulceration, gene expression profile, circulating tumor DNA) [15, 17, 18]. None of the panel members recommended treatment with pegylated interferon-α2b for patients with stage II disease.

Patients with stage IIB or IIC melanoma who are treated with interferon-α2b should have a good performance status without evidence of significant depression or psychiatric history or underlying autoimmune disease [4]. The data to support the use of adjuvant, high-dose interferon-α2b are controversial and many studies did not incorporate required sentinel lymph node biopsy into the study eligibility complicating the interpretation. In a prospective study, 499 patients with melanoma Breslow thickness > 1.5 mm, and without clinically detectable lymph node metastases, were randomly assigned to 18 months of subcutaneous interferon-α2b or observation [19]. Patients treated with interferon-α2b demonstrated a significant improvement in relapse-free survival (RFS) (*P* = 0.038) and a trend toward improved OS (*P* = 0.059). In another trial, 855 patients were randomly assigned to observation or 4 weeks induction interferon-α2b followed by 1 or 2 years of interferon-α2b maintenance therapy [20]. The study investigators reported an improvement in RFS for patients who received 1 year of maintenance interferon-α2b (hazard ratio [HR] 0.77, 95% Confidence interval [CI]: 0.63–0.96; *P* = 0.034), but no benefit in OS (HR 0.91, 95% CI: 0.74–1.10; *P* = 0.642). Several other prospective randomized trials examined interferon-α2b at a variety of doses and treatment schedules in patients with stage II melanoma, but none has demonstrated a survival benefit [5, 21–25]. A recently reported phase 3 randomized study in 1150 patients with resectable melanoma (T2bN0, T3a-bN0, T4a-bN0, and T1-4N1a-2a) who were randomly assigned to receive intravenous (IV) high-dose interferon-α2b for 5 days every week for 4 weeks or observation, produced equivalent 5-year RFS rates between groups. Moreover, 4 weeks of IV interferon-α2b resulted in higher rates of treatment-related grade 3 and higher toxicities (57.9% vs. 4.6%; *P* < .001) and worsened quality of life [26]. These studies are complicated by a lack of a standardized definition of ‘high risk for relapse’, 23 different interferon-α2b dosages/formulations/schedules were evaluated, and in some cases, the inclusion of other drugs in combination. Thus, the efficacy of interferon in sentinel node negative stage II melanoma patients remains unresolved. To date, there are no data with ipilimumab, nivolumab, pembrolizumab, or BRAF-targeted therapy (either single-agent BRAF inhibitors or combined BRAF/MEK inhibitor therapy) to justify the use of these agents/regimens in patients with stage II melanoma. However, data from planned clinical trials may provide additional information to guide the use of the anti-PD1 agent pembrolizumab in this setting.

### Immunotherapy for stage III melanoma

**Fig. 2:**
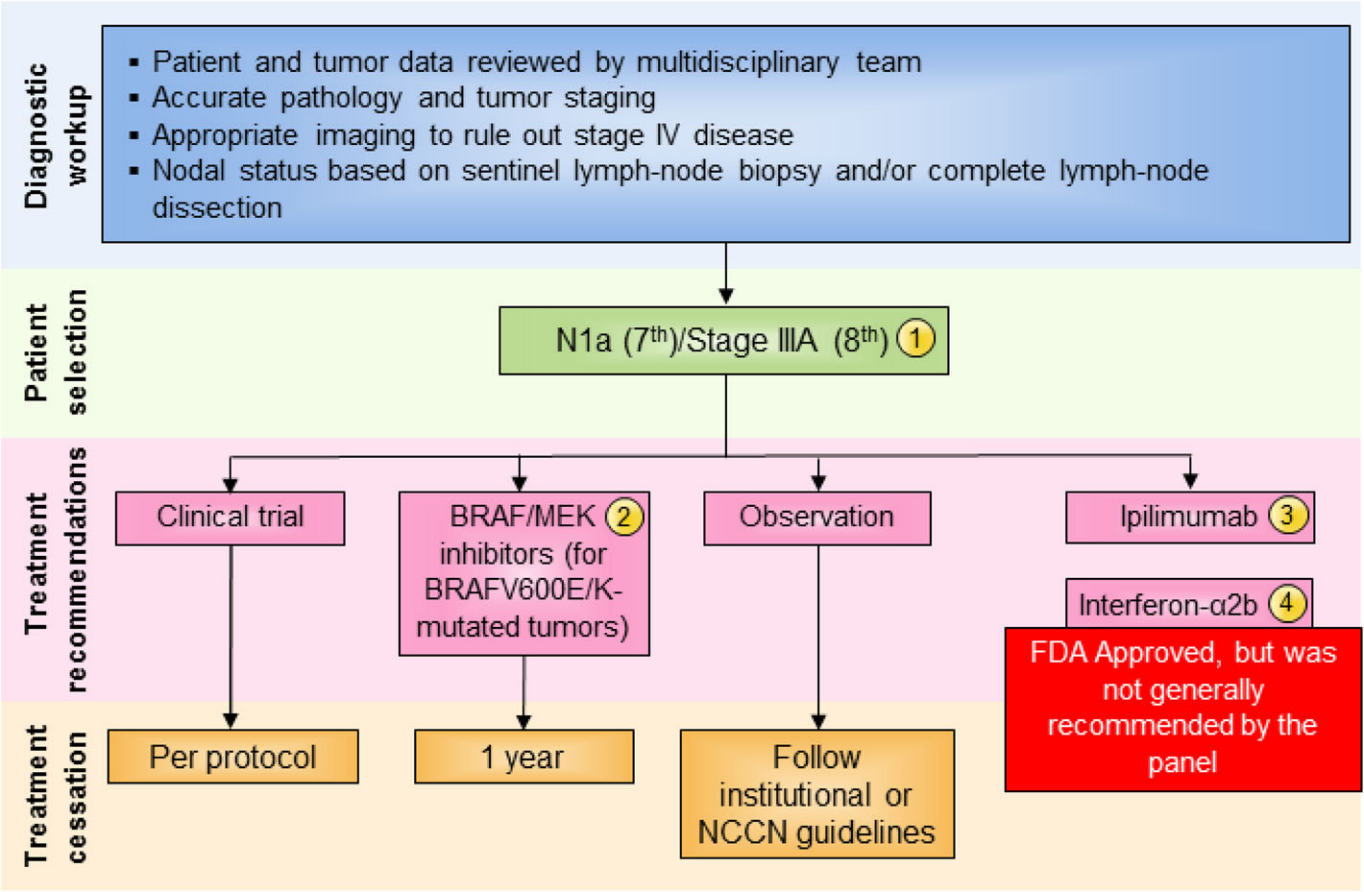
Stage III N1a (7th)/Stage IIIA (8th) melanoma immunotherapy treatment algorithm. The consensus of the panel was to separate Stage III N1a (based on AJCC 7th edition) and Stage IIIA (AJCC 8th) from other Stage III subsets based on lower risk of metastatic potential. However, a minority (30%) felt that all Stage III patients should be treated similarly. All treatment options shown may be appropriate and final selection of therapy should be individualized based on patient eligibility and treatment availability at the physician’s discretion. These algorithms represent consensus sequencing suggestions by the panel. (1) There are limited data on the role of adjuvant therapy following sentinel lymphadenectomy alone, which is anticipated to become more common. (2) There is Level A evidence to support the use the combination of dabrafenib and trametinib in patients with BRAF V600E/K mutant, Stage III melanoma independent of the volume of lymph node involvement or the number of lymph nodes involved. (3) Level A data supporting the use of nivolumab over ipilimumab was demonstrated in patients with Stage IIIB to IV resected melanoma and did not include patients with Stage IIIA (based on 7th) disease. Ipilimumab 10 mg/kg dosing was supported by a minority of panelists (10%), however, subset analysis suggests that the risk: benefit ratio for patients with Stage IIIA melanoma does not support its use in Stage IIIA patients at this time. (4) There are level A data that 1 year interferon-α2b is associated with improvement in RFS and, while this therapy was generally recommended by the consensus panel previously, only two panelists recommended considering this therapy. There are level B data to support a benefit in RFS for pegylated-interferon-α2b in patients with N1a disease and in patients with ulceration of the primary tumor site; however, no panelists considered this a reasonable option for these patients. Abbreviations: LDH, lactate dehydrogenase; NCCN, National Comprehensive Cancer Network; RFS, recurrence-free survival

Stage III comprises a heterogeneous group of patients with 5-year survival rates ranging from 30 to 80% [15]. While the previous consensus statement considered stage III patients as a single group, the Task Force strongly believed that, in patients with microscopic metastasis to a single lymph node (stage N1a), especially when the node has been excised by sentinel lymphadenectomy, cancer behaves differently than in patients with more extensive lymph node involvement (stages N1b-3). In the updated recommendations, patients with N1a disease, in accordance with the AJCC 7th edition were considered as a distinct subset; management recommendations by nodal staging are shown in Fig. 2. With the recent publication and adoption of the 8th edition of AJCC, which strived to identify a group of Stage III patients with significantly lower risk, the Task Force considered Stage IIIA (per AJJC 8th Ed.) to have lower risk of tumor recurrence compared to Stage IIIB-D. The management of stage III disease has also been complicated by recent data showing that, while immediate completion lymph node dissection was associated with a decreased rate of lymph node basin recurrence and increased disease-free survival in sentinel node-positive patients, there was no improvement in melanoma-specific survival [27]. These findings along with the availability of more effective systemic treatment will change the management for sentinel node-positive patients, although all of the reported clinical trials of adjuvant therapy mandated completion lymph node dissection as a key eligibility criterion for study participation. Thus, the recommendations for stage III management should be considered carefully in light of these recent developments.

#### Initial assessment

In all patients with stage lll melanoma, a diagnostic workup should be performed and reviewed by a multidisciplinary team for patient and tumor characteristics. Complete tumor staging information should be assessed, including pathological features of the primary tumor and any involved lymph nodes, as well as BRAF mutation testing. In addition, whole-body imaging (see Table 1) and performance status assessment should be completed prior to making treatment decisions. Nodal status should be determined based on physical examination and sentinel lymph node biopsy (SNB) with or without subsequent completion lymphadenectomy if SNB is positive. The consensus panel identified five potential immunotherapy agents with potential clinical benefit in the adjuvant therapy of patients with stage III melanoma: interferon-α2b, pegylated interferon-α2b, ipilimumab, pembrolizumab, and nivolumab [6, 28–31]. Furthermore, the consensus panel noted that the combination of the BRAF and MEK inhibitors, dabrafenib and trametinib, respectively, was recently shown to be superior to placebo in patients with stage III melanoma with BRAF V600E/K mutations; these data provide the first evidence for significant RFS and OS benefit of a targeted antitumor therapy that does not fit the putative immunotherapy approach and can be considered for patients with tumors harboring BRAF mutations [32].

**Table 1:** Clinical Issues in Tumor Immunotherapy for Cutaneous Melanoma

Clinical Issue	Current Consensus Recommendations
Biomarker Status	• The panel recognized the importance of identifying predictive biomarkers to aid in clinical decision-making • At present there are no validated biomarkers that reliably predict response to individual therapeutic agents • There is considerable interest in PD-L1 expression, mutation burden, lymphocyte infiltration, interferon-γ and related cytokine gene signatures as potential biomarkers • There are data suggesting higher response rates to monotherapy, but not combination therapy, with T cell checkpoint inhibitors when PD-L1 expression is increased but the panel does not recommend PD-L1 status be used outside of clinical trials
Laboratory Assessment	• Immunotherapy is associated with significant irAEs that require laboratory monitoring before and during active treatment • Clinicians should be alert for irAEs during therapy and for several months after stopping treatment • All panelists agreed that baseline and routine labs should include complete blood count, liver enzymes, metabolic panel, serum LDH and thyroid function studies (free T4, TSH) • Additional hormone levels should be assessed in patient with suspected treatment-related hypophysitis (free T4, TSH, ACTH, morning cortisol, cosyntropin stimulation test, LH, FSH, testosterone, prolactin) and early endocrinology referral • The frequency of laboratory testing was more controversial with most panelists recommending testing prior to each infusion for most drugs and less frequent surveillance during follow-up
Imaging Guidelines	• Confirming disease response/progression may be challenging with immunotherapy due to the delayed kinetics of response and induction of local inflammation • The panel (100%) recommends whole body imaging for melanoma patients treated with immunotherapy prior to starting and at regular intervals no more than 12 weeks apart while disease persists • A majority of the panel recommends imaging with CT scans of the chest, abdomen and pelvis and MRI of the brain • A minority recommend initial imaging with PET scans • Imaging should continue after complete responses at regular intervals for five years to identify recurrence
Treatment Cessation	• Since the kinetics of response to immunotherapy may be delayed decisions to stop treatment can be challenging • The panel recommended stopping treatment for any unresolved or recurrent high grade adverse event or when disease progression is confirmed by two independent imaging scans or clinical deterioration • Pseudo-progression has been reported for checkpoint inhibitors and T-VEC but is rare for interferon and IL-2; most panelists suggested that treatment with interferon or IL-2 should be stopped with any sign of disease progression • Repeat imaging within 1–2 months was recommended to confirm response or progression when pseudo-progression is suspected • Minority opinions included considering surgical resection for incomplete responses and tumor biopsy for equivocal cases

*Abbreviations*: *ACTH* adrenocorticotropic hormone, *CT* computed tomography, *FSH* follicle stimulating hormone, *LH* luteinizing hormone, *MRI* magnetic resonance imaging, *PD-L1* programmed cell death 1 ligand, *PET* positron emission tomography, *TSH* thyroid stimulating hormone

#### Consensus management of microscopic single node disease (stage N1a – AJCC 7th; stage IIIA – AJCC 8th)

The majority of the panel (70%) recognized that patients with microscopically involved lymph nodes (N1a disease) represents a different population from those with macroscopic nodal disease (N1b and N2–N3 disease) and agreed that the AJCC 8th edition takes this into account by redefining Stage IIIA as being associated with a lower risk than in the AJCC 7th edition. However, whereas the majority (52%) of the former panel in 2014 recommended a standard 1-year course of interferon-α2b for adjuvant therapy of patients with microscopic nodal disease, only a small number recommended this therapy in this update. Rather, the majority of the panel (58%) recommended a clinical trial, 10% recommended observation, 5% ipilimumab (10 mg/kg), and 10% adjuvant interferon-α2b, if a clinical trial was not available. No panelists recommend pegylated interferon-α2b or ipilimumab given at 3 mg/kg (see Fig. 2).

There is one prospective randomized clinical trial demonstrating a benefit in RFS for patients with microscopic nodal disease treated with pegylated interferon-α2b [6]. A post-hoc analysis of that trial also suggested patients with ulcerated primary tumors might derive more clinical benefit from pegylated interferon-α2b [33]. In this analysis, patients with ulceration of their primary melanoma (*n* = 849) were compared to patients without ulceration of their primary melanoma (*n* = 1336), and patients with ulceration demonstrated a significant improvement in RFS (*P* = 0.02), distant metastasis-free survival (*P* < 0.001) and OS (P < 0.001). The analysis also found that the greatest reduction in risk was seen in patients with ulcerated primary melanomas who were classified as stage IIb–IIIN1, demonstrating a HR of 0.58 for OS benefit (*P* < 0.0001) [34]. Thus, patients with ulcerated primary tumors and those with microscopic nodal disease could consider pegylated interferon-α2b based on this Level B data, although further evaluation of this regimen is ongoing in an EORTC trial.

Ipilimumab has been studied in patients with stage III melanoma in a prospective clinical trial (EORTC 18071), which randomized 951 patients to either placebo or ipilimumab, given at 10 mg/kg induction (4 doses every 3 weeks) followed by maintenance (every 12 weeks for up to 3 years) [30]. With a median follow up of over 5 years, ipilimumab was associated with improved RFS compared to patients treated with placebo (median 27.6 vs. 17.1 months, HR 0.76, 95% CI: 0.64–0.89; *P* = 0.0008) and OS (5-year 65% vs. 54%, HR 0.72, 95% CI: 0.58–0.88; *P* = 0.001). However, in subgroup analysis, patients with stage IIIA disease, despite being required to have one or more nodal metastases at least 1 mm in size, had no evidence of benefit (HR 0.98, 95% CI: 0.46–2.09) [30]. Thus, there was hesitation in considering adjuvant ipilimumab for patients with lower risk, stage III disease in light of known toxicity, although adjuvant ipilimumab was recommended by a minority of the panel (10%).

In an older trial, which included patients with completely resected stage IV or high-risk stage III melanoma, adjuvant granulocyte-macrophage colony stimulating factor (GM-CSF) did not demonstrate improvements in RFS or OS in a randomized, placebo-controlled phase 3 study [35]. GM-CSF, an immunomodulatory agent with pleiotropic and sometimes opposing effects on antitumor immunity, remains investigational for any stage of melanoma, although its incorporation into an oncolytic virotherapy for intratumoral administration is approved for advanced melanoma, and its role in combination immunotherapy appears promising [12, 36].

Although immunomodulatory therapy is the only intervention that had ever shown promise in the adjuvant therapy of melanoma, there is now evidence that molecularly-targeted therapies can benefit patients with resected high-risk melanoma whose tumor cells carry an activating BRAF mutation. A trial of dabrafenib and trametinib given at standard doses (CombiAD), randomized 870 patients (1:1) to either the combination of dabrafenib and trametinib (D/T) or placebo for 1 year. This trial excluded patients with stage IIIA (N1) with a < 1 mm metastatic nodal deposit. With a median follow up of 2.8 years, D/T was associated with improved RFS (HR 0.47; 95% CI: 0.39–0.58, *P* < 0.001) and OS (HR 0.57; 95% CI: 0.42–0.79, *P* < 0.001) compared to placebo. Moreover, there were no additional safety concerns that arose with D/T that had not previously been seen in patients with unresectable or stage IV melanoma [32]. While this combination is not considered immunotherapy, inhibitors of BRAF and associated pathways in the tumor cell have been shown to have immunomodulatory properties that contribute to their activity. For these patients, the choice between molecularly targeted and immune checkpoint-based adjuvant therapy remains unclear, as direct comparisons have not yet been made. However, benefit was seen across all AJCC 7th (and by extrapolation 8th) edition stage III subgroups, and this combination can be considered for any patient with stage III, BRAF^V600E/K^-mutant melanoma.

#### Consensus management of macroscopic nodal disease (stage N1b/c, N2b/c, N3b/c in 7th edition or stage IIIB-IIID in 8th edition)

**Fig. 3:**
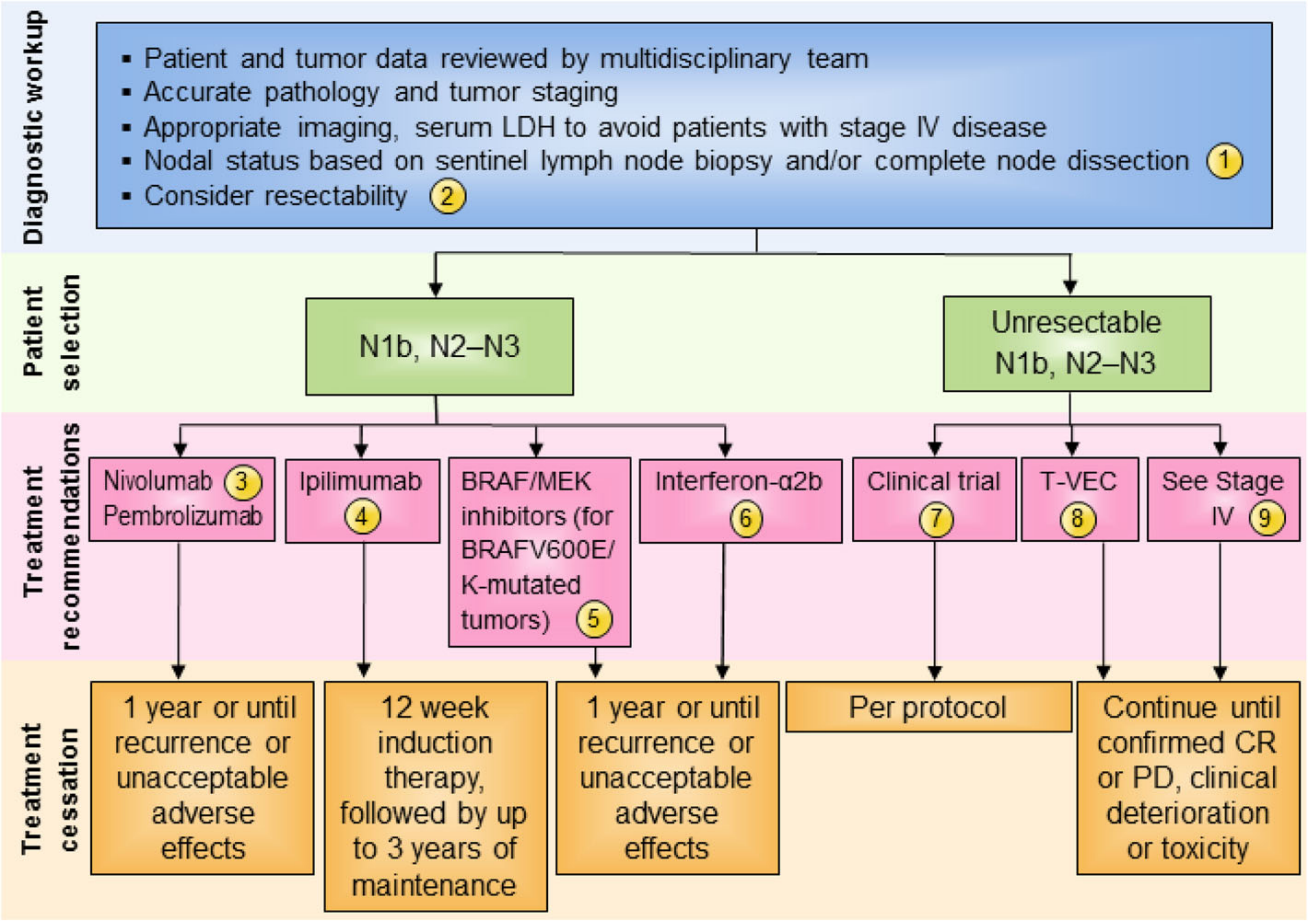
Stage III N1b-3 (AJCC 7th)/Stage IIIB-D (AJCC 8th) melanoma immunotherapy treatment algorithm. The consensus of the panel was to separate Stage III N1a (based on AJCC 7th edition) and Stage IIIA (AJCC 8th) from other Stage III subsets based on lower risk of metastatic potential. However, a minority (30%) felt that all Stage III subsets should be treated similarly. All treatment options shown may be appropriate and final selection of therapy should be individualized based on patient eligibility and treatment availability at the physician’s discretion. These algorithms represent consensus sequencing suggestions by the panel. (1) There are limited data on the role of adjuvant therapy following sentinel lymphadenectomy alone. (2) After evaluation by multi-disciplinary team with surgical oncology, if complete resection is possible patients should undergo resection followed by adjuvant therapy listed. If the tumor is considered unresectable, a different treatment paradigm should be followed. (3) In patients with Stage IIIB-IV resected melanoma, there is Level A evidence supporting the use of nivolumab over ipilimumab and pembrolizumab over placebo for stage IIIB-C and IIA patients with micrometastases > 1 mm. Accordingly, nivolumab or pembrolizumab were supported by 46% of the panel. (4) Ipilimumab at 3 mg/kg was supported by a minority of panelists (8.3%). (5) There is Level A evidence to support the use the combination of dabrafenib and trametinib in patients with BRAF V600E/K mutant, Stage III melanoma. (6) While there are Level A data that 1 year interferon-α2b is associated with improvement in RFS, no panelists recommended considering this therapy for this patient population. (7) Overall, the majority of panelists recommended a clinical trial, if available. (8) The majority of the panelists have had experience with T-VEC, and half of respondents said they would recommend T-VEC for first-line treatment for limited disease burden, and a significant minority (39%) would consider T-VEC for patients with locoregional disease. (9) Unresectable disease could be managed by options available for stage IV patients (see Fig. 4). Abbreviations: CR, complete response; LDH, lactate dehydrogenase; NCCN, National Comprehensive Cancer Network; PD, progressive disease; RFS, recurrence-free survival, TVEC, talimogene laherparepvec

Patients with macroscopic involvement of a single or multiple lymph nodes (stage N1b and N2b–N3 disease per AJCC 7th Edition; or Stages IIIB-IIID in AJCC 8th Edition) are at significant risk for melanoma recurrence. The panel recommendations for these melanoma patients are detailed in Fig. 3. Whereas the majority of the panel in 2014 recommended that these patients consider 1 year of interferon-α2b treatment (73%) [4], in the current setting, the majority of panelists recommended either a clinical trial (56%), or if a trial is not available then adjuvant nivolumab based on the results of the CheckMate 238 trial, or adjuvant pembrolizumab based on the results of the recent phase III clinical trial (46% of panelists) [31, 32, 37]. A minority of panelists would consider adjuvant ipilimumab (8%) based on the results of the EORTC 18071 trial [33]. For patients whose tumor harbors a BRAF V600E/K mutation, combination dabrafenib/trametinib may be preferred over immunotherapy since the impact of adjuvant checkpoint inhibitors on the management of subsequent disease progression is not known. Of note, no panelists recommend pegylated interferon-α2b for patients with resected macroscopic nodal disease, and only one panelist considered high-dose interferon-α2b as an option if a clinical trial was not available.

CheckMate 238 is a phase 3 trial that randomized 906 patients with resected stage IIIB-IV melanoma to either 1 year of nivolumab (3 mg/kg every 2 weeks) or ipilimumab (10 mg/kg every 3 weeks for 4 doses, followed by every 12 weeks). With minimum follow-up of 18 months, the trial met its primary endpoint showing that nivolumab was associated with an improved RFS compared with ipilimumab (RFS at 12 months 70.5% vs. 60.8% for nivolumab and ipilimumab, respectively; HR 0.65; CI: 0.51–0.83; *P* < 0.001). Furthermore, the rate of treatment-related grade 3–4 toxicity was 14.4% with nivolumab vs. 42.6% in patients treated with ipilimumab [31]. OS data were immature and not reported. The data from this trial led to the FDA-approval of nivolumab in patients with resected Stage III melanoma.

More recently, a prospective, double-blind phase III clinical trial was conducted in patients with resected, high-risk stage III melanoma. In this study patients were eligble if they had stage IIIB or IIIC, while a subset of patients with stage IIIA were also included if they had at least one micrometastasis measuring > 1 mm. The trial randomly assigned 514 patients to treatment with 200 mg of pembrolizumab and 505 patients to placebo every 3 weeks for 1 year [37]. In this study, patients were stratified by cancer stage and geographic location. At a median follow-up of 15 months, pembrolizumab was associated with significantly longer recurrence-free survival compared to placebo in the intention-to-treat population (75.4% [95% CI: 71.3–78.9] vs. 61.0% [95% CI: 56.5–65.1]; HR for recurrence or death, 0.57 [98.4% CI: 0.43–0.74; *p* < 0.001]). In a cohort of 853 patients with PD-L1-positive tumors, the 1-year rate of recurrence-free survival was 77.1% in the pembrolizumab treated group compared to 62.6% in the placebo group (HR 0.54; 95% CI: 0.42–0.69). Grade 3 or greater adverse events were observed in 14.7% of patients treated with pembrolizumab - with one treatment-related death attributed to myositis - versus 3.4% in patients treated with placebo.

In light of these newer data, patients with resected stage IIIB, IIIC, and IV melanoma could consider several options, and the panel considered anti-PD-1 antibody therapy with either nivolumab or pembrolizumab (46%), ipilimumab at 3 mg/kg (8%), D/T in BRAF mutant patients^1^ (13%), or high-dose interferon (4%) as acceptable recommendations. Almost one third of the panel members (29%) were unable to make a specific recommendation. These members suggested using either anti-PD-1 therapy or D/T, while others preferred the use of D/T if the tumor was BRAF mutant or enrollment onto a clinical trial incorporating ipilimumab at 3 mg/kg. The recommendation to use low dose ipilimumab is supported by data from the phase III U.S. Intergroup E1609 study in which patients with resected high-risk melanoma were treated with interferon-α, ipilimumab at 10 mg/kg or ipilimumab at 3 mg/kg; while there was no obvious difference in recurrence-free survival between the two ipilimumab cohorts (although no formal statistical comparison was performed), there was a significant increase in toxicity reported for the 10 mg/kg cohort compared with 3 mg/kg [38]. No panelists endorsed observation as a clinical option.

#### Consensus management of unresectable stage III/IV melanoma with injectable lesions

**Fig. 4:**
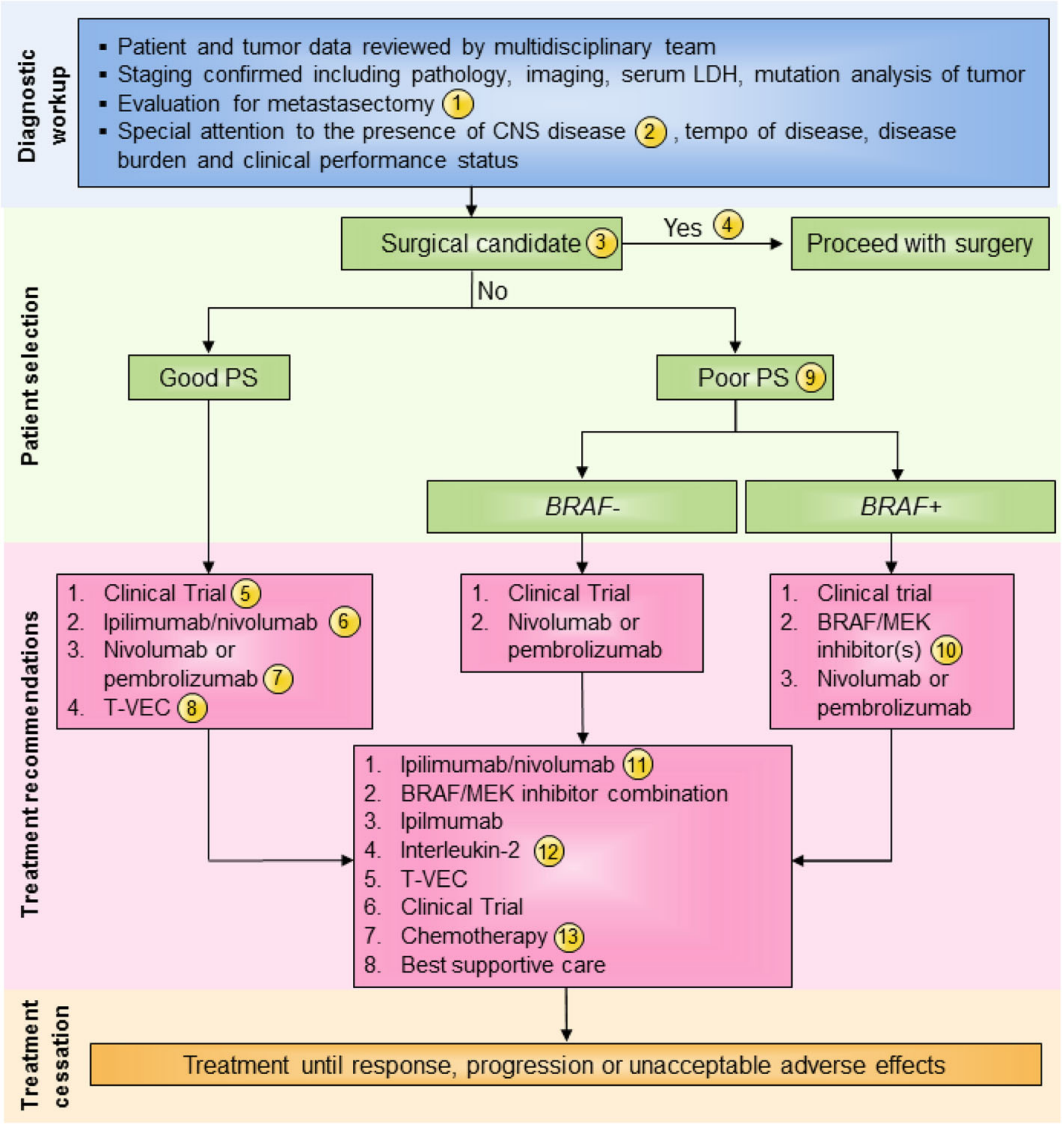
Stage IV melanoma immunotherapy treatment algorithm. All treatment options shown may be appropriate and final selection of therapy should be individualized based on patient eligibility and treatment availability at the physician’s discretion. These algorithms represent consensus sequencing suggestions by the panel. The panel recommended all patients be evaluated with full body imaging, histopathology review, serum LDH, and tumor mutation analysis with emphasis on BRAF mutations. Other factors to be considered in selecting appropriate treatment should include performance status, burden and tempo of disease and presence of CNS metastases. (1) All patients should be evaluated for resection by a multi-disciplinary team including surgical oncology before and after immunotherapy treatment, although the role of surgery is changing and may be appropriate for patients with solitary pulmonary lesions where complete extirpation is possible; each case must be individualized. (2) All patients should have an MRI of the brain prior to treatment to rule out or manage CNS metastasis. (3) There was level B data for a clinical benefit with surgical resection when complete excision of all disease is possible although first-line surgical resection was a minority opinion of the panel. (4) As determined by an experienced surgical oncologist, patient is eligible to receive surgical intervention as first-line treatment. (5) Immunotherapy was recommended for any patient with a good performance status regardless of *BRAF* mutation status and provided that any CNS disease was treated and controlled. Clinical trial was the favored first line approach by the panel. 6) In the absence of an appropriate clinical trial, the panel recommended combination ipilimumab and nivolumab based on the high response rates reported. This may also be preferred for patients with CNS disease with a minority of panelists (33.3%) recommending stereotactic radiation prior to systemic therapy for CNS lesions (7) Next, the panel recommended single agent anti-PD-1 therapy (pembrolizumab or nivolumab). The panel considered these agents to have the same therapeutic efficacy and treatment selection could be based on physician experience and patient preference. (8) The panel also recommended T-VEC in patients with accessible tumor for injection and limited visceral tumor burden. This option may be especially appropriate for elderly patients and those not eligible for checkpoint inhibitors. (9) Patients with poor performance status were not considered good candidates for combination immunotherapy and BRAF mutation was an important factor for determining therapeutic planning. Most panelists considered clinical trials to be the most important option in these patients, if available. In those patients without a BRAF mutation, the next option should be single agent anti-PD-1 therapy (pembrolizumab or nivolumab). (10) In patients with poor performance status and a BRAF mutation who are not eligible or whose tumors progress after a clinical trial, treatment with a BRAF and/or MEK inhibitor therapy is indicated. This option was also considered appropriate for patients with uncontrolled CNS disease. Single agent anti-PD-1 treatment could be considered if disease progression occurs after targeted therapy. (11) In patients with disease progression following the recommendations, management should be carefully considered. If patients can tolerate treatment, ipilimumab/nivolumab should be considered. If patients have a BRAF mutation and have not been treated with BRAF/MEK inhibitors previously these can be considered. Ipilimimab monotherapy and high-dose IL-2 can also be considered in these patients. (12) Patients should have a good PS and otherwise qualify for IL-2 administration per local institutional guidelines. (13) Dacarbazine is the only approved chemotherapy agent but temozolomide and carboplatin/paclitaxel are often used as well depending on patient preference and physician experience. Abbreviations: *BRAF*+, positive for actionable *BRAF* mutations; *BRAF*–, negative for actionable *BRAF* mutations; CNS, central nervous system; IL, interleukin; LDH, lactate dehydrogenase; PS, performance status

In patients with unresectable stage III disease, the use of T-VEC, an oncolytic herpes virus engineered to express GM-CSF, was felt to be appropriate by a significant minority of panelists (39%). This recommendation was based on results from a prospective, randomized trial in which 436 patients with unresectable stage IIIB-IV melanoma were randomized in a 2:1 fashion to treatment with T-VEC or recombinant GM-CSF [12]. The primary endpoint of the study was durable response rate (DRR), which was significantly better for T-VEC treated patients compared to control subjects (16.3% vs. 2.1%, odds ratio [OR] 8.9; *P* < 0.001). T-VEC was also associated with improved objective response rate (ORR 26.4% vs. 5.7%) and OS (median OS 23.3 months for T-VEC vs. 18.9 months for control, HR 0.79, *P* = 0.051). On a pre-specified subset analysis, however, a particularly strong effect was seen in patients with stage IIIB-IVM1a disease, where the DRR was 33% vs. 0% in stage III patients and 16% vs. 2% for stage IVM1a patients. A similar effect on OS was seen in the stage III-IVM1a patients with a 43% improvement in survival for patients treated with T-VEC [12]. Thus, there is Level A data supporting T-VEC in these patients, and T-VEC may be more appropriate for patients with limited visceral disease. Other options for this patient population would be enrollment onto a clinical trial or treatment as stage IV melanoma (see Fig. 4). Of particular interest are the multiple emerging trials of neo-adjuvant/pre-operative therapy for patients with melanoma of borderline resectability, who may be better served by initial cytoreduction and possibly a scenario, if significant response is seen, where the patient may not require resection.

### Immunotherapy for stage IV melanoma

#### Initial assessment

In patients with stage lV melanoma, a diagnostic workup that includes a multidisciplinary team review of clinical and tumor data should be conducted. Staging should be confirmed via pathological evaluation, whole body imaging, and serum LDH analysis. Genetic mutation analysis of the tumor should also be performed with special emphasis on identifying mutations in BRAF. In addition, careful attention should be paid to central nervous system (CNS) assessment since melanoma patients are at high risk of CNS metastasis. Thus, in addition to computed tomography (CT) imaging of the chest, abdomen and pelvis, an MRI of the brain should be obtained to fully stage potential metastatic melanoma patients. Surgical evaluation by a multi-disciplinary team that includes an experienced surgical oncologist for possible metastectomy is important, especially in patients with solitary pulmonary metastasis where complete extirpation is possible. If complete resection of all metastatic disease is likely, metastasectomy can be considered based on Level B retrospective outcome studies, but the panel agreed that this operative management is less compelling as systemic therapy improves [39–41]. Patients who achieve partial response (PR) or stable disease (SD) following immunotherapy should also be reassessed for possible resection [42, 43]. The panel recognizes several systemic treatment options for patients with unresectable stage IV melanoma, including immunotherapy with high-dose IL-2 (where available), ipilimumab, nivolumab, pembrolizumab, T-VEC (if accessible lesions are present), combination ipilimumab and nivolumab, clinical trial participation, and cytotoxic chemotherapy [7–12]. Additionally, vemurafenib, dabrafenib, trametinib, and the combinations of either dabrafenib and trametinib or vemurafenib and cobimetinib are options for patients with BRAF-mutated tumors [44–48]. An additional combination regimen of potent BRAF and MEK inhibitors (encorafenib and binimetinib) is anticipated to receive regulatory approval in the future.

The panel considered the overall approach to the patient with stage IV melanoma and, while previous recommendations suggested that BRAF mutation status and performance status be considered as critical elements in the decision-making process, all Task Force participants agreed that immunotherapy should be considered prior to targeted therapy in patients with good performance status, based on the potential for durable responses with immunotherapy. There is little data available to support optimal sequencing of targeted therapy and immunotherapy in this setting. However, two retrospective studies have suggested enhanced clinical benefit from immunotherapy administered prior to BRAF-targeted therapy in those patients who required both (those who did not achieve durable or curative responses to the first line of therapy) [40, 49]. A data series of 274 patients with BRAF-mutated melanoma who sequentially received BRAF inhibitors and immunotherapy (high-dose IL-2, ipilimumab, or PD-1 inhibitors) illustrated that ipilimumab therapy after BRAF inhibitors was associated with no tumor response and poor survival [50]. In another study of 93 patients with BRAF-mutated melanoma who received BRAF inhibitors (vemurafenib or dabrafenib) before or after ipilimumab, longer OS was found in the cohort of patients receiving ipilimumab prior to BRAF inhibitor therapy (14.5 vs. 9.9 months, *P* = 0.04) [49]. In both studies, the response rates to BRAF-targeted therapy was similar regardless of prior immunotherapy. Thus, starting with immunotherapy may provide patients with an opportunity for long-term benefit without negatively affecting the activity of BRAF inhibitor therapy. In order to determine optimal sequencing, the ECOG-ACRIN-led intergroup randomized protocol EA6134 (NCT02224781) has been initiated to compare the sequential administration of ipilimumab/nivolumab and dabrafenib/trametinib. OS at the 2-year landmark, the primary endpoint of this randomized phase 3 trial, is expected to be reported in 2019 or 2020.

In this edition of the guidelines, the panel suggested that key elements to consider for individual patients should include clinical performance status, tumor burden, and presence of visceral metastases (compared to M1a patients with cutaneous, soft tissue or nodal only metastatic disease), and the tempo of disease progression. While there is limited evidence, where available, most immunotherapy agents do appear to be effective against CNS metastases from melanoma [51–53]. Data recently reported from two studies also show evidence that combination nivolumab/ipilimumab has clinical activity in patients with asymptomatic brain metastases [53, 54]. In 75 patients with > 1 measurable brain metastasis who received combination ipilimumab/nivolumab, the intracranial response rate (IRR) was 56% (95% CI: 44–68); in addition, 19% of patients had a complete response (CR) [54]. Moreover, in 50 patients with untreated brain metastases, both nivolumab monotherapy (ICR 20% [95% CI: 7–41]) and combination ipilimumab/nivolumab (ICR 44% [95% CI: 24–65]) were found to be active [55]. Based on the discussion, recommendations for the management of stage IV melanoma were considered independently for patients with a good performance status, generally low disease burden and slow tempo of disease progression versus patients with a declining performance status, widespread visceral metastases and/or rapid disease progression (Fig. 4). Extent of CNS involvement, mass effect, cerebral edema and steroid requirements and symptoms will also factor into treatment decisions.

#### Consensus management of stage IV melanoma patients with a good clinical performance status

The treatment approach for patients with good performance status stage IV melanoma who are not surgical candidates should include an assessment of BRAF mutation status, history and physical examination, serum LDH, baseline laboratory evaluation and whole body imaging (see Table 1), and assessment of tempo of disease, tumor burden, and presence or absence of CNS disease before treatment selection. Only a minority of panelists felt that PD-L1 expression status (15%) or tumor cell mutation burden (10%) was important for treatment planning. For a typical patient with a good performance status, regardless of BRAF status, a majority of the panel members recommended enrollment onto a clinical trial (75%) as a first-line option, followed by treatment with combination ipilimumab and nivolumab, which was favored over single-agent PD-1 inhibitor therapy (pembrolizumab or nivolumab) by three of the five members who did not favor clinical trial. This ratio of support for combined ipilimumab and nivolumab versus single-agent anti-PD-1 therapy held up by the panel when a clinical trial was not an option (12 of 20 respondents). Half of the panelists felt that the selection of the combination of ipilimumab and nivolumab should mandate transfer of the patient to a physician or center with more immunotherapy experience due to the higher toxicity incidence and complexity associated with combination immunotherapy. Panel members (83%) also suggested that T-VEC be considered if accessible lesions for injection are present in patients whose disease has progressed after combination or monotherapy checkpoint inhibitors and who still maintain a good performance status.

Participation in clinical trials is dependent on having access to appropriate studies and ensuring that patients meet protocol-specific eligibility requirements. In addition, patients must be willing to participate in a clinical trial and provide written, informed consent. The high priority placed on clinical trials is a reflection of the progress being made in clinical drug development in melanoma and interest in defining more effective regimens with acceptable toxicity. If such clinical trials are not readily available or patients are not willing or do not qualify for participation, combination ipilimumab and nivolumab was considered the treatment of choice for patients with good performance status. This recommendation was based on a series of prospective clinical trials demonstrating improved response rates with the combination, although increased incidence of immune-related adverse events (irAEs) was also reported. In a phase 1 study, 53 melanoma patients were treated with concurrent nivolumab (doses ranged from 0.3–10 mg/kg) and ipilimumab (dose ranged from 1 to 10 mg/kg) IV every 3 weeks for four doses followed by nivolumab alone every 3 weeks for another four doses [56]. The ORR was 40% based on World Health Organization (WHO) criteria with a disease control rate of 65% [56]. Treatment-related adverse events (TRAEs) were seen in 93% of patients with grade 3 or greater events from all causes observed in 72%; 53% were considered treatment-related. The authors concluded that the maximum doses with an acceptable safety profile were nivolumab at 1 mg/kg and ipilimumab at 3 mg/kg, with objective responses seen in 53% of patients treated with this dosing regimen [56].

Following the phase 1 data, a double-blind study was conducted in 142 treatment-naïve, metastatic melanoma patients and enrolled in a 2:1 manner to treatment with ipilimumab (3 mg/kg) and nivolumab (1 mg/kg) or ipilimumab (3 mg/kg) and placebo every 3 weeks for four doses [57]. Patients in the combination group were able to receive additional maintenance nivolumab and at a median follow-up of 24.5 months, 2-year OS was 63.8% for those in the combination treatment arm vs. 53.6% in the ipilimumab arm [57]. Of note, patients in the ipilimumab arm were permitted to cross over to nivolumab monotherapy at time of disease progression, making this trial a study of combination ipilimumab and nivolumab vs. sequential ipilimumab followed by nivolumab. Interestingly, there was a 22% CR rate and improvement in progression-free survival (PFS) for the combination although median OS was not reached in either treatment group. Similar to other trials, the grade 3 or greater TRAE rate was 54% in the combination cohort compared to 20% in the ipilimumab alone cohort.

These data led to a randomized phase 3 trial in which 945 treatment-naïve patients with unresectable stage III or IV melanoma were randomized in a 1:1:1 ratio to treatment with ipilimumab and nivolumab, nivolumab alone or ipilimumab alone [11]. The study was designed with two primary endpoints, PFS and OS, with a significant improvement seen in PFS (11.5 months for the combination treated patients vs. 2.9 months for ipilimumab alone [HR 0.42, *P* < 0.001] and 6.9 months for nivolumab alone [HR 0.57, P < 0.001]). In this study, patients whose tumors exhibited > 5% PD-L1 expression had a median PFS of 14 months in both combination and nivolumab alone arms; however, in patients with PD-L1 negative tumors, the median PFS was 11.2 months for combination treated subjects compared to 5.3 months in patients treated with nivolumab alone. TRAEs of grade 3 or greater were reported in 55% of the combination treated patients, 16.3% in those receiving nivolumab alone and 27.3% in the ipilimumab alone cohort. At a minimum follow-up of 37 months, the median OS has not been reached for patients on the combination arm compared to 37.6 months and 19.9 months in patients receiving nivolumab or ipilimumab alone, respectively [58]. The three-year OS was 58% for combination therapy patients compared to 52% in nivolumab alone (HR 0.85, 95% CI: 0.68–1.07; non-significant *P*-value) and 34% in patients treated with ipilimumab alone (HR for ipilimumab/nivolumab vs. ipilimumab 0.55, 95% CI: 0.45–0.69; *P* < 0.0001; HR for nivolumab vs. ipilimumab 0.65, 95% CI: 0.53–0.80; P < 0.0001) [58].

The above described studies collectively provide Level A evidence supporting the role of combination ipilimumab and nivolumab for first-line treatment in patients with melanoma. However, the lack of a significant OS benefit for the combination over nivolumab alone, particularly in patients with BRAF WT or PD-L1-expressing tumors [58], suggests it is reasonable to consider anti-PD-1 agents alone at this time. In CheckMate 067, a sub-group analysis showed significant improvement in PFS and numerical improvement in OS with combination therapy only in patients with low (< 5 and < 1%) PD-L1 staining; however, the panel did not consider there to be sufficient data to support a role for PD-L1 expression in clinical decision-making at this time [56–58]. While adverse events are significantly greater with combination ipilimumab/nivolumab treatment compared to monotherapy, there is some evidence that health-related quality of life may not be significantly impacted by concurrent combination treatment [59] due to a greater time without disease related symptoms or treatment toxicity (as measure by QTWIST) [60].

The panel went on to recommend monotherapy with anti-PD-1 agents as another option for patients who are not able to participate in a clinical trial or are not eligible for combination ipilimumab/nivolumab. There are two agents available, pembrolizumab, which is administered at 200 mg IV every 3 weeks, and nivolumab administered at 240 mg IV every 2 weeks or 480 mg IV every 4 weeks (per a recent change to non-weight-based dosing). The panel considered these drugs equally effective, with indistinguishable toxicities, and advised that selection can be based on physician experience or patient preference.

Pembrolizumab and nivolumab are monoclonal antibodies that block the PD-1 T cell checkpoint, and there are considerable data supporting their use in the treatment of metastatic melanoma. In a clinical dose-finding study, patients with advanced melanoma were treated with pembrolizumab (initially called lambrolizumab) at a dose of 10 mg/kg every two or three weeks or 2 mg/kg every 3 weeks [9]. Patients were allowed, but not required, to have had prior ipilimumab therapy to be eligible for study participation. The study enrolled 135 patients and the response rate assessed by standard Response Evaluation Criteria in Solid Tumors (RECIST) 1.1 criteria was 38% without significant differences between doses or by prior ipilimumab exposure. The responses were durable with 81% of patients still in response at a median follow-up of 11 months. The most frequent adverse events were fatigue, rash, pruritus and diarrhea, and these were generally grade 2 or less [9]. Pembrolizumab was also evaluated in a separate multi-institutional phase 1 study evaluating doses of 2 mg/kg and 10 mg/kg every 3 weeks in patients with ipilimumab-refractory advanced melanoma [61]. In this study, 173 patients received 2 mg/kg (*n* = 89) or 10 mg/kg (*n* = 84) pembrolizumab and data were reported at a median follow-up of 8 months. The response rate by RECIST was 26% at both doses. Treatment was considered tolerable with the most frequent TRAEs being fatigue, pruritus, and rash; all were grade 2 or less except for five patients (3%) who reported grade 3 fatigue [61]. These studies led to the regulatory approval of pembrolizumab, at a dose of 2 mg/kg every 3 weeks, for the treatment of patients with metastatic melanoma. The approved dose and schedule was subsequently changed to 200 mg IV every 3 weeks.

In a multi-institutional phase 2 study, 540 melanoma patients with disease that had progressed following ipilimumab and BRAF/MEK inhibitor therapy, if their tumors harbored a BRAF (V600) mutation, were randomized 1:1:1 to treatment with pembrolizumab at 2 mg/kg every 3 weeks (*N* = 180), 10 mg/kg every 3weeks (*N* = 181) or investigator-choice chemotherapy (*N* = 179) [62]. Patients were stratified for performance status, LDH level and BRAF mutation status. The PFS was significantly better for patients in both pembrolizumab treatment arms compared to chemotherapy (HR 0.57, *P* < 0.0001 for 2 mg/kg and HR 0.50, *P* < 0.0001 for 10 mg/kg). The 6-month PFS was 34% in patients treated with pembrolizumab at 2 mg/kg, 38% at 10 mg/kg and 16% for chemotherapy. The toxicity profile was similar to previous pembrolizumab trials with an incidence of grade 3–4 adverse events of 11 and 14% in the pembrolizumab 2 mg/kg and 10 mg/kg cohorts, compared to 26% for patients receiving chemotherapy. These data were also similar to another global phase 1b clinical study in which 655 melanoma patients were treated with pembrolizumab 10 mg/kg every 2 weeks, 10 mg/kg every 3 weeks, or 2 mg/kg every 3 weeks until disease progression, intolerable toxicity, or investigator decision to stop treatment [63]. In this study, investigators evaluated the impact of pembrolizumab based on prior exposure to ipilimumab. To address this, 135 patients (48 with prior ipilimumab and 87 without) were enrolled without randomization and 520 patients were prospectively randomized (294 with prior ipilimumab and 226 without). Response rates were reported at a median follow-up of 21 months; response rates were 33% in patients with prior ipilimumab exposure and 45% in treatment-naïve patients. The 12-month PFS was 35% overall and 52% in treatment-naïve patients, and the median OS was 23 months overall and 31 months in treatment-naïve subjects. Overall, 14% of patients reported at least one grade 3 or greater TRAE. These results confirmed response rates seen in the phase 1 trials and also supported the 2 mg/kg dosing schedule.

These initial studies were followed by a randomized phase 3 clinical trial in which 834 patients with advanced melanoma were randomized 1:1:1 to pembrolizumab (10 mg/kg) every 2 weeks or every 3 weeks or four doses of ipilimumab (3 mg/kg) every 3 weeks [64]. The study was powered for primary endpoints of PFS and OS. In this study, the estimated 6-month PFS was 47.3% for pembrolizumab every 2 weeks, 46.4% for pembrolizumab every 3 weeks and 26.5% for ipilimumab (HR, 0.58; *P* < 0.001). The response rate was higher with pembrolizumab administered every 2 weeks (33.7%) and every 3 weeks (32.9%), vs. ipilimumab (11.9%) (P < 0.001 for both comparisons) and responses were durable in 89.4, 96.7, and 87.9% of patients, respectively, after a median follow-up of 7.9 months. Grade 3 or greater TRAEs were lower in the pembrolizumab cohorts (13.3 and 10.1%) compared to ipilimumab alone (19.9%).

The panel was queried about when single-agent anti-PD-1, as opposed to combination immunotherapy, was most appropriate. In considering BRAF mutation, LDH, PD-L1 expression status and mucosal histology, 42% of panelists stated that PD-L1 expression was the most important discriminating factor supporting single agent anti-PD-1 treatment, despite lack of level A evidence. One each said mucosal melanoma or PD-L1 negative status should prompt combination therapy, two stated that single-agent PD-1 therapy should always be favored, and 10 panelists felt that a number of other factors should be considered, including medical co-morbidities (e.g. autoimmune disease, history of organ transplantation, etc.), disease volume/tumor burden, site of disease, performance status, functional status, and patient preference.

Pembrolizumab has also been tested in a non-randomized phase 2 study in 52 patients with CNS metastases; eighteen patients with melanoma and 34 with non-small cell lung cancer (NSCLC) presented with untreated brain metastases and were treated with 10 mg/kg every 2 weeks until disease progression [65]. Eligible patients had metastatic lesions measuring 5–20 mm, had no neurologic symptoms, did not require corticosteroids, and for the NSCLC cohort were required to have positive tumor PD-L1 expression. A preliminary analysis was reported with evidence of CNS disease response in 4 of the 18 (22%) patients with melanoma and 6 of 18 (33%) patients with NSCLC. The responses appeared to be durable and TRAEs were typical of pembrolizumab toxicity in other studies and only 3 patients (17%) with melanoma had neurologic toxicities, including grade 3 cognitive dysfunction and grade 1–2 seizures. The authors concluded that pembrolizumab was safe in patients with CNS metastasis and might be associated with therapeutic responses.

Finally, a minority of panel members (46%) who were familiar with T-VEC recommended T-VEC be considered in patients with good performance status stage IV melanoma based on the results of the previously mentioned randomized phase 3 clinical trial [12]. This requires that tumors be clinically visible or palpable for injection or be accessible by ultrasound guidance. This option may be especially appropriate for patients who are not candidates for T cell checkpoint inhibitors, such as patients with significant co-morbid conditions, or older patients unable to tolerate significant systemic toxicity (Fig. 4).

Patients with tumors that do not respond to ipilimumab and nivolumab, monotherapy with anti-PD-1 agents, or T-VEC should be treated according to the guidelines for poor performance status patients (see Fig. 4) and treatment selection will depend on BRAF mutation status and which drug(s) an individual patient has already received. In general, panel members recommended targeted therapy (if BRAF mutation is present), combination immunotherapy (if not previously received and performance status is good), ipilimumab monotherapy (if the patient has not been previously exposed to the agent), high-dose IL-2, clinical trial participation, or chemotherapy.

There is considerable evidence supporting a role for high-dose IL-2 in the treatment of patients with stage IV melanoma, and the drug has been approved since 1998. A fairly consistent ORR of 16–17%, including 6–7% CRs, has been reported [7]. Further analysis of the original 270 patients treated in the regulatory trials at a median follow-up at 7 years demonstrated a median duration of response that was unchanged in patients achieving an initial CR or PR at 8.9 and 5.9 months, respectively [66]. The benefits of IL-2 and contemporary management of IL-2-related toxicity has been previously reported [4, 67]. Treatment generally requires referral to centers with experience in management of high-dose IL-2 and patients should have a good performance status when starting treatment.

#### Consensus management of patients with stage IV melanoma and poor clinical performance status

The panel considered that patients with a poor or declining performance status, those with extensive disease burden, rapid tempo of progression, presence of active CNS disease and those that have documented disease progression after T cell checkpoint inhibitors or T-VEC should be treated differently than those with overall good performance status, limited disease burden, slow tempo of progression and without active CNS metastasis. Patients with poor performance status should have BRAF mutation analysis to determine if there is a V600 or other targetable mutation, for which targeted therapy regimens are available [44–48]. Noting that clinical trial participation in patients with poor performance status is challenging due to protocol restrictions, the panel applauded efforts by the ASCO-Friends of Cancer Research working group, which is taking steps to broaden clinical trial eligibility and recommended that, whenever feasible, these patients be considered for clinical trial participation whether or not their tumor harbors a BRAF mutation (see Fig. 4). In the absence of a BRAF mutation, and if clinical trials are not an option, the panel recommended treatment with single agent anti-PD-1 therapy, such as pembrolizumab or nivolumab based on the Level A data described above. In patients whose tumor harbors a BRAF mutation and who are not eligible for clinical trial participation, treatment with BRAF/MEK targeted therapy should be considered, and readers are referred elsewhere for guidance on administration of these agents [68]. If patients progress on targeted therapy or are not eligible for such agents, monotherapy with pembrolizumab or nivolumab is recommended. There is evidence for activity with both BRAF/MEK inhibitors and anti-PD-1 agents alone or with ipilimumab in the treatment of CNS metastasis [69]. Combination ipilimumab/nivolumab could also be considered in selected patients where they have not previously received such treatment, the performance status decline is not related to significant medical co-morbidities and the patients is clinically able to tolerate therapy. While response rates are notably higher with combination ipilimumab/nivolumab, the incidence of serious adverse events is also higher, and the risk/benefit ratio must be considered on an individual basis. The majority of the panel (67%) recommended combination ipilimumab/nivolumab for treatment of CNS melanoma, while a minority of the panel (33%) would treat individual CNS lesions with stereotactic radiation prior to systemic immunotherapy, and this may require consultation/coordination with neurosurgery and/or radiation oncology specialists [65, 70]. As always, disease symptomatology and corticosteroid requirements will influence treatment decisions.

In patients who have failed the above treatments, regardless of performance status, other therapeutic options should include renewed consideration of targeted therapy in patients with BRAF mutated tumors if this has not been previously used. Other options include clinical trial participation, single agent ipilimumab, high-dose IL-2, T-VEC, and cytotoxic chemotherapy (Fig. 4).

Ipilimumab was initially approved for the treatment of metastatic melanoma based on several clinical trials that demonstrated durable responses and improvement in OS [8, 71]. Further follow-up studies have confirmed the potential for durable responses and long-term survival providing Level A data supporting a role for ipilimumab in melanoma [72, 73]. Here we summarize key data from ipilimumab trials that support the rationale for its use in the second-line setting in patients with advanced melanoma. The first important study was a multi-institutional, double-blind, randomized phase 3 trial in which 676 patients with advanced melanoma expressing human leukocyte antigen (HLA)-A2 were randomized to treatment with ipilimumab (3 mg/kg every 3 weeks for four doses), ipilimumab (same dose and schedule) given with an HLA-A2-restricted modified gp100 peptide vaccine, or vaccine alone [8]. Overall, patients treated with ipilimumab demonstrated improved OS compared to patients receiving vaccine alone (10 months vs. 6 months; *P* = 0.0026). This study led to FDA approval for ipilimumab as single agent therapy for melanoma in 2011. Another prospective, randomized clinical trial was subsequently reported in which 502 patients with treatment-naive melanoma were randomized to ipilimumab at 10 mg/kg every 3 weeks for four doses and dacarbazine (850 mg/m^2^) or dacarbazine (850 mg/m^2^) and placebo [71]. This trial reported improved OS in patients treated with ipilimumab and dacarbazine (11.2 months vs. 9.1 months; *P* < 0.001). The study also reported improved 3-year survival of 20.8% for ipilimumab-dacarbazine-treated patients compared to 12.2% for dacarbazine alone (HR 0.72; *P* < 0.001). An update of this study population demonstrated 5-year survival rate of 18.2% in patients in the ipilimumab and dacarbazine cohort compared to 8.8% in the dacarbazine alone arm (*P* = 0.002) [72]. A plateau in the survival curve was observed around 3 years and persisted out to 5 years. The authors also reported safety and found the only persistent grade 3 or greater irAEs involved the skin. In order to better estimate the survival benefit in patients treated with ipilimumab, a retrospective, pooled analysis of 1861 patients treated in 10 prospective and 2 retrospective trials was performed [73]. Across all studies included in the analysis, median OS was 11.4 months (range 10.7–12.1 months) and the investigators saw a similar plateau in the survival curve at approximately 3 years. A 3-year survival rate of 22% was seen in all patients with 26% in treatment-naïve subjects and 20% in previously treated patients. Ipilimumab has also been shown to have activity against CNS metastases in a single arm phase 2 clinical trial [74]. A randomized clinical study in 245 unresectable stage III-IV melanoma patients evaluated ipilimumab at 10 mg/kg intravenously on day 1 and GM-CSF at 250 μg subcutaneously on days 1–14 of each 21-day cycle [36]. In this study, an improvement in overall survival for the combination treatment was observed (17.5 vs. 12.7 months) and, unexpectedly, the incidence of serious grade 3 or greater adverse events was lower in the combination group compared to ipilimumab alone (44.9% vs. 58.3%). Although promising, further validation of this combination in a larger sample size and at ipilimumab doses of 3 mg/kg are needed.

Some panel members also recommended T-VEC in this setting. There is limited evidence supporting this recommendation. In the randomized phase 3 study, a subset analysis found that durable response was higher than control therapy in treatment-naïve patients (24% vs. 0%) when compared to those receiving T-VEC as second-line or later therapy (10 vs. 4%), and a similar trend toward better OS was seen when T-VEC was used in the first-line setting [12]. As mentioned, T-VEC treatment requires accessible lesions for direct injection. Thus, while IL-2 and T-VEC are good options to consider, careful patient selection is required to optimize therapeutic benefit.

### Special issues in tumor immunotherapy for melanoma

The panel recognized that there are several unique issues related to clinical management of patients with melanoma opting for immunotherapy. These include issues related to the clinical integration of biomarkers, laboratory assessment, and imaging in the management of patients before and during treatment. There are also concerns over management of irAEs that are unique to immunotherapy treatment and guidelines for when to stop therapy given the potential for delayed regression. While the panel largely acknowledged that there is only Level C data to inform decision-making with respect to these issues, consensus recommendations were made and are summarized in Table 1.

#### Consensus management of immune-related adverse events

Immunotherapy is associated with irAEs that manifest as autoimmune-like phenomenon involving lymphocytic infiltration and inflammation of various tissues and organ systems. These events may range from vitiligo not requiring intervention to more serious episodes of immune-related colitis, pneumonitis, hepatitis and hypophysitis [75]. More recently, there have been rare case reports of immune-related myocarditis associated with mortality [76–78]. These events are problematic and may occur early in the treatment course or weeks to even months after stopping therapy, and a high level of clinical suspicion must be maintained in patients treated with immunotherapy. The panel did not specifically address toxicity management in detail but endorsed current clinical recommendations to educate patients and caregivers about toxicities, monitor patients carefully for emergence of potential irAEs, rapidly rule out other causes and initiate corticosteroid management once a high-grade immune-mediated event is identified. There is currently some controversy as to whether there is an association between irAEs and improved therapeutic responses [79]. The panel, however, felt the data were strong enough to demonstrate prolonged responses even after treatment was stopped due to toxicity, and with the use of steroids; thus, the panel did not recommend continued treatment through significant toxicity for the purpose of enhancing clinical response.

In patients who experience grade 2 or greater adverse events, treatment may be withheld during acute management and resumed upon resolution, but treatment will likely need to be permanently discontinued in the face of a high grade or recurrent immune-mediate adverse event [14]. Additional management guidelines are widely anticipated in the near future and clinicians should monitor the literature for new guidance in this area. Several groups, including the SITC Toxicity Management Working Group, have recently published guidelines to address the management of adverse events from immune checkpoint inhibition [80–82]. We have previously reported on the management of acute IL-2 and interferon-related side effects, including interferon-associated depression in the first consensus statement on melanoma [4].

#### Consensus statement on predictive biomarkers for melanoma immunotherapy

The panel acknowledged the importance of identifying predictive biomarkers to help inform clinical decision-making in melanoma immunotherapy. Preliminary reports of higher response rates in patients treated with T cell checkpoint inhibitors who have high tumor-infiltrating lymphocytes and PD-L1 expression in the tumor microenvironment suggested these factors might serve as biomarkers [83]. In fact, PD-L1 expression has been used for patient selection and is associated with improved outcomes with anti-PD-1 therapy in NSCLC [84]. Nonetheless, PD-L1 expression has not been validated for melanoma patient selection or therapeutic monitoring, and this may relate to differences in the assay sensitivity or reliability, the dynamic regulation of PD-L1 expression and sampling error [85]. At this time, PD-L1 expression is not considered valuable in clinical management of patients with melanoma by the majority (58%) of the consensus panel. However, some panelists did consider PD-L1 expression as important in clinical decision-making in special situations, such as in patients with co-morbid medical conditions that might preclude combination immunotherapy (25% of panelists), patients older than 65 years of age (8%), patients less than 65 years of age (4%) or in the presence of BRAF mutation (4%). In these settings, high PD-L1 expression would support using single agent PD-1 blockade and reserve combination therapy for those without PD-L1 expression since these patients are less likely to respond to monotherapy [58].

Mutation burden in the tumor has also recently been recognized as a potential predictor of response to immunotherapy with T cell checkpoint inhibitors [86, 87]. Thus, it is interesting to note that melanoma, NSCLC and other tumors where these agents have shown clinical activity appear to be associated with higher levels of mutations within the tumor genome [86, 88, 89]. The biologic basis of this finding may be due to the emergence of neoantigens derived from the mutations resulting in abnormal proteins and peptide fragments within the tumor cells allowing recognition by T cells that might not recognize the native peptide [81]. Thus, mutation burden could be an important predictor of benefit for treatment with immunotherapy. In its first tissue-agnostic approval based on a biomarker, the FDA recently granted accelerated approval to pembrolizumab for the treatment of patients with unresectable or metastatic mismatch repair deficient (dMMR) or microsatellite instability-high (MSI-H) solid tumors that have progressed after prior treatment and have no alternative treatment options. This approval was based on data from 149 patients across 5 single-arm clinical trials in which pembrolizumab illustrated an ORR of 39.6%, including 11 CRs and 48 PRs [90]. Similar results led to approval of nivolumab in this population based on results from the CheckMate 142 clinical trial [91]. Another area of intense investigation is the association between therapeutic effectiveness of immunotherapy regimens and the presence of IFN-γ-related gene signatures within the tumor microenvironment [92]. While the Task Force agreed with the importance of emerging data in this area, there are not sufficient prospective validation studies to recommend use of these parameters for clinical decision-making for patients with melanoma at this time (see Table 1).

#### Consensus statement on laboratory assessment for melanoma patients on immunotherapy

The panel strongly recommended routine baseline and surveillance laboratory assessments be performed on patients undergoing treatment with tumor immunotherapy. While panelists acknowledged a lack of evidence-based data in this area, serum LDH is considered an important prognostic marker as it is part of the current AJCC (v7 and v8) staging for melanoma, and toxicity management is supported by careful laboratory analysis with baseline values for comparison. Clinicians should be alert for signs and symptoms of irAEs, which can present with isolated laboratory abnormalities, such as elevated hepatic enzymes, serum creatinine, amylase, lipase, glucose and others. A baseline complete blood count, serum chemistry panel to evaluate hepatic, renal and electrolyte parameters, and a thyroid function panel that includes at least free T4 and thyroid stimulating hormone (TSH) should be obtained on all patients. With increasing awareness of the risk of myocarditis, monitoring of creatine kinase and troponin I or T should also be considered. The panel also unanimously agreed that these same laboratory assays should be repeated during therapy but there was no agreement on the frequency of assessment. Some panel members suggested obtaining lab work prior to each infusion, whereas others suggested early monitoring and then limiting collection to periodic assessment or as clinically indicated. Patients who present with signs or symptoms of possible hypophysitis should have additional hormone levels monitored prior to starting corticosteroid intervention (see Table 1 for recommended panel).

#### Consensus statement on imaging for melanoma patients on immunotherapy

The type and frequency of imaging for patients with melanoma treated with immunotherapy continues to be controversial and there are no prospective, randomized clinical trials to guide clinical decision-making. Since tumor regression may be delayed with immunotherapy, appropriate imaging becomes increasingly important to ensure patients achieve optimal therapeutic benefit. Thus, all panel members recommended that whole body imaging be performed prior to and at regular intervals during immunotherapy. The majority of the panel use computed tomography (CT) scans of the chest, abdomen and pelvis and magnetic resonance imaging (MRI) of the brain. Additional imaging may also be necessary in some patients with suspected disease in locations not imaged with these scans, such as the neck or extremities. A minority of panel members recommended whole body positron emission tomography (PET) or PET–CT scans as the preferred imaging modality. The false-positive rate for PET imaging and difficulty providing definitive lesion measurements were reasons cited for preferring CT and MRI imaging by the majority of panel participants. Although the panel recognized the absence of Level A data to support post-treatment imaging, the consensus recommendation was that patients should be followed every 3–12 months with whole body CT imaging and selective brain imaging depending on tumor stage and location, the disease-free period from initial diagnosis and as clinically indicated (see Table 1). A minority opinion suggested that imaging could be individualized for each patient.

#### Consensus statement on clinical endpoints and treatment cessation

The panel considered the issue of when to stop treatment, which is complicated in patients receiving immunotherapy since “pseudo-progression” has been reported and is thought to be related to delayed response kinetics and/or tumor immune infiltration. This possibility has suggested that additional criteria may be needed to assess response optimally and avoid discontinuing treatment in patients who might experience delayed regression; these criteria have been termed immune-related response criteria (irRC) or iRECIST [93, 94]. While pseudo-progression has been reported with ipilimumab [8] and T-VEC [12], there is some evidence that this phenomenon may also occur with anti-PD-1 agents [95]. In a review of 655 patients treated with pembrolizumab, 24 (7%) had atypical responses defined as “early pseudo-progression” in 15 (5%) and “delayed pseudo-progression” in 9 (3%) by the investigators [95]. This study also found 14% of patients had progression by RECIST criteria but did not meet the definition for disease progression by the irRC and suggested that clinical benefit may be underestimated if standard RECIST criteria are used in monitoring clinical endpoints for immunotherapy studies. There are also case reports of pseudoprogression of melanoma brain metastases in patients treated with pembrolizumab [96].

The panel generally agreed that new lesions or an increase in tumor burden in patients treated with interferon or IL-2 is cause for treatment cessation. The assessment of response in patients receiving T cell checkpoint inhibitors or T-VEC is more challenging. The majority of the panel recommends that patients with disease progression by imaging and who are clinically asymptomatic without a decline in performance status can be safely continued on treatment and re-imaged in 1–2 months to evaluate response. There is limited Level B evidence to support this position. In a retrospective study using pooled data of 526 randomized patients from two phase 3 trials of nivolumab in treatment-naïve melanoma patients, those who received continued treatment beyond first disease progression (*N* = 85) were compared to those patients who immediately discontinued nivolumab at first signs of disease progression (*N* = 221). The authors reported that 24 of the 85 (28%) patients treated beyond progression went on to experience greater than 30% regression after further therapy [97]. The authors concluded that selected patients might derive further clinical benefit from continued treatment beyond progression. The panel also recommended that patients with unacceptable toxicity or clinical deterioration should be promptly removed from treatment and only if disease progression is documented should they move on to another therapeutic regimen.

In addition, it is critical that clinicians monitoring melanoma patients on immunotherapy be able to confirm clinical responses and stop therapy at an appropriate timepoint. The panel recognized that there is considerable controversy on how best to define when to stop therapy and agreed that there may be limited evidence to support continued treatment beyond disease progression. Because of this uncertainty, the panel considered confirmation of objective responses to be important for optimal clinical decision making, and suggested that patients achieving CR, PR or SD, should be re-imaged within 2–3 months to confirm response. A minority of the panel suggested that patients with incomplete responses, and where all remaining sites of disease can be completely excised, could be considered for surgical management or biopsy to confirm existence of viable tumor in these areas and/or identify other potential treatment options (e.g., through mutational burden analysis). Finally, the panel was asked about scenarios in which it would be appropriate to stop therapy in a patient with SD or better response. Of the panelists responding, 4% would be comfortable stopping therapy once a patient achieves a radiographic complete response, 8% would stop after achieving PET-CT-based complete response, and 29% would stop after completing 2 years of therapy. A further 38% would consider any of these endpoints appropriate to prompt treatment discontinuation. Five panelists had alternative suggestions as to when to stop treatment: after 1–2 years of therapy if disease remains stable, 1 year after documentation of a CR, or after a radiographic CR or 2 years of therapy. None of the panelists felt that pathologic CR was necessary to halt treatment.

The data to support these recommendations are, to be fair, premature. With that said, the above recommendations are made based on the anecdotal experience of each panel member who have seen the maintenance of prolonged clinical benefit off therapy, appreciating that the risks of continuing therapy indefinitely are legitimate, and the available data from melanoma clinical trials are premature. The existing published data come from the Keynote 001 study, which enrolled 655 patients with melanoma, 105 of whom developed a CR. With a median follow up of 30 months from first identification of CR, the chance of maintaining a CR was 91% in the 105 patients treated beyond response and 90% in the 67 patients who discontinued therapy for observation after CR, which was allowable per protocol [98]. In presented data at ASCO 2017, Robert and colleagues presented data from the Keynote 006 (described above) showing that in the 104 patients with SD, PR, or CR who completed 2 years of therapy with a median follow up off pembrolizumab of 9.7 months, 23 of 24 CRs and 60 of 64 PRs remained in response, while 8 of 10 patients with SD remained with stable disease.

Further, a recent pooled retrospective analysis of 2624 melanoma patients treated with PD-1 blockade from eight multi-center clinical trials submitted to the FDA, identified 692 of 1361 patients (51%) who had continued PD-1-directed treatment after documentation of RECIST-defined progressive disease [99]. The authors pooled data from all patients and found 19% of patients treated beyond progression had a 30% or greater decrease in tumor burden and this represented 4% of the entire 2624 patient population. The median overall survival was also greater in patients treated beyond progression compared to patients who did not receive treatment beyond progression (24.2 vs. 11.2 months). In this study, the rate of serious adverse events was slightly lower in the patients treated beyond progression compared to patients who stopped treatment at progression (43% vs. 54%), and immune-related adverse events were similar in incidence in both groups. The authors concluded that treatment beyond progression with anti-PD-1 therapy in might be appropriate in selected melanoma patients but clinical benefit remains to be proven.

## Conclusions

The approval of six new immunotherapy agents since 2011 has led to the emergence of cancer immunotherapy as the standard of care for patients with high-risk and advanced melanoma. However, limited data are available to guide optimal patient selection, treatment sequencing and clinical monitoring during therapy. Immunotherapy differs from standard chemotherapy in its mode of action, in being associated with a higher likelihood of durable response when response occurs, and in the potential for delayed response and appearance of irAEs that require clinical diligence to detect and treat. Further progress in the field is anticipated to focus on combination immunotherapy strategies between two or more immunotherapy agents and with targeted therapies, metabolic (e.g., indoleamine 2,3-dioxygenase [IDO], vascular endothelial growth factor [VEGF]) inhibitors and adoptively transferred T cells. This updated SITC consensus statement provides recommendations by an expert panel of melanoma specialists to assist in the clinical management of melanoma patients treated with immunotherapy, the use of which provides a beneficial therapeutic option for patients with melanoma.

## Additional files

Additional file 1: Cancer Immunotherapy Guidelines- Cutaneous melanoma version 2.0 Task Force Roster. (DOCX 13 kb)

Additional file 2: Comments from Open Review. (DOCX 14 kb)

Additional file 3: Cancer Immunotherapy Guidelines (Melanoma). (DOCX 145 kb)

## Abbreviations

CI: Confidence interval
CR: Complete response
CT: Computed tomography
CTLA-4: Cytotoxic T lymphocyte antigen-4
FDA: U.S. Food and Drug Administration
GM-CSF: Granulocyte macrophage colony-stimulating factor
HR: Hazard ratio
ICR: Intracranial response
IDO: Indoleamine 2,3-dioxygenase
IL-2: Interleukin-2
irAE: Immune-related adverse event
IV: Intravenous
LDH: Lactate dehydrogenase
MRI: Magnetic resonance imaging
NSCLC: Non-small cell lung cancer
OR: Odds ratio
OS: Overall survival
PD-1: Programmed cell death 1
PET: Positron emission tomography
PFS: Progression-free survival
PR: Partial response
SITC: Society for immunotherapy of cancer
TSH: Thyroid stimulating hormone
T-VEC: Talimogene laherparepvec
VEGF: Vascular endothelial growth factor

## Footnotes

1 There are no available data from adjuvant BRAF-targeted therapy in resected Stage IV melanoma.
