# Supplementary material for: An update on the Society for Immunotherapy of Cancer consensus statement on tumor immunotherapy for the treatment of cutaneous melanoma: version 2.0
Source: J Immunother Cancer. 2018 May 30;6:44. doi: 10.1186/s40425-018-0362-6 (PMC5977556; doi:10.1186/s40425-018-0362-6)
Supplement: Supplementary file 1 — Cancer Immunotherapy Guidelines- Cutaneous melanoma version 2.0 Task Force Roster. (DOCX 13 kb) [file 40425_2018_362_MOESM1_ESM.docx]

**ADDITIONAL FILE I:**

**Cancer Immunotherapy Guidelines- Cutaneous melanoma version 2.0 Task Force Roster**

**Steering Committee:**

Michael B. Atkins, MD, Georgetown-Lombardi Comprehensive Cancer Center

F. Stephen Hodi Jr., MD, Dana-Farber Cancer Institute

Howard L. Kaufman, MD, FACS, Replimune Group Inc.

John M. Kirkwood, MD, UPMC Hillman Cancer Center

**Task Force Participants:**

Sanjiv S. Agarwala, MD, St Luke's Cancer Center and Temple University

Joseph I. Clark, MD, Loyola University Medical Center

Marc S. Ernstoff, MD, Roswell Park Cancer Institute

Leslie A. Fecher, MD, University of Michigan

Thomas F. Gajewski, MD, PhD, University of Chicago

Brian R. Gastman, MD, Cleveland Clinic

David H. Lawson, MD, Winship Cancer Institute of Emory University

Jose Lutzky, MD, FACP, Mount Sinai Medical Center

Kim A. Margolin, MD, City of Hope

David F. McDermott, MD

Janice M. Mehnert, MD, Rutgers Cancer Institute of New Jersey

Anna C. Pavlick, DO, MD, New York University Cancer Institute

Jon M. Richards, MD, PhD, Lutheran General Hospital

Krista M. Rubin, MS, FNP-BC, Massachusetts General Hospital

William H. Sharfman, MD, Johns Hopkins Oncology Center

Steven Silverstein, MBA, Melanoma Research Foundation

Craig L. Slingluff, MD, University of Virginia

Vernon K. Sondak, MD, H. Lee Moffitt Cancer Center & Research Institute

Ryan J. Sullivan, MD, Massachusetts General Hospital

Ahmad Tarhini, MD, PhD, Cleveland Clinic Taussig Cancer Institute

John A. Thompson, MD, University of Washington

Walter J. Urba, MD, PhD, Earle A. Chiles Research Institute, Providence Cancer Center

Richard L. White Jr., MD, FACS, Carolinas Medical Center

Eric D. Whitman, MD, FACS, Atlantic Health System Cancer Care
