# Supplementary material for: An update on the Society for Immunotherapy of Cancer consensus statement on tumor immunotherapy for the treatment of cutaneous melanoma: version 2.0
Source: J Immunother Cancer. 2018 May 30;6:44. doi: 10.1186/s40425-018-0362-6 (PMC5977556; doi:10.1186/s40425-018-0362-6)
Supplement: Supplementary file 2 — Comments from Open Review. (DOCX 14 kb) [file 40425_2018_362_MOESM2_ESM.docx]

**ADDITIONAL FILE 2: Comments from Open Review**

Comments from the open review of this consensus statement will be published with this document.

| **Comment Date** | **Comment** |
| --- | --- |
| **01/05/2018** | The manuscript holds great importance in the highlihted field of melanoma immunotherapy. It summarises the most importad issues on toxicity, assesment of immunotherapy response, therapy sequencing and combinations aswell as patient eligibility. It serves as a standard guide about immunotherapy regimen for cutaneous melanoma in different stages. The presented guidelines give abundant state of art information for clinicians and a broad reader group, however there are some simple questions remaining that might be of interest: I/ How far disparities in drug approval and availabiliy in various countries worldwide can be different?  Are eventually "not FDA approved drugs" be used outside the USA somewhere in the World, out of conrol? ;II/  It would be interesting to give the percentages of the three levels of evidence (Level A, B, C) the literature was based on.; III/ In consensus management of stage II melanoma: which is the (yet unnamed) biomarker, ''known to be associated with either risk (prognostic) or responsiveness to the therapy (predictive) " enrollment of patients onto clinical trial can be based on?; IV/ The whole list of yet non-validated biomarkers that well identify patients at greatest risk of recurrence, would be eventually important to list in the paper, additional to the mentioned: ulceration, gene expression profile, circulating tumor DNA.; V/ Why is pegylated interferon a2b not recommended for patients with high-risk stage II disease?; VI/ Out of the consensus management of immune-related adverse events, immune-related myocarditis associated mortality is certainly a serious problem. Please give more exact data on this issue and some further detailes about toxicity mangement would be of special importance, also. |
| **01/05/2018** | Dear All, thank you very much for the opportunity to comment the cancer immunotherapy guidelines. The manuscript is truly well written and complete. The only comment refers to the fact that with the great success of immunotherapy, many immunotherapeutic drugs blocking the same molecule are now available. As such, the choice between the usage of one agent compared to another might be delicate. In this regard, a great example is offered  by the choice between the usage of Nivolumab or  Pembrolizumab. Both Nivolumab (Opvido, BMS) and Pembrolizumab (Keytruda, Merck) are anti-PD-1 antibodies approved by FDA and elected as very robust and generally well tolerated therapies for melanoma patients. Both agents produce objective responses in 40% of patients in the first-line setting, and in 25% to 30% of patients refractory to the cytotoxic T-lymphocyte-associated protein-4  (CTLA-4) inhibitor ipilimumab or BRAF-inhibitor therapy. The real choice between Nivolumab or Pembrolizumab primarily relates to treatment schedule. Nivolumab needs to be administered every 2 weeks while Pembrolizumab is administered every 3 weeks. As such, the real master in commander of the choice is not the treatment per se, but the preference of the patient, who should never forget that time and rapidity of intervention are fundamental in the battle against cancer. |
