# Supplementary material for: An update on the Society for Immunotherapy of Cancer consensus statement on tumor immunotherapy for the treatment of cutaneous melanoma: version 2.0
Source: J Immunother Cancer. 2018 May 30;6:44. doi: 10.1186/s40425-018-0362-6 (PMC5977556; doi:10.1186/s40425-018-0362-6)
Supplement: Supplementary file 3 — Cancer Immunotherapy Guidelines (Melanoma). (DOCX 145 kb) [file 40425_2018_362_MOESM3_ESM.docx]

**Cancer Immunotherapy
Guidelines
(Melanoma)**

**A bibliography of the literature by topic**

**Society for Immunotherapy of Cancer**

**November 21^st^, 2017**

**Table of Contents**

[TOPIC: Melanoma and Interferon 8](#_Toc499018404)

[TOPIC: Melanoma and Interleukin-2 21](#_Toc499018405)

[TOPIC: Melanoma and Ipilimumab 27](#_Toc499018406)

[TOPIC: Melanoma and Vermurafenib 40](#_Toc499018407)

[TOPIC: Melanoma and BRAF 46](#_Toc499018408)

[TOPIC: Melanoma and Dabrafenib 59](#_Toc499018409)

[TOPIC: Melanoma and Dacarbazine 59](#_Toc499018410)

[TOPIC: Melanoma and Temozolomide 64](#_Toc499018411)

[TOPIC: Melanoma and Pembrolizumab 65](#_Toc499018412)

[TOPIC: Melanoma and Nivolumab 67](#_Toc499018413)

[TOPIC: Melanoma and Talimogene Laherparepvec 68](#_Toc499018414)

[TOPIC: Melanoma and PD-1 68](#_Toc499018415)

[TOPIC: Melanoma and PD-L1 69](#_Toc499018416)

[TOPIC: Melanoma and Combination Immunotherapy 69](#_Toc499018417)

[TOPIC: Melanoma and Immunotherapy Adverse Events 86](#_Toc499018418)

[TOPIC: Melanoma and Immunotherapy Toxicity 94](#_Toc499018419)

[TOPIC: Author Recommended Additions 109](#_Toc499018420)

| **Search Terms** | **Date Limits** | **Limits** | **Search Details** | **Date Search Completed** | **Total Refs Identified** | **Record Numbers** | **Total Records Found** | **Total # Dups** | **Resulting # of Records in Bibliography** |
| --- | --- | --- | --- | --- | --- | --- | --- | --- | --- |
| All terms | All dates | Clinical Trial, Randomized Controlled Trial, Meta-Analysis, Practice Guideline, Humans |  | 11/06/2017 | 785 | 1-785 | 785 | 0 | 784 |
| Melanoma and Interferon | 01/01/2011-11/07/2017 | Clinical Trial, Randomized Controlled Trial, Meta-Analysis, Practice Guideline, Humans | ("melanoma"[MeSH Terms] OR "melanoma"[All Fields]) AND ("interferons"[MeSH Terms] OR "interferons"[All Fields] OR "interferon"[All Fields]) AND ((Clinical Trial[ptyp] OR Randomized Controlled Trial[ptyp] OR Meta-Analysis[ptyp] OR Practice Guideline[ptyp]) AND ("2011/01/01"[PDAT] : "2017/11/07"[PDAT]) AND "humans"[MeSH Terms]) | 11/06/2017 | 103 | 1-103 | 103 | 0 | 103 |
| Melanoma and Interleukin-2 | 01/01/2011-11/07/2017 | Clinical Trial, Randomized Controlled Trial, Meta-Analysis, Practice Guideline, Humans | ("melanoma"[MeSH Terms] OR "melanoma"[All Fields]) AND ("interleukin-2"[MeSH Terms] OR "interleukin-2"[All Fields] OR "interleukin 2"[All Fields]) AND ((Clinical Trial[ptyp] OR Randomized Controlled Trial[ptyp] OR Meta-Analysis[ptyp] OR Practice Guideline[ptyp]) AND ("2011/01/01"[PDAT] : "2017/11/07"[PDAT]) AND "humans"[MeSH Terms]) | 11/06/2017 | 52 | 104-143 | 52 | 12 | 40 |
| Melanoma and Ipilimumab | 01/01/2011-11/07/2017 | Clinical Trial, Randomized Controlled Trial, Meta-Analysis, Practice Guideline, Humans | ("melanoma"[MeSH Terms] OR "melanoma"[All Fields]) AND ("ipilimumab"[Supplementary Concept] OR "ipilimumab"[All Fields]) AND ((Clinical Trial[ptyp] OR Randomized Controlled Trial[ptyp] OR Meta-Analysis[ptyp] OR Practice Guideline[ptyp]) AND ("2011/01/01"[PDAT] : "2017/11/07"[PDAT]) AND "humans"[MeSH Terms]) | 2/27/2017 | 113 | 144-249 | 113 | 7 | 106 |
| Melanoma and Vermurafenib | 01/01/2011-11/07/2017 | Clinical Trial, Randomized Controlled Trial, Meta-Analysis, Practice Guideline, Humans | ("melanoma"[MeSH Terms] OR "melanoma"[All Fields]) AND ("PLX4032"[All Fields] OR "vemurafenib"[All Fields]) AND ((Clinical Trial[ptyp] OR Randomized Controlled Trial[ptyp] OR Meta-Analysis[ptyp] OR Practice Guideline[ptyp]) AND ("2011/01/01"[PDAT] : "2017/11/07"[PDAT]) AND "humans"[MeSH Terms]) | 11/06/2017 | 55 | 250-296 | 55 | 8 | 47 |
| Melanoma and BRAF | all dates | Clinical Trial, Randomized Controlled Trial, Meta-Analysis, Practice Guideline, Humans | ("melanoma"[MeSH Terms] OR "melanoma"[All Fields]) AND "BRAF"[All Fields]) AND ((Clinical Trial[ptyp] OR Randomized Controlled Trial[ptyp] OR Meta-Analysis[ptyp] OR Practice Guideline[ptyp]) AND "humans"[MeSH Terms]) | 11/06/2017 | 161 | 297-397 | 161 | 60 | 101 |
| Melanoma and Dabrafenib | all dates | Clinical Trial, Randomized Controlled Trial, Meta-Analysis, Practice Guideline, Humans | ("melanoma"[MeSH Terms] OR "melanoma"[All Fields]) AND "dabrafenib"[All Fields] AND ((Clinical Trial[ptyp] OR Randomized Controlled Trial[ptyp] OR Meta-Analysis[ptyp] OR Practice Guideline[ptyp]) AND "humans"[MeSH Terms]) | 11/06/2017 | 45 | 398 | 45 | 44 | 1 |
| Melanoma and Dacarbazine | 01/01/2011-11/07/2017 | Clinical Trial, Randomized Controlled Trial, Meta-Analysis, Practice Guideline, Humans | ("melanoma"[MeSH Terms] OR "melanoma"[All Fields]) AND ("dacarbazine"[MeSH Terms] OR "dacarbazine"[All Fields]) AND ((Clinical Trial[ptyp] OR Randomized Controlled Trial[ptyp] OR Meta-Analysis[ptyp] OR Practice Guideline[ptyp]) AND ("2011/01/01"[PDAT] : "2017/11/07"[PDAT]) AND "humans"[MeSH Terms]) | 11/06/2017 | 89 | 399-437 | 89 | 50 | 39 |
| Melanoma and Temozolomide | 01/01/2011-11/07/2017 | Clinical Trial, Randomized Controlled Trial, Meta-Analysis, Practice Guideline, Humans | ("melanoma"[MeSH Terms] OR "melanoma"[All Fields]) AND ("temozolomide"[Supplementary Concept] OR "temozolomide"[All Fields]) AND ((Clinical Trial[ptyp] OR Randomized Controlled Trial[ptyp] OR Meta-Analysis[ptyp] OR Practice Guideline[ptyp]) AND ("2011/01/01"[PDAT] : "2017/11/07"[PDAT]) AND "humans"[MeSH Terms]) | 11/06/2017 | 35 | NA | 35 | 35 | 0 |
| Melanoma and Pembrolizumab | all dates | Clinical Trial, Randomized Controlled Trial, Meta-Analysis, Practice Guideline, Humans | ("melanoma"[MeSH Terms] OR "melanoma"[All Fields]) AND ("pembrolizumab"[Supplementary Concept] OR "pembrolizumab"[All Fields]) AND ((Clinical Trial[ptyp] OR Randomized Controlled Trial[ptyp] OR Meta-Analysis[ptyp] OR Practice Guideline[ptyp]) AND "humans"[MeSH Terms]) | 11/06/2017 | 31 | 438-454 | 31 | 14 | 17 |
| Melanoma and Nivolumab | all dates | Clinical Trial, Randomized Controlled Trial, Meta-Analysis, Practice Guideline, Humans | ("melanoma"[MeSH Terms] OR "melanoma"[All Fields]) AND ("nivolumab"[Supplementary Concept] OR "nivolumab"[All Fields]) AND ((Clinical Trial[ptyp] OR Randomized Controlled Trial[ptyp] OR Meta-Analysis[ptyp] OR Practice Guideline[ptyp]) AND "humans"[MeSH Terms]) | 11/06/2017 | 40 | 455-464 | 40 | 30 | 10 |
| Melanoma and Talimogene Laherparepvec | all dates | Clinical Trial, Randomized Controlled Trial, Meta-Analysis, Practice Guideline, Humans | (melanoma) AND talimogene laherparepvec AND ( ( Clinical Trial[ptyp] OR Randomized Controlled Trial[ptyp] OR Meta-Analysis[ptyp] OR Practice Guideline[ptyp] ) AND Humans[Mesh]) | 11/06/2017 | 5 | 465 | 5 | 4 | 1 |
| Melanoma and PD-1 | all dates | Clinical Trial, Randomized Controlled Trial, Meta-Analysis, Practice Guideline, Humans | ("melanoma"[MeSH Terms] OR "melanoma"[All Fields]) AND PD-1[All Fields] AND ((Clinical Trial[ptyp] OR Randomized Controlled Trial[ptyp] OR Meta-Analysis[ptyp] OR Practice Guideline[ptyp]) AND "humans"[MeSH Terms]) | 11/06/2017 | 50 | 466-470 | 50 | 45 | 5 |
| Melanoma and PD-L1 | all dates | Clinical Trial, Randomized Controlled Trial, Meta-Analysis, Practice Guideline, Humans | ("melanoma"[MeSH Terms] OR "melanoma"[All Fields]) AND ("antigens, cd274"[MeSH Terms] OR ("antigens"[All Fields] AND "cd274"[All Fields]) OR "cd274 antigens"[All Fields] OR ("pd"[All Fields] AND "l1"[All Fields]) OR "pd l1"[All Fields]) AND ((Clinical Trial[ptyp] OR Randomized Controlled Trial[ptyp] OR Meta-Analysis[ptyp] OR Practice Guideline[ptyp]) AND "humans"[MeSH Terms]) | 11/06/2017 | 25 | 471-472 | 25 | 23 | 2 |
| Melanoma and Combination Immunotherapy | all dates | Clinical Trial, Randomized Controlled Trial, Meta-Analysis, Practice Guideline, Humans | ("melanoma"[MeSH Terms] OR "melanoma"[All Fields]) AND (combination[All Fields] AND ("immunotherapy"[MeSH Terms] OR "immunotherapy"[All Fields])) AND ((Clinical Trial[ptyp] OR Randomized Controlled Trial[ptyp] OR Meta-Analysis[ptyp] OR Practice Guideline[ptyp]) AND "humans"[MeSH Terms]) | 11/06/2017 | 164 | 473-606 | 164 | 30 | 134 |
| Melanoma and Immunotherapy Adverse Events | all dates | Clinical Trial, Randomized Controlled Trial, Meta-Analysis, Practice Guideline, Humans | ("melanoma"[MeSH Terms] OR "melanoma"[All Fields]) AND (("adverse events"[All Fields] OR "side effects"[All Fields]) AND ("immunotherapy"[MeSH Terms] OR "immunotherapy"[All Fields])) AND ((Clinical Trial[ptyp] OR Randomized Controlled Trial[ptyp] OR Meta-Analysis[ptyp] OR Practice Guideline[ptyp]) AND "humans"[MeSH Terms]) | 11/06/2017 | 121 | 607-671 | 121 | 56 | 65 |
| Melanoma and Immunotherapy Toxicity | all dates | Clinical Trial, Randomized Controlled Trial, Meta-Analysis, Practice Guideline, Humans | ("melanoma"[MeSH Terms] OR "melanoma"[All Fields]) AND ("toxicity"[Subheading] OR "toxicity"[All Fields]) AND ("immunotherapy"[MeSH Terms] OR "immunotherapy"[All Fields]) AND ((Clinical Trial[ptyp] OR Randomized Controlled Trial[ptyp] OR Meta-Analysis[ptyp] OR Practice Guideline[ptyp]) AND "humans"[MeSH Terms]) | 11/06/2017 | 212 | 672-785 | 212 | 98 | 114 |
| Author Recommended Additions | All dates | N/A | N/A | N/A | 87 | 786-838 | 87 | 34 | 53 |

# TOPIC: Melanoma and Interferon

1. [1]. Agarwala SS, Lee SJ, Yip W, Rao UN, Tarhini AA, Cohen GI, Reintgen DS, Evans TL, Brell JM, Albertini MR *et al*: **Phase III Randomized Study of 4 Weeks of High-Dose Interferon-alpha-2b in Stage T2bNO, T3a-bNO, T4a-bNO, and T1-4N1a-2a (microscopic) Melanoma: A Trial of the Eastern Cooperative Oncology Group-American College of Radiology Imaging Network Cancer Research Group (E1697)**. *Journal of clinical oncology : official journal of the American Society of Clinical Oncology* 2017, **35**(8):885-892.
2. [2]. Alrwas A, Papadopoulos NE, Cain S, Patel SP, Kim KB, Deburr TL, Bassett R, Jr., Hwu WJ, Bedikian AY, Davies MA *et al*: **Phase I trial of biochemotherapy with cisplatin, temozolomide, and dose escalation of nab-paclitaxel combined with interleukin-2 and interferon-alpha in patients with metastatic melanoma**. *Melanoma research* 2014, **24**(4):342-348.
3. [3]. Aoyagi S, Hata H, Homma E, Shimizu H: **Sequential local injection of low-dose interferon-beta for maintenance therapy in stage II and III melanoma: a single-institution matched case-control study**. *Oncology* 2012, **82**(3):139-146.
4. [4]. Ascierto PA, Chiarion-Sileni V, Muggiano A, Mandala M, Pimpinelli N, Del Vecchio M, Rinaldi G, Simeone E, Queirolo P: **Interferon alpha for the adjuvant treatment of melanoma: review of international literature and practical recommendations from an expert panel on the use of interferon**. *Journal of chemotherapy (Florence, Italy)* 2014, **26**(4):193-201.
5. [5]. Ayers M, Lunceford J, Nebozhyn M, Murphy E, Loboda A, Kaufman DR, Albright A, Cheng JD, Kang SP, Shankaran V *et al*: **IFN-gamma-related mRNA profile predicts clinical response to PD-1 blockade**. *The Journal of clinical investigation* 2017, **127**(8):2930-2940.
6. [6]. Bjoern J, Brimnes MK, Andersen MH, Thor Straten P, Svane IM: **Changes in peripheral blood level of regulatory T cells in patients with malignant melanoma during treatment with dendritic cell vaccination and low-dose IL-2**. *Scandinavian journal of immunology* 2011, **73**(3):222-233.
7. [7]. Borden EC, Jacobs B, Hollovary E, Rybicki L, Elson P, Olencki T, Triozzi P: **Gene regulatory and clinical effects of interferon beta in patients with metastatic melanoma: a phase II trial**. *Journal of interferon & cytokine research : the official journal of the International Society for Interferon and Cytokine Research* 2011, **31**(5):433-440.
8. [8]. Bouwhuis MG, Collette S, Suciu S, de Groot ER, Kruit WH, Ten Hagen TL, Aarden LA, Eggermont AM, Swaak AJ: **Changes of ferritin and CRP levels in melanoma patients treated with adjuvant interferon-alpha (EORTC 18952) and prognostic value on treatment outcome**. *Melanoma research* 2011, **21**(4):344-351.
9. [9]. Bouwhuis MG, Suciu S, Kruit W, Sales F, Stoitchkov K, Patel P, Cocquyt V, Thomas J, Lienard D, Eggermont AM *et al*: **Prognostic value of serial blood S100B determinations in stage IIB-III melanoma patients: a corollary study to EORTC trial 18952**. *European journal of cancer (Oxford, England : 1990)* 2011, **47**(3):361-368.
10. [10]. Brandberg Y, Aamdal S, Bastholt L, Hernberg M, Stierner U, von der Maase H, Hansson J: **Health-related quality of life in patients with high-risk melanoma randomised in the Nordic phase 3 trial with adjuvant intermediate-dose interferon alfa-2b**. *European journal of cancer (Oxford, England : 1990)* 2012, **48**(13):2012-2019.
11. [11]. Brandberg Y, Johansson H, Aamdal S, Bastholt L, Hernberg M, Stierner U, von der Maase H, Hansson J: **Role functioning before start of adjuvant treatment was an independent prognostic factor for survival and time to failure. A report from the Nordic adjuvant interferon trial for patients with high-risk melanoma**. *Acta oncologica (Stockholm, Sweden)* 2013, **52**(6):1086-1093.
12. [12]. Busse A, Rapion J, Fusi A, Suciu S, Nonnenmacher A, Santinami M, Kruit WH, Testori A, Punt CJ, Dalgleish AG *et al*: **Analysis of surrogate gene expression markers in peripheral blood of melanoma patients to predict treatment outcome of adjuvant pegylated interferon alpha 2b (EORTC 18991 side study)**. *Cancer immunology, immunotherapy : CII* 2013, **62**(7):1223-1233.
13. [13]. Camisaschi C, Filipazzi P, Tazzari M, Casati C, Beretta V, Pilla L, Patuzzo R, Maurichi A, Cova A, Maio M *et al*: **Effects of cyclophosphamide and IL-2 on regulatory CD4+ T cell frequency and function in melanoma patients vaccinated with HLA-class I peptides: impact on the antigen-specific T cell response**. *Cancer immunology, immunotherapy : CII* 2013, **62**(5):897-908.
14. [14]. Care MA, Westhead DR, Tooze RM: **Gene expression meta-analysis reveals immune response convergence on the IFNgamma-STAT1-IRF1 axis and adaptive immune resistance mechanisms in lymphoma**. *Genome medicine* 2015, **7**:96.
15. [15]. Carreno BM, Becker-Hapak M, Huang A, Chan M, Alyasiry A, Lie WR, Aft RL, Cornelius LA, Trinkaus KM, Linette GP: **IL-12p70-producing patient DC vaccine elicits Tc1-polarized immunity**. *The Journal of clinical investigation* 2013, **123**(8):3383-3394.
16. [16]. Chen Z, Cao M, Plana MN, Liang J, Cai H, Kuwana M, Sun L: **Utility of anti-melanoma differentiation-associated gene 5 antibody measurement in identifying patients with dermatomyositis and a high risk for developing rapidly progressive interstitial lung disease: a review of the literature and a meta-analysis**. *Arthritis care & research* 2013, **65**(8):1316-1324.
17. [17]. Coit DG, Andtbacka R, Anker CJ, Bichakjian CK, Carson WE, 3rd, Daud A, Dimaio D, Fleming MD, Guild V, Halpern AC *et al*: **Melanoma, version 2.2013: featured updates to the NCCN guidelines**. *Journal of the National Comprehensive Cancer Network : JNCCN* 2013, **11**(4):395-407.
18. [18]. Coker SA, Dandamudi UB, Beelen AP, Crosby NA, Fisher JL, Obrocea M, Ernstoff MS, Lewis LD: **A phase I, dose-escalation study of cyclical weekly oral temozolomide and weekly PEG-interferon alpha-2b in patients with refractory or advanced solid tumours**. *Journal of chemotherapy (Florence, Italy)* 2013, **25**(6):362-368.
19. [19]. Cornforth AN, Fowler AW, Carbonell DJ, Dillman RO: **Resistance to the proapoptotic effects of interferon-gamma on melanoma cells used in patient-specific dendritic cell immunotherapy is associated with improved overall survival**. *Cancer immunology, immunotherapy : CII* 2011, **60**(1):123-131.
20. [20]. Cornforth AN, Lee G, Dillman RO: **Autologous peripheral blood mononuclear cell recognition of autologous proliferating tumor cells in the context of a patient-specific vaccine trial**. *Journal of biomedicine & biotechnology* 2011, **2011**:635850.
21. [21]. Daponte A, Signoriello S, Maiorino L, Massidda B, Simeone E, Grimaldi AM, Caraco C, Palmieri G, Cossu A, Botti G *et al*: **Phase III randomized study of fotemustine and dacarbazine versus dacarbazine with or without interferon-alpha in advanced malignant melanoma**. *Journal of translational medicine* 2013, **11**:38.
22. [22]. de Rosa F, Ridolfi L, Ridolfi R, Gentili G, Valmorri L, Nanni O, Petrini M, Fiammenghi L, Granato AM, Ancarani V *et al*: **Vaccination with autologous dendritic cells loaded with autologous tumor lysate or homogenate combined with immunomodulating radiotherapy and/or preleukapheresis IFN-alpha in patients with metastatic melanoma: a randomised "proof-of-principle" phase II study**. *Journal of translational medicine* 2014, **12**:209.
23. [23]. Degen A, Weichenthal M, Ugurel S, Trefzer U, Kilian K, Garbe C, Egberts F, Poppe LM, Hauschild A, Gutzmer R: **Cutaneous side effects of combined therapy with sorafenib and pegylated interferon alpha-2b in metastatic melanoma (phase II DeCOG trial)**. *Journal der Deutschen Dermatologischen Gesellschaft = Journal of the German Society of Dermatology : JDDG* 2013, **11**(9):846-853.
24. [24]. Di Trolio R, Simeone E, Di Lorenzo G, Grimaldi AM, Romano A, Ayala F, Caraco C, Mozzillo N, Ascierto PA: **Update on PEG-interferon alpha-2b as adjuvant therapy in melanoma**. *Anticancer research* 2012, **32**(9):3901-3909.
25. [25]. Dillman RO, McClay EF, Barth NM, Amatruda TT, Schwartzberg LS, Mahdavi K, de Leon C, Ellis RE, DePriest C: **Dendritic Versus Tumor Cell Presentation of Autologous Tumor Antigens for Active Specific Immunotherapy in Metastatic Melanoma: Impact on Long-Term Survival by Extent of Disease at the Time of Treatment**. *Cancer biotherapy & radiopharmaceuticals* 2015, **30**(5):187-194.
26. [26]. Donia M, Hansen M, Sendrup SL, Iversen TZ, Ellebaek E, Andersen MH, Straten P, Svane IM: **Methods to improve adoptive T-cell therapy for melanoma: IFN-gamma enhances anticancer responses of cell products for infusion**. *The Journal of investigative dermatology* 2013, **133**(2):545-552.
27. [27]. Dudley ME, Gross CA, Somerville RP, Hong Y, Schaub NP, Rosati SF, White DE, Nathan D, Restifo NP, Steinberg SM *et al*: **Randomized selection design trial evaluating CD8+-enriched versus unselected tumor-infiltrating lymphocytes for adoptive cell therapy for patients with melanoma**. *Journal of clinical oncology : official journal of the American Society of Clinical Oncology* 2013, **31**(17):2152-2159.
28. [28]. Egberts F, Gutzmer R, Ugurel S, Becker JC, Trefzer U, Degen A, Schenck F, Frey L, Wilhelm T, Hassel JC *et al*: **Sorafenib and pegylated interferon-alpha2b in advanced metastatic melanoma: a multicenter phase II DeCOG trial**. *Annals of oncology : official journal of the European Society for Medical Oncology* 2011, **22**(7):1667-1674.
29. [29]. Egger ME, Kimbrough CW, Stromberg AJ, Quillo AR, Martin RC, 2nd, Scoggins CR, McMasters KM: **Melanoma Patient-Reported Quality of Life Outcomes Following Sentinel Lymph Node Biopsy, Completion Lymphadenectomy, and Adjuvant Interferon: Results from the Sunbelt Melanoma Trial**. *Annals of surgical oncology* 2016, **23**(3):1019-1025.
30. [30]. Eggermont AM, Suciu S, Rutkowski P, Kruit WH, Punt CJ, Dummer R, Sales F, Keilholz U, de Schaetzen G, Testori A: **Long term follow up of the EORTC 18952 trial of adjuvant therapy in resected stage IIB-III cutaneous melanoma patients comparing intermediate doses of interferon-alpha-2b (IFN) with observation: Ulceration of primary is key determinant for IFN-sensitivity**. *European journal of cancer (Oxford, England : 1990)* 2016, **55**:111-121.
31. [31]. Eggermont AM, Suciu S, Testori A, Kruit WH, Marsden J, Punt CJ, Santinami M, Sales F, Schadendorf D, Patel P *et al*: **Ulceration and stage are predictive of interferon efficacy in melanoma: results of the phase III adjuvant trials EORTC 18952 and EORTC 18991**. *European journal of cancer (Oxford, England : 1990)* 2012, **48**(2):218-225.
32. [32]. Eggermont AM, Suciu S, Testori A, Santinami M, Kruit WH, Marsden J, Punt CJ, Sales F, Dummer R, Robert C *et al*: **Long-term results of the randomized phase III trial EORTC 18991 of adjuvant therapy with pegylated interferon alfa-2b versus observation in resected stage III melanoma**. *Journal of clinical oncology : official journal of the American Society of Clinical Oncology* 2012, **30**(31):3810-3818.
33. [33]. Fishman MN, Thompson JA, Pennock GK, Gonzalez R, Diez LM, Daud AI, Weber JS, Huang BY, Tang S, Rhode PR *et al*: **Phase I trial of ALT-801, an interleukin-2/T-cell receptor fusion protein targeting p53 (aa264-272)/HLA-A*0201 complex, in patients with advanced malignancies**. *Clinical cancer research : an official journal of the American Association for Cancer Research* 2011, **17**(24):7765-7775.
34. [34]. Flaherty LE, Othus M, Atkins MB, Tuthill RJ, Thompson JA, Vetto JT, Haluska FG, Pappo AS, Sosman JA, Redman BG *et al*: **Southwest Oncology Group S0008: a phase III trial of high-dose interferon Alfa-2b versus cisplatin, vinblastine, and dacarbazine, plus interleukin-2 and interferon in patients with high-risk melanoma--an intergroup study of cancer and leukemia Group B, Children's Oncology Group, Eastern Cooperative Oncology Group, and Southwest Oncology Group**. *Journal of clinical oncology : official journal of the American Society of Clinical Oncology* 2014, **32**(33):3771-3778.
35. [35]. Fourcade J, Sun Z, Pagliano O, Chauvin JM, Sander C, Janjic B, Tarhini AA, Tawbi HA, Kirkwood JM, Moschos S *et al*: **PD-1 and Tim-3 regulate the expansion of tumor antigen-specific CD8(+) T cells induced by melanoma vaccines**. *Cancer research* 2014, **74**(4):1045-1055.
36. [36]. Gajewski TF, Salama AK, Niedzwiecki D, Johnson J, Linette G, Bucher C, Blaskovich MA, Sebti SM, Haluska F: **Phase II study of the farnesyltransferase inhibitor R115777 in advanced melanoma (CALGB 500104)**. *Journal of translational medicine* 2012, **10**:246.
37. [37]. Garbe C, Peris K, Hauschild A, Saiag P, Middleton M, Spatz A, Grob JJ, Malvehy J, Newton-Bishop J, Stratigos A *et al*: **Diagnosis and treatment of melanoma. European consensus-based interdisciplinary guideline--Update 2012**. *European journal of cancer (Oxford, England : 1990)* 2012, **48**(15):2375-2390.
38. [38]. Go RS, Lee SJ, Shin D, Callister SM, Jobe DA, Conry RM, Tarhini AA, Kirkwood JM: **ECOG phase II trial of graded-dose peginterferon alpha-2b in patients with metastatic melanoma overexpressing basic fibroblast growth factor (E2602)**. *Clinical cancer research : an official journal of the American Association for Cancer Research* 2013, **19**(23):6597-6604.
39. [39]. Grignol VP, Olencki T, Relekar K, Taylor C, Kibler A, Kefauver C, Wei L, Walker MJ, Chen HX, Kendra K *et al*: **A phase 2 trial of bevacizumab and high-dose interferon alpha 2B in metastatic melanoma**. *Journal of immunotherapy (Hagerstown, Md : 1997)* 2011, **34**(6):509-515.
40. [40]. Grignol VP, Smith AD, Shlapak D, Zhang X, Del Campo SM, Carson WE: **Increased visceral to subcutaneous fat ratio is associated with decreased overall survival in patients with metastatic melanoma receiving anti-angiogenic therapy**. *Surgical oncology* 2015, **24**(4):353-358.
41. [41]. Grob JJ, Jouary T, Dreno B, Asselineau J, Gutzmer R, Hauschild A, Leccia MT, Landthaler M, Garbe C, Sassolas B *et al*: **Adjuvant therapy with pegylated interferon alfa-2b (36 months) versus low-dose interferon alfa-2b (18 months) in melanoma patients without macrometastatic nodes: an open-label, randomised, phase 3 European Association for Dermato-Oncology (EADO) study**. *European journal of cancer (Oxford, England : 1990)* 2013, **49**(1):166-174.
42. [42]. Guenterberg KD, Grignol VP, Relekar KV, Varker KA, Chen HX, Kendra KL, Olencki TE, Carson WE, 3rd: **A pilot study of bevacizumab and interferon-alpha2b in ocular melanoma**. *American journal of clinical oncology* 2011, **34**(1):87-91.
43. [43]. Guillot B, Dalac S, Denis MG, Dupuy A, Emile JF, De La Fouchardiere A, Hindie E, Jouary T, Lassau N, Mirabel X *et al*: **[Update to the recommendations for management of melanoma stages I to III]**. *Annales de dermatologie et de venereologie* 2016, **143**(10):629-652.
44. [44]. Hansson J, Aamdal S, Bastholt L, Brandberg Y, Hernberg M, Nilsson B, Stierner U, von der Maase H: **Two different durations of adjuvant therapy with intermediate-dose interferon alfa-2b in patients with high-risk melanoma (Nordic IFN trial): a randomised phase 3 trial**. *The Lancet Oncology* 2011, **12**(2):144-152.
45. [45]. Hau HM, Tautenhahn HM, Schoenberg MB, Atanasov G, Wiltberger G, Morgul MH, Uhlmann D, Seitz AT, Simon JC, Schmelzle M *et al*: **Liver resection in multimodal concepts improves survival of metastatic melanoma: a single-centre case-matched control study**. *Anticancer research* 2014, **34**(11):6633-6639.
46. [46]. Herndon TM, Demko SG, Jiang X, He K, Gootenberg JE, Cohen MH, Keegan P, Pazdur R: **U.S. Food and Drug Administration Approval: peginterferon-alfa-2b for the adjuvant treatment of patients with melanoma**. *The oncologist* 2012, **17**(10):1323-1328.
47. [47]. Hofmann MA, Kiecker F, Kuchler I, Kors C, Trefzer U: **Serum TNF-alpha, B2M and sIL-2R levels are biological correlates of outcome in adjuvant IFN-alpha2b treatment of patients with melanoma**. *Journal of cancer research and clinical oncology* 2011, **137**(3):455-462.
48. [48]. Ives NJ, Suciu S, Eggermont AMM, Kirkwood J, Lorigan P, Markovic SN, Garbe C, Wheatley K: **Adjuvant interferon-alpha for the treatment of high-risk melanoma: An individual patient data meta-analysis**. *European journal of cancer (Oxford, England : 1990)* 2017, **82**:171-183.
49. [49]. Karasawa K, Wakatsuki M, Kato S, Kiyohara H, Kamada T: **Clinical trial of carbon ion radiotherapy for gynecological melanoma**. *Journal of radiation research* 2014, **55**(2):343-350.
50. [50]. Karpus ON, Heutinck KM, Wijnker PJ, Tak PP, Hamann J: **Triggering of the dsRNA sensors TLR3, MDA5, and RIG-I induces CD55 expression in synovial fibroblasts**. *PloS one* 2012, **7**(5):e35606.
51. [51]. Kaufman HL, Kim DW, Kim-Schulze S, DeRaffele G, Jagoda MC, Broucek JR, Zloza A: **Results of a randomized phase I gene therapy clinical trial of nononcolytic fowlpox viruses encoding T cell costimulatory molecules**. *Human gene therapy* 2014, **25**(5):452-460.
52. [52]. Kaufman HL, Kirkwood JM, Hodi FS, Agarwala S, Amatruda T, Bines SD, Clark JI, Curti B, Ernstoff MS, Gajewski T *et al*: **The Society for Immunotherapy of Cancer consensus statement on tumour immunotherapy for the treatment of cutaneous melanoma**. *Nature reviews Clinical oncology* 2013, **10**(10):588-598.
53. [53]. Khammari A, Nguyen JM, Saint-Jean M, Knol AC, Pandolfino MC, Quereux G, Brocard A, Peuvrel L, Saiagh S, Bataille V *et al*: **Adoptive T cell therapy combined with intralesional administrations of TG1042 (adenovirus expressing interferon-gamma) in metastatic melanoma patients**. *Cancer immunology, immunotherapy : CII* 2015, **64**(7):805-815.
54. [54]. Kimbrough CW, Egger ME, McMasters KM, Stromberg AJ, Martin RC, Philips P, Scoggins CR: **Molecular Staging of Sentinel Lymph Nodes Identifies Melanoma Patients at Increased Risk of Nodal Recurrence**. *Journal of the American College of Surgeons* 2016, **222**(4):357-363.
55. [55]. Kollgaard T, Ugurel-Becker S, Idorn M, Andersen MH, Becker JC, Straten PT: **Pre-Vaccination Frequencies of Th17 Cells Correlate with Vaccine-Induced T-Cell Responses to Survivin-Derived Peptide Epitopes**. *PloS one* 2015, **10**(7):e0131934.
56. [56]. Kunzmann V, Smetak M, Kimmel B, Weigang-Koehler K, Goebeler M, Birkmann J, Becker J, Schmidt-Wolf IG, Einsele H, Wilhelm M: **Tumor-promoting versus tumor-antagonizing roles of gammadelta T cells in cancer immunotherapy: results from a prospective phase I/II trial**. *Journal of immunotherapy (Hagerstown, Md : 1997)* 2012, **35**(2):205-213.
57. [57]. Leiter U, Stadler R, Mauch C, Hohenberger W, Brockmeyer N, Berking C, Sunderkotter C, Kaatz M, Schulte KW, Lehmann P *et al*: **Complete lymph node dissection versus no dissection in patients with sentinel lymph node biopsy positive melanoma (DeCOG-SLT): a multicentre, randomised, phase 3 trial**. *The Lancet Oncology* 2016, **17**(6):757-767.
58. [58]. Lian B, Si L, Cui C, Chi Z, Sheng X, Mao L, Li S, Kong Y, Tang B, Guo J: **Phase II randomized trial comparing high-dose IFN-alpha2b with temozolomide plus cisplatin as systemic adjuvant therapy for resected mucosal melanoma**. *Clinical cancer research : an official journal of the American Association for Cancer Research* 2013, **19**(16):4488-4498.
59. [59]. Lipson EJ, Sharfman WH, Chen S, McMiller TL, Pritchard TS, Salas JT, Sartorius-Mergenthaler S, Freed I, Ravi S, Wang H *et al*: **Safety and immunologic correlates of Melanoma GVAX, a GM-CSF secreting allogeneic melanoma cell vaccine administered in the adjuvant setting**. *Journal of translational medicine* 2015, **13**:214.
60. [60]. Malczewski A, Marshall A, Payne MJ, Mao L, Bafaloukos D, Si L, Pectasides D, Fountzilas G, Guo J, Gogas H *et al*: **Intravenous high-dose interferon with or without maintenance treatment in melanoma at high risk of recurrence: meta-analysis of three trials**. *Cancer medicine* 2016, **5**(1):17-23.
61. [61]. Mao L, Si L, Chi Z, Cui C, Sheng X, Li S, Tang B, Guo J: **A randomised phase II trial of 1 month versus 1 year of adjuvant high-dose interferon alpha-2b in high-risk acral melanoma patients**. *European journal of cancer (Oxford, England : 1990)* 2011, **47**(10):1498-1503.
62. [62]. Markowitz J, Luedke EA, Grignol VP, Hade EM, Paul BK, Mundy-Bosse BL, Brooks TR, Dao TV, Kondalasula SV, Lesinski GB *et al*: **A phase I trial of bortezomib and interferon-alpha-2b in metastatic melanoma**. *Journal of immunotherapy (Hagerstown, Md : 1997)* 2014, **37**(1):55-62.
63. [63]. Mauldin IS, Wages NA, Stowman AM, Wang E, Smolkin ME, Olson WC, Deacon DH, Smith KT, Galeassi NV, Chianese-Bullock KA *et al*: **Intratumoral interferon-gamma increases chemokine production but fails to increase T cell infiltration of human melanoma metastases**. *Cancer immunology, immunotherapy : CII* 2016, **65**(10):1189-1199.
64. [64]. McMasters KM, Egger ME, Edwards MJ, Ross MI, Reintgen DS, Noyes RD, Martin RC, 2nd, Goydos JS, Beitsch PD, Urist MM *et al*: **Final Results of the Sunbelt Melanoma Trial: A Multi-Institutional Prospective Randomized Phase III Study Evaluating the Role of Adjuvant High-Dose Interferon Alfa-2b and Completion Lymph Node Dissection for Patients Staged by Sentinel Lymph Node Biopsy**. *Journal of clinical oncology : official journal of the American Society of Clinical Oncology* 2016, **34**(10):1079-1086.
65. [65]. McNutt MD, Liu S, Manatunga A, Royster EB, Raison CL, Woolwine BJ, Demetrashvili MF, Miller AH, Musselman DL: **Neurobehavioral effects of interferon-alpha in patients with hepatitis-C: symptom dimensions and responsiveness to paroxetine**. *Neuropsychopharmacology : official publication of the American College of Neuropsychopharmacology* 2012, **37**(6):1444-1454.
66. [66]. Miranda MB, Lauseker M, Kraus MP, Proetel U, Hanfstein B, Fabarius A, Baerlocher GM, Heim D, Hossfeld DK, Kolb HJ *et al*: **Secondary malignancies in chronic myeloid leukemia patients after imatinib-based treatment: long-term observation in CML Study IV**. *Leukemia* 2016, **30**(6):1255-1262.
67. [67]. Mocellin S, Lens MB, Pasquali S, Pilati P, Chiarion Sileni V: **Interferon alpha for the adjuvant treatment of cutaneous melanoma**. *The Cochrane database of systematic reviews* 2013(6):Cd008955.
68. [68]. Mohr P, Hauschild A, Trefzer U, Enk A, Tilgen W, Loquai C, Gogas H, Haalck T, Koller J, Dummer R *et al*: **Intermittent High-Dose Intravenous Interferon Alfa-2b for Adjuvant Treatment of Stage III Melanoma: Final Analysis of a Randomized Phase III Dermatologic Cooperative Oncology Group Trial**. *Journal of clinical oncology : official journal of the American Society of Clinical Oncology* 2015, **33**(34):4077-4084.
69. [69]. Naing A, Papadopoulos KP, Autio KA, Ott PA, Patel MR, Wong DJ, Falchook GS, Pant S, Whiteside M, Rasco DR *et al*: **Safety, Antitumor Activity, and Immune Activation of Pegylated Recombinant Human Interleukin-10 (AM0010) in Patients With Advanced Solid Tumors**. *Journal of clinical oncology : official journal of the American Society of Clinical Oncology* 2016, **34**(29):3562-3569.
70. [70]. Navid F, Herzog CE, Sandoval J, Daryani VM, Stewart CF, Gattuso J, Mandrell B, Phipps S, Chemaitilly W, Sykes A *et al*: **Feasibility of Pegylated Interferon in Children and Young Adults With Resected High-Risk Melanoma**. *Pediatric blood & cancer* 2016, **63**(7):1207-1213.
71. [71]. Noor R, Bedikian AY, Mahoney S, Bassett R, Jr., Kim K, Papadopoulos N, Hwu WJ, Hwu P, Homsi J: **Comparison of two dosing schedules of palonosetron for the prevention of nausea and vomiting due to interleukin-2-based biochemotherapy**. *Supportive care in cancer : official journal of the Multinational Association of Supportive Care in Cancer* 2012, **20**(10):2583-2588.
72. [72]. Payne MJ, Argyropoulou K, Lorigan P, McAleer JJ, Farrugia D, Davidson N, Kelly C, Chao D, Marshall E, Han C *et al*: **Phase II pilot study of intravenous high-dose interferon with or without maintenance treatment in melanoma at high risk of recurrence**. *Journal of clinical oncology : official journal of the American Society of Clinical Oncology* 2014, **32**(3):185-190.
73. [73]. Plimack ER, Desai JR, Issa JP, Jelinek J, Sharma P, Vence LM, Bassett RL, Ilagan JL, Papadopoulos NE, Hwu WJ: **A phase I study of decitabine with pegylated interferon alpha-2b in advanced melanoma: impact on DNA methylation and lymphocyte populations**. *Investigational new drugs* 2014, **32**(5):969-975.
74. [74]. Ramirez AG, Wages NA, Hu Y, Smolkin ME, Slingluff CL, Jr.: **Defining the effects of age and gender on immune response and outcomes to melanoma vaccination: a retrospective analysis of a single-institution clinical trials' experience**. *Cancer immunology, immunotherapy : CII* 2015, **64**(12):1531-1539.
75. [75]. Ravindranath MH, Selvan SR, Terasaki PI: **Augmentation of anti-HLA-E antibodies with concomitant HLA-Ia reactivity in IFNgamma-treated autologous melanoma cell vaccine recipients**. *Journal of immunotoxicology* 2012, **9**(3):282-291.
76. [76]. Ribas A, Dummer R, Puzanov I, VanderWalde A, Andtbacka RHI, Michielin O, Olszanski AJ, Malvehy J, Cebon J, Fernandez E *et al*: **Oncolytic Virotherapy Promotes Intratumoral T Cell Infiltration and Improves Anti-PD-1 Immunotherapy**. *Cell* 2017, **170**(6):1109-1119.e1110.
77. [77]. Rittig SM, Haentschel M, Weimer KJ, Heine A, Muller MR, Brugger W, Horger MS, Maksimovic O, Stenzl A, Hoerr I *et al*: **Intradermal vaccinations with RNA coding for TAA generate CD8+ and CD4+ immune responses and induce clinical benefit in vaccinated patients**. *Molecular therapy : the journal of the American Society of Gene Therapy* 2011, **19**(5):990-999.
78. [78]. Romano E, Rossi M, Ratzinger G, de Cos MA, Chung DJ, Panageas KS, Wolchok JD, Houghton AN, Chapman PB, Heller G *et al*: **Peptide-loaded Langerhans cells, despite increased IL15 secretion and T-cell activation in vitro, elicit antitumor T-cell responses comparable to peptide-loaded monocyte-derived dendritic cells in vivo**. *Clinical cancer research : an official journal of the American Association for Cancer Research* 2011, **17**(7):1984-1997.
79. [79]. Rozera C, Cappellini GA, D'Agostino G, Santodonato L, Castiello L, Urbani F, Macchia I, Arico E, Casorelli I, Sestili P *et al*: **Intratumoral injection of IFN-alpha dendritic cells after dacarbazine activates anti-tumor immunity: results from a phase I trial in advanced melanoma**. *Journal of translational medicine* 2015, **13**:139.
80. [80]. Rudman SM, Jameson MB, McKeage MJ, Savage P, Jodrell DI, Harries M, Acton G, Erlandsson F, Spicer JF: **A phase 1 study of AS1409, a novel antibody-cytokine fusion protein, in patients with malignant melanoma or renal cell carcinoma**. *Clinical cancer research : an official journal of the American Association for Cancer Research* 2011, **17**(7):1998-2005.
81. [81]. Salerno EP, Shea SM, Olson WC, Petroni GR, Smolkin ME, McSkimming C, Chianese-Bullock KA, Slingluff CL, Jr.: **Activation, dysfunction and retention of T cells in vaccine sites after injection of incomplete Freund's adjuvant, with or without peptide**. *Cancer immunology, immunotherapy : CII* 2013, **62**(7):1149-1159.
82. [82]. Sarkar S, Schaefer M: **Antidepressant pretreatment for the prevention of interferon alfa-associated depression: a systematic review and meta-analysis**. *Psychosomatics* 2014, **55**(3):221-234.
83. [83]. Schaefer C, Butterfield LH, Lee S, Kim GG, Visus C, Albers A, Kirkwood JM, Whiteside TL: **Function but not phenotype of melanoma peptide-specific CD8(+) T cells correlate with survival in a multiepitope peptide vaccine trial (ECOG 1696)**. *International journal of cancer* 2012, **131**(4):874-884.
84. [84]. Slingluff CL, Jr., Petroni GR, Chianese-Bullock KA, Smolkin ME, Ross MI, Haas NB, von Mehren M, Grosh WW: **Randomized multicenter trial of the effects of melanoma-associated helper peptides and cyclophosphamide on the immunogenicity of a multipeptide melanoma vaccine**. *Journal of clinical oncology : official journal of the American Society of Clinical Oncology* 2011, **29**(21):2924-2932.
85. [85]. Soldevila B, Alonso N, Martinez-Arconada MJ, Granada ML, Boada A, Vallejos V, Fraile M, Fernandez-Sanmartin MA, Pujol-Borrell R, Puig-Domingo M *et al*: **Regulatory T cells and other lymphocyte subpopulations in patients with melanoma developing interferon-induced thyroiditis during high-dose interferon-alpha2b treatment**. *Clinical endocrinology* 2013, **78**(4):621-628.
86. [86]. Steele JC, Rao A, Marsden JR, Armstrong CJ, Berhane S, Billingham LJ, Graham N, Roberts C, Ryan G, Uppal H *et al*: **Phase I/II trial of a dendritic cell vaccine transfected with DNA encoding melan A and gp100 for patients with metastatic melanoma**. *Gene therapy* 2011, **18**(6):584-593.
87. [87]. Tarhini AA, Cherian J, Moschos SJ, Tawbi HA, Shuai Y, Gooding WE, Sander C, Kirkwood JM: **Safety and efficacy of combination immunotherapy with interferon alfa-2b and tremelimumab in patients with stage IV melanoma**. *Journal of clinical oncology : official journal of the American Society of Clinical Oncology* 2012, **30**(3):322-328.
88. [88]. Tarhini AA, Leng S, Moschos SJ, Yin Y, Sander C, Lin Y, Gooding WE, Kirkwood JM: **Safety and immunogenicity of vaccination with MART-1 (26-35, 27L), gp100 (209-217, 210M), and tyrosinase (368-376, 370D) in adjuvant with PF-3512676 and GM-CSF in metastatic melanoma**. *Journal of immunotherapy (Hagerstown, Md : 1997)* 2012, **35**(4):359-366.
89. [89]. Tarhini AA, Lin Y, Zahoor H, Shuai Y, Butterfield LH, Ringquist S, Gogas H, Sander C, Lee S, Agarwala SS *et al*: **Pro-Inflammatory Cytokines Predict Relapse-Free Survival after One Month of Interferon-alpha but Not Observation in Intermediate Risk Melanoma Patients**. *PloS one* 2015, **10**(7):e0132745.
90. [90]. Tarhini AA, Shin D, Lee SJ, Stuckert J, Sander CA, Kirkwood JM: **Serologic evidence of autoimmunity in E2696 and E1694 patients with high-risk melanoma treated with adjuvant interferon alfa**. *Melanoma research* 2014, **24**(2):150-157.
91. [91]. Triozzi PL, Achberger S, Aldrich W, Singh AD, Grane R, Borden EC: **The association of blood angioregulatory microRNA levels with circulating endothelial cells and angiogenic proteins in patients receiving dacarbazine and interferon**. *Journal of translational medicine* 2012, **10**:241.
92. [92]. Ulloa-Montoya F, Louahed J, Dizier B, Gruselle O, Spiessens B, Lehmann FF, Suciu S, Kruit WH, Eggermont AM, Vansteenkiste J *et al*: **Predictive gene signature in MAGE-A3 antigen-specific cancer immunotherapy**. *Journal of clinical oncology : official journal of the American Society of Clinical Oncology* 2013, **31**(19):2388-2395.
93. [93]. Van Nuffel AM, Tuyaerts S, Benteyn D, Wilgenhof S, Corthals J, Heirman C, Neyns B, Thielemans K, Bonehill A: **Epitope and HLA-type independent monitoring of antigen-specific T-cells after treatment with dendritic cells presenting full-length tumor antigens**. *Journal of immunological methods* 2012, **377**(1-2):23-36.
94. [94]. Verdegaal EM, Visser M, Ramwadhdoebe TH, van der Minne CE, van Steijn JA, Kapiteijn E, Haanen JB, van der Burg SH, Nortier JW, Osanto S: **Successful treatment of metastatic melanoma by adoptive transfer of blood-derived polyclonal tumor-specific CD4+ and CD8+ T cells in combination with low-dose interferon-alpha**. *Cancer immunology, immunotherapy : CII* 2011, **60**(7):953-963.
95. [95]. Vihinen P, Tervahartiala T, Sorsa T, Hansson J, Bastholt L, Aamdal S, Stierner U, Pyrhonen S, Syrjanen K, Lundin J *et al*: **Benefit of adjuvant interferon alfa-2b (IFN-alpha) therapy in melanoma patients with high serum MMP-8 levels**. *Cancer immunology, immunotherapy : CII* 2015, **64**(2):173-180.
96. [96]. Wang R, Jing G, Lv J, Song H, Li C, Wang X, Xia W, Wu Y, Ren G, Guo W: **Interferon-alpha-2b as an adjuvant therapy prolongs survival of patients with previously resected oral muscosal melanoma**. *Genetics and molecular research : GMR* 2015, **14**(4):11944-11954.
97. [97]. Wilgenhof S, Corthals J, Van Nuffel AM, Benteyn D, Heirman C, Bonehill A, Thielemans K, Neyns B: **Long-term clinical outcome of melanoma patients treated with messenger RNA-electroporated dendritic cell therapy following complete resection of metastases**. *Cancer immunology, immunotherapy : CII* 2015, **64**(3):381-388.
98. [98]. Wilgenhof S, Van Nuffel AM, Corthals J, Heirman C, Tuyaerts S, Benteyn D, De Coninck A, Van Riet I, Verfaillie G, Vandeloo J *et al*: **Therapeutic vaccination with an autologous mRNA electroporated dendritic cell vaccine in patients with advanced melanoma**. *Journal of immunotherapy (Hagerstown, Md : 1997)* 2011, **34**(5):448-456.
99. [99]. Xin Y, Huang Q, Zhang P, Yang M, Hou XY, Tang JQ, Zhang LZ, Jiang G: **Meta-Analysis of the Safety and Efficacy of Interferon Combined With Dacarbazine Versus Dacarbazine Alone in Cutaneous Malignant Melanoma**. *Medicine* 2016, **95**(16):e3406.
100. [100]. Yamazaki N, Kiyohara Y, Uhara H, Iizuka H, Uehara J, Otsuka F, Fujisawa Y, Takenouchi T, Isei T, Iwatsuki K *et al*: **Cytokine biomarkers to predict antitumor responses to nivolumab suggested in a phase 2 study for advanced melanoma**. *Cancer science* 2017, **108**(5):1022-1031.
101. [101]. Yamazaki N, Uhara H, Wada H, Matsuda K, Yamamoto K, Shimamoto T, Kiyohara Y: **Phase I study of pegylated interferon-alpha-2b as an adjuvant therapy in Japanese patients with malignant melanoma**. *The Journal of dermatology* 2016, **43**(10):1146-1153.
102. [102]. Yi T, Elson P, Mitsuhashi M, Jacobs B, Hollovary E, Budd TG, Spiro T, Triozzi P, Borden EC: **Phosphatase inhibitor, sodium stibogluconate, in combination with interferon (IFN) alpha 2b: phase I trials to identify pharmacodynamic and clinical effects**. *Oncotarget* 2011, **2**(12):1155-1164.
103. [103]. Ziefle S, Egberts F, Heinze S, Volkenandt M, Schmid-Wendtner M, Tilgen W, Linse R, Boettjer J, Vogt T, Spieth K *et al*: **Health-related quality of life before and during adjuvant interferon-alpha treatment for patients with malignant melanoma (DeCOG-trial)**. *Journal of immunotherapy (Hagerstown, Md : 1997)* 2011, **34**(4):403-408.

# TOPIC: Melanoma and Interleukin-2

1. [104]. Albertini MR, Hank JA, Gadbaw B, Kostlevy J, Haldeman J, Schalch H, Gan J, Kim K, Eickhoff J, Gillies SD *et al*: **Phase II trial of hu14.18-IL2 for patients with metastatic melanoma**. *Cancer immunology, immunotherapy : CII* 2012, **61**(12):2261-2271.
2. [105]. Baur AS, Lutz MB, Schierer S, Beltrame L, Theiner G, Zinser E, Ostalecki C, Heidkamp G, Haendle I, Erdmann M *et al*: **Denileukin diftitox (ONTAK) induces a tolerogenic phenotype in dendritic cells and stimulates survival of resting Treg**. *Blood* 2013, **122**(13):2185-2194.
3. [106]. Besser MJ, Shapira-Frommer R, Itzhaki O, Treves AJ, Zippel DB, Levy D, Kubi A, Shoshani N, Zikich D, Ohayon Y *et al*: **Adoptive transfer of tumor-infiltrating lymphocytes in patients with metastatic melanoma: intent-to-treat analysis and efficacy after failure to prior immunotherapies**. *Clinical cancer research : an official journal of the American Association for Cancer Research* 2013, **19**(17):4792-4800.
4. [107]. Block MS, Suman VJ, Nevala WK, Kottschade LA, Creagan ET, Kaur JS, Quevedo JF, McWilliams RR, Markovic SN: **Pilot study of granulocyte-macrophage colony-stimulating factor and interleukin-2 as immune adjuvants for a melanoma peptide vaccine**. *Melanoma research* 2011, **21**(5):438-445.
5. [108]. Butler MO, Friedlander P, Milstein MI, Mooney MM, Metzler G, Murray AP, Tanaka M, Berezovskaya A, Imataki O, Drury L *et al*: **Establishment of antitumor memory in humans using in vitro-educated CD8+ T cells**. *Science translational medicine* 2011, **3**(80):80ra34.
6. [109]. Chandran SS, Paria BC, Srivastava AK, Rothermel LD, Stephens DJ, Dudley ME, Somerville R, Wunderlich JR, Sherry RM, Yang JC *et al*: **Persistence of CTL clones targeting melanocyte differentiation antigens was insufficient to mediate significant melanoma regression in humans**. *Clinical cancer research : an official journal of the American Association for Cancer Research* 2015, **21**(3):534-543.
7. [110]. Chandran SS, Somerville RPT, Yang JC, Sherry RM, Klebanoff CA, Goff SL, Wunderlich JR, Danforth DN, Zlott D, Paria BC *et al*: **Treatment of metastatic uveal melanoma with adoptive transfer of tumour-infiltrating lymphocytes: a single-centre, two-stage, single-arm, phase 2 study**. *The Lancet Oncology* 2017, **18**(6):792-802.
8. [111]. Chi M, Dudek AZ: **Vaccine therapy for metastatic melanoma: systematic review and meta-analysis of clinical trials**. *Melanoma research* 2011, **21**(3):165-174.
9. [112]. Chiarion-Sileni V, Guida M, Ridolfi L, Romanini A, Del Bianco P, Pigozzo J, Brugnara S, Colucci G, Ridolfi R, De Salvo GL: **Central nervous system failure in melanoma patients: results of a randomised, multicentre phase 3 study of temozolomide- and dacarbazine- based regimens**. *British journal of cancer* 2011, **104**(12):1816-1821.
10. [113]. Eigentler TK, Weide B, de Braud F, Spitaleri G, Romanini A, Pflugfelder A, Gonzalez-Iglesias R, Tasciotti A, Giovannoni L, Schwager K *et al*: **A dose-escalation and signal-generating study of the immunocytokine L19-IL2 in combination with dacarbazine for the therapy of patients with metastatic melanoma**. *Clinical cancer research : an official journal of the American Association for Cancer Research* 2011, **17**(24):7732-7742.
11. [114]. Ellebaek E, Engell-Noerregaard L, Iversen TZ, Froesig TM, Munir S, Hadrup SR, Andersen MH, Svane IM: **Metastatic melanoma patients treated with dendritic cell vaccination, Interleukin-2 and metronomic cyclophosphamide: results from a phase II trial**. *Cancer immunology, immunotherapy : CII* 2012, **61**(10):1791-1804.
12. [115]. Finocchiaro LM, Glikin GC: **Cytokine-enhanced vaccine and suicide gene therapy as surgery adjuvant treatments for spontaneous canine melanoma: 9 years of follow-up**. *Cancer gene therapy* 2012, **19**(12):852-861.
13. [116]. Foureau DM, Amin A, White RL, Anderson W, Jones CP, Sarantou T, McKillop IH, Salo JC: **Sequential immune monitoring in patients with melanoma and renal cell carcinoma treated with high-dose interleukin-2: immune patterns and correlation with outcome**. *Cancer immunology, immunotherapy : CII* 2014, **63**(12):1329-1340.
14. [117]. Gangadhar TC, Clark JI, Karrison T, Gajewski TF: **Phase II study of the Src kinase inhibitor saracatinib (AZD0530) in metastatic melanoma**. *Investigational new drugs* 2013, **31**(3):769-773.
15. [118]. Gores KM, Delsing AS, Kraus SJ, Powers L, Vaena DA, Milhem MM, Monick M, Doerschug KC: **Plasma angiopoietin 2 concentrations are related to impaired lung function and organ failure in a clinical cohort receiving high-dose interleukin 2 therapy**. *Shock (Augusta, Ga)* 2014, **42**(2):115-120.
16. [119]. Greene JM, Schneble EJ, Jackson DO, Hale DF, Vreeland TJ, Flores M, Martin J, Herbert GS, Hardin MO, Yu X *et al*: **A phase I/IIa clinical trial in stage IV melanoma of an autologous tumor-dendritic cell fusion (dendritoma) vaccine with low dose interleukin-2**. *Cancer immunology, immunotherapy : CII* 2016, **65**(4):383-392.
17. [120]. Jha G, Miller JS, Curtsinger JM, Zhang Y, Mescher MF, Dudek AZ: **Randomized phase II study of IL-2 with or without an allogeneic large multivalent immunogen vaccine for the treatment of stage IV melanoma**. *American journal of clinical oncology* 2014, **37**(3):261-265.
18. [121]. Jiang BS, Beasley GM, Speicher PJ, Mosca PJ, Morse MA, Hanks B, Salama A, Tyler DS: **Immunotherapy following regional chemotherapy treatment of advanced extremity melanoma**. *Annals of surgical oncology* 2014, **21**(8):2525-2531.
19. [122]. Lee SM, Moon J, Redman BG, Chidiac T, Flaherty LE, Zha Y, Othus M, Ribas A, Sondak VK, Gajewski TF *et al*: **Phase 2 study of RO4929097, a gamma-secretase inhibitor, in metastatic melanoma: SWOG 0933**. *Cancer* 2015, **121**(3):432-440.
20. [123]. Lu YC, Parker LL, Lu T, Zheng Z, Toomey MA, White DE, Yao X, Li YF, Robbins PF, Feldman SA *et al*: **Treatment of Patients With Metastatic Cancer Using a Major Histocompatibility Complex Class II-Restricted T-Cell Receptor Targeting the Cancer Germline Antigen MAGE-A3**. *Journal of clinical oncology : official journal of the American Society of Clinical Oncology* 2017, **35**(29):3322-3329.
21. [124]. Minkis K, Garden BC, Wu S, Pulitzer MP, Lacouture ME: **The risk of rash associated with ipilimumab in patients with cancer: a systematic review of the literature and meta-analysis**. *Journal of the American Academy of Dermatology* 2013, **69**(3):e121-128.
22. [125]. Mitchell DA, Cui X, Schmittling RJ, Sanchez-Perez L, Snyder DJ, Congdon KL, Archer GE, Desjardins A, Friedman AH, Friedman HS *et al*: **Monoclonal antibody blockade of IL-2 receptor alpha during lymphopenia selectively depletes regulatory T cells in mice and humans**. *Blood* 2011, **118**(11):3003-3012.
23. [126]. Monk P, Lam E, Mortazavi A, Kendra K, Lesinski GB, Mace TA, Geyer S, Carson WE, 3rd, Tahiri S, Bhinder A *et al*: **A phase I study of high-dose interleukin-2 with sorafenib in patients with metastatic renal cell carcinoma and melanoma**. *Journal of immunotherapy (Hagerstown, Md : 1997)* 2014, **37**(3):180-186.
24. [127]. Musselman D, Royster EB, Wang M, Long Q, Trimble LM, Mann TK, Graciaa DS, McNutt MD, Auyeung NS, Oliver L *et al*: **The impact of escitalopram on IL-2-induced neuroendocrine, immune, and behavioral changes in patients with malignant melanoma: preliminary findings**. *Neuropsychopharmacology : official publication of the American College of Neuropsychopharmacology* 2013, **38**(10):1921-1928.
25. [128]. Peng RQ, Ding Y, Zhang X, Liao Y, Zheng LM, Zhang XS: **A pilot study of paclitaxel combined with gemcitabine followed by interleukin-2 and granulocyte macrophage colony-stimulating factor for patients with metastatic melanoma**. *Cancer biology & therapy* 2012, **13**(14):1443-1448.
26. [129]. Petrella TM, Tozer R, Belanger K, Savage KJ, Wong R, Smylie M, Kamel-Reid S, Tron V, Chen BE, Hunder NN *et al*: **Interleukin-21 has activity in patients with metastatic melanoma: a phase II study**. *Journal of clinical oncology : official journal of the American Society of Clinical Oncology* 2012, **30**(27):3396-3401.
27. [130]. Pilon-Thomas S, Kuhn L, Ellwanger S, Janssen W, Royster E, Marzban S, Kudchadkar R, Zager J, Gibney G, Sondak VK *et al*: **Efficacy of adoptive cell transfer of tumor-infiltrating lymphocytes after lymphopenia induction for metastatic melanoma**. *Journal of immunotherapy (Hagerstown, Md : 1997)* 2012, **35**(8):615-620.
28. [131]. Poschke I, Lovgren T, Adamson L, Nystrom M, Andersson E, Hansson J, Tell R, Masucci GV, Kiessling R: **A phase I clinical trial combining dendritic cell vaccination with adoptive T cell transfer in patients with stage IV melanoma**. *Cancer immunology, immunotherapy : CII* 2014, **63**(10):1061-1071.
29. [132]. Radvanyi LG, Bernatchez C, Zhang M, Fox PS, Miller P, Chacon J, Wu R, Lizee G, Mahoney S, Alvarado G *et al*: **Specific lymphocyte subsets predict response to adoptive cell therapy using expanded autologous tumor-infiltrating lymphocytes in metastatic melanoma patients**. *Clinical cancer research : an official journal of the American Association for Cancer Research* 2012, **18**(24):6758-6770.
30. [133]. Ridolfi L, de Rosa F, Ridolfi R, Gentili G, Valmorri L, Scarpi E, Parisi E, Romeo A, Guidoboni M: **Radiotherapy as an immunological booster in patients with metastatic melanoma or renal cell carcinoma treated with high-dose Interleukin-2: evaluation of biomarkers of immunologic and therapeutic response**. *Journal of translational medicine* 2014, **12**:262.
31. [134]. Robbins PF, Morgan RA, Feldman SA, Yang JC, Sherry RM, Dudley ME, Wunderlich JR, Nahvi AV, Helman LJ, Mackall CL *et al*: **Tumor regression in patients with metastatic synovial cell sarcoma and melanoma using genetically engineered lymphocytes reactive with NY-ESO-1**. *Journal of clinical oncology : official journal of the American Society of Clinical Oncology* 2011, **29**(7):917-924.
32. [135]. Rosenberg SA, Yang JC, Sherry RM, Kammula US, Hughes MS, Phan GQ, Citrin DE, Restifo NP, Robbins PF, Wunderlich JR *et al*: **Durable complete responses in heavily pretreated patients with metastatic melanoma using T-cell transfer immunotherapy**. *Clinical cancer research : an official journal of the American Association for Cancer Research* 2011, **17**(13):4550-4557.
33. [136]. Roviello G, Zanotti L, Correale P, Gobbi A, Wigfield S, Guglielmi A, Pacifico C, Generali D: **Is still there a role for IL-2 for solid tumors other than melanoma or renal cancer?** *Immunotherapy* 2017, **9**(1):25-32.
34. [137]. Schwartzentruber DJ, Lawson DH, Richards JM, Conry RM, Miller DM, Treisman J, Gailani F, Riley L, Conlon K, Pockaj B *et al*: **gp100 peptide vaccine and interleukin-2 in patients with advanced melanoma**. *The New England journal of medicine* 2011, **364**(22):2119-2127.
35. [138]. Seung SK, Curti BD, Crittenden M, Walker E, Coffey T, Siebert JC, Miller W, Payne R, Glenn L, Bageac A *et al*: **Phase 1 study of stereotactic body radiotherapy and interleukin-2--tumor and immunological responses**. *Science translational medicine* 2012, **4**(137):137ra174.
36. [139]. Telang S, Rasku MA, Clem AL, Carter K, Klarer AC, Badger WR, Milam RA, Rai SN, Pan J, Gragg H *et al*: **Phase II trial of the regulatory T cell-depleting agent, denileukin diftitox, in patients with unresectable stage IV melanoma**. *BMC cancer* 2011, **11**:515.
37. [140]. Tseng J, Citrin DE, Waldman M, White DE, Rosenberg SA, Yang JC: **Thrombotic microangiopathy in metastatic melanoma patients treated with adoptive cell therapy and total body irradiation**. *Cancer* 2014, **120**(9):1426-1432.
38. [141]. Viaud S, Flament C, Zoubir M, Pautier P, LeCesne A, Ribrag V, Soria JC, Marty V, Vielh P, Robert C *et al*: **Cyclophosphamide induces differentiation of Th17 cells in cancer patients**. *Cancer research* 2011, **71**(3):661-665.
39. [142]. Weide B, Eigentler TK, Pflugfelder A, Leiter U, Meier F, Bauer J, Schmidt D, Radny P, Pfohler C, Garbe C: **Survival after intratumoral interleukin-2 treatment of 72 melanoma patients and response upon the first chemotherapy during follow-up**. *Cancer immunology, immunotherapy : CII* 2011, **60**(4):487-493.
40. [143]. Weide B, Martens A, Wistuba-Hamprecht K, Zelba H, Maier L, Lipp HP, Klumpp BD, Soffel D, Eigentler TK, Garbe C: **Combined treatment with ipilimumab and intratumoral interleukin-2 in pretreated patients with stage IV melanoma-safety and efficacy in a phase II study**. *Cancer immunology, immunotherapy : CII* 2017, **66**(4):441-449.

# TOPIC: Melanoma and Ipilimumab

1. [144]. Albarel F, Gaudy C, Castinetti F, Carre T, Morange I, Conte-Devolx B, Grob JJ, Brue T: **Long-term follow-up of ipilimumab-induced hypophysitis, a common adverse event of the anti-CTLA-4 antibody in melanoma**. *European journal of endocrinology* 2015, **172**(2):195-204.
2. [145]. Araujo PB, Coelho MC, Arruda M, Gadelha MR, Neto LV: **Ipilimumab-induced hypophysitis: review of the literature**. *Journal of endocrinological investigation* 2015, **38**(11):1159-1166.
3. [146]. Ascierto PA, Del Vecchio M, Robert C, Mackiewicz A, Chiarion-Sileni V, Arance A, Lebbe C, Bastholt L, Hamid O, Rutkowski P *et al*: **Ipilimumab 10 mg/kg versus ipilimumab 3 mg/kg in patients with unresectable or metastatic melanoma: a randomised, double-blind, multicentre, phase 3 trial**. *The Lancet Oncology* 2017, **18**(5):611-622.
4. [147]. Ascierto PA, Vanella V, Grimaldi AM, Lucia F, Palla M, Simeone E, Mozzillo N: **Complete response to nivolumab monotherapy in a treatment-naive, BRAF wild-type patient with advanced mucosal melanoma and elevated lactate dehydrogenase: a case report from a phase III trial**. *Cancer immunology, immunotherapy : CII* 2016, **65**(11):1395-1400.
5. [148]. Belum VR, Benhuri B, Postow MA, Hellmann MD, Lesokhin AM, Segal NH, Motzer RJ, Wu S, Busam KJ, Wolchok JD *et al*: **Characterisation and management of dermatologic adverse events to agents targeting the PD-1 receptor**. *European journal of cancer (Oxford, England : 1990)* 2016, **60**:12-25.
6. [149]. Bjoern J, Iversen TZ, Nitschke NJ, Andersen MH, Svane IM: **Safety, immune and clinical responses in metastatic melanoma patients vaccinated with a long peptide derived from indoleamine 2,3-dioxygenase in combination with ipilimumab**. *Cytotherapy* 2016, **18**(8):1043-1055.
7. [150]. Brower V: **Combination immunotherapy breakthrough for melanoma**. *The Lancet Oncology* 2015, **16**(7):e318.
8. [151]. Chen H, Fu T, Suh WK, Tsavachidou D, Wen S, Gao J, Ng Tang D, He Q, Sun J, Sharma P: **CD4 T cells require ICOS-mediated PI3K signaling to increase T-Bet expression in the setting of anti-CTLA-4 therapy**. *Cancer immunology research* 2014, **2**(2):167-176.
9. [152]. Chiarion Sileni V, Pigozzo J, Ascierto PA, Grimaldi AM, Maio M, Di Guardo L, Marchetti P, de Rosa F, Nuzzo C, Testori A *et al*: **Efficacy and safety of ipilimumab in elderly patients with pretreated advanced melanoma treated at Italian centres through the expanded access programme**. *Journal of experimental & clinical cancer research : CR* 2014, **33**:30.
10. [153]. Chiarion-Sileni V, Pigozzo J, Ascierto PA, Simeone E, Maio M, Calabro L, Marchetti P, De Galitiis F, Testori A, Ferrucci PF *et al*: **Ipilimumab retreatment in patients with pretreated advanced melanoma: the expanded access programme in Italy**. *British journal of cancer* 2014, **110**(7):1721-1726.
11. [154]. Cho SY, Lipson EJ, Im HJ, Rowe SP, Gonzalez EM, Blackford A, Chirindel A, Pardoll DM, Topalian SL, Wahl RL: **Prediction of Response to Immune Checkpoint Inhibitor Therapy Using Early-Time-Point 18F-FDG PET/CT Imaging in Patients with Advanced Melanoma**. *Journal of nuclear medicine : official publication, Society of Nuclear Medicine* 2017, **58**(9):1421-1428.
12. [155]. Coens C, Suciu S, Chiarion-Sileni V, Grob JJ, Dummer R, Wolchok JD, Schmidt H, Hamid O, Robert C, Ascierto PA *et al*: **Health-related quality of life with adjuvant ipilimumab versus placebo after complete resection of high-risk stage III melanoma (EORTC 18071): secondary outcomes of a multinational, randomised, double-blind, phase 3 trial**. *The Lancet Oncology* 2017, **18**(3):393-403.
13. [156]. Coit DG, Thompson JA, Algazi A, Andtbacka R, Bichakjian CK, Carson WE, 3rd, Daniels GA, DiMaio D, Ernstoff M, Fields RC *et al*: **Melanoma, Version 2.2016, NCCN Clinical Practice Guidelines in Oncology**. *Journal of the National Comprehensive Cancer Network : JNCCN* 2016, **14**(4):450-473.
14. [157]. Danielli R, Ridolfi R, Chiarion-Sileni V, Queirolo P, Testori A, Plummer R, Boitano M, Calabro L, Rossi CD, Giacomo AM *et al*: **Ipilimumab in pretreated patients with metastatic uveal melanoma: safety and clinical efficacy**. *Cancer immunology, immunotherapy : CII* 2012, **61**(1):41-48.
15. [158]. de Coana YP, Wolodarski M, Poschke I, Yoshimoto Y, Yang Y, Nystrom M, Edback U, Brage SE, Lundqvist A, Masucci GV *et al*: **Ipilimumab treatment decreases monocytic MDSCs and increases CD8 effector memory T cells in long-term survivors with advanced melanoma**. *Oncotarget* 2017, **8**(13):21539-21553.
16. [159]. Dequen P, Lorigan P, Jansen JP, van Baardewijk M, Ouwens MJ, Kotapati S: **Systematic review and network meta-analysis of overall survival comparing 3 mg/kg ipilimumab with alternative therapies in the management of pretreated patients with unresectable stage III or IV melanoma**. *The oncologist* 2012, **17**(11):1376-1385.
17. [160]. Di Giacomo AM, Ascierto PA, Pilla L, Santinami M, Ferrucci PF, Giannarelli D, Marasco A, Rivoltini L, Simeone E, Nicoletti SV *et al*: **Ipilimumab and fotemustine in patients with advanced melanoma (NIBIT-M1): an open-label, single-arm phase 2 trial**. *The Lancet Oncology* 2012, **13**(9):879-886.
18. [161]. Di Giacomo AM, Ascierto PA, Queirolo P, Pilla L, Ridolfi R, Santinami M, Testori A, Simeone E, Guidoboni M, Maurichi A *et al*: **Three-year follow-up of advanced melanoma patients who received ipilimumab plus fotemustine in the Italian Network for Tumor Biotherapy (NIBIT)-M1 phase II study**. *Annals of oncology : official journal of the European Society for Medical Oncology* 2015, **26**(4):798-803.
19. [162]. Di Giacomo AM, Danielli R, Calabro L, Bertocci E, Nannicini C, Giannarelli D, Balestrazzi A, Vigni F, Riversi V, Miracco C *et al*: **Ipilimumab experience in heavily pretreated patients with melanoma in an expanded access program at the University Hospital of Siena (Italy)**. *Cancer immunology, immunotherapy : CII* 2011, **60**(4):467-477.
20. [163]. Eggermont AM, Chiarion-Sileni V, Grob JJ, Dummer R, Wolchok JD, Schmidt H, Hamid O, Robert C, Ascierto PA, Richards JM *et al*: **Prolonged Survival in Stage III Melanoma with Ipilimumab Adjuvant Therapy**. *The New England journal of medicine* 2016, **375**(19):1845-1855.
21. [164]. Eggermont AM, Chiarion-Sileni V, Grob JJ, Dummer R, Wolchok JD, Schmidt H, Hamid O, Robert C, Ascierto PA, Richards JM *et al*: **Adjuvant ipilimumab versus placebo after complete resection of high-risk stage III melanoma (EORTC 18071): a randomised, double-blind, phase 3 trial**. *The Lancet Oncology* 2015, **16**(5):522-530.
22. [165]. Feng Y, Masson E, Dai D, Parker SM, Berman D, Roy A: **Model-based clinical pharmacology profiling of ipilimumab in patients with advanced melanoma**. *British journal of clinical pharmacology* 2014, **78**(1):106-117.
23. [166]. Feng Y, Roy A, Masson E, Chen TT, Humphrey R, Weber JS: **Exposure-response relationships of the efficacy and safety of ipilimumab in patients with advanced melanoma**. *Clinical cancer research : an official journal of the American Association for Cancer Research* 2013, **19**(14):3977-3986.
24. [167]. Flaherty L, Hamid O, Linette G, Schuchter L, Hallmeyer S, Gonzalez R, Cowey CL, Pavlick A, Kudrik F, Curti B *et al*: **A single-arm, open-label, expanded access study of vemurafenib in patients with metastatic melanoma in the United States**. *Cancer journal (Sudbury, Mass)* 2014, **20**(1):18-24.
25. [168]. Govindan R, Szczesna A, Ahn MJ, Schneider CP, Gonzalez Mella PF, Barlesi F, Han B, Ganea DE, Von Pawel J, Vladimirov V *et al*: **Phase III Trial of Ipilimumab Combined With Paclitaxel and Carboplatin in Advanced Squamous Non-Small-Cell Lung Cancer**. *Journal of clinical oncology : official journal of the American Society of Clinical Oncology* 2017, **35**(30):3449-3457.
26. [169]. Hamid O, Robert C, Daud A, Hodi FS, Hwu WJ, Kefford R, Wolchok JD, Hersey P, Joseph RW, Weber JS *et al*: **Safety and tumor responses with lambrolizumab (anti-PD-1) in melanoma**. *The New England journal of medicine* 2013, **369**(2):134-144.
27. [170]. Hamid O, Schmidt H, Nissan A, Ridolfi L, Aamdal S, Hansson J, Guida M, Hyams DM, Gomez H, Bastholt L *et al*: **A prospective phase II trial exploring the association between tumor microenvironment biomarkers and clinical activity of ipilimumab in advanced melanoma**. *Journal of translational medicine* 2011, **9**:204.
28. [171]. Hao C, Tian J, Liu H, Li F, Niu H, Zhu B: **Efficacy and safety of anti-PD-1 and anti-PD-1 combined with anti-CTLA-4 immunotherapy to advanced melanoma: A systematic review and meta-analysis of randomized controlled trials**. *Medicine* 2017, **96**(26):e7325.
29. [172]. Hatswell AJ, Pennington B, Pericleous L, Rowen D, Lebmeier M, Lee D: **Patient-reported utilities in advanced or metastatic melanoma, including analysis of utilities by time to death**. *Health and quality of life outcomes* 2014, **12**:140.
30. [173]. Hersh EM, O'Day SJ, Powderly J, Khan KD, Pavlick AC, Cranmer LD, Samlowski WE, Nichol GM, Yellin MJ, Weber JS: **A phase II multicenter study of ipilimumab with or without dacarbazine in chemotherapy-naive patients with advanced melanoma**. *Investigational new drugs* 2011, **29**(3):489-498.
31. [174]. Hiniker SM, Reddy SA, Maecker HT, Subrahmanyam PB, Rosenberg-Hasson Y, Swetter SM, Saha S, Shura L, Knox SJ: **A Prospective Clinical Trial Combining Radiation Therapy With Systemic Immunotherapy in Metastatic Melanoma**. *International journal of radiation oncology, biology, physics* 2016, **96**(3):578-588.
32. [175]. Hodi FS, Chesney J, Pavlick AC, Robert C, Grossmann KF, McDermott DF, Linette GP, Meyer N, Giguere JK, Agarwala SS *et al*: **Combined nivolumab and ipilimumab versus ipilimumab alone in patients with advanced melanoma: 2-year overall survival outcomes in a multicentre, randomised, controlled, phase 2 trial**. *The Lancet Oncology* 2016, **17**(11):1558-1568.
33. [176]. Hodi FS, Lawrence D, Lezcano C, Wu X, Zhou J, Sasada T, Zeng W, Giobbie-Hurder A, Atkins MB, Ibrahim N *et al*: **Bevacizumab plus ipilimumab in patients with metastatic melanoma**. *Cancer immunology research* 2014, **2**(7):632-642.
34. [177]. Hodi FS, Lee S, McDermott DF, Rao UN, Butterfield LH, Tarhini AA, Leming P, Puzanov I, Shin D, Kirkwood JM: **Ipilimumab plus sargramostim vs ipilimumab alone for treatment of metastatic melanoma: a randomized clinical trial**. *Jama* 2014, **312**(17):1744-1753.
35. [178]. Iwama S, De Remigis A, Callahan MK, Slovin SF, Wolchok JD, Caturegli P: **Pituitary expression of CTLA-4 mediates hypophysitis secondary to administration of CTLA-4 blocking antibody**. *Science translational medicine* 2014, **6**(230):230ra245.
36. [179]. Jansen YJ, Janssens P, Hoorens A, Schreuer MS, Seremet T, Wilgenhof S, Neyns B: **Granulomatous nephritis and dermatitis in a patient with BRAF V600E mutant metastatic melanoma treated with dabrafenib and trametinib**. *Melanoma research* 2015, **25**(6):550-554.
37. [180]. Ji RR, Chasalow SD, Wang L, Hamid O, Schmidt H, Cogswell J, Alaparthy S, Berman D, Jure-Kunkel M, Siemers NO *et al*: **An immune-active tumor microenvironment favors clinical response to ipilimumab**. *Cancer immunology, immunotherapy : CII* 2012, **61**(7):1019-1031.
38. [181]. Johnson DB, Friedman DL, Berry E, Decker I, Ye F, Zhao S, Morgans AK, Puzanov I, Sosman JA, Lovly CM: **Survivorship in Immune Therapy: Assessing Chronic Immune Toxicities, Health Outcomes, and Functional Status among Long-term Ipilimumab Survivors at a Single Referral Center**. *Cancer immunology research* 2015, **3**(5):464-469.
39. [182]. Kelderman S, Heemskerk B, van Tinteren H, van den Brom RR, Hospers GA, van den Eertwegh AJ, Kapiteijn EW, de Groot JW, Soetekouw P, Jansen RL *et al*: **Lactate dehydrogenase as a selection criterion for ipilimumab treatment in metastatic melanoma**. *Cancer immunology, immunotherapy : CII* 2014, **63**(5):449-458.
40. [183]. Kitano S, Postow MA, Ziegler CG, Kuk D, Panageas KS, Cortez C, Rasalan T, Adamow M, Yuan J, Wong P *et al*: **Computational algorithm-driven evaluation of monocytic myeloid-derived suppressor cell frequency for prediction of clinical outcomes**. *Cancer immunology research* 2014, **2**(8):812-821.
41. [184]. Koguchi Y, Hoen HM, Bambina SA, Rynning MD, Fuerstenberg RK, Curti BD, Urba WJ, Milburn C, Bahjat FR, Korman AJ *et al*: **Serum Immunoregulatory Proteins as Predictors of Overall Survival of Metastatic Melanoma Patients Treated with Ipilimumab**. *Cancer research* 2015, **75**(23):5084-5092.
42. [185]. Larkin J, Chiarion-Sileni V, Gonzalez R, Grob JJ, Cowey CL, Lao CD, Schadendorf D, Dummer R, Smylie M, Rutkowski P *et al*: **Combined Nivolumab and Ipilimumab or Monotherapy in Untreated Melanoma**. *The New England journal of medicine* 2015, **373**(1):23-34.
43. [186]. Larkin J, Hatswell AJ, Nathan P, Lebmeier M, Lee D: **The Predicted Impact of Ipilimumab Usage on Survival in Previously Treated Advanced or Metastatic Melanoma in the UK**. *PloS one* 2015, **10**(12):e0145524.
44. [187]. Larkin J, Lao CD, Urba WJ, McDermott DF, Horak C, Jiang J, Wolchok JD: **Efficacy and Safety of Nivolumab in Patients With BRAF V600 Mutant and BRAF Wild-Type Advanced Melanoma: A Pooled Analysis of 4 Clinical Trials**. *JAMA oncology* 2015, **1**(4):433-440.
45. [188]. Lebbe C, Weber JS, Maio M, Neyns B, Harmankaya K, Hamid O, O'Day SJ, Konto C, Cykowski L, McHenry MB *et al*: **Survival follow-up and ipilimumab retreatment of patients with advanced melanoma who received ipilimumab in prior phase II studies**. *Annals of oncology : official journal of the European Society for Medical Oncology* 2014, **25**(11):2277-2284.
46. [189]. Leccia MT, Planchamp F, Sassolas B, Combemale P, Modiano P, Bedane C, Cupissol D, Derrey S, Dygai-Cochet I, Lamant L *et al*: **[Management of patients with metastatic cutaneous melanoma: French national guidelines. French National Cancer Institute]**. *Annales de dermatologie et de venereologie* 2014, **141**(2):111-121.
47. [190]. Lee D, Porter J, Hertel N, Hatswell AJ, Briggs A: **Modelling Comparative Efficacy of Drugs with Different Survival Profiles: Ipilimumab, Vemurafenib and Dacarbazine in Advanced Melanoma**. *BioDrugs : clinical immunotherapeutics, biopharmaceuticals and gene therapy* 2016, **30**(4):307-319.
48. [191]. Long GV, Atkinson V, Cebon JS, Jameson MB, Fitzharris BM, McNeil CM, Hill AG, Ribas A, Atkins MB, Thompson JA *et al*: **Standard-dose pembrolizumab in combination with reduced-dose ipilimumab for patients with advanced melanoma (KEYNOTE-029): an open-label, phase 1b trial**. *The Lancet Oncology* 2017, **18**(9):1202-1210.
49. [192]. Lotem M, Merims S, Frank S, Hamburger T, Nissan A, Kadouri L, Cohen J, Straussman R, Eisenberg G, Frankenburg S *et al*: **Adjuvant Autologous Melanoma Vaccine for Macroscopic Stage III Disease: Survival, Biomarkers, and Improved Response to CTLA-4 Blockade**. *Journal of immunology research* 2016, **2016**:8121985.
50. [193]. Lynch TJ, Bondarenko I, Luft A, Serwatowski P, Barlesi F, Chacko R, Sebastian M, Neal J, Lu H, Cuillerot JM *et al*: **Ipilimumab in combination with paclitaxel and carboplatin as first-line treatment in stage IIIB/IV non-small-cell lung cancer: results from a randomized, double-blind, multicenter phase II study**. *Journal of clinical oncology : official journal of the American Society of Clinical Oncology* 2012, **30**(17):2046-2054.
51. [194]. Maio M, Grob JJ, Aamdal S, Bondarenko I, Robert C, Thomas L, Garbe C, Chiarion-Sileni V, Testori A, Chen TT *et al*: **Five-year survival rates for treatment-naive patients with advanced melanoma who received ipilimumab plus dacarbazine in a phase III trial**. *Journal of clinical oncology : official journal of the American Society of Clinical Oncology* 2015, **33**(10):1191-1196.
52. [195]. Margolin K, Ernstoff MS, Hamid O, Lawrence D, McDermott D, Puzanov I, Wolchok JD, Clark JI, Sznol M, Logan TF *et al*: **Ipilimumab in patients with melanoma and brain metastases: an open-label, phase 2 trial**. *The Lancet Oncology* 2012, **13**(5):459-465.
53. [196]. McDermott D, Haanen J, Chen TT, Lorigan P, O'Day S: **Efficacy and safety of ipilimumab in metastatic melanoma patients surviving more than 2 years following treatment in a phase III trial (MDX010-20)**. *Annals of oncology : official journal of the European Society for Medical Oncology* 2013, **24**(10):2694-2698.
54. [197]. Merchant MS, Wright M, Baird K, Wexler LH, Rodriguez-Galindo C, Bernstein D, Delbrook C, Lodish M, Bishop R, Wolchok JD *et al*: **Phase I Clinical Trial of Ipilimumab in Pediatric Patients with Advanced Solid Tumors**. *Clinical cancer research : an official journal of the American Association for Cancer Research* 2016, **22**(6):1364-1370.
55. [198]. Nosrati A, Tsai KK, Goldinger SM, Tumeh P, Grimes B, Loo K, Algazi AP, Nguyen-Kim TDL, Levesque M, Dummer R *et al*: **Evaluation of clinicopathological factors in PD-1 response: derivation and validation of a prediction scale for response to PD-1 monotherapy**. *British journal of cancer* 2017, **116**(9):1141-1147.
56. [199]. Patel KR, Shoukat S, Oliver DE, Chowdhary M, Rizzo M, Lawson DH, Khosa F, Liu Y, Khan MK: **Ipilimumab and Stereotactic Radiosurgery Versus Stereotactic Radiosurgery Alone for Newly Diagnosed Melanoma Brain Metastases**. *American journal of clinical oncology* 2017, **40**(5):444-450.
57. [200]. Petrelli F, Coinu A, Cabiddu M, Borgonovo K, Ghilardi M, Lonati V, Barni S: **Early analysis of surrogate endpoints for metastatic melanoma in immune checkpoint inhibitor trials**. *Medicine* 2016, **95**(26):e3997.
58. [201]. Pico de Coana Y, Poschke I, Gentilcore G, Mao Y, Nystrom M, Hansson J, Masucci GV, Kiessling R: **Ipilimumab treatment results in an early decrease in the frequency of circulating granulocytic myeloid-derived suppressor cells as well as their Arginase1 production**. *Cancer immunology research* 2013, **1**(3):158-162.
59. [202]. Postow MA, Chesney J, Pavlick AC, Robert C, Grossmann K, McDermott D, Linette GP, Meyer N, Giguere JK, Agarwala SS *et al*: **Nivolumab and ipilimumab versus ipilimumab in untreated melanoma**. *The New England journal of medicine* 2015, **372**(21):2006-2017.
60. [203]. Puzanov I, Milhem MM, Minor D, Hamid O, Li A, Chen L, Chastain M, Gorski KS, Anderson A, Chou J *et al*: **Talimogene Laherparepvec in Combination With Ipilimumab in Previously Untreated, Unresectable Stage IIIB-IV Melanoma**. *Journal of clinical oncology : official journal of the American Society of Clinical Oncology* 2016, **34**(22):2619-2626.
61. [204]. Quinn C, Ma Q, Kudlac A, Palmer S, Barber B, Zhao Z: **Indirect Treatment Comparison of Talimogene Laherparepvec Compared with Ipilimumab and Vemurafenib for the Treatment of Patients with Metastatic Melanoma**. *Advances in therapy* 2016, **33**(4):643-657.
62. [205]. Reck M, Bondarenko I, Luft A, Serwatowski P, Barlesi F, Chacko R, Sebastian M, Lu H, Cuillerot JM, Lynch TJ: **Ipilimumab in combination with paclitaxel and carboplatin as first-line therapy in extensive-disease-small-cell lung cancer: results from a randomized, double-blind, multicenter phase 2 trial**. *Annals of oncology : official journal of the European Society for Medical Oncology* 2013, **24**(1):75-83.
63. [206]. Revicki DA, van den Eertwegh AJ, Lorigan P, Lebbe C, Linette G, Ottensmeier CH, Safikhani S, Messina M, Hoos A, Wagner S *et al*: **Health related quality of life outcomes for unresectable stage III or IV melanoma patients receiving ipilimumab treatment**. *Health and quality of life outcomes* 2012, **10**:66.
64. [207]. Ribas A, Chesney JA, Gordon MS, Abernethy AP, Logan TF, Lawson DH, Chmielowksi B, Glaspy JA, Lewis K, Huang B *et al*: **Safety profile and pharmacokinetic analyses of the anti-CTLA4 antibody tremelimumab administered as a one hour infusion**. *Journal of translational medicine* 2012, **10**:236.
65. [208]. Ribas A, Hamid O, Daud A, Hodi FS, Wolchok JD, Kefford R, Joshua AM, Patnaik A, Hwu WJ, Weber JS *et al*: **Association of Pembrolizumab With Tumor Response and Survival Among Patients With Advanced Melanoma**. *Jama* 2016, **315**(15):1600-1609.
66. [209]. Ribas A, Hodi FS, Callahan M, Konto C, Wolchok J: **Hepatotoxicity with combination of vemurafenib and ipilimumab**. *The New England journal of medicine* 2013, **368**(14):1365-1366.
67. [210]. Ribas A, Kefford R, Marshall MA, Punt CJ, Haanen JB, Marmol M, Garbe C, Gogas H, Schachter J, Linette G *et al*: **Phase III randomized clinical trial comparing tremelimumab with standard-of-care chemotherapy in patients with advanced melanoma**. *Journal of clinical oncology : official journal of the American Society of Clinical Oncology* 2013, **31**(5):616-622.
68. [211]. Ribas A, Puzanov I, Dummer R, Schadendorf D, Hamid O, Robert C, Hodi FS, Schachter J, Pavlick AC, Lewis KD *et al*: **Pembrolizumab versus investigator-choice chemotherapy for ipilimumab-refractory melanoma (KEYNOTE-002): a randomised, controlled, phase 2 trial**. *The Lancet Oncology* 2015, **16**(8):908-918.
69. [212]. Robert C, Long GV, Brady B, Dutriaux C, Maio M, Mortier L, Hassel JC, Rutkowski P, McNeil C, Kalinka-Warzocha E *et al*: **Nivolumab in previously untreated melanoma without BRAF mutation**. *The New England journal of medicine* 2015, **372**(4):320-330.
70. [213]. Robert C, Ribas A, Wolchok JD, Hodi FS, Hamid O, Kefford R, Weber JS, Joshua AM, Hwu WJ, Gangadhar TC *et al*: **Anti-programmed-death-receptor-1 treatment with pembrolizumab in ipilimumab-refractory advanced melanoma: a randomised dose-comparison cohort of a phase 1 trial**. *Lancet (London, England)* 2014, **384**(9948):1109-1117.
71. [214]. Robert C, Schachter J, Long GV, Arance A, Grob JJ, Mortier L, Daud A, Carlino MS, McNeil C, Lotem M *et al*: **Pembrolizumab versus Ipilimumab in Advanced Melanoma**. *The New England journal of medicine* 2015, **372**(26):2521-2532.
72. [215]. Robert C, Schadendorf D, Messina M, Hodi FS, O'Day S: **Efficacy and safety of retreatment with ipilimumab in patients with pretreated advanced melanoma who progressed after initially achieving disease control**. *Clinical cancer research : an official journal of the American Association for Cancer Research* 2013, **19**(8):2232-2239.
73. [216]. Robert C, Thomas L, Bondarenko I, O'Day S, Weber J, Garbe C, Lebbe C, Baurain JF, Testori A, Grob JJ *et al*: **Ipilimumab plus dacarbazine for previously untreated metastatic melanoma**. *The New England journal of medicine* 2011, **364**(26):2517-2526.
74. [217]. Saenger Y, Magidson J, Liaw B, de Moll E, Harcharik S, Fu Y, Wassmann K, Fisher D, Kirkwood J, Oh WK *et al*: **Blood mRNA expression profiling predicts survival in patients treated with tremelimumab**. *Clinical cancer research : an official journal of the American Association for Cancer Research* 2014, **20**(12):3310-3318.
75. [218]. Sarnaik AA, Yu B, Yu D, Morelli D, Hall M, Bogle D, Yan L, Targan S, Solomon J, Nichol G *et al*: **Extended dose ipilimumab with a peptide vaccine: immune correlates associated with clinical benefit in patients with resected high-risk stage IIIc/IV melanoma**. *Clinical cancer research : an official journal of the American Association for Cancer Research* 2011, **17**(4):896-906.
76. [219]. Schadendorf D, Dummer R, Hauschild A, Robert C, Hamid O, Daud A, van den Eertwegh A, Cranmer L, O'Day S, Puzanov I *et al*: **Health-related quality of life in the randomised KEYNOTE-002 study of pembrolizumab versus chemotherapy in patients with ipilimumab-refractory melanoma**. *European journal of cancer (Oxford, England : 1990)* 2016, **67**:46-54.
77. [220]. Schadendorf D, Hodi FS, Robert C, Weber JS, Margolin K, Hamid O, Patt D, Chen TT, Berman DM, Wolchok JD: **Pooled Analysis of Long-Term Survival Data From Phase II and Phase III Trials of Ipilimumab in Unresectable or Metastatic Melanoma**. *Journal of clinical oncology : official journal of the American Society of Clinical Oncology* 2015, **33**(17):1889-1894.
78. [221]. Schadendorf D, Larkin J, Wolchok J, Hodi FS, Chiarion-Sileni V, Gonzalez R, Rutkowski P, Grob JJ, Cowey CL, Lao C *et al*: **Health-related quality of life results from the phase III CheckMate 067 study**. *European journal of cancer (Oxford, England : 1990)* 2017, **82**:80-91.
79. [222]. Shahabi V, Berman D, Chasalow SD, Wang L, Tsuchihashi Z, Hu B, Panting L, Jure-Kunkel M, Ji RR: **Gene expression profiling of whole blood in ipilimumab-treated patients for identification of potential biomarkers of immune-related gastrointestinal adverse events**. *Journal of translational medicine* 2013, **11**:75.
80. [223]. Shahabi V, Whitney G, Hamid O, Schmidt H, Chasalow SD, Alaparthy S, Jackson JR: **Assessment of association between BRAF-V600E mutation status in melanomas and clinical response to ipilimumab**. *Cancer immunology, immunotherapy : CII* 2012, **61**(5):733-737.
81. [224]. Sherrill B, Wang J, Kotapati S, Chin K: **Q-TWiST analysis comparing ipilimumab/dacarbazine vs placebo/dacarbazine for patients with stage III/IV melanoma**. *British journal of cancer* 2013, **109**(1):8-13.
82. [225]. Shimanovsky A, Dasanu CA: **Updates on immunotherapy in non-small cell lung cancer**. *Expert opinion on biological therapy* 2014, **14**(4):411-418.
83. [226]. Thompson JA, Hamid O, Minor D, Amin A, Ron IG, Ridolfi R, Assi H, Berman D, Siegel J, Weber JS: **Ipilimumab in treatment-naive and previously treated patients with metastatic melanoma: retrospective analysis of efficacy and safety data from a phase II trial**. *Journal of immunotherapy (Hagerstown, Md : 1997)* 2012, **35**(1):73-77.
84. [227]. Weber J, Gibney G, Kudchadkar R, Yu B, Cheng P, Martinez AJ, Kroeger J, Richards A, McCormick L, Moberg V *et al*: **Phase I/II Study of Metastatic Melanoma Patients Treated with Nivolumab Who Had Progressed after Ipilimumab**. *Cancer immunology research* 2016, **4**(4):345-353.
85. [228]. Weber J, Hamid O, Amin A, O'Day S, Masson E, Goldberg SM, Williams D, Parker SM, Chasalow SD, Alaparthy S *et al*: **Randomized phase I pharmacokinetic study of ipilimumab with or without one of two different chemotherapy regimens in patients with untreated advanced melanoma**. *Cancer immunity* 2013, **13**:7.
86. [229]. Weber JS, Amin A, Minor D, Siegel J, Berman D, O'Day SJ: **Safety and clinical activity of ipilimumab in melanoma patients with brain metastases: retrospective analysis of data from a phase 2 trial**. *Melanoma research* 2011, **21**(6):530-534.
87. [230]. Weber JS, D'Angelo SP, Minor D, Hodi FS, Gutzmer R, Neyns B, Hoeller C, Khushalani NI, Miller WH, Jr., Lao CD *et al*: **Nivolumab versus chemotherapy in patients with advanced melanoma who progressed after anti-CTLA-4 treatment (CheckMate 037): a randomised, controlled, open-label, phase 3 trial**. *The Lancet Oncology* 2015, **16**(4):375-384.
88. [231]. Weber JS, Dummer R, de Pril V, Lebbe C, Hodi FS: **Patterns of onset and resolution of immune-related adverse events of special interest with ipilimumab: detailed safety analysis from a phase 3 trial in patients with advanced melanoma**. *Cancer* 2013, **119**(9):1675-1682.
89. [232]. Weber JS, Gibney G, Sullivan RJ, Sosman JA, Slingluff CL, Jr., Lawrence DP, Logan TF, Schuchter LM, Nair S, Fecher L *et al*: **Sequential administration of nivolumab and ipilimumab with a planned switch in patients with advanced melanoma (CheckMate 064): an open-label, randomised, phase 2 trial**. *The Lancet Oncology* 2016, **17**(7):943-955.
90. [233]. Weber JS, Hamid O, Chasalow SD, Wu DY, Parker SM, Galbraith S, Gnjatic S, Berman D: **Ipilimumab increases activated T cells and enhances humoral immunity in patients with advanced melanoma**. *Journal of immunotherapy (Hagerstown, Md : 1997)* 2012, **35**(1):89-97.
91. [234]. Weber JS, Kudchadkar RR, Yu B, Gallenstein D, Horak CE, Inzunza HD, Zhao X, Martinez AJ, Wang W, Gibney G *et al*: **Safety, efficacy, and biomarkers of nivolumab with vaccine in ipilimumab-refractory or -naive melanoma**. *Journal of clinical oncology : official journal of the American Society of Clinical Oncology* 2013, **31**(34):4311-4318.
92. [235]. Wilgenhof S, Corthals J, Heirman C, van Baren N, Lucas S, Kvistborg P, Thielemans K, Neyns B: **Phase II Study of Autologous Monocyte-Derived mRNA Electroporated Dendritic Cells (TriMixDC-MEL) Plus Ipilimumab in Patients With Pretreated Advanced Melanoma**. *Journal of clinical oncology : official journal of the American Society of Clinical Oncology* 2016, **34**(12):1330-1338.
93. [236]. Williams NL, Wuthrick EJ, Kim H, Palmer JD, Garg S, Eldredge-Hindy H, Daskalakis C, Feeney KJ, Mastrangelo MJ, Kim LJ *et al*: **Phase 1 Study of Ipilimumab Combined With Whole Brain Radiation Therapy or Radiosurgery for Melanoma Patients With Brain Metastases**. *International journal of radiation oncology, biology, physics* 2017, **99**(1):22-30.
94. [237]. Wolchok JD, Chiarion-Sileni V, Gonzalez R, Rutkowski P, Grob JJ, Cowey CL, Lao CD, Wagstaff J, Schadendorf D, Ferrucci PF *et al*: **Overall Survival with Combined Nivolumab and Ipilimumab in Advanced Melanoma**. *The New England journal of medicine* 2017, **377**(14):1345-1356.
95. [238]. Wolchok JD, Kluger H, Callahan MK, Postow MA, Rizvi NA, Lesokhin AM, Segal NH, Ariyan CE, Gordon RA, Reed K *et al*: **Nivolumab plus ipilimumab in advanced melanoma**. *The New England journal of medicine* 2013, **369**(2):122-133.
96. [239]. Wolchok JD, Weber JS, Maio M, Neyns B, Harmankaya K, Chin K, Cykowski L, de Pril V, Humphrey R, Lebbe C: **Four-year survival rates for patients with metastatic melanoma who received ipilimumab in phase II clinical trials**. *Annals of oncology : official journal of the European Society for Medical Oncology* 2013, **24**(8):2174-2180.
97. [240]. Wu Y, Shi H, Jiang M, Qiu M, Jia K, Cao T, Shang Y, Shi L, Jiang K, Wu H: **The clinical value of combination of immune checkpoint inhibitors in cancer patients: A meta-analysis of efficacy and safety**. *International journal of cancer* 2017, **141**(12):2562-2570.
98. [241]. Yamazaki N, Kiyohara Y, Uhara H, Fukushima S, Uchi H, Shibagaki N, Tsutsumida A, Yoshikawa S, Okuyama R, Ito Y *et al*: **Phase II study of ipilimumab monotherapy in Japanese patients with advanced melanoma**. *Cancer chemotherapy and pharmacology* 2015, **76**(5):997-1004.
99. [242]. Yamazaki N, Uhara H, Fukushima S, Uchi H, Shibagaki N, Kiyohara Y, Tsutsumida A, Namikawa K, Okuyama R, Otsuka Y *et al*: **Phase II study of the immune-checkpoint inhibitor ipilimumab plus dacarbazine in Japanese patients with previously untreated, unresectable or metastatic melanoma**. *Cancer chemotherapy and pharmacology* 2015, **76**(5):969-975.
100. [243]. Yuan J, Ginsberg B, Page D, Li Y, Rasalan T, Gallardo HF, Xu Y, Adams S, Bhardwaj N, Busam K *et al*: **CTLA-4 blockade increases antigen-specific CD8(+) T cells in prevaccinated patients with melanoma: three cases**. *Cancer immunology, immunotherapy : CII* 2011, **60**(8):1137-1146.
101. [244]. Yuan J, Zhou J, Dong Z, Tandon S, Kuk D, Panageas KS, Wong P, Wu X, Naidoo J, Page DB *et al*: **Pretreatment serum VEGF is associated with clinical response and overall survival in advanced melanoma patients treated with ipilimumab**. *Cancer immunology research* 2014, **2**(2):127-132.
102. [245]. Zaragoza J, Caille A, Beneton N, Bens G, Christiann F, Maillard H, Machet L: **High neutrophil to lymphocyte ratio measured before starting ipilimumab treatment is associated with reduced overall survival in patients with melanoma**. *The British journal of dermatology* 2016, **174**(1):146-151.
103. [246]. Zhang S, Liang F, Li W, Wang Q: **Risk of treatment-related mortality in cancer patients treated with ipilimumab: A systematic review and meta-analysis**. *European journal of cancer (Oxford, England : 1990)* 2017, **83**:71-79.
104. [247]. Zimmer L, Apuri S, Eroglu Z, Kottschade LA, Forschner A, Gutzmer R, Schlaak M, Heinzerling L, Krackhardt AM, Loquai C *et al*: **Ipilimumab alone or in combination with nivolumab after progression on anti-PD-1 therapy in advanced melanoma**. *European journal of cancer (Oxford, England : 1990)* 2017, **75**:47-55.
105. [248]. Zimmer L, Eigentler TK, Kiecker F, Simon J, Utikal J, Mohr P, Berking C, Kampgen E, Dippel E, Stadler R *et al*: **Open-label, multicenter, single-arm phase II DeCOG-study of ipilimumab in pretreated patients with different subtypes of metastatic melanoma**. *Journal of translational medicine* 2015, **13**:351.
106. [249]. Zimmer L, Vaubel J, Mohr P, Hauschild A, Utikal J, Simon J, Garbe C, Herbst R, Enk A, Kampgen E *et al*: **Phase II DeCOG-study of ipilimumab in pretreated and treatment-naive patients with metastatic uveal melanoma**. *PloS one* 2015, **10**(3):e0118564.

# TOPIC: Melanoma and Vermurafenib

1. [250]. Abdel-Rahman O, ElHalawani H, Ahmed H: **Doublet BRAF/MEK inhibition versus single-agent BRAF inhibition in the management of BRAF-mutant advanced melanoma, biological rationale and meta-analysis of published data**. *Clinical & translational oncology : official publication of the Federation of Spanish Oncology Societies and of the National Cancer Institute of Mexico* 2016, **18**(8):848-858.
2. [251]. Alloo A, Garibyan L, LeBoeuf N, Lin G, Werchniak A, Hodi FS, Jr., Flaherty KT, Lawrence DP, Lin JY: **Photodynamic therapy for multiple eruptive keratoacanthomas associated with vemurafenib treatment for metastatic melanoma**. *Archives of dermatology* 2012, **148**(3):363-366.
3. [252]. Anderson S, Bloom KJ, Vallera DU, Rueschoff J, Meldrum C, Schilling R, Kovach B, Lee JR, Ochoa P, Langland R *et al*: **Multisite analytic performance studies of a real-time polymerase chain reaction assay for the detection of BRAF V600E mutations in formalin-fixed, paraffin-embedded tissue specimens of malignant melanoma**. *Archives of pathology & laboratory medicine* 2012, **136**(11):1385-1391.
4. [253]. Anforth R, Carlos G, Clements A, Kefford R, Fernandez-Penas P: **Cutaneous adverse events in patients treated with BRAF inhibitor-based therapies for metastatic melanoma for longer than 52 weeks**. *The British journal of dermatology* 2015, **172**(1):239-243.
5. [254]. Arance AM, Berrocal A, Lopez-Martin JA, de la Cruz-Merino L, Soriano V, Martin Algarra S, Alonso L, Cerezuela P, La Orden B, Espinosa E: **Safety of vemurafenib in patients with BRAF V600 mutated metastatic melanoma: the Spanish experience**. *Clinical & translational oncology : official publication of the Federation of Spanish Oncology Societies and of the National Cancer Institute of Mexico* 2016, **18**(11):1147-1157.
6. [255]. Ascierto PA, McArthur GA, Dreno B, Atkinson V, Liszkay G, Di Giacomo AM, Mandala M, Demidov L, Stroyakovskiy D, Thomas L *et al*: **Cobimetinib combined with vemurafenib in advanced BRAF(V600)-mutant melanoma (coBRIM): updated efficacy results from a randomised, double-blind, phase 3 trial**. *The Lancet Oncology* 2016, **17**(9):1248-1260.
7. [256]. Beck D, Niessner H, Smalley KS, Flaherty K, Paraiso KH, Busch C, Sinnberg T, Vasseur S, Iovanna JL, Driessen S *et al*: **Vemurafenib potently induces endoplasmic reticulum stress-mediated apoptosis in BRAFV600E melanoma cells**. *Science signaling* 2013, **6**(260):ra7.
8. [257]. Blank CU, Larkin J, Arance AM, Hauschild A, Queirolo P, Del Vecchio M, Ascierto PA, Krajsova I, Schachter J, Neyns B *et al*: **Open-label, multicentre safety study of vemurafenib in 3219 patients with BRAFV600 mutation-positive metastatic melanoma: 2-year follow-up data and long-term responders' analysis**. *European journal of cancer (Oxford, England : 1990)* 2017, **79**:176-184.
9. [258]. Brose MS, Cabanillas ME, Cohen EE, Wirth LJ, Riehl T, Yue H, Sherman SI, Sherman EJ: **Vemurafenib in patients with BRAF(V600E)-positive metastatic or unresectable papillary thyroid cancer refractory to radioactive iodine: a non-randomised, multicentre, open-label, phase 2 trial**. *The Lancet Oncology* 2016, **17**(9):1272-1282.
10. [259]. Chapman PB, Hauschild A, Robert C, Haanen JB, Ascierto P, Larkin J, Dummer R, Garbe C, Testori A, Maio M *et al*: **Improved survival with vemurafenib in melanoma with BRAF V600E mutation**. *The New England journal of medicine* 2011, **364**(26):2507-2516.
11. [260]. Choe CH, McArthur GA, Caro I, Kempen JH, Amaravadi RK: **Ocular toxicity in BRAF mutant cutaneous melanoma patients treated with vemurafenib**. *American journal of ophthalmology* 2014, **158**(4):831-837.e832.
12. [261]. Debarbieux S, Dalle S, Depaepe L, Poulalhon N, Balme B, Thomas L: **Second primary melanomas treated with BRAF blockers: study by reflectance confocal microscopy**. *The British journal of dermatology* 2013, **168**(6):1230-1235.
13. [262]. Del Vecchio M, Ascierto PA, Mandala M, Sileni VC, Maio M, Di Guardo L, Simeone E, Queirolo P: **Vemurafenib in BRAFV600 mutated metastatic melanoma: a subanalysis of the Italian population of a global safety study**. *Future oncology (London, England)* 2015, **11**(9):1355-1362.
14. [263]. Dika E, Patrizi A, Ribero S, Fanti PA, Starace M, Melotti B, Sperandi F, Piraccini BM: **Hair and nail adverse events during treatment with targeted therapies for metastatic melanoma**. *European journal of dermatology : EJD* 2016, **26**(3):232-239.
15. [264]. Dummer R, Goldinger SM, Turtschi CP, Eggmann NB, Michielin O, Mitchell L, Veronese L, Hilfiker PR, Felderer L, Rinderknecht JD: **Vemurafenib in patients with BRAF(V600) mutation-positive melanoma with symptomatic brain metastases: final results of an open-label pilot study**. *European journal of cancer (Oxford, England : 1990)* 2014, **50**(3):611-621.
16. [265]. Falchook GS, Long GV, Kurzrock R, Kim KB, Arkenau TH, Brown MP, Hamid O, Infante JR, Millward M, Pavlick AC *et al*: **Dabrafenib in patients with melanoma, untreated brain metastases, and other solid tumours: a phase 1 dose-escalation trial**. *Lancet (London, England)* 2012, **379**(9829):1893-1901.
17. [266]. Flaherty KT, Hennig M, Lee SJ, Ascierto PA, Dummer R, Eggermont AM, Hauschild A, Kefford R, Kirkwood JM, Long GV *et al*: **Surrogate endpoints for overall survival in metastatic melanoma: a meta-analysis of randomised controlled trials**. *The Lancet Oncology* 2014, **15**(3):297-304.
18. [267]. Frederick DT, Salas Fragomeni RA, Schalck A, Ferreiro-Neira I, Hoff T, Cooper ZA, Haq R, Panka DJ, Kwong LN, Davies MA *et al*: **Clinical profiling of BCL-2 family members in the setting of BRAF inhibition offers a rationale for targeting de novo resistance using BH3 mimetics**. *PloS one* 2014, **9**(7):e101286.
19. [268]. Frouin E, Guillot B, Larrieux M, Tempier A, Boulle N, Foulongne V, Girard C, Costes V, Solassol J: **Cutaneous epithelial tumors induced by vemurafenib involve the MAPK and Pi3KCA pathways but not HPV nor HPyV viral infection**. *PloS one* 2014, **9**(10):e110478.
20. [269]. Goppner D, Muller J, Kruger S, Franke I, Gollnick H, Quist SR: **High incidence of naevi-associated BRAF wild-type melanoma and dysplastic naevi under treatment with the class I BRAF inhibitor vemurafenib**. *Acta dermato-venereologica* 2014, **94**(5):517-520.
21. [270]. Grippo JF, Zhang W, Heinzmann D, Yang KH, Wong J, Joe AK, Munster P, Sarapa N, Daud A: **A phase I, randomized, open-label study of the multiple-dose pharmacokinetics of vemurafenib in patients with BRAF V600E mutation-positive metastatic melanoma**. *Cancer chemotherapy and pharmacology* 2014, **73**(1):103-111.
22. [271]. Grob JJ, Amonkar MM, Karaszewska B, Schachter J, Dummer R, Mackiewicz A, Stroyakovskiy D, Drucis K, Grange F, Chiarion-Sileni V *et al*: **Comparison of dabrafenib and trametinib combination therapy with vemurafenib monotherapy on health-related quality of life in patients with unresectable or metastatic cutaneous BRAF Val600-mutation-positive melanoma (COMBI-v): results of a phase 3, open-label, randomised trial**. *The Lancet Oncology* 2015, **16**(13):1389-1398.
23. [272]. Harding JJ, Catalanotti F, Munhoz RR, Cheng DT, Yaqubie A, Kelly N, McDermott GC, Kersellius R, Merghoub T, Lacouture ME *et al*: **A Retrospective Evaluation of Vemurafenib as Treatment for BRAF-Mutant Melanoma Brain Metastases**. *The oncologist* 2015, **20**(7):789-797.
24. [273]. Haroche J, Cohen-Aubart F, Emile JF, Maksud P, Drier A, Toledano D, Barete S, Charlotte F, Cluzel P, Donadieu J *et al*: **Reproducible and sustained efficacy of targeted therapy with vemurafenib in patients with BRAF(V600E)-mutated Erdheim-Chester disease**. *Journal of clinical oncology : official journal of the American Society of Clinical Oncology* 2015, **33**(5):411-418.
25. [274]. Hyman DM, Puzanov I, Subbiah V, Faris JE, Chau I, Blay JY, Wolf J, Raje NS, Diamond EL, Hollebecque A *et al*: **Vemurafenib in Multiple Nonmelanoma Cancers with BRAF V600 Mutations**. *The New England journal of medicine* 2015, **373**(8):726-736.
26. [275]. Johnson DB, Menzies AM, Zimmer L, Eroglu Z, Ye F, Zhao S, Rizos H, Sucker A, Scolyer RA, Gutzmer R *et al*: **Acquired BRAF inhibitor resistance: A multicenter meta-analysis of the spectrum and frequencies, clinical behaviour, and phenotypic associations of resistance mechanisms**. *European journal of cancer (Oxford, England : 1990)* 2015, **51**(18):2792-2799.
27. [276]. Jubb AM, Ribas A, Sosman JA, McArthur GA, Yan Y, Rost S, Zhao S, Koeppen H: **Impact of MET expression on outcome in BRAF(V600E/K) advanced melanoma**. *Histopathology* 2013, **63**(3):351-361.
28. [277]. Kim KB, Cabanillas ME, Lazar AJ, Williams MD, Sanders DL, Ilagan JL, Nolop K, Lee RJ, Sherman SI: **Clinical responses to vemurafenib in patients with metastatic papillary thyroid cancer harboring BRAF(V600E) mutation**. *Thyroid : official journal of the American Thyroid Association* 2013, **23**(10):1277-1283.
29. [278]. Kopetz S, Desai J, Chan E, Hecht JR, O'Dwyer PJ, Maru D, Morris V, Janku F, Dasari A, Chung W *et al*: **Phase II Pilot Study of Vemurafenib in Patients With Metastatic BRAF-Mutated Colorectal Cancer**. *Journal of clinical oncology : official journal of the American Society of Clinical Oncology* 2015, **33**(34):4032-4038.
30. [279]. Larkin J, Ascierto PA, Dreno B, Atkinson V, Liszkay G, Maio M, Mandala M, Demidov L, Stroyakovskiy D, Thomas L *et al*: **Combined vemurafenib and cobimetinib in BRAF-mutated melanoma**. *The New England journal of medicine* 2014, **371**(20):1867-1876.
31. [280]. Larkin J, Del Vecchio M, Ascierto PA, Krajsova I, Schachter J, Neyns B, Espinosa E, Garbe C, Sileni VC, Gogas H *et al*: **Vemurafenib in patients with BRAF(V600) mutated metastatic melanoma: an open-label, multicentre, safety study**. *The Lancet Oncology* 2014, **15**(4):436-444.
32. [281]. Matter-Walstra K, Braun R, Kolb C, Ademi Z, Dummer R, Pestalozzi BC, Schwenkglenks M: **A cost-effectiveness analysis of trametinib plus dabrafenib as first-line therapy for metastatic BRAF V600-mutated melanoma in the Swiss setting**. *The British journal of dermatology* 2015, **173**(6):1462-1470.
33. [282]. McArthur GA, Chapman PB, Robert C, Larkin J, Haanen JB, Dummer R, Ribas A, Hogg D, Hamid O, Ascierto PA *et al*: **Safety and efficacy of vemurafenib in BRAF(V600E) and BRAF(V600K) mutation-positive melanoma (BRIM-3): extended follow-up of a phase 3, randomised, open-label study**. *The Lancet Oncology* 2014, **15**(3):323-332.
34. [283]. McArthur GA, Puzanov I, Amaravadi R, Ribas A, Chapman P, Kim KB, Sosman JA, Lee RJ, Nolop K, Flaherty KT *et al*: **Marked, homogeneous, and early [18F]fluorodeoxyglucose-positron emission tomography responses to vemurafenib in BRAF-mutant advanced melanoma**. *Journal of clinical oncology : official journal of the American Society of Clinical Oncology* 2012, **30**(14):1628-1634.
35. [284]. Perier-Muzet M, Thomas L, Poulalhon N, Debarbieux S, Bringuier PP, Duru G, Depaepe L, Balme B, Dalle S: **Melanoma patients under vemurafenib: prospective follow-up of melanocytic lesions by digital dermoscopy**. *The Journal of investigative dermatology* 2014, **134**(5):1351-1358.
36. [285]. Ponti G, Pellacani G, Tomasi A, Gelsomino F, Spallanzani A, Depenni R, Al Jalbout S, Simi L, Garagnani L, Borsari S *et al*: **The somatic affairs of BRAF: tailored therapies for advanced malignant melanoma and orphan non-V600E (V600R-M) mutations**. *Journal of clinical pathology* 2013, **66**(5):441-445.
37. [286]. Puzanov I, Amaravadi RK, McArthur GA, Flaherty KT, Chapman PB, Sosman JA, Ribas A, Shackleton M, Hwu P, Chmielowski B *et al*: **Long-term outcome in BRAF(V600E) melanoma patients treated with vemurafenib: Patterns of disease progression and clinical management of limited progression**. *European journal of cancer (Oxford, England : 1990)* 2015, **51**(11):1435-1443.
38. [287]. Ribas A, Gonzalez R, Pavlick A, Hamid O, Gajewski TF, Daud A, Flaherty L, Logan T, Chmielowski B, Lewis K *et al*: **Combination of vemurafenib and cobimetinib in patients with advanced BRAF(V600)-mutated melanoma: a phase 1b study**. *The Lancet Oncology* 2014, **15**(9):954-965.
39. [288]. Ribas A, Zhang W, Chang I, Shirai K, Ernstoff MS, Daud A, Cowey CL, Daniels G, Seja E, O'Laco E *et al*: **The effects of a high-fat meal on single-dose vemurafenib pharmacokinetics**. *Journal of clinical pharmacology* 2014, **54**(4):368-374.
40. [289]. Rinderknecht JD, Goldinger SM, Rozati S, Kamarashev J, Kerl K, French LE, Dummer R, Belloni B: **RASopathic skin eruptions during vemurafenib therapy**. *PloS one* 2013, **8**(3):e58721.
41. [290]. Robert C, Karaszewska B, Schachter J, Rutkowski P, Mackiewicz A, Stroiakovski D, Lichinitser M, Dummer R, Grange F, Mortier L *et al*: **Improved overall survival in melanoma with combined dabrafenib and trametinib**. *The New England journal of medicine* 2015, **372**(1):30-39.
42. [291]. Romero AI, Chaput N, Poirier-Colame V, Rusakiewicz S, Jacquelot N, Chaba K, Mortier E, Jacques Y, Caillat-Zucman S, Flament C *et al*: **Regulation of CD4(+)NKG2D(+) Th1 cells in patients with metastatic melanoma treated with sorafenib: role of IL-15Ralpha and NKG2D triggering**. *Cancer research* 2014, **74**(1):68-80.
43. [292]. Schadendorf D, Long GV, Stroiakovski D, Karaszewska B, Hauschild A, Levchenko E, Chiarion-Sileni V, Schachter J, Garbe C, Dutriaux C *et al*: **Three-year pooled analysis of factors associated with clinical outcomes across dabrafenib and trametinib combination therapy phase 3 randomised trials**. *European journal of cancer (Oxford, England : 1990)* 2017, **82**:45-55.
44. [293]. Sosman JA, Kim KB, Schuchter L, Gonzalez R, Pavlick AC, Weber JS, McArthur GA, Hutson TE, Moschos SJ, Flaherty KT *et al*: **Survival in BRAF V600-mutant advanced melanoma treated with vemurafenib**. *The New England journal of medicine* 2012, **366**(8):707-714.
45. [294]. Trunzer K, Pavlick AC, Schuchter L, Gonzalez R, McArthur GA, Hutson TE, Moschos SJ, Flaherty KT, Kim KB, Weber JS *et al*: **Pharmacodynamic effects and mechanisms of resistance to vemurafenib in patients with metastatic melanoma**. *Journal of clinical oncology : official journal of the American Society of Clinical Oncology* 2013, **31**(14):1767-1774.
46. [295]. Yamazaki N, Kiyohara Y, Sugaya N, Uhara H: **Phase I/II study of vemurafenib in patients with unresectable or recurrent melanoma with BRAF(V) (600) mutations**. *The Journal of dermatology* 2015, **42**(7):661-666.
47. [296]. Zimmer L, Livingstone E, Hillen U, Domkes S, Becker A, Schadendorf D: **Panniculitis with arthralgia in patients with melanoma treated with selective BRAF inhibitors and its management**. *Archives of dermatology* 2012, **148**(3):357-361.

# TOPIC: Melanoma and BRAF

1. [297]. Adjei AA, Cohen RB, Franklin W, Morris C, Wilson D, Molina JR, Hanson LJ, Gore L, Chow L, Leong S *et al*: **Phase I pharmacokinetic and pharmacodynamic study of the oral, small-molecule mitogen-activated protein kinase kinase 1/2 inhibitor AZD6244 (ARRY-142886) in patients with advanced cancers**. *Journal of clinical oncology : official journal of the American Society of Clinical Oncology* 2008, **26**(13):2139-2146.
2. [298]. Ahmad T, Eisen T: **Kinase inhibition with BAY 43-9006 in renal cell carcinoma**. *Clinical cancer research : an official journal of the American Association for Cancer Research* 2004, **10**(18 Pt 2):6388s-6392s.
3. [299]. Algazi AP, Cha E, Ortiz-Urda SM, McCalmont T, Bastian BC, Hwang J, Pampaloni MH, Behr S, Chong K, Cortez B *et al*: **The combination of axitinib followed by paclitaxel/carboplatin yields extended survival in advanced BRAF wild-type melanoma: results of a clinical/correlative prospective phase II clinical trial**. *British journal of cancer* 2015, **112**(8):1326-1331.
4. [300]. Algazi AP, Weber JS, Andrews SC, Urbas P, Munster PN, DeConti RC, Hwang J, Sondak VK, Messina JL, McCalmont T *et al*: **Phase I clinical trial of the Src inhibitor dasatinib with dacarbazine in metastatic melanoma**. *British journal of cancer* 2012, **106**(1):85-91.
5. [301]. Anforth RM, Blumetti TC, Kefford RF, Sharma R, Scolyer RA, Kossard S, Long GV, Fernandez-Penas P: **Cutaneous manifestations of dabrafenib (GSK2118436): a selective inhibitor of mutant BRAF in patients with metastatic melanoma**. *The British journal of dermatology* 2012, **167**(5):1153-1160.
6. [302]. Anwar MA, Murad F, Dawson E, Abd Elmageed ZY, Tsumagari K, Kandil E: **Immunohistochemistry as a reliable method for detection of BRAF-V600E mutation in melanoma: a systematic review and meta-analysis of current published literature**. *The Journal of surgical research* 2016, **203**(2):407-415.
7. [303]. Ascierto PA, Minor D, Ribas A, Lebbe C, O'Hagan A, Arya N, Guckert M, Schadendorf D, Kefford RF, Grob JJ *et al*: **Phase II trial (BREAK-2) of the BRAF inhibitor dabrafenib (GSK2118436) in patients with metastatic melanoma**. *Journal of clinical oncology : official journal of the American Society of Clinical Oncology* 2013, **31**(26):3205-3211.
8. [304]. Ascierto PA, Schadendorf D, Berking C, Agarwala SS, van Herpen CM, Queirolo P, Blank CU, Hauschild A, Beck JT, St-Pierre A *et al*: **MEK162 for patients with advanced melanoma harbouring NRAS or Val600 BRAF mutations: a non-randomised, open-label phase 2 study**. *The Lancet Oncology* 2013, **14**(3):249-256.
9. [305]. Banerji U, Affolter A, Judson I, Marais R, Workman P: **BRAF and NRAS mutations in melanoma: potential relationships to clinical response to HSP90 inhibitors**. *Molecular cancer therapeutics* 2008, **7**(4):737-739.
10. [306]. Banerji U, Camidge DR, Verheul HM, Agarwal R, Sarker D, Kaye SB, Desar IM, Timmer-Bonte JN, Eckhardt SG, Lewis KD *et al*: **The first-in-human study of the hydrogen sulfate (Hyd-sulfate) capsule of the MEK1/2 inhibitor AZD6244 (ARRY-142886): a phase I open-label multicenter trial in patients with advanced cancer**. *Clinical cancer research : an official journal of the American Association for Cancer Research* 2010, **16**(5):1613-1623.
11. [307]. Beasley GM, Coleman AP, Raymond A, Sanders G, Selim MA, Peterson BL, Brady MS, Davies MA, Augustine C, Tyler DS: **A phase I multi-institutional study of systemic sorafenib in conjunction with regional melphalan for in-transit melanoma of the extremity**. *Annals of surgical oncology* 2012, **19**(12):3896-3905.
12. [308]. Board RE, Ellison G, Orr MC, Kemsley KR, McWalter G, Blockley LY, Dearden SP, Morris C, Ranson M, Cantarini MV *et al*: **Detection of BRAF mutations in the tumour and serum of patients enrolled in the AZD6244 (ARRY-142886) advanced melanoma phase II study**. *British journal of cancer* 2009, **101**(10):1724-1730.
13. [309]. Bollag G, Hirth P, Tsai J, Zhang J, Ibrahim PN, Cho H, Spevak W, Zhang C, Zhang Y, Habets G *et al*: **Clinical efficacy of a RAF inhibitor needs broad target blockade in BRAF-mutant melanoma**. *Nature* 2010, **467**(7315):596-599.
14. [310]. Carlino MS, Gowrishankar K, Saunders CA, Pupo GM, Snoyman S, Zhang XD, Saw R, Becker TM, Kefford RF, Long GV *et al*: **Antiproliferative effects of continued mitogen-activated protein kinase pathway inhibition following acquired resistance to BRAF and/or MEK inhibition in melanoma**. *Molecular cancer therapeutics* 2013, **12**(7):1332-1342.
15. [311]. Carlino MS, Saunders CA, Haydu LE, Menzies AM, Martin Curtis C, Jr., Lebowitz PF, Kefford RF, Long GV: **(18)F-labelled fluorodeoxyglucose-positron emission tomography (FDG-PET) heterogeneity of response is prognostic in dabrafenib treated BRAF mutant metastatic melanoma**. *European journal of cancer (Oxford, England : 1990)* 2013, **49**(2):395-402.
16. [312]. Catalanotti F, Solit DB, Pulitzer MP, Berger MF, Scott SN, Iyriboz T, Lacouture ME, Panageas KS, Wolchok JD, Carvajal RD *et al*: **Phase II trial of MEK inhibitor selumetinib (AZD6244, ARRY-142886) in patients with BRAFV600E/K-mutated melanoma**. *Clinical cancer research : an official journal of the American Association for Cancer Research* 2013, **19**(8):2257-2264.
17. [313]. Chen G, McQuade JL, Panka DJ, Hudgens CW, Amin-Mansour A, Mu XJ, Bahl S, Jane-Valbuena J, Wani KM, Reuben A *et al*: **Clinical, Molecular, and Immune Analysis of Dabrafenib-Trametinib Combination Treatment for BRAF Inhibitor-Refractory Metastatic Melanoma: A Phase 2 Clinical Trial**. *JAMA oncology* 2016, **2**(8):1056-1064.
18. [314]. Coit DG, Thompson JA, Andtbacka R, Anker CJ, Bichakjian CK, Carson WE, 3rd, Daniels GA, Daud A, Dimaio D, Fleming MD *et al*: **Melanoma, version 4.2014**. *Journal of the National Comprehensive Cancer Network : JNCCN* 2014, **12**(5):621-629.
19. [315]. Corcoran RB, Atreya CE, Falchook GS, Kwak EL, Ryan DP, Bendell JC, Hamid O, Messersmith WA, Daud A, Kurzrock R *et al*: **Combined BRAF and MEK Inhibition With Dabrafenib and Trametinib in BRAF V600-Mutant Colorectal Cancer**. *Journal of clinical oncology : official journal of the American Society of Clinical Oncology* 2015, **33**(34):4023-4031.
20. [316]. Coupe N, Corrie P, Hategan M, Larkin J, Gore M, Gupta A, Wise A, Suter S, Ciria C, Love S *et al*: **PACMEL: a phase 1 dose escalation trial of trametinib (GSK1120212) in combination with paclitaxel**. *European journal of cancer (Oxford, England : 1990)* 2015, **51**(3):359-366.
21. [317]. Cui C, Mao L, Chi Z, Si L, Sheng X, Kong Y, Li S, Lian B, Gu K, Tao M *et al*: **A phase II, randomized, double-blind, placebo-controlled multicenter trial of Endostar in patients with metastatic melanoma**. *Molecular therapy : the journal of the American Society of Gene Therapy* 2013, **21**(7):1456-1463.
22. [318]. Davies MA, Fox PS, Papadopoulos NE, Bedikian AY, Hwu WJ, Lazar AJ, Prieto VG, Culotta KS, Madden TL, Xu Q *et al*: **Phase I study of the combination of sorafenib and temsirolimus in patients with metastatic melanoma**. *Clinical cancer research : an official journal of the American Association for Cancer Research* 2012, **18**(4):1120-1128.
23. [319]. Davies MA, Saiag P, Robert C, Grob JJ, Flaherty KT, Arance A, Chiarion-Sileni V, Thomas L, Lesimple T, Mortier L *et al*: **Dabrafenib plus trametinib in patients with BRAFV600-mutant melanoma brain metastases (COMBI-MB): a multicentre, multicohort, open-label, phase 2 trial**. *The Lancet Oncology* 2017, **18**(7):863-873.
24. [320]. Devji T, Levine O, Neupane B, Beyene J, Xie F: **Systemic Therapy for Previously Untreated Advanced BRAF-Mutated Melanoma: A Systematic Review and Network Meta-Analysis of Randomized Clinical Trials**. *JAMA oncology* 2017, **3**(3):366-373.
25. [321]. Dickson MA, Gordon MS, Edelman G, Bendell JC, Kudchadkar RR, LoRusso PM, Johnston SH, Clary DO, Schwartz GK: **Phase I study of XL281 (BMS-908662), a potent oral RAF kinase inhibitor, in patients with advanced solid tumors**. *Investigational new drugs* 2015, **33**(2):349-356.
26. [322]. Dienstmann R, Lassen U, Cebon J, Desai J, Brown MP, Evers S, Su F, Zhang W, Boisserie F, Lestini B *et al*: **First-in-Man Dose-Escalation Study of the Selective BRAF Inhibitor RG7256 in Patients with BRAF V600-Mutated Advanced Solid Tumors**. *Targeted oncology* 2016, **11**(2):149-156.
27. [323]. Eisen T, Ahmad T, Flaherty KT, Gore M, Kaye S, Marais R, Gibbens I, Hackett S, James M, Schuchter LM *et al*: **Sorafenib in advanced melanoma: a Phase II randomised discontinuation trial analysis**. *British journal of cancer* 2006, **95**(5):581-586.
28. [324]. Falchook GS, Lewis KD, Infante JR, Gordon MS, Vogelzang NJ, DeMarini DJ, Sun P, Moy C, Szabo SA, Roadcap LT *et al*: **Activity of the oral MEK inhibitor trametinib in patients with advanced melanoma: a phase 1 dose-escalation trial**. *The Lancet Oncology* 2012, **13**(8):782-789.
29. [325]. Falchook GS, Long GV, Kurzrock R, Kim KB, Arkenau HT, Brown MP, Hamid O, Infante JR, Millward M, Pavlick A *et al*: **Dose selection, pharmacokinetics, and pharmacodynamics of BRAF inhibitor dabrafenib (GSK2118436)**. *Clinical cancer research : an official journal of the American Association for Cancer Research* 2014, **20**(17):4449-4458.
30. [326]. Ferrucci PF, Minchella I, Mosconi M, Gandini S, Verrecchia F, Cocorocchio E, Passoni C, Pari C, Testori A, Coco P *et al*: **Dacarbazine in combination with bevacizumab for the treatment of unresectable/metastatic melanoma: a phase II study**. *Melanoma research* 2015, **25**(3):239-245.
31. [327]. Flaherty KT, Infante JR, Daud A, Gonzalez R, Kefford RF, Sosman J, Hamid O, Schuchter L, Cebon J, Ibrahim N *et al*: **Combined BRAF and MEK inhibition in melanoma with BRAF V600 mutations**. *The New England journal of medicine* 2012, **367**(18):1694-1703.
32. [328]. Flaherty KT, Puzanov I, Kim KB, Ribas A, McArthur GA, Sosman JA, O'Dwyer PJ, Lee RJ, Grippo JF, Nolop K *et al*: **Inhibition of mutated, activated BRAF in metastatic melanoma**. *The New England journal of medicine* 2010, **363**(9):809-819.
33. [329]. Flaherty KT, Robert C, Hersey P, Nathan P, Garbe C, Milhem M, Demidov LV, Hassel JC, Rutkowski P, Mohr P *et al*: **Improved survival with MEK inhibition in BRAF-mutated melanoma**. *The New England journal of medicine* 2012, **367**(2):107-114.
34. [330]. Flaherty KT, Schiller J, Schuchter LM, Liu G, Tuveson DA, Redlinger M, Lathia C, Xia C, Petrenciuc O, Hingorani SR *et al*: **A phase I trial of the oral, multikinase inhibitor sorafenib in combination with carboplatin and paclitaxel**. *Clinical cancer research : an official journal of the American Association for Cancer Research* 2008, **14**(15):4836-4842.
35. [331]. Frankel AE, Eskiocak U, Gill JG, Yuan S, Ramesh V, Froehlich TW, Ahn C, Morrison SJ: **Digoxin Plus Trametinib Therapy Achieves Disease Control in BRAF Wild-Type Metastatic Melanoma Patients**. *Neoplasia (New York, NY)* 2017, **19**(4):255-260.
36. [332]. Galanis E, Markovic SN, Suman VJ, Nuovo GJ, Vile RG, Kottke TJ, Nevala WK, Thompson MA, Lewis JE, Rumilla KM *et al*: **Phase II trial of intravenous administration of Reolysin((R)) (Reovirus Serotype-3-dearing Strain) in patients with metastatic melanoma**. *Molecular therapy : the journal of the American Society of Gene Therapy* 2012, **20**(10):1998-2003.
37. [333]. Giuse NB, Kusnoor SV, Koonce TY, Naylor HM, Chen SC, Blasingame MN, Anderson IA, Micheel CM, Levy MA, Ye F *et al*: **Guiding Oncology Patients Through the Maze of Precision Medicine**. *Journal of health communication* 2016, **21 Suppl 1**:5-17.
38. [334]. Grob JJ, Amonkar MM, Martin-Algarra S, Demidov LV, Goodman V, Grotzinger K, Haney P, Kampgen E, Karaszewska B, Mauch C *et al*: **Patient perception of the benefit of a BRAF inhibitor in metastatic melanoma: quality-of-life analyses of the BREAK-3 study comparing dabrafenib with dacarbazine**. *Annals of oncology : official journal of the European Society for Medical Oncology* 2014, **25**(7):1428-1436.
39. [335]. Guillot B, Dalac S, Denis M, Dupuy A, Emile JF, De La Fouchardiere A, Hindie E, Jouary T, Lassau N, Mirabel X *et al*: **[Guidelines for stage I to III melanoma]**. *Bulletin du cancer* 2016, **103**(9):743-752.
40. [336]. Gupta A, Love S, Schuh A, Shanyinde M, Larkin JM, Plummer R, Nathan PD, Danson S, Ottensmeier CH, Lorigan P *et al*: **DOC-MEK: a double-blind randomized phase II trial of docetaxel with or without selumetinib in wild-type BRAF advanced melanoma**. *Annals of oncology : official journal of the European Society for Medical Oncology* 2014, **25**(5):968-974.
41. [337]. Haas NB, Quirt I, Hotte S, McWhirter E, Polintan R, Litwin S, Adams PD, McBryan T, Wang L, Martin LP *et al*: **Phase II trial of vorinostat in advanced melanoma**. *Investigational new drugs* 2014, **32**(3):526-534.
42. [338]. Hauschild A, Grob JJ, Demidov LV, Jouary T, Gutzmer R, Millward M, Rutkowski P, Blank CU, Miller WH, Jr., Kaempgen E *et al*: **Dabrafenib in BRAF-mutated metastatic melanoma: a multicentre, open-label, phase 3 randomised controlled trial**. *Lancet (London, England)* 2012, **380**(9839):358-365.
43. [339]. Henary H, Hong DS, Falchook GS, Tsimberidou A, George GC, Wen S, Wheler J, Fu S, Naing A, Piha-Paul S *et al*: **Melanoma patients in a phase I clinic: molecular aberrations, targeted therapy and outcomes**. *Annals of oncology : official journal of the European Society for Medical Oncology* 2013, **24**(8):2158-2165.
44. [340]. Herceg D, Buzina DS, Ceovic R, Dotlic S, Ilic I, Orehovec SS, Herceg GH, Mijatovic D, Separovic R, Silovski T *et al*: **[CROATIAN SOCIETY FOR MEDICAL ONCOLOGY CLINICAL GUIDELINES FOR DIAGNOSIS, TREATMENT AND FOLLOW-UP OF PATIENTS WITH MELANOMA]**. *Lijecnicki vjesnik* 2016, **138**(1-2):22-29.
45. [341]. Hersh EM, Del Vecchio M, Brown MP, Kefford R, Loquai C, Testori A, Bhatia S, Gutzmer R, Conry R, Haydon A *et al*: **A randomized, controlled phase III trial of nab-Paclitaxel versus dacarbazine in chemotherapy-naive patients with metastatic melanoma**. *Annals of oncology : official journal of the European Society for Medical Oncology* 2015, **26**(11):2267-2274.
46. [342]. Hong DS, Vence L, Falchook G, Radvanyi LG, Liu C, Goodman V, Legos JJ, Blackman S, Scarmadio A, Kurzrock R *et al*: **BRAF(V600) inhibitor GSK2118436 targeted inhibition of mutant BRAF in cancer patients does not impair overall immune competency**. *Clinical cancer research : an official journal of the American Association for Cancer Research* 2012, **18**(8):2326-2335.
47. [343]. Jeck WR, Parker J, Carson CC, Shields JM, Sambade MJ, Peters EC, Burd CE, Thomas NE, Chiang DY, Liu W *et al*: **Targeted next generation sequencing identifies clinically actionable mutations in patients with melanoma**. *Pigment cell & melanoma research* 2014, **27**(4):653-663.
48. [344]. Johnson AS, Crandall H, Dahlman K, Kelley MC: **Preliminary results from a prospective trial of preoperative combined BRAF and MEK-targeted therapy in advanced BRAF mutation-positive melanoma**. *Journal of the American College of Surgeons* 2015, **220**(4):581-593.e581.
49. [345]. Johnson DB, Flaherty KT, Weber JS, Infante JR, Kim KB, Kefford RF, Hamid O, Schuchter L, Cebon J, Sharfman WH *et al*: **Combined BRAF (Dabrafenib) and MEK inhibition (Trametinib) in patients with BRAFV600-mutant melanoma experiencing progression with single-agent BRAF inhibitor**. *Journal of clinical oncology : official journal of the American Society of Clinical Oncology* 2014, **32**(33):3697-3704.
50. [346]. Kelley MC: **Immune Responses to BRAF-Targeted Therapy in Melanoma: Is Targeted Therapy Immunotherapy?** *Critical reviews in oncogenesis* 2016, **21**(1-2):83-91.
51. [347]. Kim J, Lazar AJ, Davies MA, Homsi J, Papadopoulos NE, Hwu WJ, Bedikian AY, Woodman SE, Patel SP, Hwu P *et al*: **BRAF, NRAS and KIT sequencing analysis of spindle cell melanoma**. *Journal of cutaneous pathology* 2012, **39**(9):821-825.
52. [348]. Kim KB, Kefford R, Pavlick AC, Infante JR, Ribas A, Sosman JA, Fecher LA, Millward M, McArthur GA, Hwu P *et al*: **Phase II study of the MEK1/MEK2 inhibitor Trametinib in patients with metastatic BRAF-mutant cutaneous melanoma previously treated with or without a BRAF inhibitor**. *Journal of clinical oncology : official journal of the American Society of Clinical Oncology* 2013, **31**(4):482-489.
53. [349]. Kim SY, Kim SN, Hahn HJ, Lee YW, Choe YB, Ahn KJ: **Metaanalysis of BRAF mutations and clinicopathologic characteristics in primary melanoma**. *Journal of the American Academy of Dermatology* 2015, **72**(6):1036-1046.e1032.
54. [350]. Kirkwood JM, Bastholt L, Robert C, Sosman J, Larkin J, Hersey P, Middleton M, Cantarini M, Zazulina V, Kemsley K *et al*: **Phase II, open-label, randomized trial of the MEK1/2 inhibitor selumetinib as monotherapy versus temozolomide in patients with advanced melanoma**. *Clinical cancer research : an official journal of the American Association for Cancer Research* 2012, **18**(2):555-567.
55. [351]. Lassen UN, Meulendijks D, Siu LL, Karanikas V, Mau-Sorensen M, Schellens JH, Jonker DJ, Hansen AR, Simcox ME, Schostack KJ *et al*: **A phase I monotherapy study of RG7212, a first-in-class monoclonal antibody targeting TWEAK signaling in patients with advanced cancers**. *Clinical cancer research : an official journal of the American Association for Cancer Research* 2015, **21**(2):258-266.
56. [352]. Latimer NR, Abrams KR, Amonkar MM, Stapelkamp C, Swann RS: **Adjusting for the Confounding Effects of Treatment Switching-The BREAK-3 Trial: Dabrafenib Versus Dacarbazine**. *The oncologist* 2015, **20**(7):798-805.
57. [353]. Latimer NR, Amonkar MM, Stapelkamp C, Sun P: **Adjusting for confounding effects of treatment switching in a randomized phase II study of dabrafenib plus trametinib in BRAF V600+ metastatic melanoma**. *Melanoma research* 2015, **25**(6):528-536.
58. [354]. Latimer NR, Bell H, Abrams KR, Amonkar MM, Casey M: **Adjusting for treatment switching in the METRIC study shows further improved overall survival with trametinib compared with chemotherapy**. *Cancer medicine* 2016, **5**(5):806-815.
59. [355]. Lee CI, Menzies AM, Haydu LE, Azer M, Clements A, Kefford RF, Long GV: **Features and management of pyrexia with combined dabrafenib and trametinib in metastatic melanoma**. *Melanoma research* 2014, **24**(5):468-474.
60. [356]. Lee JH, Choi JW, Kim YS: **Frequencies of BRAF and NRAS mutations are different in histological types and sites of origin of cutaneous melanoma: a meta-analysis**. *The British journal of dermatology* 2011, **164**(4):776-784.
61. [357]. Long GV, Stroyakovskiy D, Gogas H, Levchenko E, de Braud F, Larkin J, Garbe C, Jouary T, Hauschild A, Grob JJ *et al*: **Combined BRAF and MEK inhibition versus BRAF inhibition alone in melanoma**. *The New England journal of medicine* 2014, **371**(20):1877-1888.
62. [358]. Long GV, Stroyakovskiy D, Gogas H, Levchenko E, de Braud F, Larkin J, Garbe C, Jouary T, Hauschild A, Grob JJ *et al*: **Dabrafenib and trametinib versus dabrafenib and placebo for Val600 BRAF-mutant melanoma: a multicentre, double-blind, phase 3 randomised controlled trial**. *Lancet (London, England)* 2015, **386**(9992):444-451.
63. [359]. Long GV, Trefzer U, Davies MA, Kefford RF, Ascierto PA, Chapman PB, Puzanov I, Hauschild A, Robert C, Algazi A *et al*: **Dabrafenib in patients with Val600Glu or Val600Lys BRAF-mutant melanoma metastatic to the brain (BREAK-MB): a multicentre, open-label, phase 2 trial**. *The Lancet Oncology* 2012, **13**(11):1087-1095.
64. [360]. Long GV, Weber JS, Infante JR, Kim KB, Daud A, Gonzalez R, Sosman JA, Hamid O, Schuchter L, Cebon J *et al*: **Overall Survival and Durable Responses in Patients With BRAF V600-Mutant Metastatic Melanoma Receiving Dabrafenib Combined With Trametinib**. *Journal of clinical oncology : official journal of the American Society of Clinical Oncology* 2016, **34**(8):871-878.
65. [361]. LoRusso PM, Boerner SA, Pilat MJ, Forman KM, Zuccaro CY, Kiefer JA, Liang WS, Hunsberger S, Redman BG, Markovic SN *et al*: **Pilot Trial of Selecting Molecularly Guided Therapy for Patients with Non-V600 BRAF-Mutant Metastatic Melanoma: Experience of the SU2C/MRA Melanoma Dream Team**. *Molecular cancer therapeutics* 2015, **14**(8):1962-1971.
66. [362]. Ludeke I, Terheyden P, Grisanti S, Luke M: **[Development of serous retinopathy during therapy of a metastatic cutaneous melanoma]**. *Der Ophthalmologe : Zeitschrift der Deutschen Ophthalmologischen Gesellschaft* 2016, **113**(10):861-863.
67. [363]. Mahalingam D, Malik L, Beeram M, Rodon J, Sankhala K, Mita A, Benjamin D, Ketchum N, Michalek J, Tolcher A *et al*: **Phase II study evaluating the efficacy, safety, and pharmacodynamic correlative study of dual antiangiogenic inhibition using bevacizumab in combination with sorafenib in patients with advanced malignant melanoma**. *Cancer chemotherapy and pharmacology* 2014, **74**(1):77-84.
68. [364]. Mai R, Zhou S, Zhong W, Rong S, Cong Z, Li Y, Xie Q, Chen H, Li X, Liu S *et al*: **Therapeutic efficacy of combined BRAF and MEK inhibition in metastatic melanoma: a comprehensive network meta-analysis of randomized controlled trials**. *Oncotarget* 2015, **6**(29):28502-28512.
69. [365]. Martin-Algarra S, Fernandez-Figueras MT, Lopez-Martin JA, Santos-Briz A, Arance A, Lozano MD, Berrocal A, Rios-Martin JJ, Espinosa E, Rodriguez-Peralto JL: **Guidelines for biomarker testing in metastatic melanoma: a National Consensus of the Spanish Society of Pathology and the Spanish Society of Medical Oncology**. *Clinical & translational oncology : official publication of the Federation of Spanish Oncology Societies and of the National Cancer Institute of Mexico* 2014, **16**(4):362-373.
70. [366]. Martinez-Garcia M, Banerji U, Albanell J, Bahleda R, Dolly S, Kraeber-Bodere F, Rojo F, Routier E, Guarin E, Xu ZX *et al*: **First-in-human, phase I dose-escalation study of the safety, pharmacokinetics, and pharmacodynamics of RO5126766, a first-in-class dual MEK/RAF inhibitor in patients with solid tumors**. *Clinical cancer research : an official journal of the American Association for Cancer Research* 2012, **18**(17):4806-4819.
71. [367]. Menzies AM, Ashworth MT, Swann S, Kefford RF, Flaherty K, Weber J, Infante JR, Kim KB, Gonzalez R, Hamid O *et al*: **Characteristics of pyrexia in BRAFV600E/K metastatic melanoma patients treated with combined dabrafenib and trametinib in a phase I/II clinical trial**. *Annals of oncology : official journal of the European Society for Medical Oncology* 2015, **26**(2):415-421.
72. [368]. Moreau S, Saiag P, Aegerter P, Bosset D, Longvert C, Helias-Rodzewicz Z, Marin C, Peschaud F, Chagnon S, Zimmermann U *et al*: **Prognostic value of BRAF(V(6)(0)(0)) mutations in melanoma patients after resection of metastatic lymph nodes**. *Annals of surgical oncology* 2012, **19**(13):4314-4321.
73. [369]. Nathanson KL, Martin AM, Wubbenhorst B, Greshock J, Letrero R, D'Andrea K, O'Day S, Infante JR, Falchook GS, Arkenau HT *et al*: **Tumor genetic analyses of patients with metastatic melanoma treated with the BRAF inhibitor dabrafenib (GSK2118436)**. *Clinical cancer research : an official journal of the American Association for Cancer Research* 2013, **19**(17):4868-4878.
74. [370]. O'Day SJ, Pavlick AC, Albertini MR, Hamid O, Schalch H, Lang Z, Ling J, Mata M, Reddy M, Foster B: **Clinical and pharmacologic evaluation of two dose levels of intetumumab (CNTO 95) in patients with melanoma or angiosarcoma**. *Investigational new drugs* 2012, **30**(3):1074-1081.
75. [371]. Ott PA, Hamilton A, Min C, Safarzadeh-Amiri S, Goldberg L, Yoon J, Yee H, Buckley M, Christos PJ, Wright JJ *et al*: **A phase II trial of sorafenib in metastatic melanoma with tissue correlates**. *PloS one* 2010, **5**(12):e15588.
76. [372]. Ouellet D, Gibiansky E, Leonowens C, O'Hagan A, Haney P, Switzky J, Goodman VL: **Population pharmacokinetics of dabrafenib, a BRAF inhibitor: effect of dose, time, covariates, and relationship with its metabolites**. *Journal of clinical pharmacology* 2014, **54**(6):696-706.
77. [373]. Ouellet D, Grossmann KF, Limentani G, Nebot N, Lan K, Knowles L, Gordon MS, Sharma S, Infante JR, Lorusso PM *et al*: **Effects of particle size, food, and capsule shell composition on the oral bioavailability of dabrafenib, a BRAF inhibitor, in patients with BRAF mutation-positive tumors**. *Journal of pharmaceutical sciences* 2013, **102**(9):3100-3109.
78. [374]. Ouellet D, Kassir N, Chiu J, Mouksassi MS, Leonowens C, Cox D, DeMarini DJ, Gardner O, Crist W, Patel K: **Population pharmacokinetics and exposure-response of trametinib, a MEK inhibitor, in patients with BRAF V600 mutation-positive melanoma**. *Cancer chemotherapy and pharmacology* 2016, **77**(4):807-817.
79. [375]. Pasquali S, Chiarion-Sileni V, Rossi CR, Mocellin S: **Immune checkpoint inhibitors and targeted therapies for metastatic melanoma: A network meta-analysis**. *Cancer treatment reviews* 2017, **54**:34-42.
80. [376]. Patel SP, Lazar AJ, Papadopoulos NE, Liu P, Infante JR, Glass MR, Vaughn CS, LoRusso PM, Cohen RB, Davies MA *et al*: **Clinical responses to selumetinib (AZD6244; ARRY-142886)-based combination therapy stratified by gene mutations in patients with metastatic melanoma**. *Cancer* 2013, **119**(4):799-805.
81. [377]. Planchard D, Besse B, Groen HJM, Souquet PJ, Quoix E, Baik CS, Barlesi F, Kim TM, Mazieres J, Novello S *et al*: **Dabrafenib plus trametinib in patients with previously treated BRAF(V600E)-mutant metastatic non-small cell lung cancer: an open-label, multicentre phase 2 trial**. *The Lancet Oncology* 2016, **17**(7):984-993.
82. [378]. Puntervoll HE, Molven A, Akslen LA: **Frequency of somatic BRAF mutations in melanocytic lesions from patients in a CDK4 melanoma family**. *Pigment cell & melanoma research* 2014, **27**(1):149-151.
83. [379]. Rangwala R, Leone R, Chang YC, Fecher LA, Schuchter LM, Kramer A, Tan KS, Heitjan DF, Rodgers G, Gallagher M *et al*: **Phase I trial of hydroxychloroquine with dose-intense temozolomide in patients with advanced solid tumors and melanoma**. *Autophagy* 2014, **10**(8):1369-1379.
84. [380]. Robert C, Dummer R, Gutzmer R, Lorigan P, Kim KB, Nyakas M, Arance A, Liszkay G, Schadendorf D, Cantarini M *et al*: **Selumetinib plus dacarbazine versus placebo plus dacarbazine as first-line treatment for BRAF-mutant metastatic melanoma: a phase 2 double-blind randomised study**. *The Lancet Oncology* 2013, **14**(8):733-740.
85. [381]. Safaee Ardekani G, Jafarnejad SM, Tan L, Saeedi A, Li G: **The prognostic value of BRAF mutation in colorectal cancer and melanoma: a systematic review and meta-analysis**. *PloS one* 2012, **7**(10):e47054.
86. [382]. Sarker D, Ang JE, Baird R, Kristeleit R, Shah K, Moreno V, Clarke PA, Raynaud FI, Levy G, Ware JA *et al*: **First-in-human phase I study of pictilisib (GDC-0941), a potent pan-class I phosphatidylinositol-3-kinase (PI3K) inhibitor, in patients with advanced solid tumors**. *Clinical cancer research : an official journal of the American Association for Cancer Research* 2015, **21**(1):77-86.
87. [383]. Schadendorf D, Amonkar MM, Milhem M, Grotzinger K, Demidov LV, Rutkowski P, Garbe C, Dummer R, Hassel JC, Wolter P *et al*: **Functional and symptom impact of trametinib versus chemotherapy in BRAF V600E advanced or metastatic melanoma: quality-of-life analyses of the METRIC study**. *Annals of oncology : official journal of the European Society for Medical Oncology* 2014, **25**(3):700-706.
88. [384]. Schadendorf D, Amonkar MM, Stroyakovskiy D, Levchenko E, Gogas H, de Braud F, Grob JJ, Bondarenko I, Garbe C, Lebbe C *et al*: **Health-related quality of life impact in a randomised phase III study of the combination of dabrafenib and trametinib versus dabrafenib monotherapy in patients with BRAF V600 metastatic melanoma**. *European journal of cancer (Oxford, England : 1990)* 2015, **51**(7):833-840.
89. [385]. Slingluff CL, Jr., Petroni GR, Molhoek KR, Brautigan DL, Chianese-Bullock KA, Shada AL, Smolkin ME, Olson WC, Gaucher A, Chase CM *et al*: **Clinical activity and safety of combination therapy with temsirolimus and bevacizumab for advanced melanoma: a phase II trial (CTEP 7190/Mel47)**. *Clinical cancer research : an official journal of the American Association for Cancer Research* 2013, **19**(13):3611-3620.
90. [386]. Solit DB, Osman I, Polsky D, Panageas KS, Daud A, Goydos JS, Teitcher J, Wolchok JD, Germino FJ, Krown SE *et al*: **Phase II trial of 17-allylamino-17-demethoxygeldanamycin in patients with metastatic melanoma**. *Clinical cancer research : an official journal of the American Association for Cancer Research* 2008, **14**(24):8302-8307.
91. [387]. Thurneysen S, Cheng PF, Nagel HW, Kunz M, Jaberg-Bentele N, Nageli M, Ziegler M, Guenova E, Goldinger SM, Mangana J *et al*: **An exploratory study investigating the metabolic activity and local cytokine profile in patients with melanoma treated with pazopanib and paclitaxel**. *The British journal of dermatology* 2016, **175**(5):966-978.
92. [388]. Tolcher AW, Patnaik A, Papadopoulos KP, Rasco DW, Becerra CR, Allred AJ, Orford K, Aktan G, Ferron-Brady G, Ibrahim N *et al*: **Phase I study of the MEK inhibitor trametinib in combination with the AKT inhibitor afuresertib in patients with solid tumors and multiple myeloma**. *Cancer chemotherapy and pharmacology* 2015, **75**(1):183-189.
93. [389]. von Moos R, Seifert B, Simcock M, Goldinger SM, Gillessen S, Ochsenbein A, Michielin O, Cathomas R, Schlappi M, Moch H *et al*: **First-line temozolomide combined with bevacizumab in metastatic melanoma: a multicentre phase II trial (SAKK 50/07)**. *Annals of oncology : official journal of the European Society for Medical Oncology* 2012, **23**(2):531-536.
94. [390]. Wagle N, Van Allen EM, Treacy DJ, Frederick DT, Cooper ZA, Taylor-Weiner A, Rosenberg M, Goetz EM, Sullivan RJ, Farlow DN *et al*: **MAP kinase pathway alterations in BRAF-mutant melanoma patients with acquired resistance to combined RAF/MEK inhibition**. *Cancer discovery* 2014, **4**(1):61-68.
95. [391]. Wilmott JS, Haydu LE, Menzies AM, Lum T, Hyman J, Thompson JF, Hersey P, Kefford RF, Scolyer RA, Long GV: **Dynamics of chemokine, cytokine, and growth factor serum levels in BRAF-mutant melanoma patients during BRAF inhibitor treatment**. *Journal of immunology (Baltimore, Md : 1950)* 2014, **192**(5):2505-2513.
96. [392]. Wilson MA, Zhao F, Khare S, Roszik J, Woodman SE, D'Andrea K, Wubbenhorst B, Rimm DL, Kirkwood JM, Kluger HM *et al*: **Copy Number Changes Are Associated with Response to Treatment with Carboplatin, Paclitaxel, and Sorafenib in Melanoma**. *Clinical cancer research : an official journal of the American Association for Cancer Research* 2016, **22**(2):374-382.
97. [393]. Wilson MA, Zhao F, Letrero R, D'Andrea K, Rimm DL, Kirkwood JM, Kluger HM, Lee SJ, Schuchter LM, Flaherty KT *et al*: **Correlation of somatic mutations and clinical outcome in melanoma patients treated with Carboplatin, Paclitaxel, and sorafenib**. *Clinical cancer research : an official journal of the American Association for Cancer Research* 2014, **20**(12):3328-3337.
98. [394]. Xia J, Jia P, Hutchinson KE, Dahlman KB, Johnson D, Sosman J, Pao W, Zhao Z: **A meta-analysis of somatic mutations from next generation sequencing of 241 melanomas: a road map for the study of genes with potential clinical relevance**. *Molecular cancer therapeutics* 2014, **13**(7):1918-1928.
99. [395]. Yamazaki N, Kiyohara Y, Uhara H, Uehara J, Fujimoto M, Takenouchi T, Otsuka M, Uchi H, Ihn H, Minami H: **Efficacy and safety of nivolumab in Japanese patients with previously untreated advanced melanoma: A phase II study**. *Cancer science* 2017, **108**(6):1223-1230.
100. [396]. Yip D, Le MN, Chan JL, Lee JH, Mehnert JA, Yudd A, Kempf J, Shih WJ, Chen S, Goydos JS: **A phase 0 trial of riluzole in patients with resectable stage III and IV melanoma**. *Clinical cancer research : an official journal of the American Association for Cancer Research* 2009, **15**(11):3896-3902.
101. [397]. Zimmer L, Barlesi F, Martinez-Garcia M, Dieras V, Schellens JH, Spano JP, Middleton MR, Calvo E, Paz-Ares L, Larkin J *et al*: **Phase I expansion and pharmacodynamic study of the oral MEK inhibitor RO4987655 (CH4987655) in selected patients with advanced cancer with RAS-RAF mutations**. *Clinical cancer research : an official journal of the American Association for Cancer Research* 2014, **20**(16):4251-4261.

# TOPIC: Melanoma and Dabrafenib

1. [398]. Kulkarni D, Song K, Briley L, King K, Dabrowski C, Mookerjee B, Legos J, Spraggs C: **Pyrexia in dabrafenib-treated melanoma patients is not associated with common genetic variation or HLA polymorphisms**. *Pharmacogenomics* 2016, **17**(5):459-462.

# TOPIC: Melanoma and Dacarbazine

1. [399]. **Veliparib plus temozolomide in metastatic melanoma trends toward increased PFS but results are not statistically significant**. *Oncology (Williston Park, NY)* 2011, **25**(12):1213, 1232.
2. [400]. Aarntzen EH, De Vries IJ, Lesterhuis WJ, Schuurhuis D, Jacobs JF, Bol K, Schreibelt G, Mus R, De Wilt JH, Haanen JB *et al*: **Targeting CD4(+) T-helper cells improves the induction of antitumor responses in dendritic cell-based vaccination**. *Cancer research* 2013, **73**(1):19-29.
3. [401]. Beasley GM, Speicher P, Augustine CK, Dolber PC, Peterson BL, Sharma K, Mosca PJ, Royal R, Ross M, Zager JS *et al*: **A multicenter phase I dose escalation trial to evaluate safety and tolerability of intra-arterial temozolomide for patients with advanced extremity melanoma using normothermic isolated limb infusion**. *Annals of surgical oncology* 2015, **22**(1):287-294.
4. [402]. Bedane C, Leccia MT, Sassolas B, Bregman B, Lebbe C: **Treatment patterns and outcomes in patients with advanced melanoma in France**. *Current medical research and opinion* 2013, **29**(10):1297-1305.
5. [403]. Bedikian AY, DeConti RC, Conry R, Agarwala S, Papadopoulos N, Kim KB, Ernstoff M: **Phase 3 study of docosahexaenoic acid-paclitaxel versus dacarbazine in patients with metastatic malignant melanoma**. *Annals of oncology : official journal of the European Society for Medical Oncology* 2011, **22**(4):787-793.
6. [404]. Bedikian AY, Garbe C, Conry R, Lebbe C, Grob JJ: **Dacarbazine with or without oblimersen (a Bcl-2 antisense oligonucleotide) in chemotherapy-naive patients with advanced melanoma and low-normal serum lactate dehydrogenase: 'The AGENDA trial'**. *Melanoma research* 2014, **24**(3):237-243.
7. [405]. Carvajal RD, Schwartz GK, Mann H, Smith I, Nathan PD: **Study design and rationale for a randomised, placebo-controlled, double-blind study to assess the efficacy of selumetinib (AZD6244; ARRY-142886) in combination with dacarbazine in patients with metastatic uveal melanoma (SUMIT)**. *BMC cancer* 2015, **15**:467.
8. [406]. Carvajal RD, Sosman JA, Quevedo JF, Milhem MM, Joshua AM, Kudchadkar RR, Linette GP, Gajewski TF, Lutzky J, Lawson DH *et al*: **Effect of selumetinib vs chemotherapy on progression-free survival in uveal melanoma: a randomized clinical trial**. *Jama* 2014, **311**(23):2397-2405.
9. [407]. Carvajal RD, Wong MK, Thompson JA, Gordon MS, Lewis KD, Pavlick AC, Wolchok JD, Rojas PB, Schwartz JD, Bedikian AY: **A phase 2 randomised study of ramucirumab (IMC-1121B) with or without dacarbazine in patients with metastatic melanoma**. *European journal of cancer (Oxford, England : 1990)* 2014, **50**(12):2099-2107.
10. [408]. Dronca RS, Allred JB, Perez DG, Nevala WK, Lieser EA, Thompson M, Maples WJ, Creagan ET, Pockaj BA, Kaur JS *et al*: **Phase II study of temozolomide (TMZ) and everolimus (RAD001) therapy for metastatic melanoma: a North Central Cancer Treatment Group study, N0675**. *American journal of clinical oncology* 2014, **37**(4):369-376.
11. [409]. Dummer R, Schadendorf D, Ascierto PA, Arance A, Dutriaux C, Di Giacomo AM, Rutkowski P, Del Vecchio M, Gutzmer R, Mandala M *et al*: **Binimetinib versus dacarbazine in patients with advanced NRAS-mutant melanoma (NEMO): a multicentre, open-label, randomised, phase 3 trial**. *The Lancet Oncology* 2017, **18**(4):435-445.
12. [410]. Eisen T, Marais R, Affolter A, Lorigan P, Robert C, Corrie P, Ottensmeier C, Chevreau C, Chao D, Nathan PD *et al*: **Sorafenib and dacarbazine as first-line therapy for advanced melanoma: phase I and open-label phase II studies**. *British journal of cancer* 2011, **105**(3):353-359.
13. [411]. Gronchi A, Ferrari S, Quagliuolo V, Broto JM, Pousa AL, Grignani G, Basso U, Blay JY, Tendero O, Beveridge RD *et al*: **Histotype-tailored neoadjuvant chemotherapy versus standard chemotherapy in patients with high-risk soft-tissue sarcomas (ISG-STS 1001): an international, open-label, randomised, controlled, phase 3, multicentre trial**. *The Lancet Oncology* 2017, **18**(6):812-822.
14. [412]. Hofmann MA, Hauschild A, Mohr P, Garbe C, Weichenthal M, Trefzer U, Drecoll U, Tilgen W, Schadendorf D, Kaatz M *et al*: **Prospective evaluation of supportive care with or without CVD chemotherapy as a second-line treatment in advanced melanoma by patient's choice: a multicentre Dermatologic Cooperative Oncology Group trial**. *Melanoma research* 2011, **21**(6):516-523.
15. [413]. Hong DS, Kurzrock R, Falchook GS, Andresen C, Kwak J, Ren M, Xu L, George GC, Kim KB, Nguyen LM *et al*: **Phase 1b study of lenvatinib (E7080) in combination with temozolomide for treatment of advanced melanoma**. *Oncotarget* 2015, **6**(40):43127-43134.
16. [414]. Jiang BS, Speicher PJ, Thomas S, Mosca PJ, Abernethy AP, Tyler DS: **Quality of life after isolated limb infusion for in-transit melanoma of the extremity**. *Annals of surgical oncology* 2015, **22**(5):1694-1700.
17. [415]. Jiang G, Li RH, Sun C, Jia HY, Lei TC, Liu YQ: **Efficacy and safety between temozolomide alone and temozolomide-based double therapy for malignant melanoma: a meta-analysis**. *Tumour biology : the journal of the International Society for Oncodevelopmental Biology and Medicine* 2014, **35**(1):315-322.
18. [416]. Jiang G, Li RH, Sun C, Liu YQ, Zheng JN: **Dacarbazine combined targeted therapy versus dacarbazine alone in patients with malignant melanoma: a meta-analysis**. *PloS one* 2014, **9**(12):e111920.
19. [417]. Khan OA, Gore M, Lorigan P, Stone J, Greystoke A, Burke W, Carmichael J, Watson AJ, McGown G, Thorncroft M *et al*: **A phase I study of the safety and tolerability of olaparib (AZD2281, KU0059436) and dacarbazine in patients with advanced solid tumours**. *British journal of cancer* 2011, **104**(5):750-755.
20. [418]. Kottschade LA, Suman VJ, Perez DG, McWilliams RR, Kaur JS, Amatruda TT, 3rd, Geoffroy FJ, Gross HM, Cohen PA, Jaslowski AJ *et al*: **A randomized phase 2 study of temozolomide and bevacizumab or nab-paclitaxel, carboplatin, and bevacizumab in patients with unresectable stage IV melanoma : a North Central Cancer Treatment Group study, N0775**. *Cancer* 2013, **119**(3):586-592.
21. [419]. Kyte JA, Gaudernack G, Dueland S, Trachsel S, Julsrud L, Aamdal S: **Telomerase peptide vaccination combined with temozolomide: a clinical trial in stage IV melanoma patients**. *Clinical cancer research : an official journal of the American Association for Cancer Research* 2011, **17**(13):4568-4580.
22. [420]. Lee CK, Jung M, Choi HJ, Kim HR, Kim HS, Roh MR, Ahn JB, Chung HC, Heo SJ, Rha SY *et al*: **Results of a Phase II Study to Evaluate the Efficacy of Docetaxel and Carboplatin in Metastatic Malignant Melanoma Patients Who Failed First-Line Therapy Containing Dacarbazine**. *Cancer research and treatment : official journal of Korean Cancer Association* 2015, **47**(4):781-789.
23. [421]. Middleton MR, Friedlander P, Hamid O, Daud A, Plummer R, Falotico N, Chyla B, Jiang F, McKeegan E, Mostafa NM *et al*: **Randomized phase II study evaluating veliparib (ABT-888) with temozolomide in patients with metastatic melanoma**. *Annals of oncology : official journal of the European Society for Medical Oncology* 2015, **26**(10):2173-2179.
24. [422]. Movva S, Verschraegen CF, Rabinowitz I, Mangalik A, Parks V, Lee FC: **Phase I dose finding study of carboplatin, paclitaxel, and temozolomide in advanced solid tumors**. *Melanoma research* 2011, **21**(1):76-79.
25. [423]. O'Day S, Pavlick A, Loquai C, Lawson D, Gutzmer R, Richards J, Schadendorf D, Thompson JA, Gonzalez R, Trefzer U *et al*: **A randomised, phase II study of intetumumab, an anti-alphav-integrin mAb, alone and with dacarbazine in stage IV melanoma**. *British journal of cancer* 2011, **105**(3):346-352.
26. [424]. Ott PA, Chang J, Madden K, Kannan R, Muren C, Escano C, Cheng X, Shao Y, Mendoza S, Gandhi A *et al*: **Oblimersen in combination with temozolomide and albumin-bound paclitaxel in patients with advanced melanoma: a phase I trial**. *Cancer chemotherapy and pharmacology* 2013, **71**(1):183-191.
27. [425]. Patel PM, Suciu S, Mortier L, Kruit WH, Robert C, Schadendorf D, Trefzer U, Punt CJ, Dummer R, Davidson N *et al*: **Extended schedule, escalated dose temozolomide versus dacarbazine in stage IV melanoma: final results of a randomised phase III study (EORTC 18032)**. *European journal of cancer (Oxford, England : 1990)* 2011, **47**(10):1476-1483.
28. [426]. Plummer R, Lorigan P, Brown E, Zaucha R, Moiseyenko V, Demidov L, Soriano V, Chmielowska E, Andres R, Kudryavtseva G *et al*: **Phase I-II study of plitidepsin and dacarbazine as first-line therapy for advanced melanoma**. *British journal of cancer* 2013, **109**(6):1451-1459.
29. [427]. Plummer R, Lorigan P, Steven N, Scott L, Middleton MR, Wilson RH, Mulligan E, Curtin N, Wang D, Dewji R *et al*: **A phase II study of the potent PARP inhibitor, Rucaparib (PF-01367338, AG014699), with temozolomide in patients with metastatic melanoma demonstrating evidence of chemopotentiation**. *Cancer chemotherapy and pharmacology* 2013, **71**(5):1191-1199.
30. [428]. Plummer R, Stephens P, Aissat-Daudigny L, Cambois A, Moachon G, Brown PD, Campone M: **Phase 1 dose-escalation study of the PARP inhibitor CEP-9722 as monotherapy or in combination with temozolomide in patients with solid tumors**. *Cancer chemotherapy and pharmacology* 2014, **74**(2):257-265.
31. [429]. Poklepovic A, Youssefian LE, Winning M, Birdsell CA, Crosby NA, Ramakrishnan V, Ernstoff MS, Roberts JD: **Phase I trial of bortezomib and dacarbazine in melanoma and soft tissue sarcoma**. *Investigational new drugs* 2013, **31**(4):937-942.
32. [430]. Quinn C, Ma Q, Kudlac A, Palmer S, Barber B, Zhao Z: **Relative Efficacy of Granulocyte-Macrophage Colony-Stimulating Factor, Dacarbazine, and Glycoprotein 100 in Metastatic Melanoma: An Indirect Treatment Comparison**. *Advances in therapy* 2017, **34**(2):495-512.
33. [431]. Robinson DW, Jr., Cormier JN, Zhao N, Uhlar CM, Revicki DA, Cella D: **Health-related quality of life among patients with metastatic melanoma: results from an international phase 2 multicenter study**. *Melanoma research* 2012, **22**(1):54-62.
34. [432]. Segura PP, Gil M, Balana C, Chacon I, Langa JM, Martin M, Bruna J: **Phase II trial of temozolomide for leptomeningeal metastases in patients with solid tumors**. *Journal of neuro-oncology* 2012, **109**(1):137-142.
35. [433]. Tawbi HA, Beumer JH, Tarhini AA, Moschos S, Buch SC, Egorin MJ, Lin Y, Christner S, Kirkwood JM: **Safety and efficacy of decitabine in combination with temozolomide in metastatic melanoma: a phase I/II study and pharmacokinetic analysis**. *Annals of oncology : official journal of the European Society for Medical Oncology* 2013, **24**(4):1112-1119.
36. [434]. Tawbi HA, Villaruz L, Tarhini A, Moschos S, Sulecki M, Viverette F, Shipe-Spotloe J, Radkowski R, Kirkwood JM: **Inhibition of DNA repair with MGMT pseudosubstrates: phase I study of lomeguatrib in combination with dacarbazine in patients with advanced melanoma and other solid tumours**. *British journal of cancer* 2011, **105**(6):773-777.
37. [435]. Teimouri F, Nikfar S, Abdollahi M: **Efficacy and side effects of dacarbazine in comparison with temozolomide in the treatment of malignant melanoma: a meta-analysis consisting of 1314 patients**. *Melanoma research* 2013, **23**(5):381-389.
38. [436]. Van Nuffel AM, Benteyn D, Wilgenhof S, Corthals J, Heirman C, Neyns B, Thielemans K, Bonehill A: **Intravenous and intradermal TriMix-dendritic cell therapy results in a broad T-cell response and durable tumor response in a chemorefractory stage IV-M1c melanoma patient**. *Cancer immunology, immunotherapy : CII* 2012, **61**(7):1033-1043.
39. [437]. Xia C, Leon-Ferre R, Laux D, Deutsch J, Smith BJ, Frees M, Milhem M: **Treatment of resistant metastatic melanoma using sequential epigenetic therapy (decitabine and panobinostat) combined with chemotherapy (temozolomide)**. *Cancer chemotherapy and pharmacology* 2014, **74**(4):691-697.

# TOPIC: Melanoma and Temozolomide

No unique search results

# TOPIC: Melanoma and Pembrolizumab

1. [438]. Abdel-Rahman O: **Evaluation of efficacy and safety of different pembrolizumab dose/schedules in treatment of non-small-cell lung cancer and melanoma: a systematic review**. *Immunotherapy* 2016, **8**(12):1383-1391.
2. [439]. Abdel-Rahman O: **PD-L1 expression and outcome of advanced melanoma patients treated with anti-PD-1/PD-L1 agents: a meta-analysis**. *Immunotherapy* 2016, **8**(9):1081-1089.
3. [440]. Bagcchi S: **Pembrolizumab for treatment of refractory melanoma**. *The Lancet Oncology* 2014, **15**(10):e419.
4. [441]. Carbognin L, Pilotto S, Milella M, Vaccaro V, Brunelli M, Calio A, Cuppone F, Sperduti I, Giannarelli D, Chilosi M *et al*: **Differential Activity of Nivolumab, Pembrolizumab and MPDL3280A according to the Tumor Expression of Programmed Death-Ligand-1 (PD-L1): Sensitivity Analysis of Trials in Melanoma, Lung and Genitourinary Cancers**. *PloS one* 2015, **10**(6):e0130142.
5. [442]. Daud AI, Wolchok JD, Robert C, Hwu WJ, Weber JS, Ribas A, Hodi FS, Joshua AM, Kefford R, Hersey P *et al*: **Programmed Death-Ligand 1 Expression and Response to the Anti-Programmed Death 1 Antibody Pembrolizumab in Melanoma**. *Journal of clinical oncology : official journal of the American Society of Clinical Oncology* 2016, **34**(34):4102-4109.
6. [443]. Gargett T, Yu W, Dotti G, Yvon ES, Christo SN, Hayball JD, Lewis ID, Brenner MK, Brown MP: **GD2-specific CAR T Cells Undergo Potent Activation and Deletion Following Antigen Encounter but can be Protected From Activation-induced Cell Death by PD-1 Blockade**. *Molecular therapy : the journal of the American Society of Gene Therapy* 2016, **24**(6):1135-1149.
7. [444]. Goldberg SB, Gettinger SN, Mahajan A, Chiang AC, Herbst RS, Sznol M, Tsiouris AJ, Cohen J, Vortmeyer A, Jilaveanu L *et al*: **Pembrolizumab for patients with melanoma or non-small-cell lung cancer and untreated brain metastases: early analysis of a non-randomised, open-label, phase 2 trial**. *The Lancet Oncology* 2016, **17**(7):976-983.
8. [445]. Guan X, Wang H, Ma F, Qian H, Yi Z, Xu B: **The Efficacy and Safety of Programmed Cell Death 1 and Programmed Cell Death 1 Ligand Inhibitors for Advanced Melanoma: A Meta-Analysis of Clinical Trials Following the PRISMA Guidelines**. *Medicine* 2016, **95**(11):e3134.
9. [446]. Hodi FS, Hwu WJ, Kefford R, Weber JS, Daud A, Hamid O, Patnaik A, Ribas A, Robert C, Gangadhar TC *et al*: **Evaluation of Immune-Related Response Criteria and RECIST v1.1 in Patients With Advanced Melanoma Treated With Pembrolizumab**. *Journal of clinical oncology : official journal of the American Society of Clinical Oncology* 2016, **34**(13):1510-1517.
10. [447]. Hua C, Boussemart L, Mateus C, Routier E, Boutros C, Cazenave H, Viollet R, Thomas M, Roy S, Benannoune N *et al*: **Association of Vitiligo With Tumor Response in Patients With Metastatic Melanoma Treated With Pembrolizumab**. *JAMA dermatology* 2016, **152**(1):45-51.
11. [448]. Huang AC, Postow MA, Orlowski RJ, Mick R, Bengsch B, Manne S, Xu W, Harmon S, Giles JR, Wenz B *et al*: **T-cell invigoration to tumour burden ratio associated with anti-PD-1 response**. *Nature* 2017, **545**(7652):60-65.
12. [449]. Lin Z, Chen X, Li Z, Luo Y, Fang Z, Xu B, Han M: **PD-1 Antibody Monotherapy for Malignant Melanoma: A Systematic Review and Meta-Analysis**. *PloS one* 2016, **11**(8):e0160485.
13. [450]. Nishino M, Giobbie-Hurder A, Hatabu H, Ramaiya NH, Hodi FS: **Incidence of Programmed Cell Death 1 Inhibitor-Related Pneumonitis in Patients With Advanced Cancer: A Systematic Review and Meta-analysis**. *JAMA oncology* 2016, **2**(12):1607-1616.
14. [451]. Patnaik A, Kang SP, Rasco D, Papadopoulos KP, Elassaiss-Schaap J, Beeram M, Drengler R, Chen C, Smith L, Espino G *et al*: **Phase I Study of Pembrolizumab (MK-3475; Anti-PD-1 Monoclonal Antibody) in Patients with Advanced Solid Tumors**. *Clinical cancer research : an official journal of the American Association for Cancer Research* 2015, **21**(19):4286-4293.
15. [452]. Ribas A, Shin DS, Zaretsky J, Frederiksen J, Cornish A, Avramis E, Seja E, Kivork C, Siebert J, Kaplan-Lefko P *et al*: **PD-1 Blockade Expands Intratumoral Memory T Cells**. *Cancer immunology research* 2016, **4**(3):194-203.
16. [453]. Tumeh PC, Harview CL, Yearley JH, Shintaku IP, Taylor EJ, Robert L, Chmielowski B, Spasic M, Henry G, Ciobanu V *et al*: **PD-1 blockade induces responses by inhibiting adaptive immune resistance**. *Nature* 2014, **515**(7528):568-571.
17. [454]. Yamazaki N, Takenouchi T, Fujimoto M, Ihn H, Uchi H, Inozume T, Kiyohara Y, Uhara H, Nakagawa K, Furukawa H *et al*: **Phase 1b study of pembrolizumab (MK-3475; anti-PD-1 monoclonal antibody) in Japanese patients with advanced melanoma (KEYNOTE-041)**. *Cancer chemotherapy and pharmacology* 2017, **79**(4):651-660.

# TOPIC: Melanoma and Nivolumab

1. [455]. Brahmer JR, Drake CG, Wollner I, Powderly JD, Picus J, Sharfman WH, Stankevich E, Pons A, Salay TM, McMiller TL *et al*: **Phase I study of single-agent anti-programmed death-1 (MDX-1106) in refractory solid tumors: safety, clinical activity, pharmacodynamics, and immunologic correlates**. *Journal of clinical oncology : official journal of the American Society of Clinical Oncology* 2010, **28**(19):3167-3175.
2. [456]. Brahmer JR, Tykodi SS, Chow LQ, Hwu WJ, Topalian SL, Hwu P, Drake CG, Camacho LH, Kauh J, Odunsi K *et al*: **Safety and activity of anti-PD-L1 antibody in patients with advanced cancer**. *The New England journal of medicine* 2012, **366**(26):2455-2465.
3. [457]. Freeman-Keller M, Kim Y, Cronin H, Richards A, Gibney G, Weber JS: **Nivolumab in Resected and Unresectable Metastatic Melanoma: Characteristics of Immune-Related Adverse Events and Association with Outcomes**. *Clinical cancer research : an official journal of the American Association for Cancer Research* 2016, **22**(4):886-894.
4. [458]. Gibney GT, Kudchadkar RR, DeConti RC, Thebeau MS, Czupryn MP, Tetteh L, Eysmans C, Richards A, Schell MJ, Fisher KJ *et al*: **Safety, correlative markers, and clinical results of adjuvant nivolumab in combination with vaccine in resected high-risk metastatic melanoma**. *Clinical cancer research : an official journal of the American Association for Cancer Research* 2015, **21**(4):712-720.
5. [459]. Kazandjian D, Khozin S, Blumenthal G, Zhang L, Tang S, Libeg M, Kluetz P, Sridhara R, Keegan P, Pazdur R: **Benefit-Risk Summary of Nivolumab for Patients With Metastatic Squamous Cell Lung Cancer After Platinum-Based Chemotherapy: A Report From the US Food and Drug Administration**. *JAMA oncology* 2016, **2**(1):118-122.
6. [460]. Krcik EM: **Radiation Therapy Plus Anti-Programmed Death Ligand 1 Immunotherapy: A Review on Overall Survival**. *Radiologic technology* 2016, **88**(1):123-128.
7. [461]. Lipson EJ, Sharfman WH, Drake CG, Wollner I, Taube JM, Anders RA, Xu H, Yao S, Pons A, Chen L *et al*: **Durable cancer regression off-treatment and effective reinduction therapy with an anti-PD-1 antibody**. *Clinical cancer research : an official journal of the American Association for Cancer Research* 2013, **19**(2):462-468.
8. [462]. Sharma P, Callahan MK, Bono P, Kim J, Spiliopoulou P, Calvo E, Pillai RN, Ott PA, de Braud F, Morse M *et al*: **Nivolumab monotherapy in recurrent metastatic urothelial carcinoma (CheckMate 032): a multicentre, open-label, two-stage, multi-arm, phase 1/2 trial**. *The Lancet Oncology* 2016, **17**(11):1590-1598.
9. [463]. Topalian SL, Hodi FS, Brahmer JR, Gettinger SN, Smith DC, McDermott DF, Powderly JD, Carvajal RD, Sosman JA, Atkins MB *et al*: **Safety, activity, and immune correlates of anti-PD-1 antibody in cancer**. *The New England journal of medicine* 2012, **366**(26):2443-2454.
10. [464]. Topalian SL, Sznol M, McDermott DF, Kluger HM, Carvajal RD, Sharfman WH, Brahmer JR, Lawrence DP, Atkins MB, Powderly JD *et al*: **Survival, durable tumor remission, and long-term safety in patients with advanced melanoma receiving nivolumab**. *Journal of clinical oncology : official journal of the American Society of Clinical Oncology* 2014, **32**(10):1020-1030.

# TOPIC: Melanoma and Talimogene Laherparepvec

1. [465]. Andtbacka RH, Kaufman HL, Collichio F, Amatruda T, Senzer N, Chesney J, Delman KA, Spitler LE, Puzanov I, Agarwala SS *et al*: **Talimogene Laherparepvec Improves Durable Response Rate in Patients With Advanced Melanoma**. *Journal of clinical oncology : official journal of the American Society of Clinical Oncology* 2015, **33**(25):2780-2788.

# TOPIC: Melanoma and PD-1

1. [466]. Gandini S, Massi D, Mandala M: **PD-L1 expression in cancer patients receiving anti PD-1/PD-L1 antibodies: A systematic review and meta-analysis**. *Critical reviews in oncology/hematology* 2016, **100**:88-98.
2. [467]. Goldberg JM, Fisher DE, Demetri GD, Neuberg D, Allsop SA, Fonseca C, Nakazaki Y, Nemer D, Raut CP, George S *et al*: **Biologic Activity of Autologous, Granulocyte-Macrophage Colony-Stimulating Factor Secreting Alveolar Soft-Part Sarcoma and Clear Cell Sarcoma Vaccines**. *Clinical cancer research : an official journal of the American Association for Cancer Research* 2015, **21**(14):3178-3186.
3. [468]. Gros A, Robbins PF, Yao X, Li YF, Turcotte S, Tran E, Wunderlich JR, Mixon A, Farid S, Dudley ME *et al*: **PD-1 identifies the patient-specific CD8(+) tumor-reactive repertoire infiltrating human tumors**. *The Journal of clinical investigation* 2014, **124**(5):2246-2259.
4. [469]. Reuben JM, Lee BN, Li C, Gomez-Navarro J, Bozon VA, Parker CA, Hernandez IM, Gutierrez C, Lopez-Berestein G, Camacho LH: **Biologic and immunomodulatory events after CTLA-4 blockade with ticilimumab in patients with advanced malignant melanoma**. *Cancer* 2006, **106**(11):2437-2444.
5. [470]. Thibult ML, Rivals JP, Mamessier E, Gertner-Dardenne J, Pastor S, Speiser DE, Derre L, Olive D: **CpG-ODN-induced sustained expression of BTLA mediating selective inhibition of human B cells**. *Journal of molecular medicine (Berlin, Germany)* 2013, **91**(2):195-205.

# TOPIC: Melanoma and PD-L1

1. [471]. Levy A, Massard C, Soria JC, Deutsch E: **Concurrent irradiation with the anti-programmed cell death ligand-1 immune checkpoint blocker durvalumab: Single centre subset analysis from a phase 1/2 trial**. *European journal of cancer (Oxford, England : 1990)* 2016, **68**:156-162.
2. [472]. Wang Q, Liu F, Liu L: **Prognostic significance of PD-L1 in solid tumor: An updated meta-analysis**. *Medicine* 2017, **96**(18):e6369.

# TOPIC: Melanoma and Combination Immunotherapy

1. [473]. Adams S, O'Neill DW, Nonaka D, Hardin E, Chiriboga L, Siu K, Cruz CM, Angiulli A, Angiulli F, Ritter E *et al*: **Immunization of malignant melanoma patients with full-length NY-ESO-1 protein using TLR7 agonist imiquimod as vaccine adjuvant**. *Journal of immunology (Baltimore, Md : 1950)* 2008, **181**(1):776-784.
2. [474]. Albertini MR, Hank JA, Schiller JH, Khorsand M, Borchert AA, Gan J, Bechhofer R, Storer B, Reisfeld RA, Sondel PM: **Phase IB trial of chimeric antidisialoganglioside antibody plus interleukin 2 for melanoma patients**. *Clinical cancer research : an official journal of the American Association for Cancer Research* 1997, **3**(8):1277-1288.
3. [475]. Anderson CM, Buzaid AC, Sussman J, Lee JJ, Ali-Osman F, Braunschweiger PG, Plager C, Bedikian A, Papadopoulos N, Eton O *et al*: **Nitric oxide and neopterin levels and clinical response in stage III melanoma patients receiving concurrent biochemotherapy**. *Melanoma research* 1998, **8**(2):149-155.
4. [476]. Antoine EC, Rixe O, Vuillemin E, Benhammouda A, Borel C, Ghironzi GC, Mularoni E, Franks C, Auclerc G, Soubrane C *et al*: **A phase II study of tamoxifen combined with cisplatin-interleukin 2 and alpha-interferon in metastatic melanoma**. *American journal of clinical oncology* 1995, **18**(5):421-424.
5. [477]. Appay V, Voelter V, Rufer N, Reynard S, Jandus C, Gasparini D, Lienard D, Speiser DE, Schneider P, Cerottini JC *et al*: **Combination of transient lymphodepletion with busulfan and fludarabine and peptide vaccination in a phase I clinical trial for patients with advanced melanoma**. *Journal of immunotherapy (Hagerstown, Md : 1997)* 2007, **30**(2):240-250.
6. [478]. Asemissen AM, Scheibenbogen C, Letsch A, Hellstrand K, Thoren F, Gehlsen K, Schmittel A, Thiel E, Keilholz U: **Addition of histamine to interleukin 2 treatment augments type 1 T-cell responses in patients with melanoma in vivo: immunologic results from a randomized clinical trial of interleukin 2 with or without histamine (MP 104)**. *Clinical cancer research : an official journal of the American Association for Cancer Research* 2005, **11**(1):290-297.
7. [479]. Atkins MB, O'Boyle KR, Sosman JA, Weiss GR, Margolin KA, Ernest ML, Kappler K, Mier JW, Sparano JA, Fisher RI *et al*: **Multiinstitutional phase II trial of intensive combination chemoimmunotherapy for metastatic melanoma**. *Journal of clinical oncology : official journal of the American Society of Clinical Oncology* 1994, **12**(8):1553-1560.
8. [480]. Atzpodien J, Lopez Hanninen E, Kirchner H, Franzke A, Korfer A, Volkenandt M, Duensing S, Schomburg A, Chaitchik S, Poliwoda H: **Chemoimmunotherapy of advanced malignant melanoma: sequential administration of subcutaneous interleukin-2 and interferon-alpha after intravenous dacarbazine and carboplatin or intravenous dacarbazine, cisplatin, carmustine and tamoxifen**. *European journal of cancer (Oxford, England : 1990)* 1995, **31a**(6):876-881.
9. [481]. Atzpodien J, Neuber K, Kamanabrou D, Fluck M, Brocker EB, Neumann C, Runger TM, Schuler G, von den Driesch P, Muller I *et al*: **Combination chemotherapy with or without s.c. IL-2 and IFN-alpha: results of a prospectively randomized trial of the Cooperative Advanced Malignant Melanoma Chemoimmunotherapy Group (ACIMM)**. *British journal of cancer* 2002, **86**(2):179-184.
10. [482]. Atzpodien J, Reitz M: **GM-CSF plus antigenic peptide vaccination in locally advanced melanoma patients**. *Cancer biotherapy & radiopharmaceuticals* 2007, **22**(4):551-555.
11. [483]. Bajetta E, Del Vecchio M, Vitali M, Martinetti A, Ferrari L, Queirolo P, Sertoli MR, Cainelli T, Cellerino R, Cascinelli N: **A feasibility study using polychemotherapy (cisplatin + vindesine + dacarbazine) plus interferon-alpha or monochemotherapy with dacarbazine plus interferon-alpha in metastatic melanoma**. *Tumori* 2001, **87**(4):219-222.
12. [484]. Baldueva IA, Novik AV, Moiseenko VM, Nekhaeva TL, Danilova AB, Danilov AO, Protsenko SA, Petrova T, Uleiskaia GI, Shchekina LA *et al*: **[Phase II clinical trial of autologous dendritic cell vaccine with immunologic adjuvant in cutaneous melanoma patients]**. *Voprosy onkologii* 2012, **58**(2):212-221.
13. [485]. Banzet P, Dufourmental C: **[Present concepts of the treatment of malignant melanoma (author's transl)]**. *Acta chirurgica Belgica* 1975, **74**(4):395-405.
14. [486]. Barrio MM, de Motta PT, Kaplan J, von Euw EM, Bravo AI, Chacon RD, Mordoh J: **A phase I study of an allogeneic cell vaccine (VACCIMEL) with GM-CSF in melanoma patients**. *Journal of immunotherapy (Hagerstown, Md : 1997)* 2006, **29**(4):444-454.
15. [487]. Berkelhammer J, Mastrangelo MJ, Bellet RE, Berd D, Prehn RT: **Chemoimmunotherapy increases the lymphocyte reactivity of melanoma patients**. *European journal of cancer (Oxford, England : 1990)* 1979, **15**(2):197-204.
16. [488]. Betticher DC, Lee SM, Morris C, Clemons M, Thatcher N: **Dacarbazine and interferon-alpha 2a in advanced malignant melanoma: high response rate and prolongation of response duration occur in different patient subpopulations**. *Melanoma research* 1995, **5**(4):277-282.
17. [489]. Bins A, Mallo H, Sein J, van den Bogaard C, Nooijen W, Vyth-Dreese F, Nuijen B, de Gast GC, Haanen JB: **Phase I clinical study with multiple peptide vaccines in combination with tetanus toxoid and GM-CSF in advanced-stage HLA-A*0201-positive melanoma patients**. *Journal of immunotherapy (Hagerstown, Md : 1997)* 2007, **30**(2):234-239.
18. [490]. Carmo-Pereira J, Costa FO, Henriques E: **Cytotoxic chemotherapy of disseminated cutaneous malignant melanoma--a prospective and randomized clinical trial of procarbazine, vindesine and lomustine versus procarbazine, DTIC and lomustine**. *European journal of cancer & clinical oncology* 1986, **22**(12):1435-1439.
19. [491]. Castello G, Ruocco V, Satriano RA, Zarrilli D: **Role of interferons in the therapy of melanoma**. *Melanoma research* 1992, **1**(5-6):311-325.
20. [492]. Chang AE, Rosenberg SA: **Overview of interleukin-2 as an immunotherapeutic agent**. *Seminars in surgical oncology* 1989, **5**(6):385-390.
21. [493]. Chang JW, Hsieh JJ, Shen YC, Ho E, Chuang CK, Chen YR, Liao SK, Chen JS, Leong SP, Hou MM *et al*: **Immunotherapy with dendritic cells pulsed by autologous dactinomycin-induced melanoma apoptotic bodies for patients with malignant melanoma**. *Melanoma research* 2009, **19**(5):309-315.
22. [494]. Chapuis AG, Lee SM, Thompson JA, Roberts IM, Margolin KA, Bhatia S, Sloan HL, Lai I, Wagener F, Shibuya K *et al*: **Combined IL-21-primed polyclonal CTL plus CTLA4 blockade controls refractory metastatic melanoma in a patient**. *The Journal of experimental medicine* 2016, **213**(7):1133-1139.
23. [495]. Chen JL, Dawoodji A, Tarlton A, Gnjatic S, Tajar A, Karydis I, Browning J, Pratap S, Verfaille C, Venhaus RR *et al*: **NY-ESO-1 specific antibody and cellular responses in melanoma patients primed with NY-ESO-1 protein in ISCOMATRIX and boosted with recombinant NY-ESO-1 fowlpox virus**. *International journal of cancer* 2015, **136**(6):E590-601.
24. [496]. Chianese-Bullock KA, Irvin WP, Jr., Petroni GR, Murphy C, Smolkin M, Olson WC, Coleman E, Boerner SA, Nail CJ, Neese PY *et al*: **A multipeptide vaccine is safe and elicits T-cell responses in participants with advanced stage ovarian cancer**. *Journal of immunotherapy (Hagerstown, Md : 1997)* 2008, **31**(4):420-430.
25. [497]. Chianese-Bullock KA, Woodson EM, Tao H, Boerner SA, Smolkin M, Grosh WW, Neese PY, Merrill P, Petroni GR, Slingluff CL, Jr.: **Autoimmune toxicities associated with the administration of antitumor vaccines and low-dose interleukin-2**. *Journal of immunotherapy (Hagerstown, Md : 1997)* 2005, **28**(4):412-419.
26. [498]. Choi BS, Sondel PM, Hank JA, Schalch H, Gan J, King DM, Kendra K, Mahvi D, Lee LY, Kim K *et al*: **Phase I trial of combined treatment with ch14.18 and R24 monoclonal antibodies and interleukin-2 for patients with melanoma or sarcoma**. *Cancer immunology, immunotherapy : CII* 2006, **55**(7):761-774.
27. [499]. Clark JW, Longo DL: **Adoptive therapies: quo vadis?** *Pathology and immunopathology research* 1988, **7**(6):442-458.
28. [500]. Costanzi JJ, Fletcher WS, Balcerzak SP, Taylor S, Eyre HJ, O'Bryan RM, Al-Sarraf M, Frank J: **Combination chemotherapy plus levamisole in the treatment of disseminated malignant melanoma. A Southwest Oncology Group study**. *Cancer* 1984, **53**(4):833-836.
29. [501]. Creagan ET, Rowland KM, Jr., Suman VJ, Kardinal CG, Marschke RF, Jr., Marks RS, Maples WJ: **Phase II study of combined levamisole with recombinant interleukin-2 in patients with advanced malignant melanoma**. *American journal of clinical oncology* 1997, **20**(5):490-492.
30. [502]. Danielli R, Patuzzo R, Di Giacomo AM, Gallino G, Maurichi A, Di Florio A, Cutaia O, Lazzeri A, Fazio C, Miracco C *et al*: **Intralesional administration of L19-IL2/L19-TNF in stage III or stage IVM1a melanoma patients: results of a phase II study**. *Cancer immunology, immunotherapy : CII* 2015, **64**(8):999-1009.
31. [503]. Dorval T, Negrier S, Chevreau C, Avril MF, Baume D, Cupissol D, Oskam R, de Peuter R, Vinke J, Herrera A *et al*: **Randomized trial of treatment with cisplatin and interleukin-2 either alone or in combination with interferon-alpha-2a in patients with metastatic melanoma: a Federation Nationale des Centres de Lutte Contre le Cancer Multicenter, parallel study**. *Cancer* 1999, **85**(5):1060-1066.
32. [504]. Dudley ME, Wunderlich JR, Yang JC, Hwu P, Schwartzentruber DJ, Topalian SL, Sherry RM, Marincola FM, Leitman SF, Seipp CA *et al*: **A phase I study of nonmyeloablative chemotherapy and adoptive transfer of autologous tumor antigen-specific T lymphocytes in patients with metastatic melanoma**. *Journal of immunotherapy (Hagerstown, Md : 1997)* 2002, **25**(3):243-251.
33. [505]. Dudley ME, Wunderlich JR, Yang JC, Sherry RM, Topalian SL, Restifo NP, Royal RE, Kammula U, White DE, Mavroukakis SA *et al*: **Adoptive cell transfer therapy following non-myeloablative but lymphodepleting chemotherapy for the treatment of patients with refractory metastatic melanoma**. *Journal of clinical oncology : official journal of the American Society of Clinical Oncology* 2005, **23**(10):2346-2357.
34. [506]. Elias EG, Zapas JL, Beam SL, Brown SD: **GM-CSF and IL-2 combination as adjuvant therapy in cutaneous melanoma: early results of a phase II clinical trial**. *Oncology (Williston Park, NY)* 2005, **19**(4 Suppl 2):15-18.
35. [507]. Escobar A, Lopez M, Serrano A, Ramirez M, Perez C, Aguirre A, Gonzalez R, Alfaro J, Larrondo M, Fodor M *et al*: **Dendritic cell immunizations alone or combined with low doses of interleukin-2 induce specific immune responses in melanoma patients**. *Clinical and experimental immunology* 2005, **142**(3):555-568.
36. [508]. Filipazzi P, Pilla L, Mariani L, Patuzzo R, Castelli C, Camisaschi C, Maurichi A, Cova A, Rigamonti G, Giardino F *et al*: **Limited induction of tumor cross-reactive T cells without a measurable clinical benefit in early melanoma patients vaccinated with human leukocyte antigen class I-modified peptides**. *Clinical cancer research : an official journal of the American Association for Cancer Research* 2012, **18**(23):6485-6496.
37. [509]. Finck S, Lampert R, Brandt L: **[Hyperdynamic hemodynamics following high-dose interleukin 2-interferon alpha therapy in patients with metastatic renal cell carcinoma. Immunotherapy as a clinical sepsis model?]**. *Der Anaesthesist* 1996, **45**(12):1171-1178.
38. [510]. Flaherty LE: **Rationale for intergroup trial E-3695 comparing concurrent biochemotherapy with cisplatin, vinblastine, and DTIC alone in patients with metastatic melanoma**. *The cancer journal from Scientific American* 2000, **6 Suppl 1**:S15-20.
39. [511]. Fourcade J, Kudela P, Andrade Filho PA, Janjic B, Land SR, Sander C, Krieg A, Donnenberg A, Shen H, Kirkwood JM *et al*: **Immunization with analog peptide in combination with CpG and montanide expands tumor antigen-specific CD8+ T cells in melanoma patients**. *Journal of immunotherapy (Hagerstown, Md : 1997)* 2008, **31**(8):781-791.
40. [512]. Frost GA, Halliday GM, Damian DL: **Photodynamic therapy-induced immunosuppression in humans is prevented by reducing the rate of light delivery**. *The Journal of investigative dermatology* 2011, **131**(4):962-968.
41. [513]. Green DS, Dalgleish AG, Belonwu N, Fischer MD, Bodman-Smith MD: **Topical imiquimod and intralesional interleukin-2 increase activated lymphocytes and restore the Th1/Th2 balance in patients with metastatic melanoma**. *The British journal of dermatology* 2008, **159**(3):606-614.
42. [514]. Hainsworth JD, Infante JR, Spigel DR, Peyton JD, Thompson DS, Lane CM, Clark BL, Rubin MS, Trent DF, Burris HA, 3rd: **Bevacizumab and everolimus in the treatment of patients with metastatic melanoma: a phase 2 trial of the Sarah Cannon Oncology Research Consortium**. *Cancer* 2010, **116**(17):4122-4129.
43. [515]. Haluska FG, Multani PS: **Melanoma**. *Cancer chemotherapy and biological response modifiers* 1999, **18**:470-488.
44. [516]. Hodi FS, Butler M, Oble DA, Seiden MV, Haluska FG, Kruse A, Macrae S, Nelson M, Canning C, Lowy I *et al*: **Immunologic and clinical effects of antibody blockade of cytotoxic T lymphocyte-associated antigen 4 in previously vaccinated cancer patients**. *Proceedings of the National Academy of Sciences of the United States of America* 2008, **105**(8):3005-3010.
45. [517]. Homes EC, Morton DL, Eilber FR, Golub SE, Sulit HL: **Immunotherapy in malignant melanomaa**. *National Cancer Institute monograph* 1976, **44**:85-86.
46. [518]. Huncharek M, Caubet JF, McGarry R: **Single-agent DTIC versus combination chemotherapy with or without immunotherapy in metastatic melanoma: a meta-analysis of 3273 patients from 20 randomized trials**. *Melanoma research* 2001, **11**(1):75-81.
47. [519]. Isacson R, Kedar E, Barak V, Gazit Z, Yurim O, Kalichman I, Ben-Bassat H, Biran S, Schlesinger M, Franks CR *et al*: **Chemo-immunotherapy in patients with metastatic melanoma using sequential treatment with dacarbazine and recombinant human interleukin-2: evaluation of hematologic and immunologic parameters and correlation with clinical response**. *Immunology letters* 1992, **33**(2):127-134.
48. [520]. Jacobs JF, Punt CJ, Lesterhuis WJ, Sutmuller RP, Brouwer HM, Scharenborg NM, Klasen IS, Hilbrands LB, Figdor CG, de Vries IJ *et al*: **Dendritic cell vaccination in combination with anti-CD25 monoclonal antibody treatment: a phase I/II study in metastatic melanoma patients**. *Clinical cancer research : an official journal of the American Association for Cancer Research* 2010, **16**(20):5067-5078.
49. [521]. Jacquillat C, Banzet P, Maral J: **Clinical trials of chemotherapy and chemoimmunotherapy in primary malignant melanoma**. *Recent results in cancer research Fortschritte der Krebsforschung Progres dans les recherches sur le cancer* 1982, **80**:254-258.
50. [522]. Jahn B, Brieger J, Fenchel K, Mitrou PS, Bergmann L: **In vivo regulation of transforming growth factor beta 1 transcription by immunotherapy: interleukin-2 impairs interferon-alpha-stimulated increase in steady-state mRNA levels of transforming growth factor beta 1**. *Cancer immunology, immunotherapy : CII* 1994, **38**(5):304-310.
51. [523]. Jansen RL, Slingerland R, Goey SH, Franks CR, Bolhuis RL, Stoter G: **Interleukin-2 and interferon-alpha in the treatment of patients with advanced non-small-cell lung cancer**. *Journal of immunotherapy : official journal of the Society for Biological Therapy* 1992, **12**(1):70-73.
52. [524]. Jimbow K, Yanbe H, Nishio C: **Chemoimmunotherapy for melanoma with DTIC, ACNU, VCR and OK432: evaluation of new combinations for survival rates, side effects and cellular immunity**. *The Journal of dermatology* 1981, **8**(4):259-266.
53. [525]. Jin S, Zhang Q, Kang X, Wang J, Sun W: **Malignant melanoma therapy by chemotherapy and autoimmunity induced by cytokine**. *Cancer biotherapy & radiopharmaceuticals* 2009, **24**(2):237-241.
54. [526]. Karakousis CP, Didolkar MS, Lopez R, Baffi R, Moore R, Holyoke ED: **Chemoimmunotherapy (DTIC and Corynebacterium parvum) as adjuvant treatment in malignant melanoma**. *Cancer treatment reports* 1979, **63**(11-12):1739-1743.
55. [527]. Keilholz U, Scheibenbogen C, Mohler T, Brossart P, Maclachlan D, Geueke AM, Jochim A, Hunstein W: **Addition of dacarbazine or cisplatin to interferon-alpha/interleukin-2 in metastatic melanoma: toxicity and immunological effects**. *Melanoma research* 1995, **5**(4):283-287.
56. [528]. Keilholz U, Stoter G, Punt CJ, Scheibenbogen C, Lejeune F, Eggermont AM: **Recombinant interleukin-2-based treatments for advanced melanoma: the experience of the European Organization for Research and Treatment of Cancer Melanoma Cooperative Group**. *The cancer journal from Scientific American* 1997, **3 Suppl 1**:S22-28.
57. [529]. Khayat D, Borel C, Tourani JM, Benhammouda A, Antoine E, Rixe O, Vuillemin E, Bazex PA, Thill L, Franks R *et al*: **Sequential chemoimmunotherapy with cisplatin, interleukin-2, and interferon alfa-2a for metastatic melanoma**. *Journal of clinical oncology : official journal of the American Society of Clinical Oncology* 1993, **11**(11):2173-2180.
58. [530]. Kirchner HH, Atzpodien J, Poliwoda H: **[Chemo-/immunotherapy in advanced malignant melanoma: carboplatin and DTIC or cisplatin, dtic, bcnu and tamoxifen followed by immunotherapy with interleukin 2 and interferon alpha-2a]**. *Medizinische Klinik (Munich, Germany : 1983)* 1996, **91 Suppl 3**:44-49.
59. [531]. Kirkwood JM, Lee S, Moschos SJ, Albertini MR, Michalak JC, Sander C, Whiteside T, Butterfield LH, Weiner L: **Immunogenicity and antitumor effects of vaccination with peptide vaccine+/-granulocyte-monocyte colony-stimulating factor and/or IFN-alpha2b in advanced metastatic melanoma: Eastern Cooperative Oncology Group Phase II Trial E1696**. *Clinical cancer research : an official journal of the American Association for Cancer Research* 2009, **15**(4):1443-1451.
60. [532]. Kruit WH, Suciu S, Dreno B, Mortier L, Robert C, Chiarion-Sileni V, Maio M, Testori A, Dorval T, Grob JJ *et al*: **Selection of immunostimulant AS15 for active immunization with MAGE-A3 protein: results of a randomized phase II study of the European Organisation for Research and Treatment of Cancer Melanoma Group in Metastatic Melanoma**. *Journal of clinical oncology : official journal of the American Society of Clinical Oncology* 2013, **31**(19):2413-2420.
61. [533]. Landi G, Polverelli M, Moscatelli G, Morelli R, Landi C, Fiscelli O, Erbazzi A: **Sentinel lymph node biopsy in patients with primary cutaneous melanoma: study of 455 cases**. *Journal of the European Academy of Dermatology and Venereology : JEADV* 2000, **14**(1):35-45.
62. [534]. Lee KH, Wang E, Nielsen MB, Wunderlich J, Migueles S, Connors M, Steinberg SM, Rosenberg SA, Marincola FM: **Increased vaccine-specific T cell frequency after peptide-based vaccination correlates with increased susceptibility to in vitro stimulation but does not lead to tumor regression**. *Journal of immunology (Baltimore, Md : 1950)* 1999, **163**(11):6292-6300.
63. [535]. Legat A, Maby-El Hajjami H, Baumgaertner P, Cagnon L, Abed Maillard S, Geldhof C, Iancu EM, Lebon L, Guillaume P, Dojcinovic D *et al*: **Vaccination with LAG-3Ig (IMP321) and Peptides Induces Specific CD4 and CD8 T-Cell Responses in Metastatic Melanoma Patients--Report of a Phase I/IIa Clinical Trial**. *Clinical cancer research : an official journal of the American Association for Cancer Research* 2016, **22**(6):1330-1340.
64. [536]. Legha SS, Ring S, Eton O, Bedikian A, Plager C, Papadopoulos N: **Development and results of biochemotherapy in metastatic melanoma: the University of Texas M.D. Anderson Cancer Center experience**. *The cancer journal from Scientific American* 1997, **3 Suppl 1**:S9-15.
65. [537]. Lindsey KR, Gritz L, Sherry R, Abati A, Fetsch PA, Goldfeder LC, Gonzales MI, Zinnack KA, Rogers-Freezer L, Haworth L *et al*: **Evaluation of prime/boost regimens using recombinant poxvirus/tyrosinase vaccines for the treatment of patients with metastatic melanoma**. *Clinical cancer research : an official journal of the American Association for Cancer Research* 2006, **12**(8):2526-2537.
66. [538]. Locke F, Clark JI, Gajewski TF: **A phase II study of oxaliplatin, docetaxel, and GM-CSF in patients with previously treated advanced melanoma**. *Cancer chemotherapy and pharmacology* 2010, **65**(3):509-514.
67. [539]. Mahalingam D, Fountzilas C, Moseley J, Noronha N, Tran H, Chakrabarty R, Selvaggi G, Coffey M, Thompson B, Sarantopoulos J: **A phase II study of REOLYSIN(R) (pelareorep) in combination with carboplatin and paclitaxel for patients with advanced malignant melanoma**. *Cancer chemotherapy and pharmacology* 2017, **79**(4):697-703.
68. [540]. Mainwaring PN, Atkinson H, Chang J, Moore J, Hancock BW, Guillou PJ, Oskam R, Gore ME: **Differential responses to chemoimmunotherapy in patients with metastatic malignant melanoma**. *European journal of cancer (Oxford, England : 1990)* 1997, **33**(9):1388-1392.
69. [541]. Maio M, Mackiewicz A, Testori A, Trefzer U, Ferraresi V, Jassem J, Garbe C, Lesimple T, Guillot B, Gascon P *et al*: **Large randomized study of thymosin alpha 1, interferon alfa, or both in combination with dacarbazine in patients with metastatic melanoma**. *Journal of clinical oncology : official journal of the American Society of Clinical Oncology* 2010, **28**(10):1780-1787.
70. [542]. Maker AV, Phan GQ, Attia P, Yang JC, Sherry RM, Topalian SL, Kammula US, Royal RE, Haworth LR, Levy C *et al*: **Tumor regression and autoimmunity in patients treated with cytotoxic T lymphocyte-associated antigen 4 blockade and interleukin 2: a phase I/II study**. *Annals of surgical oncology* 2005, **12**(12):1005-1016.
71. [543]. Marincola FM, White DE, Wise AP, Rosenberg SA: **Combination therapy with interferon alfa-2a and interleukin-2 for the treatment of metastatic cancer**. *Journal of clinical oncology : official journal of the American Society of Clinical Oncology* 1995, **13**(5):1110-1122.
72. [544]. Masucci GV, Mansson-Brahme E, Ragnarsson-Olding B, Nilsson B, Wagenius G, Hansson J: **Alternating chemo-immunotherapy with temozolomide and low-dose interleukin-2 in patients with metastatic melanoma**. *Melanoma research* 2006, **16**(4):357-363.
73. [545]. Meijer SL, Dols A, Urba WJ, Hu HM, Smith IJ, Vetto J, Wood W, Doran T, Chu Y, Sayaharuban P *et al*: **Adoptive cellular therapy with tumor vaccine draining lymph node lymphocytes after vaccination with HLA-B7/beta2-microglobulin gene-modified autologous tumor cells**. *Journal of immunotherapy (Hagerstown, Md : 1997)* 2002, **25**(4):359-372.
74. [546]. Merad M, Angevin E, Wolfers J, Flament C, Lorenzi I, Triebel F, Escudier B, Zitvogel L: **Generation of monocyte-derived dendritic cells from patients with renal cell cancer: modulation of their functional properties after therapy with biological response modifiers (IFN-alpha plus IL-2 and IL-12)**. *Journal of immunotherapy (Hagerstown, Md : 1997)* 2000, **23**(3):369-378.
75. [547]. Middleton M, Sarno M, Agarwala SS, Glaspy J, Laurent A, McMasters K, Naredi P, O'Day S, Whitman E, Danson S *et al*: **Pharmacokinetics of histamine dihydrochloride in healthy volunteers and cancer patients: implications for combined immunotherapy with interleukin-2**. *Journal of clinical pharmacology* 2002, **42**(7):774-781.
76. [548]. Morton DL, Foshag LJ, Nizze JA, Gupta RK, Famatiga E, Hoon DS, Irie RF: **Active specific immunotherapy in malignant melanoma**. *Seminars in surgical oncology* 1989, **5**(6):420-425.
77. [549]. Morton DL, Goodnight JE, Jr.: **Clinical trials of immunotherapy: present status**. *Cancer* 1978, **42**(5):2224-2233.
78. [550]. Murray JL, Kleinerman ES, Jia SF, Rosenblum MG, Eton O, Buzaid A, Legha S, Ross MI, Thompson L, Mujoo K *et al*: **Phase Ia/Ib trial of anti-GD2 chimeric monoclonal antibody 14.18 (ch14.18) and recombinant human granulocyte-macrophage colony-stimulating factor (rhGM-CSF) in metastatic melanoma**. *Journal of immunotherapy with emphasis on tumor immunology : official journal of the Society for Biological Therapy* 1996, **19**(3):206-217.
79. [551]. Newlands ES, Oon CJ, Roberts JT, Elliott P, Mould RF, Topham C, Madden FJ, Newton KA, Westbury G: **Clinical trial of combination chemotherapy and specific active immunotherapy in disseminated melanoma**. *British journal of cancer* 1976, **34**(2):174-179.
80. [552]. Nicholson S, Guile K, John J, Clarke IA, Diffley J, Donnellan P, Michael A, Szlosarek P, Dalgleish AG: **A randomized phase II trial of SRL172 (Mycobacterium vaccae) +/- low-dose interleukin-2 in the treatment of metastatic malignant melanoma**. *Melanoma research* 2003, **13**(4):389-393.
81. [553]. Nistico P, Capone I, Palermo B, Del Bello D, Ferraresi V, Moschella F, Arico E, Valentini M, Bracci L, Cognetti F *et al*: **Chemotherapy enhances vaccine-induced antitumor immunity in melanoma patients**. *International journal of cancer* 2009, **124**(1):130-139.
82. [554]. O'Brien ME, Saini A, Smith IE, Webb A, Gregory K, Mendes R, Ryan C, Priest K, Bromelow KV, Palmer RD *et al*: **A randomized phase II study of SRL172 (Mycobacterium vaccae) combined with chemotherapy in patients with advanced inoperable non-small-cell lung cancer and mesothelioma**. *British journal of cancer* 2000, **83**(7):853-857.
83. [555]. Olencki T, Finke J, Tubbs R, Tuason L, Greene T, McLain D, Swanson SJ, Herzog P, Stanley J, Edinger M *et al*: **Immunomodulatory effects of interleukin-2 and interleukin-4 in patients with malignancy**. *Journal of immunotherapy with emphasis on tumor immunology : official journal of the Society for Biological Therapy* 1996, **19**(1):69-80.
84. [556]. Olofsson Bagge R, Mattsson J, Hafstrom L: **Regional hyperthermic perfusion with melphalan after surgery for recurrent malignant melanoma of the extremities--long-term follow-up of a randomised trial**. *International journal of hyperthermia : the official journal of European Society for Hyperthermic Oncology, North American Hyperthermia Group* 2014, **30**(5):295-298.
85. [557]. Ott PA, Chang JL, Oratz R, Jones A, Farrell K, Muggia F, Pavlick AC: **Phase II trial of dacarbazine and thalidomide for the treatment of metastatic melanoma**. *Chemotherapy* 2009, **55**(4):221-227.
86. [558]. Panelli MC, Wunderlich J, Jeffries J, Wang E, Mixon A, Rosenberg SA, Marincola FM: **Phase 1 study in patients with metastatic melanoma of immunization with dendritic cells presenting epitopes derived from the melanoma-associated antigens MART-1 and gp100**. *Journal of immunotherapy (Hagerstown, Md : 1997)* 2000, **23**(4):487-498.
87. [559]. Paterson AH, Willans D, Jerry LM, McPherson TA: **Malignant melanoma (stage 1): a clinical trial of adjuvant BCG immunotherapy**. *Recent results in cancer research Fortschritte der Krebsforschung Progres dans les recherches sur le cancer* 1978, **68**:387-392.
88. [560]. Pilla L, Patuzzo R, Rivoltini L, Maio M, Pennacchioli E, Lamaj E, Maurichi A, Massarut S, Marchiano A, Santantonio C *et al*: **A phase II trial of vaccination with autologous, tumor-derived heat-shock protein peptide complexes Gp96, in combination with GM-CSF and interferon-alpha in metastatic melanoma patients**. *Cancer immunology, immunotherapy : CII* 2006, **55**(8):958-968.
89. [561]. Powell DJ, Jr., Dudley ME, Hogan KA, Wunderlich JR, Rosenberg SA: **Adoptive transfer of vaccine-induced peripheral blood mononuclear cells to patients with metastatic melanoma following lymphodepletion**. *Journal of immunology (Baltimore, Md : 1950)* 2006, **177**(9):6527-6539.
90. [562]. Presant CA, Bartolucci AA, Smalley RV, Vogler WR: **Cyclophosphamide plus 5-(3,3-dimethyl-1-triazeno)-imidazole-4-carboxamide (DTIC) with or without Corynebacterium parvum in metastatic malignant melanoma**. *Cancer* 1979, **44**(3):899-905.
91. [563]. Proebstle TM, Scheibenbogen C, Sterry W, Keilholz U: **A phase II study of dacarbazine, cisplatin, interferon-alpha and high-dose interleukin-2 in 'poor-risk' metastatic melanoma**. *European journal of cancer (Oxford, England : 1990)* 1996, **32a**(9):1530-1533.
92. [564]. Punt CJ, van Herpen CM, Jansen RL, Vreugdenhil G, Muller EW, de Mulder PH: **Chemoimmunotherapy with bleomycin, vincristine, lomustine, dacarbazine (BOLD) plus interferon alpha for metastatic melanoma: a multicentre phase II study**. *British journal of cancer* 1997, **76**(2):266-269.
93. [565]. Quan WD, Jr., Quan FM: **Activity of continuous infusion + pulse interleukin-2 with famotidine in metastatic melanoma**. *Cancer biotherapy & radiopharmaceuticals* 2009, **24**(1):1-6.
94. [566]. Queirolo P, Ponte M, Gipponi M, Cafiero F, Peressini A, Semino C, Pietra G, Lionetto R, Vecchio S, Ribizzi I *et al*: **Adoptive immunotherapy with tumor-infiltrating lymphocytes and subcutaneous recombinant interleukin-2 plus interferon alfa-2a for melanoma patients with nonresectable distant disease: a phase I/II pilot trial. Melanoma Istituto Scientifico Tumori Group**. *Annals of surgical oncology* 1999, **6**(3):272-278.
95. [567]. Ribas A, Comin-Anduix B, Chmielowski B, Jalil J, de la Rocha P, McCannel TA, Ochoa MT, Seja E, Villanueva A, Oseguera DK *et al*: **Dendritic cell vaccination combined with CTLA4 blockade in patients with metastatic melanoma**. *Clinical cancer research : an official journal of the American Association for Cancer Research* 2009, **15**(19):6267-6276.
96. [568]. Richards JM, Gale D, Mehta N, Lestingi T: **Combination of chemotherapy with interleukin-2 and interferon alfa for the treatment of metastatic melanoma**. *Journal of clinical oncology : official journal of the American Society of Clinical Oncology* 1999, **17**(2):651-657.
97. [569]. Ridolfi L, Fiorentini G, Guida M, Michiara M, Freschi A, Aitini E, Ballardini M, Bichisao E, Ridolfi R: **Multicentre, open, noncomparative Phase II trial to evaluate the efficacy and tolerability of fotemustine, cisplatin, alpha-interferon and interleukin-2 in advanced melanoma patients**. *Melanoma research* 2009, **19**(2):100-105.
98. [570]. Ridolfi L, Ridolfi R: **Preliminary experiences of intralesional immunotherapy in cutaneous metastatic melanoma**. *Hepato-gastroenterology* 2002, **49**(44):335-339.
99. [571]. Robidoux A, Gutterman JU, Bodey GP, Sr., Hersh EM: **Actinomycin-D plus 5-(3,3-dimethyl-1-triazeno)-imidazole-4-carboxamine (DTIC) with or without intravenous Corynebacterium parvum in metastatic malignant melanoma**. *Cancer* 1982, **49**(11):2246-2251.
100. [572]. Romano E, Michielin O, Voelter V, Laurent J, Bichat H, Stravodimou A, Romero P, Speiser DE, Triebel F, Leyvraz S *et al*: **MART-1 peptide vaccination plus IMP321 (LAG-3Ig fusion protein) in patients receiving autologous PBMCs after lymphodepletion: results of a Phase I trial**. *Journal of translational medicine* 2014, **12**:97.
101. [573]. Rosenberg SA, Lotze MT, Yang JC, Linehan WM, Seipp C, Calabro S, Karp SE, Sherry RM, Steinberg S, White DE: **Combination therapy with interleukin-2 and alpha-interferon for the treatment of patients with advanced cancer**. *Journal of clinical oncology : official journal of the American Society of Clinical Oncology* 1989, **7**(12):1863-1874.
102. [574]. Rosenberg SA, Yang JC, Schwartzentruber DJ, Hwu P, Marincola FM, Topalian SL, Restifo NP, Dudley ME, Schwarz SL, Spiess PJ *et al*: **Immunologic and therapeutic evaluation of a synthetic peptide vaccine for the treatment of patients with metastatic melanoma**. *Nature medicine* 1998, **4**(3):321-327.
103. [575]. Rosenberg SA, Yang JC, Schwartzentruber DJ, Hwu P, Marincola FM, Topalian SL, Seipp CA, Einhorn JH, White DE, Steinberg SM: **Prospective randomized trial of the treatment of patients with metastatic melanoma using chemotherapy with cisplatin, dacarbazine, and tamoxifen alone or in combination with interleukin-2 and interferon alfa-2b**. *Journal of clinical oncology : official journal of the American Society of Clinical Oncology* 1999, **17**(3):968-975.
104. [576]. Sabado RL, Pavlick A, Gnjatic S, Cruz CM, Vengco I, Hasan F, Spadaccia M, Darvishian F, Chiriboga L, Holman RM *et al*: **Resiquimod as an immunologic adjuvant for NY-ESO-1 protein vaccination in patients with high-risk melanoma**. *Cancer immunology research* 2015, **3**(3):278-287.
105. [577]. Sasse AD, Sasse EC, Clark LG, Ulloa L, Clark OA: **Chemoimmunotherapy versus chemotherapy for metastatic malignant melanoma**. *The Cochrane database of systematic reviews* 2007(1):Cd005413.
106. [578]. Scheibenbogen C, Schadendorf D, Bechrakis NE, Nagorsen D, Hofmann U, Servetopoulou F, Letsch A, Philipp A, Foerster MH, Schmittel A *et al*: **Effects of granulocyte-macrophage colony-stimulating factor and foreign helper protein as immunologic adjuvants on the T-cell response to vaccination with tyrosinase peptides**. *International journal of cancer* 2003, **104**(2):188-194.
107. [579]. Shelton E, Laharie D, Scott FI, Mamtani R, Lewis JD, Colombel JF, Ananthakrishnan AN: **Cancer Recurrence Following Immune-Suppressive Therapies in Patients With Immune-Mediated Diseases: A Systematic Review and Meta-analysis**. *Gastroenterology* 2016, **151**(1):97-109.e104.
108. [580]. Shiloni E, Pouillart P, Janssens J, Splinter T, Di Peri T, Symann M, Roest GJ, Palmer PA, Franks CR: **Sequential dacarbazine chemotherapy followed by recombinant interleukin-2 in metastatic melanoma. A pilot multicentre phase I-II study**. *European journal of cancer & clinical oncology* 1989, **25 Suppl 3**:S45-49.
109. [581]. Slingluff CL, Jr., Lee S, Zhao F, Chianese-Bullock KA, Olson WC, Butterfield LH, Whiteside TL, Leming PD, Kirkwood JM: **A randomized phase II trial of multiepitope vaccination with melanoma peptides for cytotoxic T cells and helper T cells for patients with metastatic melanoma (E1602)**. *Clinical cancer research : an official journal of the American Association for Cancer Research* 2013, **19**(15):4228-4238.
110. [582]. Smith IJ, Kurt RA, Baher AG, Denman S, Justice L, Doran T, Gilbert M, Alvord WG, Urba WJ: **Immune effects of escalating doses of granulocyte-macrophage colony-stimulating factor added to a fixed, low-dose, inpatient interleukin-2 regimen: a randomized phase I trial in patients with metastatic melanoma and renal cell carcinoma**. *Journal of immunotherapy (Hagerstown, Md : 1997)* 2003, **26**(2):130-138.
111. [583]. Soiffer RJ, Chapman PB, Murray C, Williams L, Unger P, Collins H, Houghton AN, Ritz J: **Administration of R24 monoclonal antibody and low-dose interleukin 2 for malignant melanoma**. *Clinical cancer research : an official journal of the American Association for Cancer Research* 1997, **3**(1):17-24.
112. [584]. Sondel PM, Hank JA: **Combination therapy with interleukin-2 and antitumor monoclonal antibodies**. *The cancer journal from Scientific American* 1997, **3 Suppl 1**:S121-127.
113. [585]. Stanojevic-Bakic N, Vuckovic-Dekic L, Milosevic D, Sasic M: **Effect of T-activin therapy on indomethacin modulation of lymphoproliferative response in vitro of melanoma patients**. *Panminerva medica* 1998, **40**(4):314-318.
114. [586]. Stoter G, Goey SH, Eggermont AM, Slingerland R, Braakman E, Lamers C, Bolhuis RL: **Interleukin-2. The experience of the Rotterdam Cancer Institute; Daniel den Hoed Kliniek**. *Biotherapy (Dordrecht, Netherlands)* 1990, **2**(3):261-265.
115. [587]. Straatsma BR, Nusinowitz S, Young TA, Gordon LK, Chun MW, Rosen C, Seja E, Economou JS, Glaspy JA, Bozon V *et al*: **Surveillance of the eye and vision in clinical trials of CP-675,206 for metastatic melanoma**. *American journal of ophthalmology* 2007, **143**(6):958-969.
116. [588]. Strobbe LJ, Hart AA, Rumke P, Israels SP, Nieweg OE, Kroon BB: **Topical dinitrochlorobenzene combined with systemic dacarbazine in the treatment of recurrent melanoma**. *Melanoma research* 1997, **7**(6):507-512.
117. [589]. Tarhini AA, Kirkwood JM, Gooding WE, Moschos S, Agarwala SS: **A phase 2 trial of sequential temozolomide chemotherapy followed by high-dose interleukin 2 immunotherapy for metastatic melanoma**. *Cancer* 2008, **113**(7):1632-1640.
118. [590]. Tartour E, Mathiot C, Fridman WH: **Current status of interleukin-2 therapy in cancer**. *Biomedicine & pharmacotherapy = Biomedecine & pharmacotherapie* 1992, **46**(10):473-484.
119. [591]. Tas F, Camlica H, Kurul S, Aydiner A, Topuz E: **Combination chemotherapy with docetaxel and irinotecan in metastatic malignant melanoma**. *Clinical oncology (Royal College of Radiologists (Great Britain))* 2003, **15**(3):132-135.
120. [592]. Terheyden P, Becker JC, Kampgen E, Brocker EB: **Sequential interferon-alpha2b, interleukin-2 and fotemustine for patients with metastatic melanoma**. *Melanoma research* 2000, **10**(5):475-482.
121. [593]. Topalian SL, Rosenberg SA: **Therapy of cancer using the adoptive transfer of activated killer cells and interleukin-2**. *Acta haematologica* 1987, **78 Suppl 1**:75-76.
122. [594]. Trepiakas R, Berntsen A, Hadrup SR, Bjorn J, Geertsen PF, Straten PT, Andersen MH, Pedersen AE, Soleimani A, Lorentzen T *et al*: **Vaccination with autologous dendritic cells pulsed with multiple tumor antigens for treatment of patients with malignant melanoma: results from a phase I/II trial**. *Cytotherapy* 2010, **12**(6):721-734.
123. [595]. Tuettenberg A, Becker C, Huter E, Knop J, Enk AH, Jonuleit H: **Induction of strong and persistent MelanA/MART-1-specific immune responses by adjuvant dendritic cell-based vaccination of stage II melanoma patients**. *International journal of cancer* 2006, **118**(10):2617-2627.
124. [596]. Vaishampayan U, Abrams J, Darrah D, Jones V, Mitchell MS: **Active immunotherapy of metastatic melanoma with allogeneic melanoma lysates and interferon alpha**. *Clinical cancer research : an official journal of the American Association for Cancer Research* 2002, **8**(12):3696-3701.
125. [597]. Vaughan MM, Moore J, Riches PG, Johnston SR, A'Hern RP, Hill ME, Eisen T, Ayliffe MJ, Thomas JM, Gore ME: **GM-CSF with biochemotherapy (cisplatin, DTIC, tamoxifen, IL-2 and interferon-alpha): a phase I trial in melanoma**. *Annals of oncology : official journal of the European Society for Medical Oncology* 2000, **11**(9):1183-1189.
126. [598]. Veelken H, Rosenthal FM, Schneller F, von Schilling C, Guettler IC, Herrmann F, Mertelsmann R, Lindemann A: **Combination of interleukin-2 and interferon-alpha in renal cell carcinoma and malignant melanoma: a phase II clinical trial**. *Biotechnology therapeutics* 1992, **3**(1-2):1-14.
127. [599]. Verdi CJ, Taylor CW, Croghan MK, Dalke P, Meyskens FL, Hersh EM: **Phase I study of low-dose cyclophosphamide and recombinant interleukin-2 for the treatment of advanced cancer**. *Journal of immunotherapy : official journal of the Society for Biological Therapy* 1992, **11**(4):286-291.
128. [600]. Veronesi U, Adamus J, Aubert C, Bajetta E, Beretta G, Bonadonna G, Bufalino R, Cascinelli N, Cocconi G, Durand J *et al*: **A randomized trial of adjuvant chemotherapy and immunotherapy in cutaneous melanoma**. *The New England journal of medicine* 1982, **307**(15):913-916.
129. [601]. Wages NA, Slingluff CL, Jr., Petroni GR: **A Phase I/II adaptive design to determine the optimal treatment regimen from a set of combination immunotherapies in high-risk melanoma**. *Contemporary clinical trials* 2015, **41**:172-179.
130. [602]. Wages NA, Slingluff CL, Jr., Petroni GR: **Statistical controversies in clinical research: early-phase adaptive design for combination immunotherapies**. *Annals of oncology : official journal of the European Society for Medical Oncology* 2017, **28**(4):696-701.
131. [603]. Weber J, Sondak VK, Scotland R, Phillip R, Wang F, Rubio V, Stuge TB, Groshen SG, Gee C, Jeffery GG *et al*: **Granulocyte-macrophage-colony-stimulating factor added to a multipeptide vaccine for resected Stage II melanoma**. *Cancer* 2003, **97**(1):186-200.
132. [604]. Weide B, Carralot JP, Reese A, Scheel B, Eigentler TK, Hoerr I, Rammensee HG, Garbe C, Pascolo S: **Results of the first phase I/II clinical vaccination trial with direct injection of mRNA**. *Journal of immunotherapy (Hagerstown, Md : 1997)* 2008, **31**(2):180-188.
133. [605]. Wood WC, Cosimi AB, Carey RW, Kaufman SD: **Randomized trial of adjuvant therapy for "high risk" primary malignant melanoma**. *Surgery* 1978, **83**(6):677-681.
134. [606]. Yamaguchi Y, Ohta K, Kawabuchi Y, Ohshita A, Okita R, Okawaki M, Hironaka K, Matsuura K, Toge T: **Feasibility study of adoptive immunotherapy for metastatic lung tumors using peptide-pulsed dendritic cell-activated killer (PDAK) cells**. *Anticancer research* 2005, **25**(3c):2407-2415.

# TOPIC: Melanoma and Immunotherapy Adverse Events

1. [607]. Adamina M, Weber WP, Rosenthal R, Schumacher R, Zajac P, Guller U, Frey DM, Oertli D, Zuber M, Heberer M *et al*: **Heterologous prime-boost immunotherapy of melanoma patients with Influenza virosomes, and recombinant Vaccinia virus encoding 5 melanoma epitopes and 3 co-stimulatory molecules. A multi-centre phase I/II open labeled clinical trial**. *Contemporary clinical trials* 2008, **29**(2):165-181.
2. [608]. Allen BJ, Singla AA, Rizvi SM, Graham P, Bruchertseifer F, Apostolidis C, Morgenstern A: **Analysis of patient survival in a Phase I trial of systemic targeted alpha-therapy for metastatic melanoma**. *Immunotherapy* 2011, **3**(9):1041-1050.
3. [609]. Andreesen R, Scheibenbogen C, Brugger W, Krause S, Meerpohl HG, Leser HG, Engler H, Lohr GW: **Adoptive transfer of tumor cytotoxic macrophages generated in vitro from circulating blood monocytes: a new approach to cancer immunotherapy**. *Cancer research* 1990, **50**(23):7450-7456.
4. [610]. Baars JW, Fonk JC, Scheper RJ, von Blomberg-van der Flier BM, Bril H, von Valk P, Pinedo HM, Wagstaff J: **Treatment with tumour infiltrating lymphocytes and interleukin-2 in patients with metastatic melanoma: a pilot study**. *Biotherapy (Dordrecht, Netherlands)* 1992, **4**(4):289-297.
5. [611]. Bartlett JB, Michael A, Clarke IA, Dredge K, Nicholson S, Kristeleit H, Polychronis A, Pandha H, Muller GW, Stirling DI *et al*: **Phase I study to determine the safety, tolerability and immunostimulatory activity of thalidomide analogue CC-5013 in patients with metastatic malignant melanoma and other advanced cancers**. *British journal of cancer* 2004, **90**(5):955-961.
6. [612]. Bedikian AY, Papadopoulos NE, Kim KB, Vardeleon A, Smith T, Lu B, Deitcher SR: **A pilot study with vincristine sulfate liposome infusion in patients with metastatic melanoma**. *Melanoma research* 2008, **18**(6):400-404.
7. [613]. Belli F, Arienti F, Sule-Suso J, Clemente C, Mascheroni L, Cattelan A, Santantonio C, Gallino GF, Melani C, Rao S *et al*: **Active immunization of metastatic melanoma patients with interleukin-2-transduced allogeneic melanoma cells: evaluation of efficacy and tolerability**. *Cancer immunology, immunotherapy : CII* 1997, **44**(4):197-203.
8. [614]. Besser MJ, Shapira-Frommer R, Treves AJ, Zippel D, Itzhaki O, Hershkovitz L, Levy D, Kubi A, Hovav E, Chermoshniuk N *et al*: **Clinical responses in a phase II study using adoptive transfer of short-term cultured tumor infiltration lymphocytes in metastatic melanoma patients**. *Clinical cancer research : an official journal of the American Association for Cancer Research* 2010, **16**(9):2646-2655.
9. [615]. Brossart P, Wirths S, Stuhler G, Reichardt VL, Kanz L, Brugger W: **Induction of cytotoxic T-lymphocyte responses in vivo after vaccinations with peptide-pulsed dendritic cells**. *Blood* 2000, **96**(9):3102-3108.
10. [616]. Calabro L, Morra A, Fonsatti E, Cutaia O, Fazio C, Annesi D, Lenoci M, Amato G, Danielli R, Altomonte M *et al*: **Efficacy and safety of an intensified schedule of tremelimumab for chemotherapy-resistant malignant mesothelioma: an open-label, single-arm, phase 2 study**. *The Lancet Respiratory medicine* 2015, **3**(4):301-309.
11. [617]. Carthon BC, Wolchok JD, Yuan J, Kamat A, Ng Tang DS, Sun J, Ku G, Troncoso P, Logothetis CJ, Allison JP *et al*: **Preoperative CTLA-4 blockade: tolerability and immune monitoring in the setting of a presurgical clinical trial**. *Clinical cancer research : an official journal of the American Association for Cancer Research* 2010, **16**(10):2861-2871.
12. [618]. Czarnetzki BM, Macher E, Suciu S, Thomas D, Steerenberg PA, Rumke P: **Long-term adjuvant immunotherapy in stage I high risk malignant melanoma, comparing two BCG preparations versus non-treatment in a randomised multicentre study (EORTC Protocol 18781)**. *European journal of cancer (Oxford, England : 1990)* 1993, **29a**(9):1237-1242.
13. [619]. Daud A, Valkov N, Centeno B, Derderian J, Sullivan P, Munster P, Urbas P, Deconti RC, Berghorn E, Liu Z *et al*: **Phase II trial of karenitecin in patients with malignant melanoma: clinical and translational study**. *Clinical cancer research : an official journal of the American Association for Cancer Research* 2005, **11**(8):3009-3016.
14. [620]. Davis ID, Chen Q, Morris L, Quirk J, Stanley M, Tavarnesi ML, Parente P, Cavicchiolo T, Hopkins W, Jackson H *et al*: **Blood dendritic cells generated with Flt3 ligand and CD40 ligand prime CD8+ T cells efficiently in cancer patients**. *Journal of immunotherapy (Hagerstown, Md : 1997)* 2006, **29**(5):499-511.
15. [621]. Duval L, Schmidt H, Kaltoft K, Fode K, Jensen JJ, Sorensen SM, Nishimura MI, von der Maase H: **Adoptive transfer of allogeneic cytotoxic T lymphocytes equipped with a HLA-A2 restricted MART-1 T-cell receptor: a phase I trial in metastatic melanoma**. *Clinical cancer research : an official journal of the American Association for Cancer Research* 2006, **12**(4):1229-1236.
16. [622]. Elliott GT, McLeod RA, Perez J, Von Eschen KB: **Interim results of a phase II multicenter clinical trial evaluating the activity of a therapeutic allogeneic melanoma vaccine (theraccine) in the treatment of disseminated malignant melanoma**. *Seminars in surgical oncology* 1993, **9**(3):264-272.
17. [623]. Finkelstein SE, Carey T, Fricke I, Yu D, Goetz D, Gratz M, Dunn M, Urbas P, Daud A, DeConti R *et al*: **Changes in dendritic cell phenotype after a new high-dose weekly schedule of interleukin-2 therapy for kidney cancer and melanoma**. *Journal of immunotherapy (Hagerstown, Md : 1997)* 2010, **33**(8):817-827.
18. [624]. Foppoli M, Citterio G, Polastri D, Guerrieri R: **The feasibility of repetitive courses of high-dose continuous intravenous infusion interleukin-2 and subcutaneous alpha-interferon with polychemotherapy in advanced malignant melanoma**. *Tumori* 1995, **81**(2):102-106.
19. [625]. Gollob JA, Sciambi CJ, Peterson BL, Richmond T, Thoreson M, Moran K, Dressman HK, Jelinek J, Issa JP: **Phase I trial of sequential low-dose 5-aza-2'-deoxycytidine plus high-dose intravenous bolus interleukin-2 in patients with melanoma or renal cell carcinoma**. *Clinical cancer research : an official journal of the American Association for Cancer Research* 2006, **12**(15):4619-4627.
20. [626]. Gomella LG, Mastrangelo MJ, McCue PA, Maguire HJ, Mulholland SG, Lattime EC: **Phase i study of intravesical vaccinia virus as a vector for gene therapy of bladder cancer**. *The Journal of urology* 2001, **166**(4):1291-1295.
21. [627]. Hersey P, Halliday GM, Farrelly ML, DeSilva C, Lett M, Menzies SW: **Phase I/II study of treatment with matured dendritic cells with or without low dose IL-2 in patients with disseminated melanoma**. *Cancer immunology, immunotherapy : CII* 2008, **57**(7):1039-1051.
22. [628]. Hersey P, Menzies SW, Halliday GM, Nguyen T, Farrelly ML, DeSilva C, Lett M: **Phase I/II study of treatment with dendritic cell vaccines in patients with disseminated melanoma**. *Cancer immunology, immunotherapy : CII* 2004, **53**(2):125-134.
23. [629]. Horvath J, Szabo-Szabari M, Sinkovics JG: **Autologous activated lymphocyte therapy in a community hospital**. *Acta microbiologica et immunologica Hungarica* 1994, **41**(2):205-214.
24. [630]. Houghton AN, Mintzer D, Cordon-Cardo C, Welt S, Fliegel B, Vadhan S, Carswell E, Melamed MR, Oettgen HF, Old LJ: **Mouse monoclonal IgG3 antibody detecting GD3 ganglioside: a phase I trial in patients with malignant melanoma**. *Proceedings of the National Academy of Sciences of the United States of America* 1985, **82**(4):1242-1246.
25. [631]. Hui KM, Ang PT, Huang L, Tay SK: **Phase I study of immunotherapy of cutaneous metastases of human carcinoma using allogeneic and xenogeneic MHC DNA-liposome complexes**. *Gene therapy* 1997, **4**(8):783-790.
26. [632]. Kefford RF, Clingan PR, Brady B, Ballmer A, Morganti A, Hersey P: **A randomized, double-blind, placebo-controlled study of high-dose bosentan in patients with stage IV metastatic melanoma receiving first-line dacarbazine chemotherapy**. *Molecular cancer* 2010, **9**:69.
27. [633]. Keilholz U, Scheibenbogen C, Brado M, Georgi P, Maclachlan D, Brado B, Hunstein W: **Regional adoptive immunotherapy with interleukin-2 and lymphokine-activated killer (LAK) cells for liver metastases**. *European journal of cancer (Oxford, England : 1990)* 1994, **30a**(1):103-105.
28. [634]. Keilholz U, Scheibenbogen C, Tilgen W, Bergmann L, Weidmann E, Seither E, Richter M, Brado B, Mitrou PS, Hunstein W: **Interferon-alpha and interleukin-2 in the treatment of metastatic melanoma. Comparison of two phase II trials**. *Cancer* 1993, **72**(2):607-614.
29. [635]. Klein O, Ebert LM, Nicholaou T, Browning J, Russell SE, Zuber M, Jackson HM, Dimopoulos N, Tan BS, Hoos A *et al*: **Melan-A-specific cytotoxic T cells are associated with tumor regression and autoimmunity following treatment with anti-CTLA-4**. *Clinical cancer research : an official journal of the American Association for Cancer Research* 2009, **15**(7):2507-2513.
30. [636]. Kradin RL, Kurnick JT, Lazarus DS, Preffer FI, Dubinett SM, Pinto CE, Gifford J, Davidson E, Grove B, Callahan RJ *et al*: **Tumour-infiltrating lymphocytes and interleukin-2 in treatment of advanced cancer**. *Lancet (London, England)* 1989, **1**(8638):577-580.
31. [637]. Krause SW, Neumann C, Soruri A, Mayer S, Peters JH, Andreesen R: **The treatment of patients with disseminated malignant melanoma by vaccination with autologous cell hybrids of tumor cells and dendritic cells**. *Journal of immunotherapy (Hagerstown, Md : 1997)* 2002, **25**(5):421-428.
32. [638]. Lawson DH, Lee S, Zhao F, Tarhini AA, Margolin KA, Ernstoff MS, Atkins MB, Cohen GI, Whiteside TL, Butterfield LH *et al*: **Randomized, Placebo-Controlled, Phase III Trial of Yeast-Derived Granulocyte-Macrophage Colony-Stimulating Factor (GM-CSF) Versus Peptide Vaccination Versus GM-CSF Plus Peptide Vaccination Versus Placebo in Patients With No Evidence of Disease After Complete Surgical Resection of Locally Advanced and/or Stage IV Melanoma: A Trial of the Eastern Cooperative Oncology Group-American College of Radiology Imaging Network Cancer Research Group (E4697)**. *Journal of clinical oncology : official journal of the American Society of Clinical Oncology* 2015, **33**(34):4066-4076.
33. [639]. Lee JH, Lee Y, Lee M, Heo MK, Song JS, Kim KH, Lee H, Yi NJ, Lee KW, Suh KS *et al*: **A phase I/IIa study of adjuvant immunotherapy with tumour antigen-pulsed dendritic cells in patients with hepatocellular carcinoma**. *British journal of cancer* 2015, **113**(12):1666-1676.
34. [640]. Leong SP, Enders-Zohr P, Zhou YM, Stuntebeck S, Habib FA, Allen RE, Jr., Sagebiel RW, Glassberg AB, Lowenberg DW, Hayes FA: **Recombinant human granulocyte macrophage-colony stimulating factor (rhGM-CSF) and autologous melanoma vaccine mediate tumor regression in patients with metastatic melanoma**. *Journal of immunotherapy (Hagerstown, Md : 1997)* 1999, **22**(2):166-174.
35. [641]. Lopez M, Fechtenbaum J, David B, Martinache C, Chokri M, Canepa S, De Gramont A, Louvet C, Gorin I, Mortel O *et al*: **Adoptive immunotherapy with activated macrophages grown in vitro from blood monocytes in cancer patients: a pilot study**. *Journal of immunotherapy : official journal of the Society for Biological Therapy* 1992, **11**(3):209-217.
36. [642]. Mackensen A, Herbst B, Chen JL, Kohler G, Noppen C, Herr W, Spagnoli GC, Cerundolo V, Lindemann A: **Phase I study in melanoma patients of a vaccine with peptide-pulsed dendritic cells generated in vitro from CD34(+) hematopoietic progenitor cells**. *International journal of cancer* 2000, **86**(3):385-392.
37. [643]. Mackiewicz J, Karczewska-Dzionk A, Laciak M, Kapcinska M, Wiznerowicz M, Burzykowski T, Zakowska M, Rose-John S, Mackiewicz A: **Whole Cell Therapeutic Vaccine Modified With Hyper-IL6 for Combinational Treatment of Nonresected Advanced Melanoma**. *Medicine* 2015, **94**(21):e853.
38. [644]. Mier JW, Aronson FR, Numerof RP, Vachino G, Atkins MB: **Toxicity of immunotherapy with interleukin-2 and lymphokine-activated killer cells**. *Pathology and immunopathology research* 1988, **7**(6):459-476.
39. [645]. Mitchell MS, Abrams J, Thompson JA, Kashani-Sabet M, DeConti RC, Hwu WJ, Atkins MB, Whitman E, Ernstoff MS, Haluska FG *et al*: **Randomized trial of an allogeneic melanoma lysate vaccine with low-dose interferon Alfa-2b compared with high-dose interferon Alfa-2b for Resected stage III cutaneous melanoma**. *Journal of clinical oncology : official journal of the American Society of Clinical Oncology* 2007, **25**(15):2078-2085.
40. [646]. Mittelman A, Chen ZJ, Yang H, Wong GY, Ferrone S: **Human high molecular weight melanoma-associated antigen (HMW-MAA) mimicry by mouse anti-idiotypic monoclonal antibody MK2-23: induction of humoral anti-HMW-MAA immunity and prolongation of survival in patients with stage IV melanoma**. *Proceedings of the National Academy of Sciences of the United States of America* 1992, **89**(2):466-470.
41. [647]. Nagayama H, Sato K, Morishita M, Uchimaru K, Oyaizu N, Inazawa T, Yamasaki T, Enomoto M, Nakaoka T, Nakamura T *et al*: **Results of a phase I clinical study using autologous tumour lysate-pulsed monocyte-derived mature dendritic cell vaccinations for stage IV malignant melanoma patients combined with low dose interleukin-2**. *Melanoma research* 2003, **13**(5):521-530.
42. [648]. O'Rourke MG, Johnson M, Lanagan C, See J, Yang J, Bell JR, Slater GJ, Kerr BM, Crowe B, Purdie DM *et al*: **Durable complete clinical responses in a phase I/II trial using an autologous melanoma cell/dendritic cell vaccine**. *Cancer immunology, immunotherapy : CII* 2003, **52**(6):387-395.
43. [649]. Osanto S, Schiphorst PP, Weijl NI, Dijkstra N, Van Wees A, Brouwenstein N, Vaessen N, Van Krieken JH, Hermans J, Cleton FJ *et al*: **Vaccination of melanoma patients with an allogeneic, genetically modified interleukin 2-producing melanoma cell line**. *Human gene therapy* 2000, **11**(5):739-750.
44. [650]. Paciucci PA, Holland JF, Glidewell O, Odchimar R: **Recombinant interleukin-2 by continuous infusion and adoptive transfer of recombinant interleukin-2-activated cells in patients with advanced cancer**. *Journal of clinical oncology : official journal of the American Society of Clinical Oncology* 1989, **7**(7):869-878.
45. [651]. Ravaud A, Legrand E, Delaunay MM, Bussieres E, Coulon V, Cany L, Huet S, Verdier D, Kind M, Chomy F *et al*: **A phase I trial of repeated tumour-infiltrating lymphocyte (TIL) infusion in metastatic melanoma**. *British journal of cancer* 1995, **71**(2):331-336.
46. [652]. Rochlitz C, Dreno B, Jantscheff P, Cavalli F, Squiban P, Acres B, Baudin M, Escudier B, Heinzerling L, Morant R *et al*: **Immunotherapy of metastatic melanoma by intratumoral injections of Vero cells producing human IL-2: phase II randomized study comparing two dose levels**. *Cancer gene therapy* 2002, **9**(3):289-295.
47. [653]. Rosenberg SA, Lotze MT, Muul LM, Leitman S, Chang AE, Ettinghausen SE, Matory YL, Skibber JM, Shiloni E, Vetto JT *et al*: **Observations on the systemic administration of autologous lymphokine-activated killer cells and recombinant interleukin-2 to patients with metastatic cancer**. *The New England journal of medicine* 1985, **313**(23):1485-1492.
48. [654]. Sadanaga N, Nagashima H, Mashino K, Tahara K, Yamaguchi H, Ohta M, Fujie T, Tanaka F, Inoue H, Takesako K *et al*: **Dendritic cell vaccination with MAGE peptide is a novel therapeutic approach for gastrointestinal carcinomas**. *Clinical cancer research : an official journal of the American Association for Cancer Research* 2001, **7**(8):2277-2284.
49. [655]. Salcedo M, Bercovici N, Taylor R, Vereecken P, Massicard S, Duriau D, Vernel-Pauillac F, Boyer A, Baron-Bodo V, Mallard E *et al*: **Vaccination of melanoma patients using dendritic cells loaded with an allogeneic tumor cell lysate**. *Cancer immunology, immunotherapy : CII* 2006, **55**(7):819-829.
50. [656]. Schiza A, Wenthe J, Mangsbo S, Eriksson E, Nilsson A, Totterman TH, Loskog A, Ullenhag G: **Adenovirus-mediated CD40L gene transfer increases Teffector/Tregulatory cell ratio and upregulates death receptors in metastatic melanoma patients**. *Journal of translational medicine* 2017, **15**(1):79.
51. [657]. Shackleton M, Davis ID, Hopkins W, Jackson H, Dimopoulos N, Tai T, Chen Q, Parente P, Jefford M, Masterman KA *et al*: **The impact of imiquimod, a Toll-like receptor-7 ligand (TLR7L), on the immunogenicity of melanoma peptide vaccination with adjuvant Flt3 ligand**. *Cancer immunity* 2004, **4**:9.
52. [658]. Stebbing J, Dalgleish A, Gifford-Moore A, Martin A, Gleeson C, Wilson G, Brunet LR, Grange J, Mudan S: **An intra-patient placebo-controlled phase I trial to evaluate the safety and tolerability of intradermal IMM-101 in melanoma**. *Annals of oncology : official journal of the European Society for Medical Oncology* 2012, **23**(5):1314-1319.
53. [659]. Tanaka F, Haraguchi N, Isikawa K, Inoue H, Mori M: **Potential role of dendritic cell vaccination with MAGE peptides in gastrointestinal carcinomas**. *Oncology reports* 2008, **20**(5):1111-1116.
54. [660]. Thompson JA, Curti BD, Redman BG, Bhatia S, Weber JS, Agarwala SS, Sievers EL, Hughes SD, DeVries TA, Hausman DF: **Phase I study of recombinant interleukin-21 in patients with metastatic melanoma and renal cell carcinoma**. *Journal of clinical oncology : official journal of the American Society of Clinical Oncology* 2008, **26**(12):2034-2039.
55. [661]. Thompson JA, Gold PJ, Fefer A: **Outpatient chemoimmunotherapy for the treatment of metastatic melanoma**. *Seminars in oncology* 1997, **24**(1 Suppl 4):S44-48.
56. [662]. Thurner B, Haendle I, Roder C, Dieckmann D, Keikavoussi P, Jonuleit H, Bender A, Maczek C, Schreiner D, von den Driesch P *et al*: **Vaccination with mage-3A1 peptide-pulsed mature, monocyte-derived dendritic cells expands specific cytotoxic T cells and induces regression of some metastases in advanced stage IV melanoma**. *The Journal of experimental medicine* 1999, **190**(11):1669-1678.
57. [663]. Toh U, Yamana H, Kido K, Mine T, Fujii T, Horiuchi H, Sasatomi T, Ishibashi N, Yutani S, Fujita H *et al*: **[Autologous tumor specific immunotherapy of refractory cancers with ex vivo-generated T cells stimulated by autologous tumor cell]**. *Gan to kagaku ryoho Cancer & chemotherapy* 2003, **30**(11):1566-1570.
58. [664]. Ursu R, Taillibert S, Banissi C, Vicaut E, Bailon O, Le Rhun E, Guillamo JS, Psimaras D, Tibi A, Sacko A *et al*: **Immunotherapy with CpG-ODN in neoplastic meningitis: A phase I trial**. *Cancer science* 2015, **106**(9):1212-1218.
59. [665]. Van Gool AR, Fekkes D, Kruit WH, Mulder PG, Ten Hagen TL, Bannink M, Maes M, Eggermont AM: **Serum amino acids, biopterin and neopterin during long-term immunotherapy with interferon-alpha in high-risk melanoma patients**. *Psychiatry research* 2003, **119**(1-2):125-132.
60. [666]. Veelken H, Mackensen A, Lahn M, Kohler G, Becker D, Franke B, Brennscheidt U, Kulmburg P, Rosenthal FM, Keller H *et al*: **A phase-I clinical study of autologous tumor cells plus interleukin-2-gene-transfected allogeneic fibroblasts as a vaccine in patients with cancer**. *International journal of cancer* 1997, **70**(3):269-277.
61. [667]. Wallack MK, McNally K, Michaelides M, Bash J, Bartolucci A, Siegler H, Balch C, Wanebo H: **A phase I/II SECSG (Southeastern Cancer Study Group) pilot study of surgical adjuvant immunotherapy with vaccinia melanoma oncolysates (VMO)**. *The American surgeon* 1986, **52**(3):148-151.
62. [668]. Weide B, Pascolo S, Scheel B, Derhovanessian E, Pflugfelder A, Eigentler TK, Pawelec G, Hoerr I, Rammensee HG, Garbe C: **Direct injection of protamine-protected mRNA: results of a phase 1/2 vaccination trial in metastatic melanoma patients**. *Journal of immunotherapy (Hagerstown, Md : 1997)* 2009, **32**(5):498-507.
63. [669]. Wolchok JD, Neyns B, Linette G, Negrier S, Lutzky J, Thomas L, Waterfield W, Schadendorf D, Smylie M, Guthrie T, Jr. *et al*: **Ipilimumab monotherapy in patients with pretreated advanced melanoma: a randomised, double-blind, multicentre, phase 2, dose-ranging study**. *The Lancet Oncology* 2010, **11**(2):155-164.
64. [670]. Wolchok JD, Yuan J, Houghton AN, Gallardo HF, Rasalan TS, Wang J, Zhang Y, Ranganathan R, Chapman PB, Krown SE *et al*: **Safety and immunogenicity of tyrosinase DNA vaccines in patients with melanoma**. *Molecular therapy : the journal of the American Society of Gene Therapy* 2007, **15**(11):2044-2050.
65. [671]. Yuan J, Gnjatic S, Li H, Powel S, Gallardo HF, Ritter E, Ku GY, Jungbluth AA, Segal NH, Rasalan TS *et al*: **CTLA-4 blockade enhances polyfunctional NY-ESO-1 specific T cell responses in metastatic melanoma patients with clinical benefit**. *Proceedings of the National Academy of Sciences of the United States of America* 2008, **105**(51):20410-20415.

# TOPIC: Melanoma and Immunotherapy Toxicity

1. [672]. Aamdal S, Bruntsch U, Kerger J, Verweij J, ten Bokkel Huinink W, Wanders J, Rastogi R, Franklin HR, Kaye SB: **Zeniplatin in advanced malignant melanoma and renal cancer: phase II studies with unexpected nephrotoxicity**. *Cancer chemotherapy and pharmacology* 1997, **40**(5):439-443.
2. [673]. Albertini MR, Ranheim EA, Zuleger CL, Sondel PM, Hank JA, Bridges A, Newton MA, McFarland T, Collins J, Clements E *et al*: **Phase I study to evaluate toxicity and feasibility of intratumoral injection of alpha-gal glycolipids in patients with advanced melanoma**. *Cancer immunology, immunotherapy : CII* 2016, **65**(8):897-907.
3. [674]. Albertini MR, Sosman JA, Hank JA, Moore KH, Borchert A, Schell K, Kohler PC, Bechhofer R, Storer B, Sondel PM: **The influence of autologous lymphokine-activated killer cell infusions on the toxicity and antitumor effect of repetitive cycles of interleukin-2**. *Cancer* 1990, **66**(12):2457-2464.
4. [675]. Baars JW, Hack CE, Wagstaff J, Eerenberg-Belmer AJ, Wolbink GJ, Thijs LG, Strack van Schijndel RJ, van der Vall HL, Pinedo HM: **The activation of polymorphonuclear neutrophils and the complement system during immunotherapy with recombinant interleukin-2**. *British journal of cancer* 1992, **65**(1):96-101.
5. [676]. Balch CM, Smalley RV, Bartolucci AA, Burns D, Presant CA, Durant JR: **A randomized prospective clinical trial of adjuvant C. parvum immunotherapy in 260 patients with clinically localized melanoma (Stage I): prognostic factors analysis and preliminary results of immunotherapy**. *Cancer* 1982, **49**(6):1079-1084.
6. [677]. Bedrosian I, Mick R, Xu S, Nisenbaum H, Faries M, Zhang P, Cohen PA, Koski G, Czerniecki BJ: **Intranodal administration of peptide-pulsed mature dendritic cell vaccines results in superior CD8+ T-cell function in melanoma patients**. *Journal of clinical oncology : official journal of the American Society of Clinical Oncology* 2003, **21**(20):3826-3835.
7. [678]. Belli F, Testori A, Rivoltini L, Maio M, Andreola G, Sertoli MR, Gallino G, Piris A, Cattelan A, Lazzari I *et al*: **Vaccination of metastatic melanoma patients with autologous tumor-derived heat shock protein gp96-peptide complexes: clinical and immunologic findings**. *Journal of clinical oncology : official journal of the American Society of Clinical Oncology* 2002, **20**(20):4169-4180.
8. [679]. Besser MJ, Shapira-Frommer R, Treves AJ, Zippel D, Itzhaki O, Schallmach E, Kubi A, Shalmon B, Hardan I, Catane R *et al*: **Minimally cultured or selected autologous tumor-infiltrating lymphocytes after a lympho-depleting chemotherapy regimen in metastatic melanoma patients**. *Journal of immunotherapy (Hagerstown, Md : 1997)* 2009, **32**(4):415-423.
9. [680]. Bettinotti MP, Panelli MC, Ruppe E, Mocellin S, Phan GQ, White DE, Marincola FM: **Clinical and immunological evaluation of patients with metastatic melanoma undergoing immunization with the HLA-Cw*0702-associated epitope MAGE-A12:170-178**. *International journal of cancer* 2003, **105**(2):210-216.
10. [681]. Bigner DD, Brown M, Coleman RE, Friedman AH, Friedman HS, McLendon RE, Bigner SH, Zhao XG, Wikstrand CJ, Pegram CN *et al*: **Phase I studies of treatment of malignant gliomas and neoplastic meningitis with 131I-radiolabeled monoclonal antibodies anti-tenascin 81C6 and anti-chondroitin proteoglycan sulfate Me1-14 F (ab')2--a preliminary report**. *Journal of neuro-oncology* 1995, **24**(1):109-122.
11. [682]. Bol KF, Mensink HW, Aarntzen EH, Schreibelt G, Keunen JE, Coulie PG, de Klein A, Punt CJ, Paridaens D, Figdor CG *et al*: **Long overall survival after dendritic cell vaccination in metastatic uveal melanoma patients**. *American journal of ophthalmology* 2014, **158**(5):939-947.
12. [683]. Borrione P, Montacchini L, Beggiato E, Pileri A, Bianchi A, Massaia M: **Clinical and immunological studies in advanced cancer patients sequentially treated with anti CD3 monoclonal antibody (OKT3) and interleukin-2**. *Leukemia & lymphoma* 1996, **21**(3-4):325-330.
13. [684]. Burdette-Radoux S, Tozer RG, Lohmann RC, Quirt I, Ernst DS, Walsh W, Wainman N, Colevas AD, Eisenhauer EA: **Phase II trial of flavopiridol, a cyclin dependent kinase inhibitor, in untreated metastatic malignant melanoma**. *Investigational new drugs* 2004, **22**(3):315-322.
14. [685]. Burgdorf SK, Fischer A, Claesson MH, Kirkin AF, Dzhandzhugazyan KN, Rosenberg J: **Vaccination with melanoma lysate-pulsed dendritic cells, of patients with advanced colorectal carcinoma: report from a phase I study**. *Journal of experimental & clinical cancer research : CR* 2006, **25**(2):201-206.
15. [686]. Butterfield LH, Comin-Anduix B, Vujanovic L, Lee Y, Dissette VB, Yang JQ, Vu HT, Seja E, Oseguera DK, Potter DM *et al*: **Adenovirus MART-1-engineered autologous dendritic cell vaccine for metastatic melanoma**. *Journal of immunotherapy (Hagerstown, Md : 1997)* 2008, **31**(3):294-309.
16. [687]. Butterfield LH, Ribas A, Dissette VB, Amarnani SN, Vu HT, Oseguera D, Wang HJ, Elashoff RM, McBride WH, Mukherji B *et al*: **Determinant spreading associated with clinical response in dendritic cell-based immunotherapy for malignant melanoma**. *Clinical cancer research : an official journal of the American Association for Cancer Research* 2003, **9**(3):998-1008.
17. [688]. Bystryn JC, Zeleniuch-Jacquotte A, Oratz R, Shapiro RL, Harris MN, Roses DF: **Double-blind trial of a polyvalent, shed-antigen, melanoma vaccine**. *Clinical cancer research : an official journal of the American Association for Cancer Research* 2001, **7**(7):1882-1887.
18. [689]. Caraceni A, Gangeri L, Martini C, Belli F, Brunelli C, Baldini M, Mascheroni L, Lenisa L, Cascinelli N: **Neurotoxicity of interferon-alpha in melanoma therapy: results from a randomized controlled trial**. *Cancer* 1998, **83**(3):482-489.
19. [690]. Casper ES, Bajorin D: **Phase II trial of carboplatin in patients with advanced melanoma**. *Investigational new drugs* 1990, **8**(2):187-190.
20. [691]. Chakraborty NG, Sporn JR, Tortora AF, Kurtzman SH, Yamase H, Ergin MT, Mukherji B: **Immunization with a tumor-cell-lysate-loaded autologous-antigen-presenting-cell-based vaccine in melanoma**. *Cancer immunology, immunotherapy : CII* 1998, **47**(1):58-64.
21. [692]. Chang AE, Redman BG, Whitfield JR, Nickoloff BJ, Braun TM, Lee PP, Geiger JD, Mule JJ: **A phase I trial of tumor lysate-pulsed dendritic cells in the treatment of advanced cancer**. *Clinical cancer research : an official journal of the American Association for Cancer Research* 2002, **8**(4):1021-1032.
22. [693]. Citterio G, Pellegatta F, Lucca GD, Fragasso G, Scaglietti U, Pini D, Fortis C, Tresoldi M, Rugarli C: **Plasma nitrate plus nitrite changes during continuous intravenous infusion interleukin 2**. *British journal of cancer* 1996, **74**(8):1297-1301.
23. [694]. Conlon KC, Lugli E, Welles HC, Rosenberg SA, Fojo AT, Morris JC, Fleisher TA, Dubois SP, Perera LP, Stewart DM *et al*: **Redistribution, hyperproliferation, activation of natural killer cells and CD8 T cells, and cytokine production during first-in-human clinical trial of recombinant human interleukin-15 in patients with cancer**. *Journal of clinical oncology : official journal of the American Society of Clinical Oncology* 2015, **33**(1):74-82.
24. [695]. Cui CL, Chi ZH, Yuan XQ, Lian HY, Si L, Guo J: **[Hepatic intra-arterial bio-chemotherapy for the treatment of melanoma patients with liver metastasis: a phase II clinical study]**. *Ai zheng = Aizheng = Chinese journal of cancer* 2008, **27**(8):845-850.
25. [696]. Cure H, Souteyrand P, Ouabdesselam R, Roche H, Ravaud A, D'Incan M, Viens P, Fargeot P, Lentz MA, Fumoleau P *et al*: **Results of a phase II trial with cystemustine at 90 mg/m(2) as a first- or second-line treatment in advanced malignant melanoma: a trial of the EORTC Clinical Studies Group**. *Melanoma research* 1999, **9**(6):607-610.
26. [697]. Daud AI, DeConti RC, Andrews S, Urbas P, Riker AI, Sondak VK, Munster PN, Sullivan DM, Ugen KE, Messina JL *et al*: **Phase I trial of interleukin-12 plasmid electroporation in patients with metastatic melanoma**. *Journal of clinical oncology : official journal of the American Society of Clinical Oncology* 2008, **26**(36):5896-5903.
27. [698]. DeConti RC, Algazi AP, Andrews S, Urbas P, Born O, Stoeckigt D, Floren L, Hwang J, Weber J, Sondak VK *et al*: **Phase II trial of sagopilone, a novel epothilone analog in metastatic melanoma**. *British journal of cancer* 2010, **103**(10):1548-1553.
28. [699]. Dessureault S, Noyes D, Lee D, Dunn M, Janssen W, Cantor A, Sotomayor E, Messina J, Antonia SJ: **A phase-I trial using a universal GM-CSF-producing and CD40L-expressing bystander cell line (GM.CD40L) in the formulation of autologous tumor cell-based vaccines for cancer patients with stage IV disease**. *Annals of surgical oncology* 2007, **14**(2):869-884.
29. [700]. Diaz A, Alfonso M, Alonso R, Saurez G, Troche M, Catala M, Diaz RM, Perez R, Vazquez AM: **Immune responses in breast cancer patients immunized with an anti-idiotype antibody mimicking NeuGc-containing gangliosides**. *Clinical immunology (Orlando, Fla)* 2003, **107**(2):80-89.
30. [701]. Dillman RO, DeLeon C, Beutel LD, Barth NM, Schwartzberg LS, Spitler LE, Garfield DH, O'Connor AA, Nayak SK: **Short-term autologous tumor cell lines for the active specific immunotherapy of patients with metastatic melanoma**. *Critical reviews in oncology/hematology* 2001, **39**(1-2):115-123.
31. [702]. Dutcher JP, Creekmore S, Weiss GR, Margolin K, Markowitz AB, Roper M, Parkinson D, Ciobanu N, Fisher RI, Boldt DH *et al*: **A phase II study of interleukin-2 and lymphokine-activated killer cells in patients with metastatic malignant melanoma**. *Journal of clinical oncology : official journal of the American Society of Clinical Oncology* 1989, **7**(4):477-485.
32. [703]. Economou JS, Hoban M, Lee JD, Essner R, Swisher S, McBride W, Hoon DB, Morton DL: **Production of tumor necrosis factor alpha and interferon gamma in interleukin-2-treated melanoma patients: correlation with clinical toxicity**. *Cancer immunology, immunotherapy : CII* 1991, **34**(1):49-52.
33. [704]. Emmert S, Zutt M, Haenssle H, Neumann C, Kretschmer L: **Inefficacy of vindesine monotherapy in advanced stage IV malignant melanoma patients previously treated with other chemotherapeutic agents**. *Melanoma research* 2003, **13**(3):299-302.
34. [705]. Ernst DS, Eisenhauer E, Wainman N, Davis M, Lohmann R, Baetz T, Belanger K, Smylie M: **Phase II study of perifosine in previously untreated patients with metastatic melanoma**. *Investigational new drugs* 2005, **23**(6):569-575.
35. [706]. Eton O, Kharkevitch DD, Gianan MA, Ross MI, Itoh K, Pride MW, Donawho C, Buzaid AC, Mansfield PF, Lee JE *et al*: **Active immunotherapy with ultraviolet B-irradiated autologous whole melanoma cells plus DETOX in patients with metastatic melanoma**. *Clinical cancer research : an official journal of the American Association for Cancer Research* 1998, **4**(3):619-627.
36. [707]. Eton O, Ross MI, East MJ, Mansfield PF, Papadopoulos N, Ellerhorst JA, Bedikian AY, Lee JE: **Autologous tumor-derived heat-shock protein peptide complex-96 (HSPPC-96) in patients with metastatic melanoma**. *Journal of translational medicine* 2010, **8**:9.
37. [708]. Filippetti M, Torsello A, Cordiali Fei P, Bordignon V, Trento E, Tonachella R, Piperno G, Prignano G: **[IL-2 bronchoscopic istillation and immune cell activation: preliminary results of the BRIIL-2 study for treatment of pulmonary metastasis from renal cancer and melanoma]**. *La Clinica terapeutica* 2009, **160**(2):139-143.
38. [709]. Foon KA, Lutzky J, Baral RN, Yannelli JR, Hutchins L, Teitelbaum A, Kashala OL, Das R, Garrison J, Reisfeld RA *et al*: **Clinical and immune responses in advanced melanoma patients immunized with an anti-idiotype antibody mimicking disialoganglioside GD2**. *Journal of clinical oncology : official journal of the American Society of Clinical Oncology* 2000, **18**(2):376-384.
39. [710]. Foon KA, Sen G, Hutchins L, Kashala OL, Baral R, Banerjee M, Chakraborty M, Garrison J, Reisfeld RA, Bhattacharya-Chatterjee M: **Antibody responses in melanoma patients immunized with an anti-idiotype antibody mimicking disialoganglioside GD2**. *Clinical cancer research : an official journal of the American Association for Cancer Research* 1998, **4**(5):1117-1124.
40. [711]. Galanis E, Hersh EM, Stopeck AT, Gonzalez R, Burch P, Spier C, Akporiaye ET, Rinehart JJ, Edmonson J, Sobol RE *et al*: **Immunotherapy of advanced malignancy by direct gene transfer of an interleukin-2 DNA/DMRIE/DOPE lipid complex: phase I/II experience**. *Journal of clinical oncology : official journal of the American Society of Clinical Oncology* 1999, **17**(10):3313-3323.
41. [712]. Ginsberg BA, Gallardo HF, Rasalan TS, Adamow M, Mu Z, Tandon S, Bewkes BB, Roman RA, Chapman PB, Schwartz GK *et al*: **Immunologic response to xenogeneic gp100 DNA in melanoma patients: comparison of particle-mediated epidermal delivery with intramuscular injection**. *Clinical cancer research : an official journal of the American Association for Cancer Research* 2010, **16**(15):4057-4065.
42. [713]. Goedegebuure PS, Douville LM, Li H, Richmond GC, Schoof DD, Scavone M, Eberlein TJ: **Adoptive immunotherapy with tumor-infiltrating lymphocytes and interleukin-2 in patients with metastatic malignant melanoma and renal cell carcinoma: a pilot study**. *Journal of clinical oncology : official journal of the American Society of Clinical Oncology* 1995, **13**(8):1939-1949.
43. [714]. Guillot B, Khamari A, Cupissol D, Delaunay M, Bedane C, Dreno B, Picot MC, Dereure O: **Temozolomide associated with PEG-interferon in patients with metastatic melanoma: a multicenter prospective phase I/II study**. *Melanoma research* 2008, **18**(2):141-146.
44. [715]. Hsueh EC, Nathanson L, Foshag LJ, Essner R, Nizze JA, Stern SL, Morton DL: **Active specific immunotherapy with polyvalent melanoma cell vaccine for patients with in-transit melanoma metastases**. *Cancer* 1999, **85**(10):2160-2169.
45. [716]. Ives NJ, Stowe RL, Lorigan P, Wheatley K: **Chemotherapy compared with biochemotherapy for the treatment of metastatic melanoma: a meta-analysis of 18 trials involving 2,621 patients**. *Journal of clinical oncology : official journal of the American Society of Clinical Oncology* 2007, **25**(34):5426-5434.
46. [717]. Joshua AM, Monzon JG, Mihalcioiu C, Hogg D, Smylie M, Cheng T: **A phase 2 study of tremelimumab in patients with advanced uveal melanoma**. *Melanoma research* 2015, **25**(4):342-347.
47. [718]. Karp SE: **Low-dose intravenous bolus interleukin-2 with interferon-alpha therapy for metastatic melanoma and renal cell carcinoma**. *Journal of immunotherapy (Hagerstown, Md : 1997)* 1998, **21**(1):56-61.
48. [719]. Khorana AA, Rosenblatt JD, Sahasrabudhe DM, Evans T, Ladrigan M, Marquis D, Rosell K, Whiteside T, Phillippe S, Acres B *et al*: **A phase I trial of immunotherapy with intratumoral adenovirus-interferon-gamma (TG1041) in patients with malignant melanoma**. *Cancer gene therapy* 2003, **10**(4):251-259.
49. [720]. Kirkwood JM, Ibrahim JG, Sosman JA, Sondak VK, Agarwala SS, Ernstoff MS, Rao U: **High-dose interferon alfa-2b significantly prolongs relapse-free and overall survival compared with the GM2-KLH/QS-21 vaccine in patients with resected stage IIB-III melanoma: results of intergroup trial E1694/S9512/C509801**. *Journal of clinical oncology : official journal of the American Society of Clinical Oncology* 2001, **19**(9):2370-2380.
50. [721]. Krishnadas DK, Shusterman S, Bai F, Diller L, Sullivan JE, Cheerva AC, George RE, Lucas KG: **A phase I trial combining decitabine/dendritic cell vaccine targeting MAGE-A1, MAGE-A3 and NY-ESO-1 for children with relapsed or therapy-refractory neuroblastoma and sarcoma**. *Cancer immunology, immunotherapy : CII* 2015, **64**(10):1251-1260.
51. [722]. Kruit WH, Goey SH, Calabresi F, Lindemann A, Stahel RA, Poliwoda H, Osterwalder B, Stoter G: **Final report of a phase II study of interleukin 2 and interferon alpha in patients with metastatic melanoma**. *British journal of cancer* 1995, **71**(6):1319-1321.
52. [723]. Lau R, Wang F, Jeffery G, Marty V, Kuniyoshi J, Bade E, Ryback ME, Weber J: **Phase I trial of intravenous peptide-pulsed dendritic cells in patients with metastatic melanoma**. *Journal of immunotherapy (Hagerstown, Md : 1997)* 2001, **24**(1):66-78.
53. [724]. Laurent J, Speiser DE, Appay V, Touvrey C, Vicari M, Papaioannou A, Canellini G, Rimoldi D, Rufer N, Romero P *et al*: **Impact of 3 different short-term chemotherapy regimens on lymphocyte-depletion and reconstitution in melanoma patients**. *Journal of immunotherapy (Hagerstown, Md : 1997)* 2010, **33**(7):723-734.
54. [725]. Lee P, Wang F, Kuniyoshi J, Rubio V, Stuges T, Groshen S, Gee C, Lau R, Jeffery G, Margolin K *et al*: **Effects of interleukin-12 on the immune response to a multipeptide vaccine for resected metastatic melanoma**. *Journal of clinical oncology : official journal of the American Society of Clinical Oncology* 2001, **19**(18):3836-3847.
55. [726]. Legha SS: **Interferons in the treatment of malignant melanoma. A review of recent trials**. *Cancer* 1986, **57**(8 Suppl):1675-1677.
56. [727]. Lesimple T, Moisan A, Carsin A, Ollivier I, Mousseau M, Meunier B, Leberre C, Collet B, Quillien V, Drenou B *et al*: **Injection by various routes of melanoma antigen-associated macrophages: biodistribution and clinical effects**. *Cancer immunology, immunotherapy : CII* 2003, **52**(7):438-444.
57. [728]. Li X, Naylor MF, Le H, Nordquist RE, Teague TK, Howard CA, Murray C, Chen WR: **Clinical effects of in situ photoimmunotherapy on late-stage melanoma patients: a preliminary study**. *Cancer biology & therapy* 2010, **10**(11):1081-1087.
58. [729]. Lindner P, Rizell M, Mattsson J, Hellstrand K, Naredi P: **Combined treatment with histamine dihydrochloride, interleukin-2 and interferon-alpha in patients with metastatic melanoma**. *Anticancer research* 2004, **24**(3b):1837-1842.
59. [730]. Lissoni P, Vaghi M, Ardizzoia A, Malugani F, Fumagalli E, Bordin V, Fumagalli L, Bordoni A, Mengo S, Gardani GS *et al*: **A phase II study of chemoneuroimmunotherapy with platinum, subcutaneous low-dose interleukin-2 and the pineal neurohormone melatonin (P.I.M.) as a second-line therapy in metastatic melanoma patients progressing on dacarbazine plus interferon-alpha**. *In vivo (Athens, Greece)* 2002, **16**(2):93-96.
60. [731]. Lopez M, Escobar A, Alfaro J, Fodor M, Larrondo M, Ferrada C, Salazar-Onfray F: **[Advances in cellular immunotherapy for malignant melanoma]**. *Revista medica de Chile* 2004, **132**(9):1115-1126.
61. [732]. Luiten RM, Kueter EW, Mooi W, Gallee MP, Rankin EM, Gerritsen WR, Clift SM, Nooijen WJ, Weder P, van de Kasteele WF *et al*: **Immunogenicity, including vitiligo, and feasibility of vaccination with autologous GM-CSF-transduced tumor cells in metastatic melanoma patients**. *Journal of clinical oncology : official journal of the American Society of Clinical Oncology* 2005, **23**(35):8978-8991.
62. [733]. Mahvi DM, Henry MB, Albertini MR, Weber S, Meredith K, Schalch H, Rakhmilevich A, Hank J, Sondel P: **Intratumoral injection of IL-12 plasmid DNA--results of a phase I/IB clinical trial**. *Cancer gene therapy* 2007, **14**(8):717-723.
63. [734]. Marchand M, van Baren N, Weynants P, Brichard V, Dreno B, Tessier MH, Rankin E, Parmiani G, Arienti F, Humblet Y *et al*: **Tumor regressions observed in patients with metastatic melanoma treated with an antigenic peptide encoded by gene MAGE-3 and presented by HLA-A1**. *International journal of cancer* 1999, **80**(2):219-230.
64. [735]. Margolin KA, Rayner AA, Hawkins MJ, Atkins MB, Dutcher JP, Fisher RI, Weiss GR, Doroshow JH, Jaffe HS, Roper M *et al*: **Interleukin-2 and lymphokine-activated killer cell therapy of solid tumors: analysis of toxicity and management guidelines**. *Journal of clinical oncology : official journal of the American Society of Clinical Oncology* 1989, **7**(4):486-498.
65. [736]. Markovic SN, Suman VJ, Nevala WK, Geeraerts L, Creagan ET, Erickson LA, Rowland KM, Jr., Morton RF, Horvath WL, Pittelkow MR: **A dose-escalation study of aerosolized sargramostim in the treatment of metastatic melanoma: an NCCTG Study**. *American journal of clinical oncology* 2008, **31**(6):573-579.
66. [737]. Mitchell MS, Darrah D, Yeung D, Halpern S, Wallace A, Voland J, Jones V, Kan-Mitchell J: **Phase I trial of adoptive immunotherapy with cytolytic T lymphocytes immunized against a tyrosinase epitope**. *Journal of clinical oncology : official journal of the American Society of Clinical Oncology* 2002, **20**(4):1075-1086.
67. [738]. Mittelman A, Chen ZJ, Kageshita T, Yang H, Yamada M, Baskind P, Goldberg N, Puccio C, Ahmed T, Arlin Z *et al*: **Active specific immunotherapy in patients with melanoma. A clinical trial with mouse antiidiotypic monoclonal antibodies elicited with syngeneic anti-high-molecular-weight-melanoma-associated antigen monoclonal antibodies**. *The Journal of clinical investigation* 1990, **86**(6):2136-2144.
68. [739]. Moller P, Sun Y, Dorbic T, Alijagic S, Makki A, Jurgovsky K, Schroff M, Henz BM, Wittig B, Schadendorf D: **Vaccination with IL-7 gene-modified autologous melanoma cells can enhance the anti-melanoma lytic activity in peripheral blood of patients with a good clinical performance status: a clinical phase I study**. *British journal of cancer* 1998, **77**(11):1907-1916.
69. [740]. Morgan LR, Krementz ET, Fan SW, Fan D: **Adoptive immunotherapy of advanced renal cell cancer using PHA-stimulated autologous lymphocytes**. *Anticancer research* 1993, **13**(5c):1763-1767.
70. [741]. Morgan RA, Chinnasamy N, Abate-Daga D, Gros A, Robbins PF, Zheng Z, Dudley ME, Feldman SA, Yang JC, Sherry RM *et al*: **Cancer regression and neurological toxicity following anti-MAGE-A3 TCR gene therapy**. *Journal of immunotherapy (Hagerstown, Md : 1997)* 2013, **36**(2):133-151.
71. [742]. Nakai N, Asai J, Ueda E, Takenaka H, Katoh N, Kishimoto S: **Vaccination of Japanese patients with advanced melanoma with peptide, tumor lysate or both peptide and tumor lysate-pulsed mature, monocyte-derived dendritic cells**. *The Journal of dermatology* 2006, **33**(7):462-472.
72. [743]. Newton DA, Romano C, Gattoni-Celli S: **Semiallogeneic cell hybrids as therapeutic vaccines for cancer**. *Journal of immunotherapy (Hagerstown, Md : 1997)* 2000, **23**(2):246-254.
73. [744]. Osorio M, Gracia E, Rodriguez E, Saurez G, Arango Mdel C, Noris E, Torriella A, Joan A, Gomez E, Anasagasti L *et al*: **Heterophilic NeuGcGM3 ganglioside cancer vaccine in advanced melanoma patients: results of a Phase Ib/IIa study**. *Cancer biology & therapy* 2008, **7**(4):488-495.
74. [745]. Ota K: **Review of ubenimex (Bestatin): clinical research**. *Biomedicine & pharmacotherapy = Biomedecine & pharmacotherapie* 1991, **45**(2-3):55-60.
75. [746]. Ota K, Uzuka Y: **Clinical trials of bestatin for leukemia and solid tumors**. *Biotherapy (Dordrecht, Netherlands)* 1992, **4**(3):205-214.
76. [747]. Parney IF, Chang LJ, Farr-Jones MA, Hao C, Smylie M, Petruk KC: **Technical hurdles in a pilot clinical trial of combined B7-2 and GM-CSF immunogene therapy for glioblastomas and melanomas**. *Journal of neuro-oncology* 2006, **78**(1):71-80.
77. [748]. Propper DJ, Chao D, Braybrooke JP, Bahl P, Thavasu P, Balkwill F, Turley H, Dobbs N, Gatter K, Talbot DC *et al*: **Low-dose IFN-gamma induces tumor MHC expression in metastatic malignant melanoma**. *Clinical cancer research : an official journal of the American Association for Cancer Research* 2003, **9**(1):84-92.
78. [749]. Punt CJ, Suciu S, Gore MA, Koller J, Kruit WH, Thomas J, Patel P, Lienard D, Eggermont AM, Keilholz U: **Chemoimmunotherapy with dacarbazine, cisplatin, interferon-alpha2b and interleukin-2 versus two cycles of dacarbazine followed by chemoimmunotherapy in patients with metastatic melanoma: a randomised phase II study of the European Organization for Research and Treatment of Cancer Melanoma Group**. *European journal of cancer (Oxford, England : 1990)* 2006, **42**(17):2991-2995.
79. [750]. Quaak SG, van den Berg JH, Toebes M, Schumacher TN, Haanen JB, Beijnen JH, Nuijen B: **GMP production of pDERMATT for vaccination against melanoma in a phase I clinical trial**. *European journal of pharmaceutics and biopharmaceutics : official journal of Arbeitsgemeinschaft fur Pharmazeutische Verfahrenstechnik eV* 2008, **70**(2):429-438.
80. [751]. Quan W, Jr., Brick W, Vinogradov M, Taylor WC, Khan N, Burgess R: **Repeated cycles with 72-hour continuous infusion interleukin-2 in kidney cancer and melanoma**. *Cancer biotherapy & radiopharmaceuticals* 2004, **19**(3):350-354.
81. [752]. Quan W, Jr., Ramirez M, Taylor C, Quan F, Vinogradov M, Walker P: **Administration of high-dose continuous infusion interleukin-2 to patients age 70 or over**. *Cancer biotherapy & radiopharmaceuticals* 2005, **20**(1):11-15.
82. [753]. Quan WD, Jr., Dean GE, Spears L, Spears CP, Groshen S, Merritt JA, Mitchell MS: **Active specific immunotherapy of metastatic melanoma with an antiidiotype vaccine: a phase I/II trial of I-Mel-2 plus SAF-m**. *Journal of clinical oncology : official journal of the American Society of Clinical Oncology* 1997, **15**(5):2103-2110.
83. [754]. Raja C, Graham P, Abbas Rizvi SM, Song E, Goldsmith H, Thompson J, Bosserhoff A, Morgenstern A, Apostolidis C, Kearsley J *et al*: **Interim analysis of toxicity and response in phase 1 trial of systemic targeted alpha therapy for metastatic melanoma**. *Cancer biology & therapy* 2007, **6**(6):846-852.
84. [755]. Recchia F, Candeloro G, Necozione S, Fumagalli L, Bratta M, Rea S: **Multicenter phase II study of chemoimmunotherapy in the treatment of metastatic melanoma**. *Anti-cancer drugs* 2008, **19**(2):201-207.
85. [756]. Redman BG, Chang AE, Whitfield J, Esper P, Jiang G, Braun T, Roessler B, Mule JJ: **Phase Ib trial assessing autologous, tumor-pulsed dendritic cells as a vaccine administered with or without IL-2 in patients with metastatic melanoma**. *Journal of immunotherapy (Hagerstown, Md : 1997)* 2008, **31**(6):591-598.
86. [757]. Ridolfi R, Chiarion-Sileni V, Guida M, Romanini A, Labianca R, Freschi A, Lo Re G, Nortilli R, Brugnara S, Vitali P *et al*: **Cisplatin, dacarbazine with or without subcutaneous interleukin-2, and interferon alpha-2b in advanced melanoma outpatients: results from an Italian multicenter phase III randomized clinical trial**. *Journal of clinical oncology : official journal of the American Society of Clinical Oncology* 2002, **20**(6):1600-1607.
87. [758]. Rochlitz C, Jantscheff P, Bongartz G, Dietrich PY, Quiquerez AL, Schatz C, Mehtali M, Courtney M, Tartour E, Dorval T *et al*: **Gene therapy study of cytokine-transfected xenogeneic cells (Vero-interleukin-2) in patients with metastatic solid tumors**. *Cancer gene therapy* 1999, **6**(3):271-281.
88. [759]. Rochlitz CF, Jantscheff P, Bongartz G, Dietrich PY, Quiquerez AL, Schatz C, Mehtali M, Courtney M, Tartour E, Dorval T *et al*: **Gene therapy with cytokine-transfected xenogeneic cells in metastatic tumors**. *Advances in experimental medicine and biology* 1998, **451**:531-537.
89. [760]. Rosenberg SA, Lotze MT, Muul LM, Leitman S, Chang AE, Vetto JT, Seipp CA, Simpson C: **A new approach to the therapy of cancer based on the systemic administration of autologous lymphokine-activated killer cells and recombinant interleukin-2**. *Surgery* 1986, **100**(2):262-272.
90. [761]. Royal RE, Steinberg SM, Krouse RS, Heywood G, White DE, Hwu P, Marincola FM, Parkinson DR, Schwartzentruber DJ, Topalian SL *et al*: **Correlates of response to IL-2 therapy in patients treated for metastatic renal cancer and melanoma**. *The cancer journal from Scientific American* 1996, **2**(2):91-98.
91. [762]. Ryan CW, Shulman KL, Richards JM, Kugler JW, Sosman JA, Ansari RH, Vokes EE, Vogelzang NJ: **CI-980 in advanced melanoma and hormone refractory prostate cancer**. *Investigational new drugs* 2000, **18**(2):187-191.
92. [763]. Salgaller ML, Tjoa BA, Lodge PA, Ragde H, Kenny G, Boynton A, Murphy GP: **Dendritic cell-based immunotherapy of prostate cancer**. *Critical reviews in immunology* 1998, **18**(1-2):109-119.
93. [764]. Schroff RW, Morgan AC, Jr., Woodhouse CS, Abrams PG, Farrell MM, Carpenter BE, Oldham RK, Foon KA: **Monoclonal antibody therapy in malignant melanoma: factors effecting in vivo localization**. *Journal of biological response modifiers* 1987, **6**(4):457-472.
94. [765]. Seledtsov VI, Niza NA, Felde MA, Shishkov AA, Samarin DM, Seledtsova GV, Seledtsov DV: **Xenovaccinotherapy for colorectal cancer**. *Biomedicine & pharmacotherapy = Biomedecine & pharmacotherapie* 2007, **61**(2-3):125-130.
95. [766]. Seo W, Ogasawara H, Sakagami M: **[Chemohormonal therapy for malignant melanomas]**. *Nihon Jibiinkoka Gakkai kaiho* 1996, **99**(7):1004-1009.
96. [767]. Smithers M, O'Connell K, MacFadyen S, Chambers M, Greenwood K, Boyce A, Abdul-Jabbar I, Barker K, Grimmett K, Walpole E *et al*: **Clinical response after intradermal immature dendritic cell vaccination in metastatic melanoma is associated with immune response to particulate antigen**. *Cancer immunology, immunotherapy : CII* 2003, **52**(1):41-52.
97. [768]. Sondak VK, Liu PY, Tuthill RJ, Kempf RA, Unger JM, Sosman JA, Thompson JA, Weiss GR, Redman BG, Jakowatz JG *et al*: **Adjuvant immunotherapy of resected, intermediate-thickness, node-negative melanoma with an allogeneic tumor vaccine: overall results of a randomized trial of the Southwest Oncology Group**. *Journal of clinical oncology : official journal of the American Society of Clinical Oncology* 2002, **20**(8):2058-2066.
98. [769]. Stift A, Friedl J, Dubsky P, Bachleitner-Hofmann T, Schueller G, Zontsich T, Benkoe T, Radelbauer K, Brostjan C, Jakesz R *et al*: **Dendritic cell-based vaccination in solid cancer**. *Journal of clinical oncology : official journal of the American Society of Clinical Oncology* 2003, **21**(1):135-142.
99. [770]. Teulings HE, Limpens J, Jansen SN, Zwinderman AH, Reitsma JB, Spuls PI, Luiten RM: **Vitiligo-like depigmentation in patients with stage III-IV melanoma receiving immunotherapy and its association with survival: a systematic review and meta-analysis**. *Journal of clinical oncology : official journal of the American Society of Clinical Oncology* 2015, **33**(7):773-781.
100. [771]. Thompson JA, Gold PJ, Markowitz DR, Byrd DR, Lindgren CG, Fefer A: **Updated analysis of an outpatient chemoimmunotherapy regimen for treating metastatic melanoma**. *The cancer journal from Scientific American* 1997, **3 Suppl 1**:S29-34.
101. [772]. Triozzi PL, Aldrich W, Allen KO, Carlisle RR, LoBuglio AF, Conry RM: **Phase I study of a plasmid DNA vaccine encoding MART-1 in patients with resected melanoma at risk for relapse**. *Journal of immunotherapy (Hagerstown, Md : 1997)* 2005, **28**(4):382-388.
102. [773]. Vansteenkiste J, Zielinski M, Linder A, Dahabreh J, Gonzalez EE, Malinowski W, Lopez-Brea M, Vanakesa T, Jassem J, Kalofonos H *et al*: **Adjuvant MAGE-A3 immunotherapy in resected non-small-cell lung cancer: phase II randomized study results**. *Journal of clinical oncology : official journal of the American Society of Clinical Oncology* 2013, **31**(19):2396-2403.
103. [774]. Verdecchia GM, Ridolfi L, Ridolfi R, Riccobon A, Bertagni A, Vagliasindi A, Petrini M, Stefanelli M, Milandri C, Amadori D: **[Adjuvant adoptive immunotherapy in patients with stage III and resected stage IV melanoma: a pilot study]**. *Tumori* 2003, **89**(4 Suppl):298-300.
104. [775]. Vilella R, Benitez D, Mila J, Lozano M, Vilana R, Pomes J, Tomas X, Costa J, Vilalta A, Malvehy J *et al*: **Pilot study of treatment of biochemotherapy-refractory stage IV melanoma patients with autologous dendritic cells pulsed with a heterologous melanoma cell line lysate**. *Cancer immunology, immunotherapy : CII* 2004, **53**(7):651-658.
105. [776]. Wallack MK, Bash JA, McNally KR, Leftheriotis E: **Serological evaluation of melanoma patients in a phase I/II trial of vaccinia melanoma oncolysate (VMO) immunotherapy**. *Cancer detection and prevention Supplement : official publication of the International Society for Preventive Oncology, Inc* 1987, **1**:351-359.
106. [777]. Wallack MK, Sivanandham M, Ditaranto K, Shaw P, Balch CM, Urist MM, Bland KI, Murray D, Robinson WA, Flaherty L *et al*: **Increased survival of patients treated with a vaccinia melanoma oncolysate vaccine: second interim analysis of data from a phase III, multi-institutional trial**. *Annals of surgery* 1997, **226**(2):198-206.
107. [778]. Wang F, Bade E, Kuniyoshi C, Spears L, Jeffery G, Marty V, Groshen S, Weber J: **Phase I trial of a MART-1 peptide vaccine with incomplete Freund's adjuvant for resected high-risk melanoma**. *Clinical cancer research : an official journal of the American Association for Cancer Research* 1999, **5**(10):2756-2765.
108. [779]. Wei Y, Sticca RP, Holmes LM, Burgin KE, Li J, Williamson J, Evans L, Smith SJ, Stephenson JJ, Wagner TE: **Dendritoma vaccination combined with low dose interleukin-2 in metastatic melanoma patients induced immunological and clinical responses**. *International journal of oncology* 2006, **28**(3):585-593.
109. [780]. Whitehead RP, Unger JM, Flaherty LE, Kraut EH, Mills GM, Klein CE, Chapman RA, Doolittle GC, Hammond N, Sondak VK: **A phase II trial of pyrazine diazohydroxide in patients with disseminated malignant melanoma and no prior chemotherapy--Southwest Oncology Group study**. *Investigational new drugs* 2002, **20**(1):105-111.
110. [781]. Wiseman CL, Rao VS, Kennedy PS, Presant CA, Smith JD, McKenna RJ: **Clinical responses with active specific intralymphatic immunotherapy for cancer--a phase I-II trial**. *The Western journal of medicine* 1989, **151**(3):283-288.
111. [782]. Yee C, Thompson JA, Byrd D, Riddell SR, Roche P, Celis E, Greenberg PD: **Adoptive T cell therapy using antigen-specific CD8+ T cell clones for the treatment of patients with metastatic melanoma: in vivo persistence, migration, and antitumor effect of transferred T cells**. *Proceedings of the National Academy of Sciences of the United States of America* 2002, **99**(25):16168-16173.
112. [783]. Yuan J, Ku GY, Gallardo HF, Orlandi F, Manukian G, Rasalan TS, Xu Y, Li H, Vyas S, Mu Z *et al*: **Safety and immunogenicity of a human and mouse gp100 DNA vaccine in a phase I trial of patients with melanoma**. *Cancer immunity* 2009, **9**:5.
113. [784]. Zajac P, Oertli D, Marti W, Adamina M, Bolli M, Guller U, Noppen C, Padovan E, Schultz-Thater E, Heberer M *et al*: **Phase I/II clinical trial of a nonreplicative vaccinia virus expressing multiple HLA-A0201-restricted tumor-associated epitopes and costimulatory molecules in metastatic melanoma patients**. *Human gene therapy* 2003, **14**(16):1497-1510.
114. [785]. Zhang L, Morgan RA, Beane JD, Zheng Z, Dudley ME, Kassim SH, Nahvi AV, Ngo LT, Sherry RM, Phan GQ *et al*: **Tumor-infiltrating lymphocytes genetically engineered with an inducible gene encoding interleukin-12 for the immunotherapy of metastatic melanoma**. *Clinical cancer research : an official journal of the American Association for Cancer Research* 2015, **21**(10):2278-2288.

# TOPIC: Author Recommended Additions

1. [786]. **Cancer Stat Facts: Melanoma of the Skin** [https://seer.cancer.gov/statfacts/html/melan.html]
2. [787]. **Cancer Immunotherapy Guidelines** [https://www.sitcancer.org/research/cancer-immunotherapy-guidelines]
3. [788]. **FDA grants accelerated approval to pembrolizumab for first tissue/site agnostic indication** [https://www.fda.gov/Drugs/InformationOnDrugs/ApprovedDrugs/ucm560040.htm]
4. [789]. In: *Clinical Practice Guidelines We Can Trust.* edn. Edited by Graham R, Mancher M, Miller Wolman D, Greenfield S, Steinberg E. Washington (DC); 2011.
5. [790]. Ackerman A, Klein O, McDermott DF, Wang W, Ibrahim N, Lawrence DP, Gunturi A, Flaherty KT, Hodi FS, Kefford R *et al*: **Outcomes of patients with metastatic melanoma treated with immunotherapy prior to or after BRAF inhibitors**. *Cancer* 2014, **120**(11):1695-1701.
6. [791]. Ascierto PA, Simeone E, Sileni VC, Del Vecchio M, Marchetti P, Cappellini GC, Ridolfi R, de Rosa F, Cognetti F, Ferraresi V *et al*: **Sequential treatment with ipilimumab and BRAF inhibitors in patients with metastatic melanoma: data from the Italian cohort of the ipilimumab expanded access program**. *Cancer Invest* 2014, **32**(4):144-149.
7. [792]. Atkins MB, Kunkel L, Sznol M, Rosenberg SA: **High-dose recombinant interleukin-2 therapy in patients with metastatic melanoma: long-term survival update**. *The cancer journal from Scientific American* 2000, **6 Suppl 1**:S11-14.
8. [793]. Atkins MB, Lotze MT, Dutcher JP, Fisher RI, Weiss G, Margolin K, Abrams J, Sznol M, Parkinson D, Hawkins M *et al*: **High-dose recombinant interleukin 2 therapy for patients with metastatic melanoma: analysis of 270 patients treated between 1985 and 1993**. *Journal of clinical oncology : official journal of the American Society of Clinical Oncology* 1999, **17**(7):2105-2116.
9. [794]. Bottomley A, Coens C, Suciu S, Santinami M, Kruit W, Testori A, Marsden J, Punt C, Sales F, Gore M *et al*: **Adjuvant therapy with pegylated interferon alfa-2b versus observation in resected stage III melanoma: a phase III randomized controlled trial of health-related quality of life and symptoms by the European Organisation for Research and Treatment of Cancer Melanoma Group**. *Journal of clinical oncology : official journal of the American Society of Clinical Oncology* 2009, **27**(18):2916-2923.
10. [795]. Deutsch GB, Flaherty DC, Kirchoff DD, Bailey M, Vitug S, Foshag LJ, Faries MB, Bilchik AJ: **Association of Surgical Treatment, Systemic Therapy, and Survival in Patients With Abdominal Visceral Melanoma Metastases, 1965-2014: Relevance of Surgical Cure in the Era of Modern Systemic Therapy**. *JAMA Surg* 2017, **152**(7):672-678.
11. [796]. Deutsch GB, Kirchoff DD, Faries MB: **Metastasectomy for stage IV melanoma**. *Surg Oncol Clin N Am* 2015, **24**(2):279-298.
12. [797]. Dutcher JP, Schwartzentruber DJ, Kaufman HL, Agarwala SS, Tarhini AA, Lowder JN, Atkins MB: **High dose interleukin-2 (Aldesleukin) - expert consensus on best management practices-2014**. *Journal for ImmunoTherapy of Cancer* 2014, **2**(1):26.
13. [798]. Eggermont AM, Suciu S, Santinami M, Testori A, Kruit WH, Marsden J, Punt CJ, Sales F, Gore M, Mackie R *et al*: **Adjuvant therapy with pegylated interferon alfa-2b versus observation alone in resected stage III melanoma: final results of EORTC 18991, a randomised phase III trial**. *Lancet (London, England)* 2008, **372**(9633):117-126.
14. [799]. Faries MB, Thompson JF, Cochran AJ, Andtbacka RH, Mozzillo N, Zager JS, Jahkola T, Bowles TL, Testori A, Beitsch PD *et al*: **Completion Dissection or Observation for Sentinel-Node Metastasis in Melanoma**. *The New England journal of medicine* 2017, **376**(23):2211-2222.
15. [800]. Gerami P, Cook RW, Wilkinson J, Russell MC, Dhillon N, Amaria RN, Gonzalez R, Lyle S, Johnson CE, Oelschlager KM *et al*: **Development of a prognostic genetic signature to predict the metastatic risk associated with cutaneous melanoma**. *Clinical cancer research : an official journal of the American Association for Cancer Research* 2015, **21**(1):175-183.
16. [801]. Grob JJ, Dreno B, de la Salmoniere P, Delaunay M, Cupissol D, Guillot B, Souteyrand P, Sassolas B, Cesarini JP, Lionnet S *et al*: **Randomised trial of interferon alpha-2a as adjuvant therapy in resected primary melanoma thicker than 1.5 mm without clinically detectable node metastases. French Cooperative Group on Melanoma**. *Lancet (London, England)* 1998, **351**(9120):1905-1910.
17. [802]. Guirguis LM, Yang JC, White DE, Steinberg SM, Liewehr DJ, Rosenberg SA, Schwartzentruber DJ: **Safety and efficacy of high-dose interleukin-2 therapy in patients with brain metastases**. *Journal of immunotherapy (Hagerstown, Md : 1997)* 2002, **25**(1):82-87.
18. [803]. Hancock BW, Wheatley K, Harris S, Ives N, Harrison G, Horsman JM, Middleton MR, Thatcher N, Lorigan PC, Marsden JR *et al*: **Adjuvant interferon in high-risk melanoma: the AIM HIGH Study--United Kingdom Coordinating Committee on Cancer Research randomized study of adjuvant low-dose extended-duration interferon Alfa-2a in high-risk resected malignant melanoma**. *Journal of clinical oncology : official journal of the American Society of Clinical Oncology* 2004, **22**(1):53-61.
19. [804]. Hauschild A, Weichenthal M, Balda BR, Becker JC, Wolff HH, Tilgen W, Schulte KW, Ring J, Schadendorf D, Lischner S *et al*: **Prospective randomized trial of interferon alfa-2b and interleukin-2 as adjuvant treatment for resected intermediate- and high-risk primary melanoma without clinically detectable node metastasis**. *Journal of clinical oncology : official journal of the American Society of Clinical Oncology* 2003, **21**(15):2883-2888.
20. [805]. Hauschild A, Weichenthal M, Rass K, Linse R, Berking C, Bottjer J, Vogt T, Spieth K, Eigentler T, Brockmeyer NH *et al*: **Efficacy of low-dose interferon {alpha}2a 18 versus 60 months of treatment in patients with primary melanoma of >= 1.5 mm tumor thickness: results of a randomized phase III DeCOG trial**. *Journal of clinical oncology : official journal of the American Society of Clinical Oncology* 2010, **28**(5):841-846.
21. [806]. Hauschild A, Weichenthal M, Rass K, Linse R, Ulrich J, Stadler R, Volkenandt M, Grabbe S, Proske U, Schadendorf D *et al*: **Prospective randomized multicenter adjuvant dermatologic cooperative oncology group trial of low-dose interferon alfa-2b with or without a modified high-dose interferon alfa-2b induction phase in patients with lymph node-negative melanoma**. *Journal of clinical oncology : official journal of the American Society of Clinical Oncology* 2009, **27**(21):3496-3502.
22. [807]. Hayes AJ, Maynard L, Coombes G, Newton-Bishop J, Timmons M, Cook M, Theaker J, Bliss JM, Thomas JM, Group UKMS *et al*: **Wide versus narrow excision margins for high-risk, primary cutaneous melanomas: long-term follow-up of survival in a randomised trial**. *The Lancet Oncology* 2016, **17**(2):184-192.
23. [808]. Hodi FS, O'Day SJ, McDermott DF, Weber RW, Sosman JA, Haanen JB, Gonzalez R, Robert C, Schadendorf D, Hassel JC *et al*: **Improved survival with ipilimumab in patients with metastatic melanoma**. *The New England journal of medicine* 2010, **363**(8):711-723.
24. [809]. Johnson DB, Balko JM, Compton ML, Chalkias S, Gorham J, Xu Y, Hicks M, Puzanov I, Alexander MR, Bloomer TL *et al*: **Fulminant Myocarditis with Combination Immune Checkpoint Blockade**. *The New England journal of medicine* 2016, **375**(18):1749-1755.
25. [810]. Kirkwood JM, Ibrahim JG, Sondak VK, Richards J, Flaherty LE, Ernstoff MS, Smith TJ, Rao U, Steele M, Blum RH: **High- and low-dose interferon alfa-2b in high-risk melanoma: first analysis of intergroup trial E1690/S9111/C9190**. *Journal of clinical oncology : official journal of the American Society of Clinical Oncology* 2000, **18**(12):2444-2458.
26. [811]. Kirkwood JM, Manola J, Ibrahim J, Sondak V, Ernstoff MS, Rao U, Eastern Cooperative Oncology G: **A pooled analysis of eastern cooperative oncology group and intergroup trials of adjuvant high-dose interferon for melanoma**. *Clinical cancer research : an official journal of the American Association for Cancer Research* 2004, **10**(5):1670-1677.
27. [812]. Kirkwood JM, Strawderman MH, Ernstoff MS, Smith TJ, Borden EC, Blum RH: **Interferon alfa-2b adjuvant therapy of high-risk resected cutaneous melanoma: the Eastern Cooperative Oncology Group Trial EST 1684**. *Journal of clinical oncology : official journal of the American Society of Clinical Oncology* 1996, **14**(1):7-17.
28. [813]. Klemen ND, Feingold PL, Goff SL, Hughes MS, Kammula US, Yang JC, Schrump DS, Rosenberg SA, Sherry RM: **Metastasectomy Following Immunotherapy with Adoptive Cell Transfer for Patients with Advanced Melanoma**. *Annals of surgical oncology* 2017, **24**(1):135-141.
29. [814]. Larkin J, Chiarion-Sileni V, Gonzalez R, Rutkowski P, Grob J-J, Cowey CL, Lao CD, Schadendorf D, Ferrucci PF, Smylie M *et al*: **Abstract CT075: Overall survival (OS) results from a phase III trial of nivolumab (NIVO) combined with ipilimumab (IPI) in treatment-naïve patients with advanced melanoma (CheckMate 067)**. *Cancer research* 2017, **77**(13 Supplement):CT075-CT075.
30. [815]. Le DT, Durham JN, Smith KN, Wang H, Bartlett BR, Aulakh LK, Lu S, Kemberling H, Wilt C, Luber BS *et al*: **Mismatch repair deficiency predicts response of solid tumors to PD-1 blockade**. *Science* 2017, **357**(6349):409-413.
31. [816]. Lee RJ, Gremel G, Marshall A, Myers KA, Fisher N, Dunn J, Dhomen N, Corrie PG, Middleton MR, Lorigan P *et al*: **Circulating tumor DNA predicts survival in patients with resected high risk stage II/III melanoma**. *Annals of oncology : official journal of the European Society for Medical Oncology* 2017.
32. [817]. Long GV, Atkinson V, Menzies AM, Lo S, Guminski AD, Brown MP, Gonzalez MM, Diamante K, Sandhu SK, Scolyer RA *et al*: **A randomized phase II study of nivolumab or nivolumab combined with ipilimumab in patients (pts) with melanoma brain metastases (mets): The Anti-PD1 Brain Collaboration (ABC)**. *Journal of Clinical Oncology* 2017, **35**(15_suppl):9508-9508.
33. [818]. Long GV, Hauschild A, Santinami M, Atkinson V, Mandala M, Chiarion-Sileni V, Larkin J, Nyakas M, Dutriaux C, Haydon A *et al*: **Adjuvant Dabrafenib plus Trametinib in Stage III BRAF-Mutated Melanoma**. *The New England journal of medicine* 2017, **377**(19):1813-1823.
34. [819]. Long GV, Weber JS, Larkin J, Atkinson V, Grob JJ, Schadendorf D, Dummer R, Robert C, Marquez-Rodas I, McNeil C *et al*: **Nivolumab for Patients With Advanced Melanoma Treated Beyond Progression: Analysis of 2 Phase 3 Clinical Trials**. *JAMA oncology* 2017, **3**(11):1511-1519.
35. [820]. Luke JJ, Flaherty KT, Ribas A, Long GV: **Targeted agents and immunotherapies: optimizing outcomes in melanoma**. *Nature reviews Clinical oncology* 2017, **14**(8):463-482.
36. [821]. Maurichi A, Miceli R, Camerini T, Mariani L, Patuzzo R, Ruggeri R, Gallino G, Tolomio E, Tragni G, Valeri B *et al*: **Prediction of survival in patients with thin melanoma: results from a multi-institution study**. *Journal of clinical oncology : official journal of the American Society of Clinical Oncology* 2014, **32**(23):2479-2485.
37. [822]. McArthur GA, Maio M, Arance A, Nathan P, Blank C, Avril MF, Garbe C, Hauschild A, Schadendorf D, Hamid O *et al*: **Vemurafenib in metastatic melanoma patients with brain metastases: an open-label, single-arm, phase 2, multicentre study**. *Annals of oncology : official journal of the European Society for Medical Oncology* 2017, **28**(3):634-641.
38. [823]. McGranahan N, Furness AJ, Rosenthal R, Ramskov S, Lyngaa R, Saini SK, Jamal-Hanjani M, Wilson GA, Birkbak NJ, Hiley CT *et al*: **Clonal neoantigens elicit T cell immunoreactivity and sensitivity to immune checkpoint blockade**. *Science* 2016, **351**(6280):1463-1469.
39. [824]. Ollila DW, Lopez NE, Hsueh EC: **Metastasectomy for Stage IV Melanoma in the Era of Effective Systemic Agents**. *Critical reviews in oncogenesis* 2016, **21**(1-2):37-55.
40. [825]. Parakh S, Park JJ, Mendis S, Rai R, Xu W, Lo S, Drummond M, Rowe C, Wong A, McArthur G *et al*: **Efficacy of anti-PD-1 therapy in patients with melanoma brain metastases**. *British journal of cancer* 2017, **116**(12):1558-1563.
41. [826]. Puzanov I, Diab A, Abdallah K, Bingham CO, Brogdon C, Dadu R, Hamad L, Kim S, Lacouture ME, LeBoeuf NR *et al*: **Managing toxicities associated with immune checkpoint inhibitors: consensus recommendations from the Society for Immunotherapy of Cancer (SITC) Toxicity Management Working Group**. *Journal for ImmunoTherapy of Cancer* 2017, **5**(1):95.
42. [827]. Reck M, Rodriguez-Abreu D, Robinson AG, Hui R, Csoszi T, Fulop A, Gottfried M, Peled N, Tafreshi A, Cuffe S *et al*: **Pembrolizumab versus Chemotherapy for PD-L1-Positive Non-Small-Cell Lung Cancer**. *The New England journal of medicine* 2016, **375**(19):1823-1833.
43. [828]. Rizvi NA, Hellmann MD, Snyder A, Kvistborg P, Makarov V, Havel JJ, Lee W, Yuan J, Wong P, Ho TS *et al*: **Cancer immunology. Mutational landscape determines sensitivity to PD-1 blockade in non-small cell lung cancer**. *Science* 2015, **348**(6230):124-128.
44. [829]. Snyder A, Makarov V, Merghoub T, Yuan J, Zaretsky JM, Desrichard A, Walsh LA, Postow MA, Wong P, Ho TS *et al*: **Genetic basis for clinical response to CTLA-4 blockade in melanoma**. *The New England journal of medicine* 2014, **371**(23):2189-2199.
45. [830]. Spain L, Diem S, Larkin J: **Management of toxicities of immune checkpoint inhibitors**. *Cancer treatment reviews* 2016, **44**:51-60.
46. [831]. Sunshine JC, Nguyen PL, Kaunitz GJ, Cottrell TR, Berry S, Esandrio J, Xu H, Ogurtsova A, Bleich KB, Cornish TC *et al*: **PD-L1 Expression in Melanoma: A Quantitative Immunohistochemical Antibody Comparison**. *Clinical cancer research : an official journal of the American Association for Cancer Research* 2017, **23**(16):4938-4944.
47. [832]. Tarhini AA, Lee SJ, Hodi FS, Rao UNM, Cohen GI, Hamid O, Hutchins LF, Sosman JA, Kluger HM, Sondak VK *et al*: **A phase III randomized study of adjuvant ipilimumab (3 or 10 mg/kg) versus high-dose interferon alfa-2b for resected high-risk melanoma (U.S. Intergroup E1609): Preliminary safety and efficacy of the ipilimumab arms**. *Journal of Clinical Oncology* 2017, **35**(15_suppl):9500-9500.
48. [833]. Tawbi HA-H, Forsyth PAJ, Algazi AP, Hamid O, Hodi FS, Moschos SJ, Khushalani NI, Gonzalez R, Lao CD, Postow MA *et al*: **Efficacy and safety of nivolumab (NIVO) plus ipilimumab (IPI) in patients with melanoma (MEL) metastatic to the brain: Results of the phase II study CheckMate 204**. *Journal of Clinical Oncology* 2017, **35**(15_suppl):9507-9507.
49. [834]. Vilain RE, Menzies AM, Wilmott JS, Kakavand H, Madore J, Guminski A, Liniker E, Kong BY, Cooper AJ, Howle JR *et al*: **Dynamic Changes in PD-L1 Expression and Immune Infiltrates Early During Treatment Predict Response to PD-1 Blockade in Melanoma**. *Clinical cancer research : an official journal of the American Association for Cancer Research* 2017, **23**(17):5024-5033.
50. [835]. Weber J, Mandala M, Del Vecchio M, Gogas HJ, Arance AM, Cowey CL, Dalle S, Schenker M, Chiarion-Sileni V, Marquez-Rodas I *et al*: **Adjuvant Nivolumab versus Ipilimumab in Resected Stage III or IV Melanoma**. *The New England journal of medicine* 2017, **377**(19):1824-1835.
51. [836]. Wolchok JD, Hoos A, O'Day S, Weber JS, Hamid O, Lebbe C, Maio M, Binder M, Bohnsack O, Nichol G *et al*: **Guidelines for the evaluation of immune therapy activity in solid tumors: immune-related response criteria**. *Clinical cancer research : an official journal of the American Association for Cancer Research* 2009, **15**(23):7412-7420.
52. [837]. Wong SL, Balch CM, Hurley P, Agarwala SS, Akhurst TJ, Cochran A, Cormier JN, Gorman M, Kim TY, McMasters KM *et al*: **Sentinel lymph node biopsy for melanoma: American Society of Clinical Oncology and Society of Surgical Oncology joint clinical practice guideline**. *Journal of clinical oncology : official journal of the American Society of Clinical Oncology* 2012, **30**(23):2912-2918.
53. [838]. Yang JC, Abad J, Sherry R: **Treatment of oligometastases after successful immunotherapy**. *Semin Radiat Oncol* 2006, **16**(2):131-135.
